# Supplementary material for: Microtubule-binding protein MAP1B regulates interstitial axon branching of cortical neurons via the tubulin tyrosination cycle
Source: EMBO J. 2024 Feb 22;43(7):5. doi: 10.1038/s44318-024-00050-3 (PMC10987652; doi:10.1038/s44318-024-00050-3)
Supplement: Supplementary file 1 — Appendix [file 44318_2024_50_MOESM1_ESM.pdf]

APPENDIX

Microtubule-binding Protein MAP1B Regulates Interstitial Axon Branching of Cortical Neurons via the Tubulin Tyrosination Cycle

Jakub Ziak, Joelle Dorskind, Brian Trigg, Sriram Sudarsanam, Xinyu Jin,

Randal Hand and Alex L. Kolodkin

Table of contents:

Appendix Figure S1............................ 2
Appendix Figure S2............................ 4
Appendix Figure S3............................ 6
Appendix Figure S4............................ 8
Appendix Figure S5............................ 10
Appendix Figure S6............................ 12
Appendix Figure S7............................ 14
Appendix Figure S8............................ 16
Appendix Figure S9............................ 18
Appendix Figure S10........................... 20
Appendix Figure S11........................... 22

Appendix Figure S1

A Complete data for Figure 1B

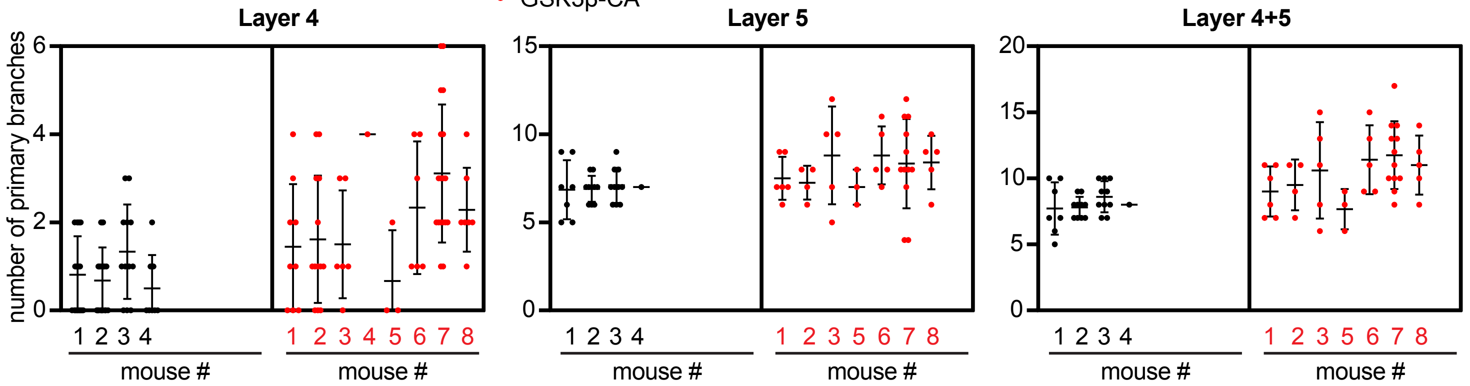

B Complete data for Figure 1C

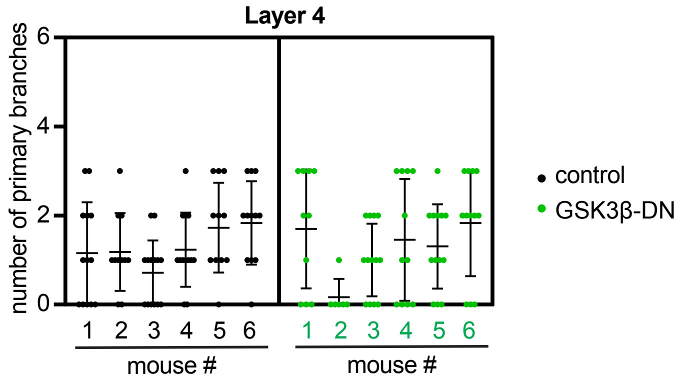

C Complete data for Figure 1D & Figure EV1F

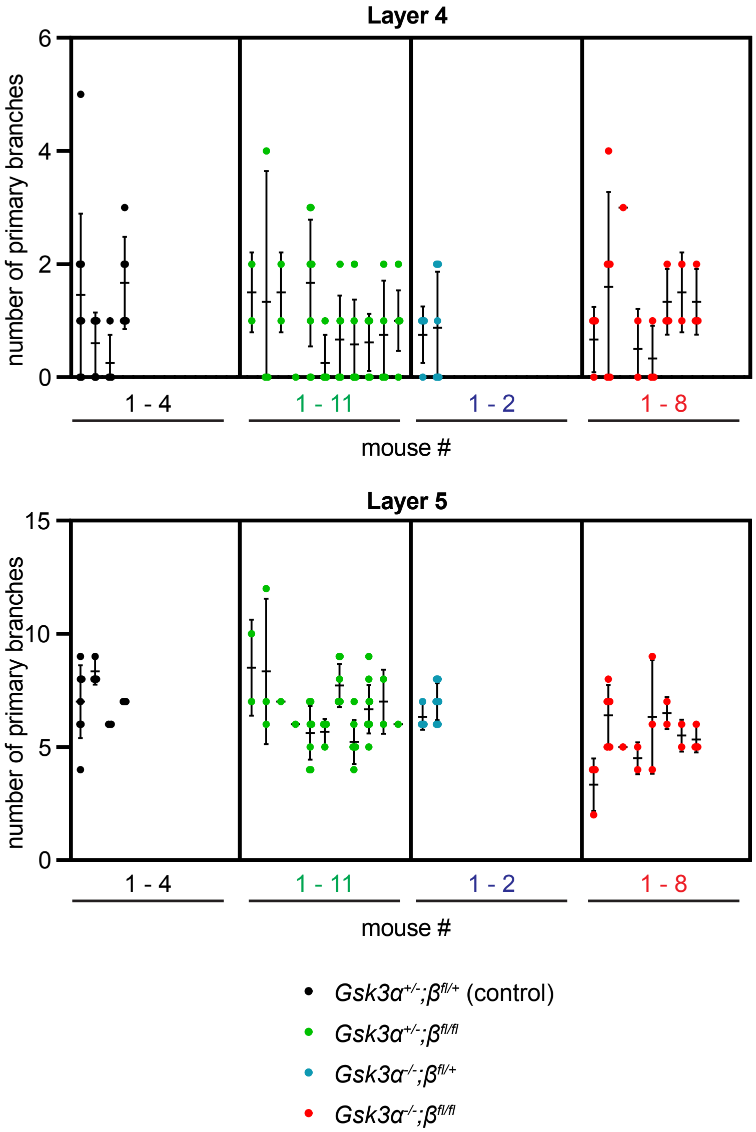

D Complete data for Figure EV1B

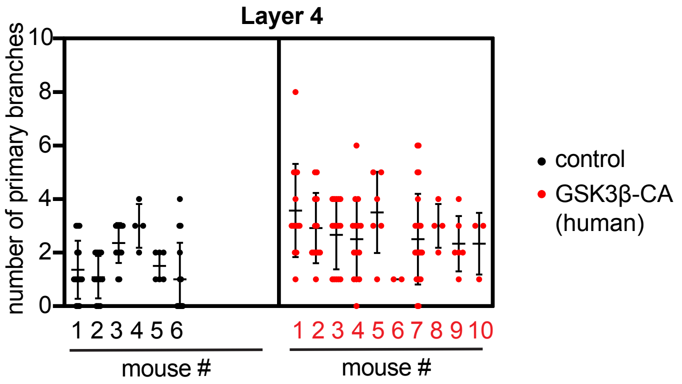

E Complete data for Figure EV1D

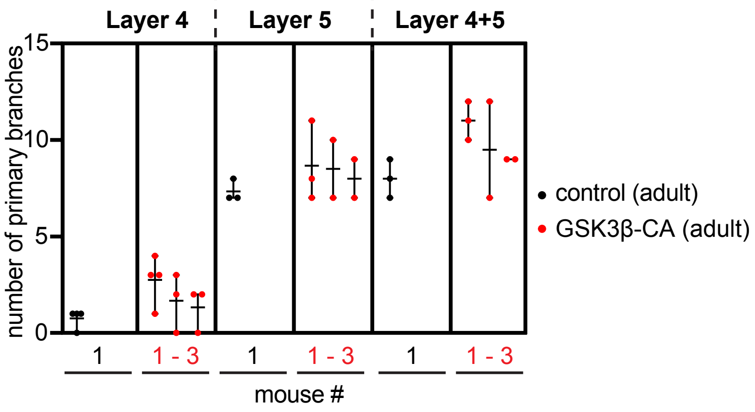

Appendix Figure S1: Complete data for Figure 1 and Figure EV1

(A) Complete data from interstitial axon branching analysis in mice overexpressing GSK3β-CA, related to Fig 1B. For this plot and every subsequent plot, each dot represents a single neuron. Each column represents a single animal; mean ± SD is plotted.

(B) Complete data from interstitial axon branching analysis in mice overexpressing GSK3β-DN, related to Fig 1C.

(C) Complete data from interstitial axon branching analysis in *Gsk3α* and *Gsk3β* knockout mice, related to Fig 1E & Fig EV1F.

(D) Complete data from the original screen: interstitial axon branching quantification in mice overexpressing human GSK3β-CA, related to Fig EV1B.

(E) Complete data from interstitial axon branching analysis in adult mice overexpressing GSK3β-CA, related to Fig EV1D.

Appendix Figure S2

A Complete data for Figure 2A

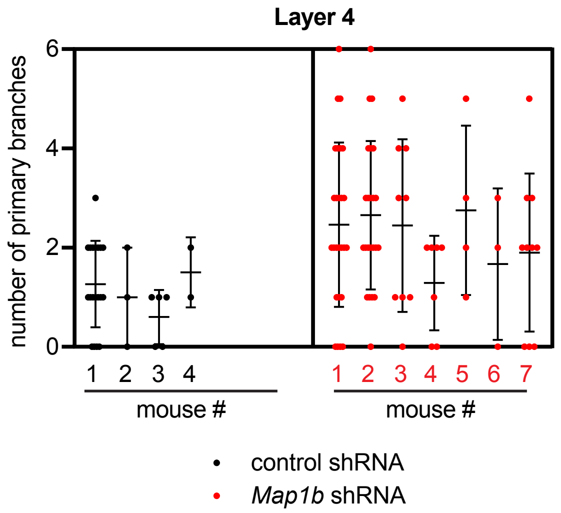

B Complete data for Figure 2C

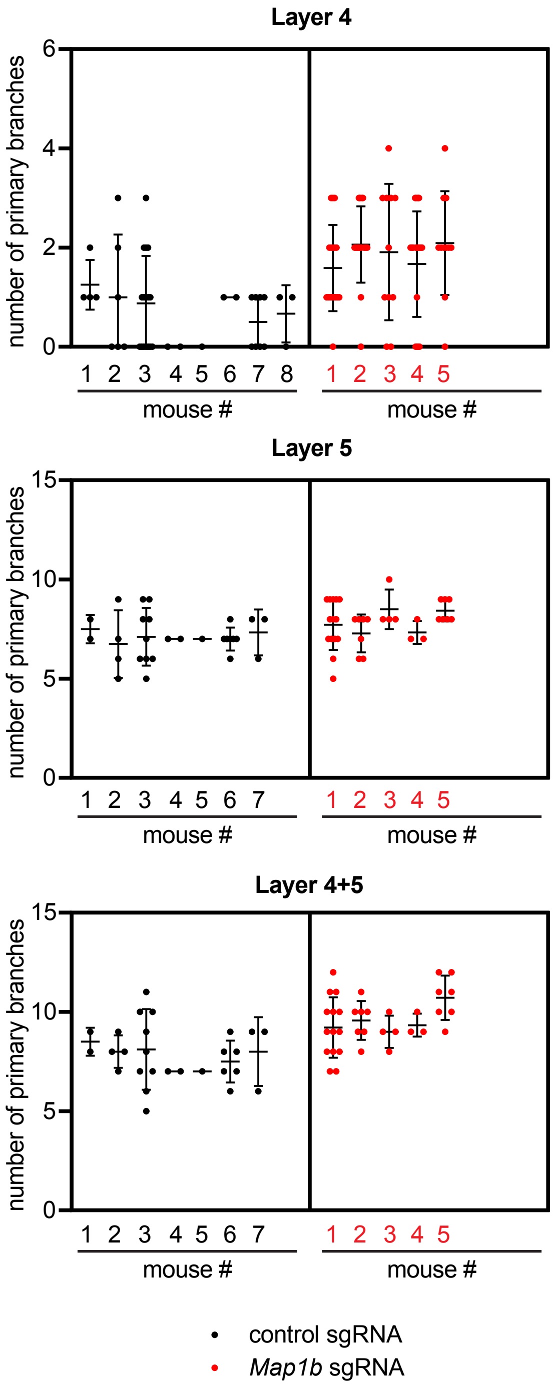

C Complete data for Figure 2E

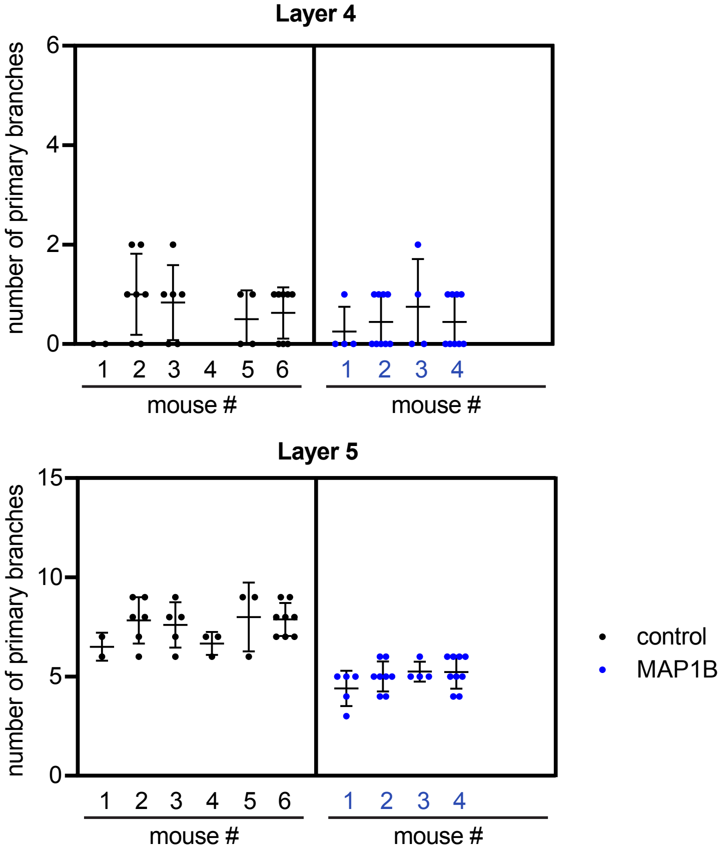

Appendix Figure S2: Complete data for Figure 2

(A) Complete data from interstitial axon branching analysis in mice with *Map1b* shRNA-mediated knockdown, related to Fig 2A.

(B) Complete data from interstitial axon branching analysis in mice with *Map1b* sgRNA-mediated knockdown, related to Fig 2C.

(C) Complete data from interstitial axon branching analysis in mice overexpressing naïve MAP1B, related to Fig 2E.

Appendix Figure S3

A Complete data for Figure 3A

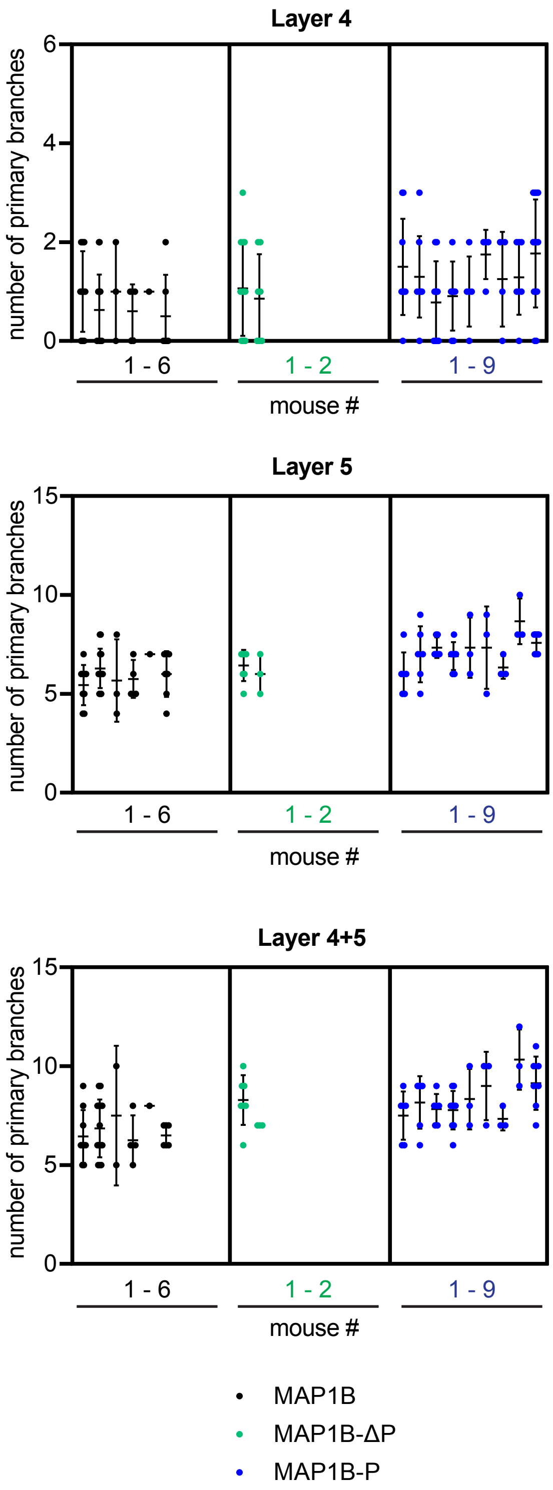

B Complete data for Figure 3C

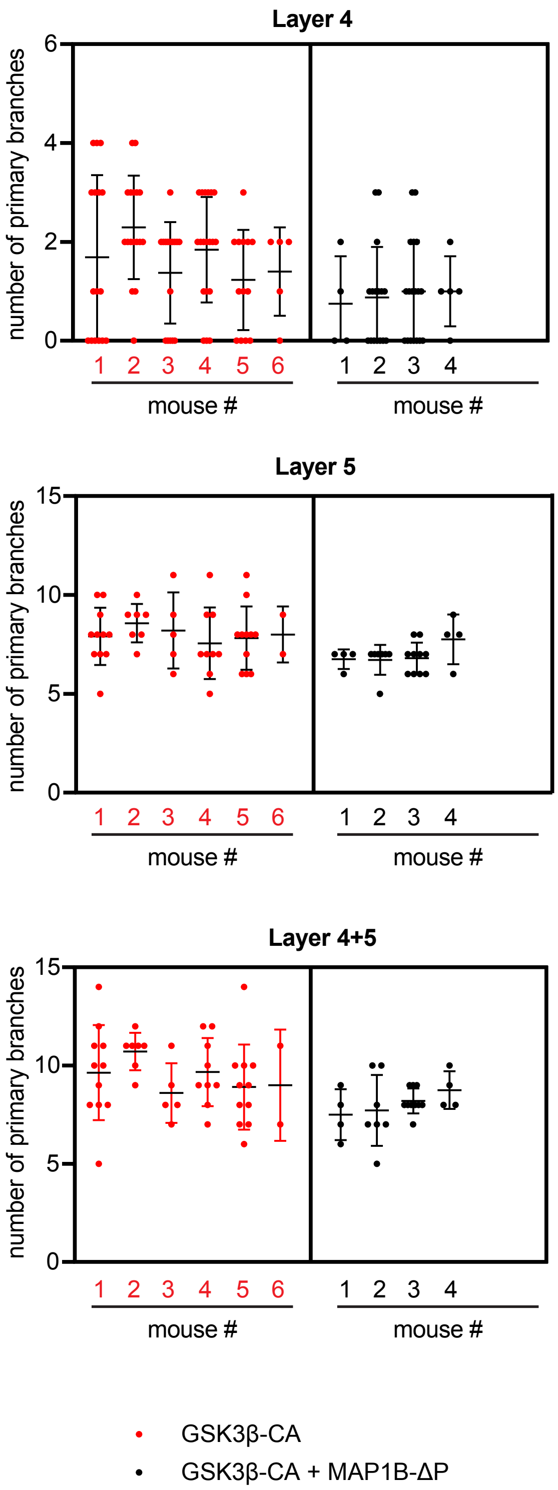

Appendix Figure S3: Complete data for Figure 3

(A) Complete data from interstitial axon branching analysis in mice overexpressing naïve MAP1B, MAP1B-ΔP and MAP1B-P, related to Fig 3A

(B) Complete data from GSK3β-MAP1B rescue experiment, related to Fig 3C.

Appendix Figure S4

A Complete data for Figure 4C

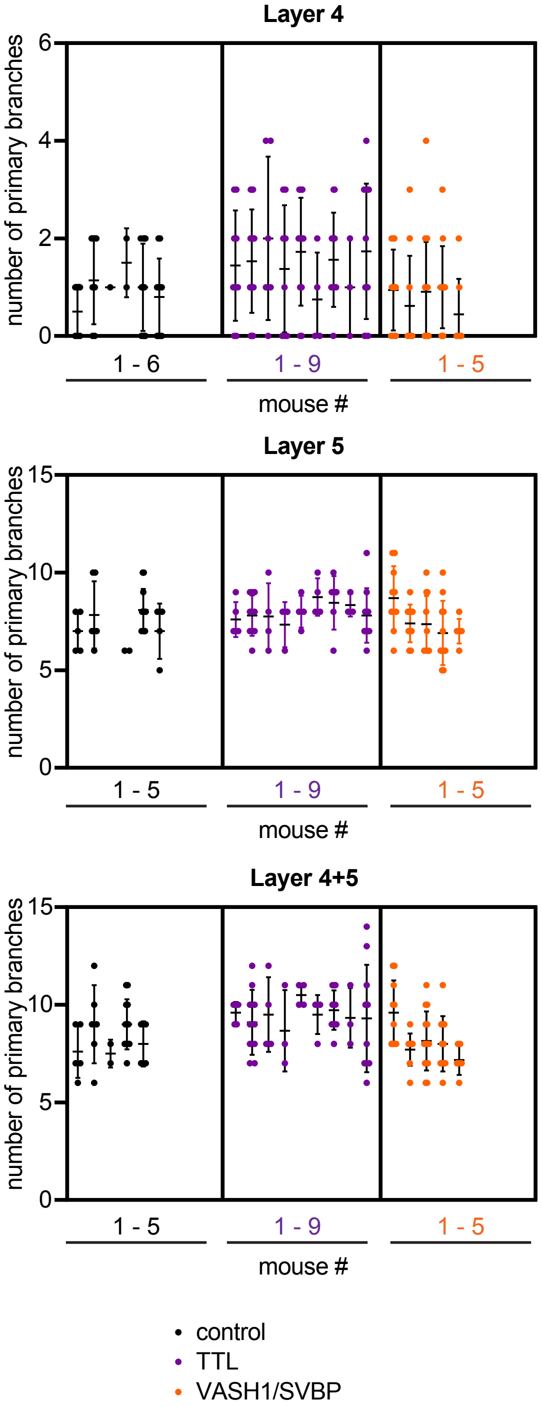

B Complete data for Figure 4F

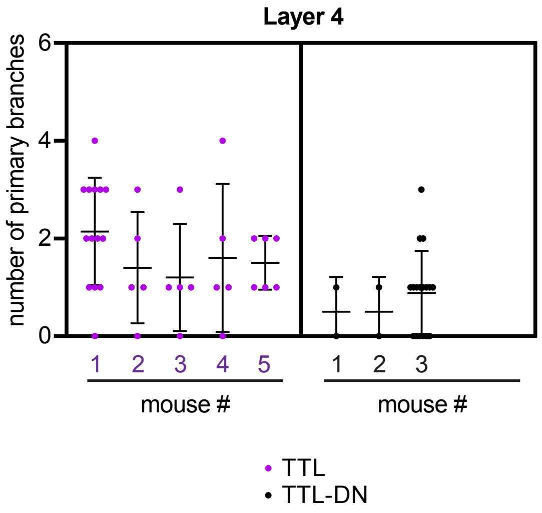

Appendix Figure S4: Complete data for Figure 4

(A) Complete data from interstitial axon branching analysis in mice overexpressing TTL or VASH1/SVBP, related to Fig 4C.

(B) Complete data from interstitial axon branching analysis in mice overexpressing TTL or TTL-DN, related to Fig 4F.

Appendix Figure S5

A Complete data for Figure 5A

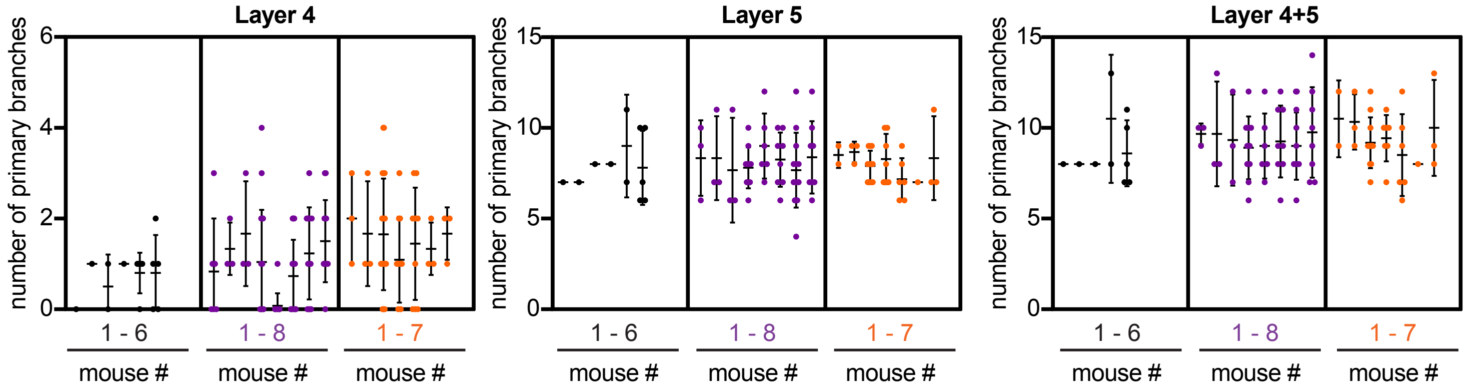

• control sgRNA
• *Ttl* sgRNA
• *Svbp* sgRNA

B Complete data for Figure 6C

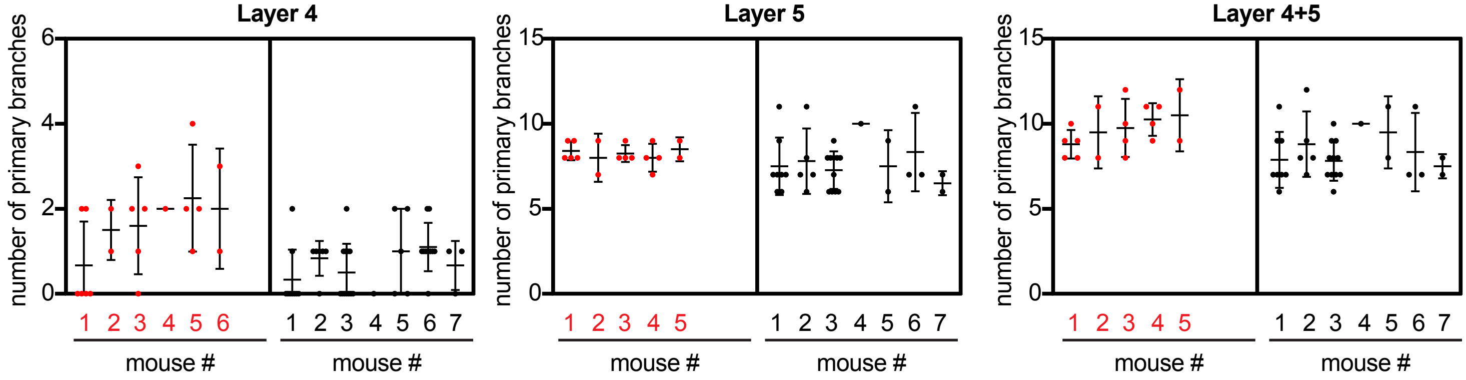

• GSK3β-CA + control sgRNA
• GSK3β-CA + *Ttl* sgRNA

C Complete data for Figure EV5C

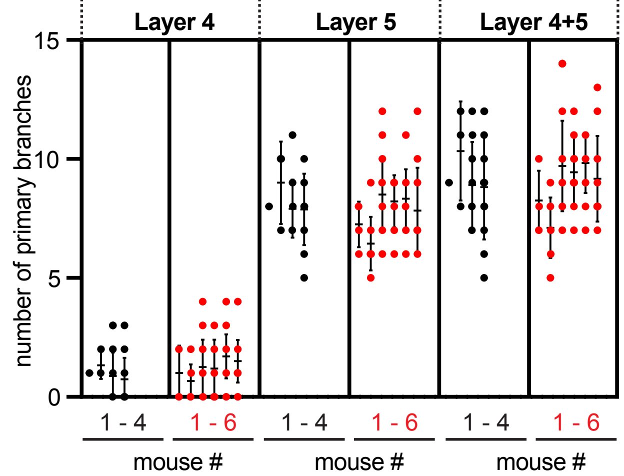

• control sgRNA
• *Svbp* + *Matcap* sgRNA

D Complete data for Figure 6F

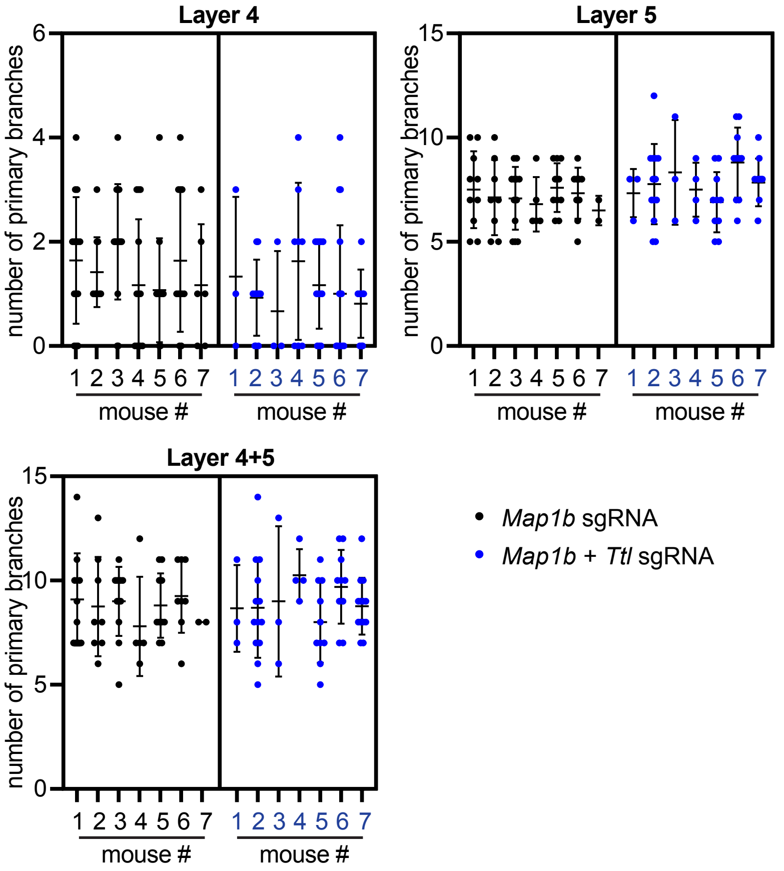

Appendix Figure S5: Complete data for Figure 5, Figure EV5 and Figure 6.

(A) Complete data from interstitial axon branching analysis in mice with sgRNA-mediated knockdown of *Ttl* and *Svbp*, related to Fig 5A.

(B) Complete data from GSK3β-TTL rescue experiment, related to Fig 6C.

(C) Complete data from combined *Svbp* and *Matcap* sgRNA-mediated knockdown, related to Fig EV5C.

(D) Complete data from MAP1B-TTL interaction experiment, related to Fig 6F.

Appendix Figure S6

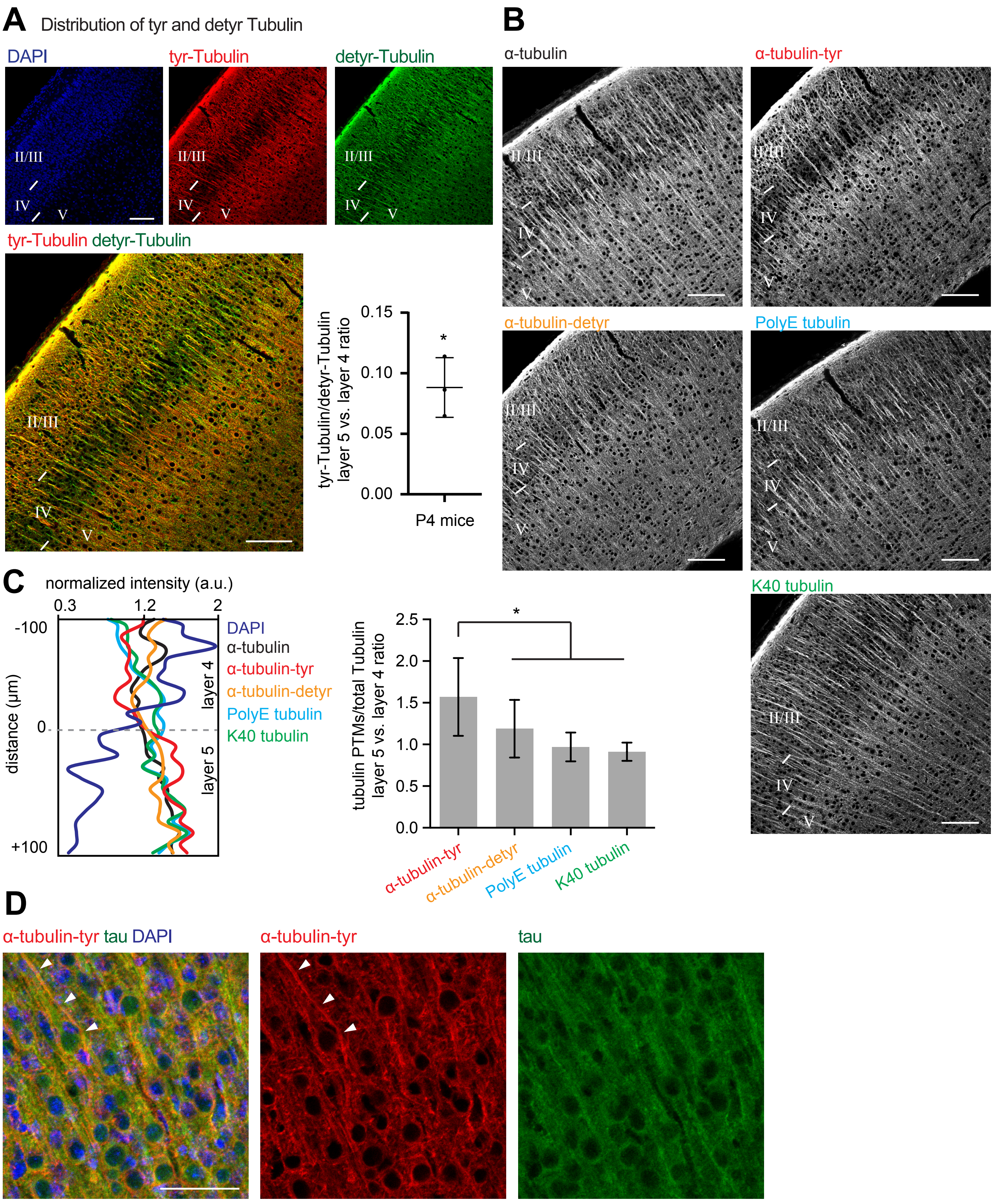

Appendix Figure S6. Distribution of tubulin posttranslational modifications in the developing cortex.

(A) Immunostaining of P4 cortex with an antibody directed against tyrosinated tubulin (Tyr-Tubulin, YL1/2 clone) and detyrosinated tubulin (detyr-Tubulin, clone RM4407). Note significant increase in the Tyr-T signal between layer 4 and 5. Graph on the right shows relative increase of Tyr-T/detyr-T signal between layers 5 and 4, n=3 mice. * $p<0.05$, paired t-test. Scale bars: 100 µm.

(B) Immunostaining of α-tubulin and four different tubulin posttranslational modifications. Scale bars: 100 µm.

(C) Left: Traces of normalized fluorescent intensities of individual immunostainings from (B); Right: relative ratio between layer 5 and layer 4 for each condition (individual PTMs were normalized to total tubulin levels). Note that only tubulin tyrosination increases significantly. One-way ANOVA with post-hoc Dunnett's test, * $p<0.05$

(D) Immunolabeling of tyr-tubulin and tau shows that tyr-tubulin is present in both axons (tau^+ structures) and dendrites (arrowheads, weaker tau signal). Scale bar: 50 µm

Appendix Figure S7

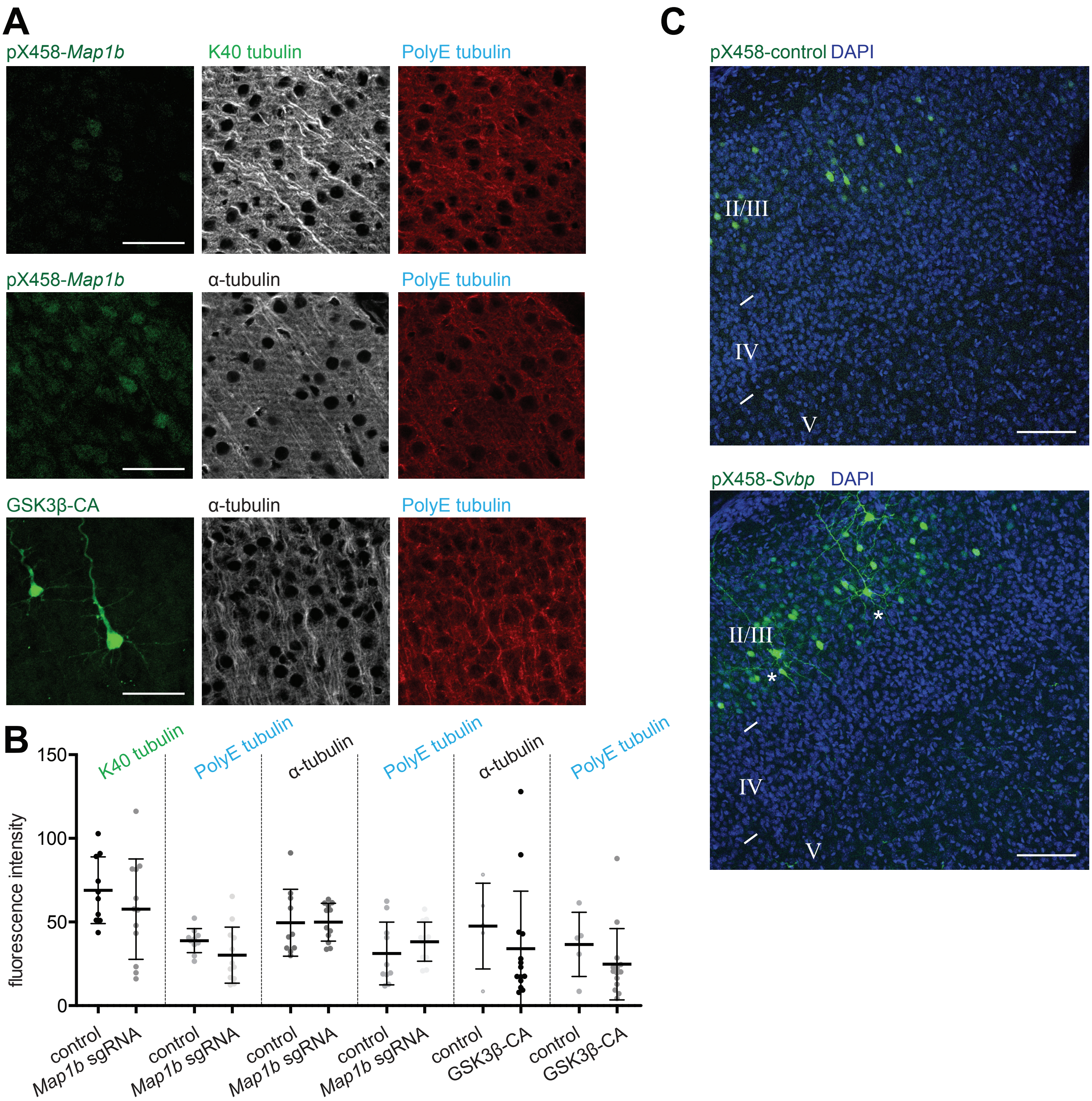

Appendix Figure S7. GSK3β/MAP1B signaling does not influence tubulin polyglutamylation and K40 acetylation.

(A) Layer 2/3 CPNs were in utero electroporated at E15.5 with pX458 plasmids to delete MAP1B. Brains were analyzed by P7. There are no changes in the amount of K40 acetylation or tubulin polyglutamylation in GFP^{+} neurons as compared to GFP^{-} cells. Overexpression of GSK3β-CA does not change the amount of polyglutamylated tubulins. Scale bars: 50 µm

(B) Quantification reveals comparable fluorescence in cell bodies under both MAP1B knockout and GSK3β-CA overexpression conditions.

(C) Mild neuronal migration defects after deletion of SVBP. Layer 2/3 CPNs were in utero electroporated at E15.5 with pX458 plasmids. Brains were analyzed at P14 (see Fig 5 for details). Note GFP+ cells at the border between layer III and IV (asterisk) after SVBP deletion. Scale bars: 100 µm.

Appendix Figure S8

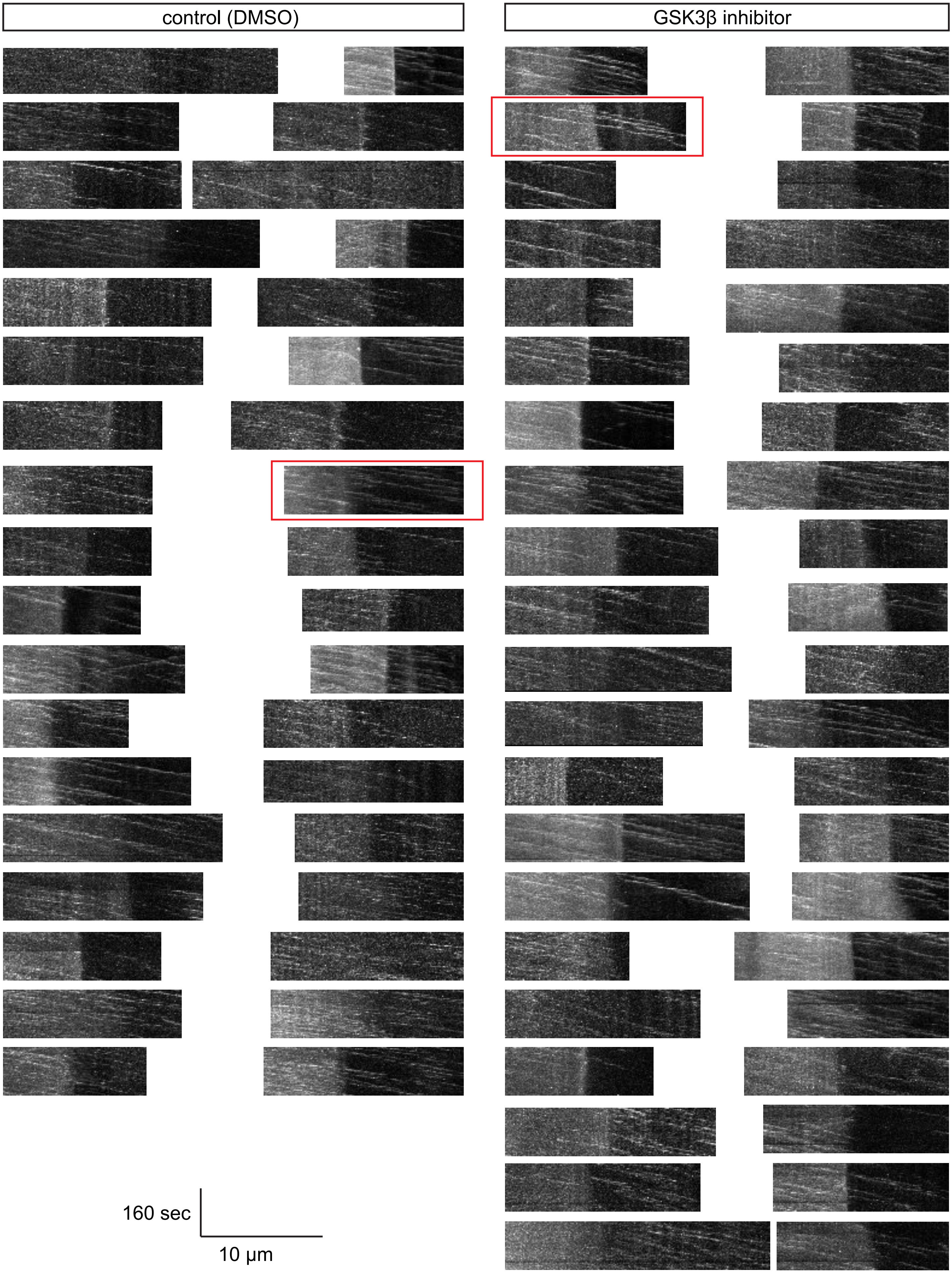

Appendix Figure S8. All EB3-GFP kymographs for Figure 6.

Complete kymographs depicting individual EB3-GFP particles that were scored in this experiment, under both control (DMSO) conditions and GSK3β inhibition. Red rectangles highlight kymographs shown in the Figure 6.

Appendix Figure S9

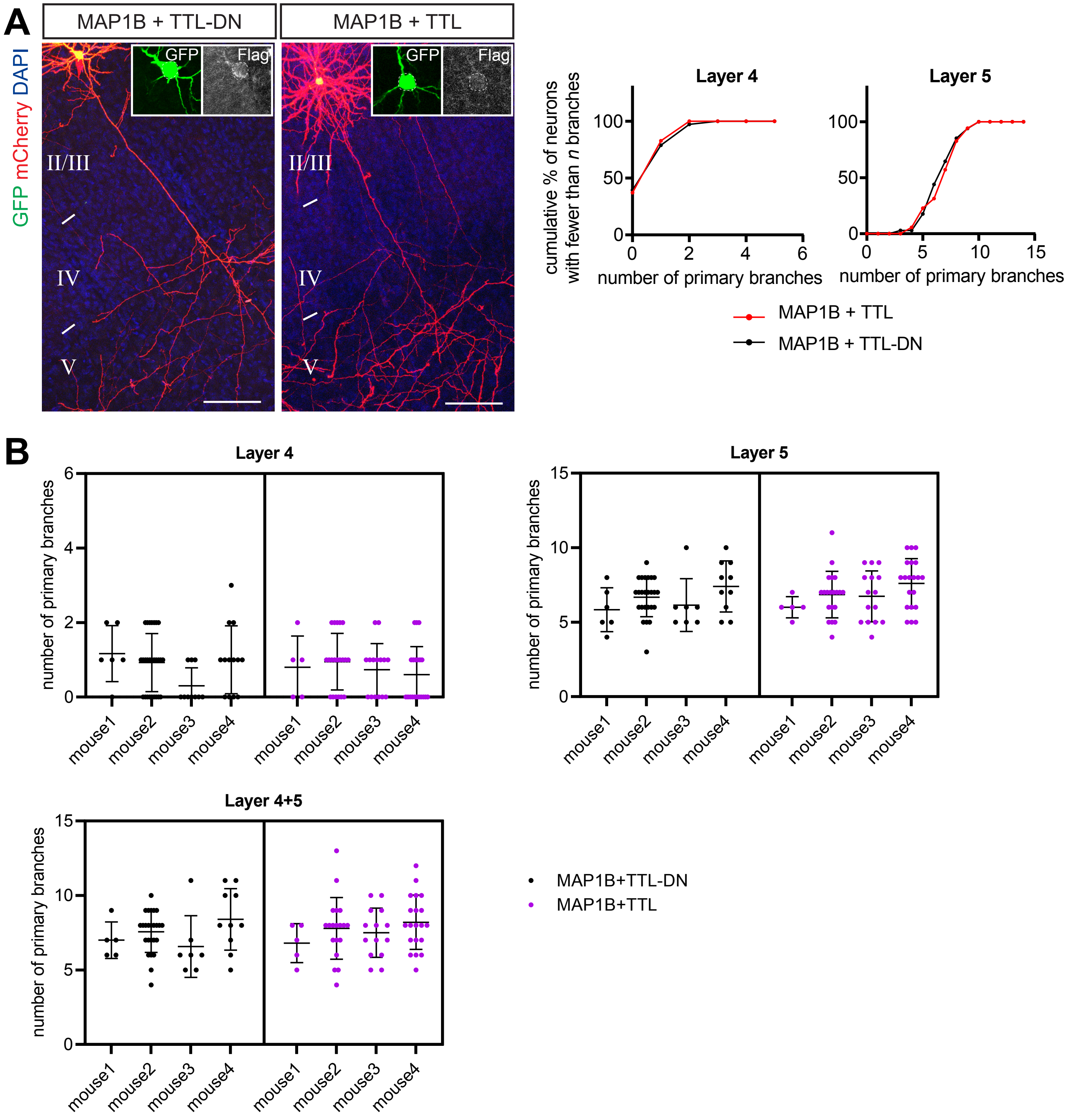

Appendix Figure S9. TTL does not release MAP1B-induced interstitial axon branching inhibition.

(A) Testing the interaction between MAP1B and TTL. MAP1B was overexpressed together with TTL-DN (control group) or TTL (experimental group). Note that in neurons overexpressing MAP1B, TTL is not sufficient to promote interstitial axon branching. Data analysis and presentation as in Figure 1. Scale bars: 100 μm.

(B) Complete data for the experiment shown in the panel A in this figure.

Appendix Figure S10

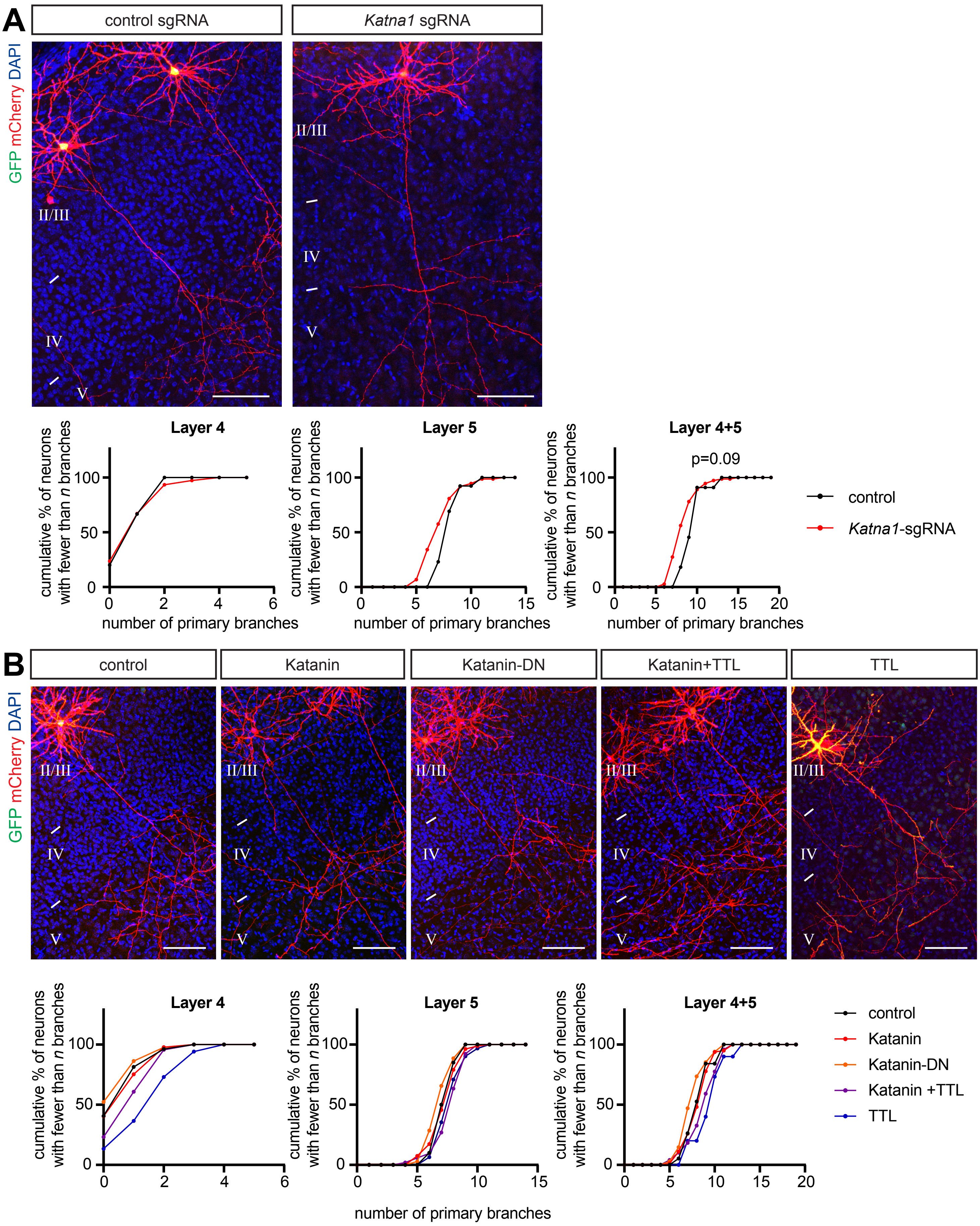

Appendix Figure S10. Katanin is neither sufficient nor necessary for interstitial axon branching.

(A) p60 Katanin is not necessary for interstitial axon branching. Removal of *Katna1* via CRISPR/Cas9 in layer 2/3 CPNs does not influence axon collateral branching. Data analysis and presentation as in Figure 1. Scale bars: 100 µm.

(B) Katanin is not sufficient to induce or suppress interstitial axon branching. Wild-type and dominant-negative (ref) p60 subunit of Katanin was expressed in layer 2/3 CPNs using our bipartite system. In addition, TTL does not prime neurons towards increased interstitial axon branching. Data analysis and presentation as in Figure 1. Scale bars: 100 µm.

Appendix Figure S11

A Complete data for Appendix Figure S10A

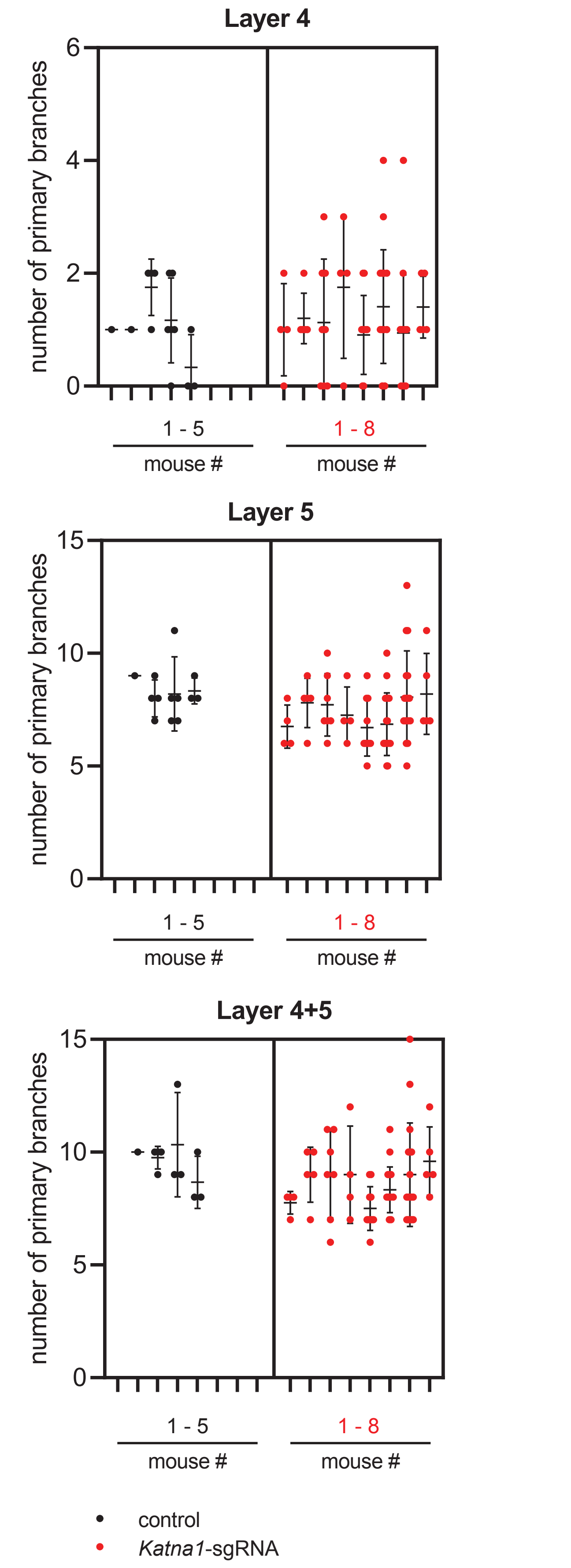

B Complete data for Appendix Figure S10B

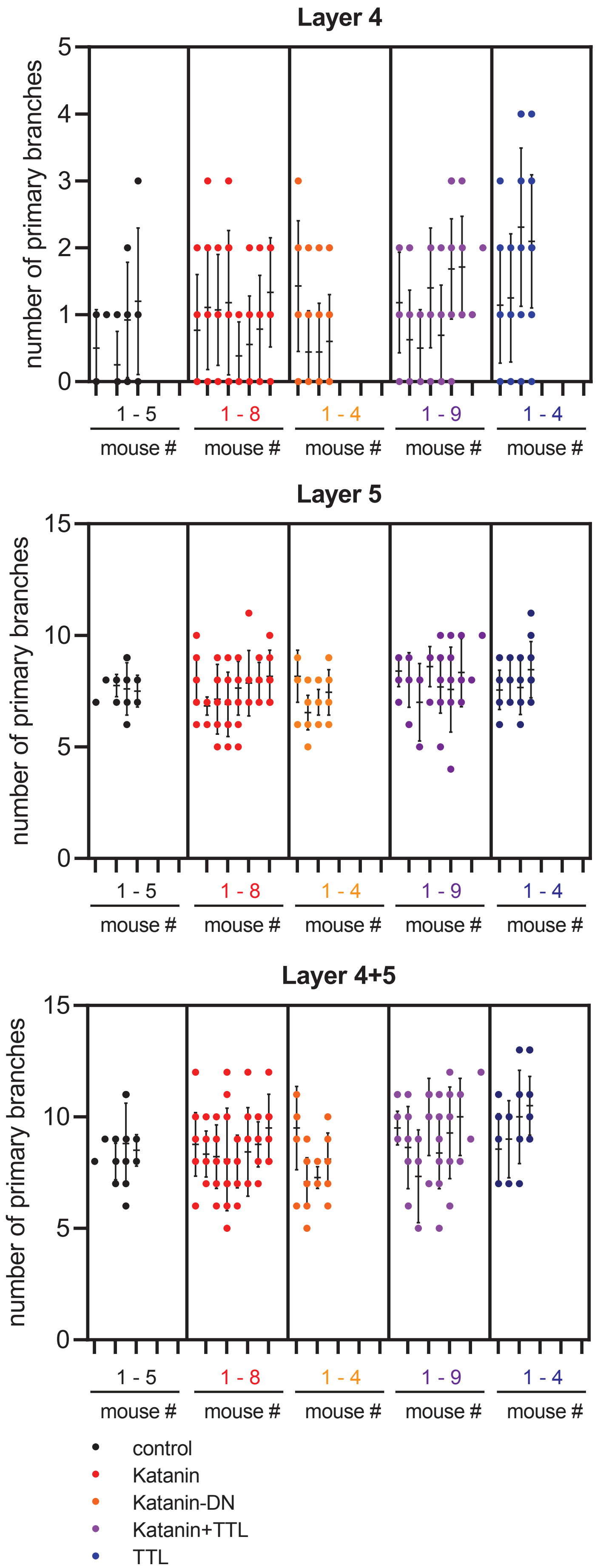

Appendix Figure S11: Complete data for Appendix Figure S10.

(A) Complete data from interstitial axon branching analysis in mice with sgRNA-mediated knockdown of *Katna1*, related to Appendix Fig S10A.

(B) Complete data from Katanin, Katanin-DN and TTL overexpression experiment, related to Appendix Fig S10C.
