# Supplementary material for: Microtubule-binding protein MAP1B regulates interstitial axon branching of cortical neurons via the tubulin tyrosination cycle
Source: EMBO J. 2024 Feb 22;43(7):5. doi: 10.1038/s44318-024-00050-3 (PMC10987652; doi:10.1038/s44318-024-00050-3)
Supplement: Supplementary file 2 — Table EV1 [file 44318_2024_50_MOESM2_ESM.pdf]

Table EV1.

Table presents mouse strain/mouse lines, plasmids used for in utero electroporations, age of analyses, number of animals and neurons quantified, statistical tests, p values, and details of the post-hoc tests (where applicable).

figures	mouse strain & genotype	plasmids	age of analysis	number of animals	number of neurons	number of neurons per animal	Statistics
Figure 1B	WT CD1	control: pCAG-CreERT2, pEF1-Flex-FlpO, pCAG-FSF-mCherry, pCAG-FSF-GFP	P14	4	66	8-25 neurons/animal	L4 p=0.0124, L5 p=0.0057, L4+5 p=0.0128
	WT CD1	experimental: pCAG-CreERT2, pEF1-Flex-FlpO, pCAG-FSF-mCherry, pCAG-FSF-GSK3BCA-IRES-GFP	P14	8	63	1-18 neurons/animal	nested t-test (mixed model)
Figure 1C	WT CD1	control: pCAG-CreERT2, pEF1-Flex-FlpO, pCAG-FSF-mCherry, pCAG-FSF-GFP	P14	6	74	10-14 neurons/animal	L4, p=0.9754
	WT CD1	experimental: pCAG-CreERT2, pEF1-Flex-FlpO, pCAG-FSF-mCherry, pCAG-FSF-GSK3BDN-IRES-GFP	P14	6	64	5-13 neurons/animal	nested t-test (mixed model)
Figure 1D, E	*GSK3 α+/-;βfl/+ (control 'het'), GSK3α+/-;βfl/fl and GSK3α+/+;βfl/fl ('Gsk3B KO'), GSK3α-/-;βfl/fl ('double KO')*	pCAG-CreERT2, pEF1-Flex-FlpO, pCAG-FSF-mCherry	P14+P21	22 (4xhet, 11xGsk3B KO, 8x double KO)	131 (28xhet, 71xGsk3B KO, 25x double KO)	1-13 neurons/animal	nested ANOVA (mixed model), L4 p=0.5462, L5 p=0.039, L4+L5 p=0.2636
Figure 1F	WT CD1		P4	5		4-8 slices/animal	Paired t test, p=0.0006
Figure 2A, B	WT CD1	control: pCAG-GFP, pPrime-dsRed-miR30-shRNA scrambled	P14	4	29	3-19 neurons/animal	L4 p=0.0038
	WT CD1	experimental: pCAG-GFP, pPrime-dsRed-miR30-shRNA-MAP1B	P14	7	82	4-26 neurons/animal	nested t-test (mixed model)
Figure 2C, D	WT CD1	control: pCAG-CreERT2, pTRE-Flex-FlpO, pCAG-FSF-mCherry-tTA, 2x pX458-sgRNA-control2x-Cas9-T2A-GFP	P14	8	42	1-16 neurons/animal	L4 p<0.0001, L5 p=0.0074, L4+L5 p=0.001
	WT CD1	experimental: pCAG-CreERT2, pTRE-Flex-FlpO, pCAG-FSF-mCherry-tTA, 2x pX458-sgRNA-MAP1B2x-Cas9-T2A-GFP	P14	5	76	11-21 neurons/animal	nested t-test (mixed model)
Figure 2E, F	WT CD1	control: pCAG-CreERT2, pEF1-Flex-FlpO, pCAG-FSF-mCherry, pCAG-FSF-GFP	P21	6	27	2-8 neurons/animal	L4 p=0.166, L5 p<0.001
	WT CD1	experimental: pCAG-CreERT2, pEF1-Flex-FlpO, pCAG-FSF-MAP1B-Flag, pCAG-FSF-GFP	P21	4	25	3-8 neurons/animal	nested t-test (mixed model)
Figure 3A, B	WT CD1	control: pCAG-CreERT2, pEF1-Flex-FlpO, pCAG-FSF-MAP1B-Flag, pCAG-FSF-GFP	P14	6	41	1-16 neurons/animal	L4 p=0.0346, L5 p=0.0051, L4+L5 p=0.0033
	WT CD1	experimental: pCAG-CreERT2, pEF1-Flex-FlpO, pCAG-FSF-MAP1B-dP-Flag, pCAG-FSF-GFP	P14	2	22	7-15 neurons/animal	nested ANOVA (mixed model) with post-hoc Dunnet's test
	WT CD1	experimental: pCAG-CreERT2, pEF1-Flex-FlpO, pCAG-FSF-MAP1B-P-Flag, pCAG-FSF-GFP	P14	9	91	4-13 neurons/animal	additional analysis shown in Fig EV3E: L4 p=0.0184, L5 p=0.2456
Figure 3C, D	WT CD1	control: pCAG-CreERT2, pEF1-Flex-FlpO, pCAG-FSF-mCherry, pCAG-FSF-GSK3BCA-IRES-GFP	P14	6	86	5-19 neurons/animal	L4 p=0.015, L5 p=0.0024, L4+L5 p=0.0014
	WT CD1	experimental: pCAG-CreERT2, pEF1-Flex-FlpO, pCAG-FSF-mCherry, pCAG-FSF-GSK3BCA-IRES-GFP, pCAG-FSF-MAP1B-dP-Flag	P14	5	48	4-18 neurons/animal	nested t-test (mixed model)
Figure 4B	WT CD1	control: pCAG-GFP, pMini-TagRFP-T_A1aY1, pX330-2xsgRNARosa26-Cas9	P4	4	15	2, 1, 6, 6 neurons/animal	L4 vs L5 signal, p=0.0181, Wilcoxon test
Figure 4C, D	WT CD1	control: pCAG-CreERT2, pEF1-Flex-FlpO, pCAG-FSF-mCherry, pCAG-FSF-GFP	P14	5	42	1-16 neurons/animal	L4 p<0.0001, L5 p=0.23, L4+L5 p=0.0074
	WT CD1	experimental: pCAG-CreERT2, pEF1-Flex-FlpO, pCAG-FSF-mCherry, pCAG-FSF-VASH1-IRES-GFP, pCAG-FSF-SVBP-IRES-GFP	P14	6	87	9-22 neurons/animal	nested ANOVA (mixed model) with post-hoc Dunnet's test
	WT CD1	experimental: pCAG-CreERT2, pEF1-Flex-FlpO, pCAG-FSF-mCherry, pCAG-FSF-TTL-IRES-GFP	P14	9	87	4-18 neurons/animal	
Figure 4F, G	WT CD1	pCAG-CreERT2, pEF1-Flex-FlpO, pCAG-FSF-mCherry, pCAG-FSF-TTL-IRES-GFP	P14	5	33	5-14 neurons/animal	L4, p=0.0343
	WT CD1	pCAG-CreERT2, pEF1-Flex-FlpO, pCAG-FSF-mCherry, pCAG-FSF-TTL-DN-IRES-GFP	P14	3	21	2-17 neurons/animal	nested t-test (mixed model)
Figure 5A, B	WT CD1	control: pCAG-CreERT2, pTRE-Flex-FlpO, pCAG-FSF-mCherry-tTA, 2x pX458-sgRNA-control2x-Cas9-T2A-GFP	P14	6	15	1-5 neurons/animal	L4 p=0.042, L5 p=0.882, L4+L5 p=0.723
	WT CD1	experimental: pCAG-CreERT2, pTRE-Flex-FlpO, pCAG-FSF-mCherry-tTA, 2x pX458-sgRNA-TTL2x-Cas9-T2A-GFP	P14	8	88	3-23 neurons/animal	nested ANOVA (mixed model) with post-hoc Dunnet's test
	WT CD1	experimental: pCAG-CreERT2, pTRE-Flex-FlpO, pCAG-FSF-mCherry-tTA, 2x pX458-sgRNA-SVBP2x-Cas9-T2A-GFP	P14	7	55	3-23 neurons/animal	
Figure 6B	WT CD1, dissociated neurons	pCAG-mCherry, pCAG-EB3-GFP	DIV4	4 wells/group	22 control, 24 inhibitor	5-7 neurons/well	unpaired t test, p=0.0002
Figure 6C, D	WT CD1	control: pCAG-CreERT2, pEF1-Flex-FlpO, pCAG-FSF-mCherry, pCAG-FSF-GSK3BCA-IRES-GFP, pX458-sgRNA control-Cas9-T2A-GFP	P14	6	20	1-6 neurons/animal	L4 p=0.0123, L5 p=0.0942, L4+L5 p=0.0021
	WT CD1	experimental: pCAG-CreERT2, pEF1-Flex-FlpO, pCAG-FSF-mCherry, pCAG-FSF-GSK3BCA-IRES-GFP, pX458-sgRNA-TTL-Cas9-T2A-GFP	P14	7	46	1-12 neurons/animal	nested t-test (mixed model)
Figure 6E, F	WT CD1	control: pCAG-CreERT2, pTRE-Flex-FlpO, pCAG-FSF-mCherry-tTA, 2x pX458-sgRNA-MAP1B2x-Cas9-T2A-GFP	P14	7	83	2-12 neurons/animal	L4 p=0.0319, L5 p=0.081, L4+L5 p=0.88
	WT CD1	experimental: pCAG-CreERT2, pTRE-Flex-FlpO, pCAG-FSF-mCherry-tTA, 2x pX458-sgRNA-MAP1B2x-Cas9-T2A-GFP, pX458-sgRNA-TTL2x-Cas9-T2A-GFP	P14	7	72	3-16 neurons/animal	nested t-test (mixed model)
Figure EV1B	WT CD1	control: pCAG-CreERT2, pEF1-Flex-FlpO, pCAG-FSF-mCherry, pCAG-FSF-GFP	P14	6	65	4-15 neurons/animal	L4, p=0.0022
	WT CD1	experimental: pCAG-CreERT2, pEF1-Flex-FlpO, pCAG-FSF-mCherry, pCAG-FSF-GSK3BCA(human)-IRES-GFP	P14	10	100	2-24 neurons/animal	nested t-test (mixed model)
Figure EV1C	WT CD1	control: pCAG-CreERT2, pEF1-Flex-FlpO, pCAG-FSF-mCherry, pCAG-FSF-GFP	P14	same as Figure EV1B			n/a
	WT CD1	experimental: pCAG-CreERT2, pEF1-Flex-FlpO, pCAG-FSF-mCherry, pCAG-FSF-GSK3BCA-IRES-GFP	P14	same as Figure EV1B			n/a
Figure EV1D	WT CD1	control: pCAG-CreERT2, pEF1-Flex-FlpO, pCAG-FSF-mCherry, pCAG-FSF-GFP	P56	1	4	4 neurons/animal	L4 p=0.2927, L5 p=0.2996, L4+5 p=0.2431
	WT CD1	experimental: pCAG-CreERT2, pEF1-Flex-FlpO, pCAG-FSF-mCherry, pCAG-FSF-GSK3BCA-IRES-GFP	P56	3	10	3-4 neurons/animal	nested t-test (mixed model)
Figure EV1F	*GSK3 α-/-;βfl/+*	pCAG-CreERT2, pEF1-Flex-FlpO, pCAG-FSF-mCherry	P14+P21	2	19	7-12 neurons/animal	nested t-test, L4 p=0.622, L5 p=0.56, L4+5 p=0.717
Figure EV1G, H	*GSK3α+/-;βfl/fl and GSK3α+/+;βfl/fl ('Gsk3B KO')*	pCAG-CreERT2, pEF1-Flex-FlpO, pCAG-FSF-mCherry	P14+P21	3	9	2-4 neurons/animal	neurons are from the figure 1D, p = 0.03 (number of dendritic intersections at the level of AIS)
	GSK3α-/-;βfl/fl ('double KO')	pCAG-CreERT2, pEF1-Flex-FlpO, pCAG-FSF-mCherry	P14+P21	4	12	2-5 neurons/animal	nested t-test (mixed model)
Figure EV2C	*βcatenin fl/+ or APC fl/+*	pCAG-CreERT2, pEF1-Flex-FlpO, pCAG-FSF-mCherry	P14	at least 3 from each group	n/a		
Figure EV2D	WT CD1	control: pCAG-GFP, pPrime-dsRed-miR30-shRNA scrambled	P14	at least 3 from each group	n/a		
	WT CD1	experimental: pCAG-GFP, pPrime-dsRed-miR30-shRNA-Macf1 or Clasp1 or Clasp2	P14	at least 3 from each group	n/a		
Figure EV3B	WT CD1	pCAG-mCherry, pMini-donor, pX330-2xsgRNA-Cas9	P14	at least 3 from each group	n/a		
Figure EV3C, D	WT CD1	pCAG-mCherry, pMini-donor, pX330-2xsgRNA-Cas9	P4, P14	at least 3 from each group	n/a		
Figure EV3E	WT CD1	comparison of data from Fig. 3A & Fig. 2E	P14+P21				L4 p=0.0184, L5 p=0.2456, nested t-test (mixed model)
Figure EV3F	WT CD1		P4	4		5 slices/animal	unpaired t test, p=0.04
Figure EV4B	WT CD1	experimental: pCAG-CreERT2, pEF1-Flex-FlpO, pCAG-FSF-mCherry, pCAG-FSF-VASH1-IRES-GFP, pCAG-FSF-SVBP-IRES-GFP	P4	2	34	6, 28 neurons	
		experimental: pCAG-CreERT2, pEF1-Flex-FlpO, pCAG-FSF-mCherry, pCAG-FSF-TTL-IRES-GFP	P4	2	35	6, 29 neurons	unpaired t test, p=0.0055
Figure EV5A	WT CD1	pCAG-CreERT2, pEF1-Flex-FlpO, pCAG-FSF-mCherry, pCAG-FSF-TTL-IRES-GFP or pCAG-FSF-Vash-IRES-GFP+pCAG-FSF-SVBP-IRES-GFP	P14 (samples from Fig. 4C)			ctrl 10&12 neurons, SVBP 4 neurons, TTL 3 neurons	ctrl vs SVBP p=0.0093, ctrl vs TTL p=0.0225, unpaired t-test
Figure EV5B	WT CD1	2x pX458-sgRNA-TTL2x-Cas9-T2A-GFP	P7	3	18 control, 21 experi	6-13 neurons/animal	ctrl (GFP-) vs TTL (GFP+) p<0.001 unpaired t-test
	WT CD1	2x pX458-sgRNA-SVBP2x-Cas9-T2A-GFP	P7	3	21 control, 22 experi	3-13 neurons/animal	ctrl (GFP-) vs TTL (GFP+) p<0.001 unpaired t-test
Figure EV5C, D	WT CD1	control: pCAG-CreERT2, pTRE-Flex-FlpO, pCAG-FSF-mCherry-tTA, 2x pX458-sgRNA-control2x-Cas9-T2A-GFP	P14	4	55	1-27 neurons/animal	L4 p=0.008, L5 p=0.638, L4+L5 p=0.875
	WT CD1	experimental: pCAG-CreERT2, pTRE-Flex-FlpO, pCAG-FSF-mCherry-tTA, pX458-sgRNA-SVBP-Cas9-T2A-GFP, pX458-sgRNA-MATCAP-Cas9-T2A-GFP	P14	6	109	4-48 neurons/animal	nested t-test (mixed model)
Figure EV5E	WT CD1	comparison of data from Fig. 5A & Suppl. Fig. 5A	P14				L4 p=0.229, nested t-test (mixed model)
Appendix Figure S6A	WT CD1		P4	3		5-6 slices/animal	paired t test, p=0.025
Appendix Figure S6B, C	WT CD1		P4	3		7 - 16 slices/condition analyzed	one-way ANOVA, p<0.0001
Appendix Figure S7A	WT CD1	control: pCAG-CreERT2, pTRE-Flex-FlpO, pCAG-FSF-mCherry-tTA, 2x pX458-sgRNA-control2x-Cas9-T2A-GFP	P7	2		10-12 neurons/condition	t test for each condition:
		experimental: pCAG-CreERT2, pTRE-Flex-FlpO, pCAG-FSF-mCherry-tTA, 2x pX458-sgRNA-MAP1B2x-Cas9-T2A-GFP	P7	2		10-12 neurons/condition	ctrl vs Map1bKO - K40 tubulin p=0.323, PolyE tubulin p=0.1445, a-tubulin p=0.966, PolyE tubulin p=0.292
		control: pCAG-CreERT2, pEF1-Flex-FlpO, pCAG-FSF-mCherry, pCAG-FSF-GFP	P4	1		5 neurons	ctrl vs GSK3B-CA - a-tubulin p=0.436, PolyE tubulin p=0.29
		experimental: pCAG-CreERT2, pEF1-Flex-FlpO, pCAG-FSF-mCherry, pCAG-FSF-GSK3BCA-IRES-GFP	P4	2		14 neurons	
Appendix Figure S9A	WT CD1	control: pCAG-CreERT2, pEF1-Flex-FlpO, pCAG-FSF-MAP1B-Flag, pCAG-FSF-TTL-DN-IRES-GFP	P14	4	41	5-21 neurons/animal	L4 p=0.673, L5 p=0.45, L4+L5 p=0.548 nested t-test (mixed model)
	WT CD1	experimental: pCAG-CreERT2, pEF1-Flex-FlpO, pCAG-FSF-MAP1B-Flag, pCAG-FSF-TTL-IRES-GFP	P14	4	37	7-27 neurons/animal	
Appendix Figure S10A	WT CD1	control: pCAG-CreERT2, pTRE-Flex-FlpO, pCAG-FSF-mCherry-dTA, 2x pX458-sgRNA-control2x-Cas9-T2A-GFP	P14	5	15	1-6 neurons/animal	L4 p=0.85, L5 p=0.13, L4+L5 p=0.095
	WT CD1	control: pCAG-CreERT2, pTRE-Flex-FlpO, pCAG-FSF-mCherry-dTA, 2x pX458-sgRNA-Katna1(2x)-Cas9-T2A-GFP	P14	8	76	4-22 neurons/animal	nested t-test (mixed model)
Appendix Figure S10B	WT CD1	negative control: pCAG-CreERT2, pEF1-Flex-FlpO, pCAG-FSF-mCherry, pCAG-FSF-GFP	P14	5	27	1-13 neurons/animal	L4 p=0.01, L5 p=0.12, L4+5 p=0.023
	WT CD1	experimental: pCAG-CreERT2, pEF1-Flex-FlpO, pCAG-FSF-mCherry, pCAG-FSF-Katanin-p60-YPET-IRES-GFP	P14	8	89	6-14 neurons/animal	nested ANOVA (mixed model) with post-hoc Tukey test
	WT CD1	experimental: pCAG-CreERT2, pEF1-Flex-FlpO, pCAG-FSF-mCherry, pCAG-FSF-KataninDN-p60-YPET-IRES-GFP	P14	4	44	7-18 neurons/animal	
	WT CD1	experimental: pCAG-CreERT2, pEF1-Flex-FlpO, pCAG-FSF-mCherry, pCAG-FSF-Katanin-p60-YPET-IRES-GFP, pCAG-FSF-TTL-IRES-GFP	P14	9	69	1-19 neurons/animal	
	WT CD1	positive control: pCAG-CreERT2, pEF1-Flex-FlpO, pCAG-FSF-mCherry, pCAG-FSF-TTL-IRES-GFP	P14	4	52	4-21 neurons/animal	
		TOTAL		311	2512		

POST-HOC TEST DETAILS

For Figure 1E:

Dunnett's multiple comparisons test	Mean Diff.	95.00% CI of diff.	Below threshold?	Summary	Adjusted P Value
control-L4 vs. GSK3BKO-L4	0.2023	-0.4984 to 0.9031	No	ns	0.7111
control-L4 vs. doubleKO-L4	-0.09443	-0.9068 to 0.7180	No	ns	0.9434
control-L5 vs. GSK3BKO-L5	0.4671	-1.074 to 2.008	No	ns	0.6778
control-L5 vs. doubleKO-L5	1.717	0.07202 to 3.361	Yes	*	**0.0405**
control-L4+L5 vs. GSK3BKO-L4+L5	0.4956	-1.404 to 2.396	No	ns	0.7443
control-L4+L5 vs. doubleKO-L4+L5	1.378	-0.6691 to 3.425	No	ns	0.2071

For Figure 3B:

Dunnett's multiple comparisons test	Mean Diff.	95.00% CI of diff.	Below threshold?	Summary	Adjusted P Value
L4-MAP1B vs. L4-MAP1B-dP	-0.2542	-0.8936 to 0.3851	No	ns	0.5353
L4-MAP1B vs. L4-MAP1B-P	-0.5461	-1.008 to -0.08380	Yes	*	**0.0213**
L5-MAP1B vs. L5-MAP1B-dP	-0.348	-1.537 to 0.8410	No	ns	0.7116
L5-MAP1B vs. L5-MAP1B-P	-1.183	-1.937 to -0.4298	Yes	**	**0.0032**
L4+L5-MAP1B vs. L4+L5-MAP1B-dP	-1.114	-2.577 to 0.3491	No	ns	0.1455
L4+L5-MAP1B vs. L4+L5-MAP1B-P	-1.587	-2.525 to -0.6495	Yes	**	**0.0018**

For Figure 4D:

Dunnett's multiple comparisons test	Mean Diff.	95.00% CI of diff.	Below threshold?	Summary	Adjusted P Value
ctrl-4 vs. VASH-L4	0.1125	-0.3031 to 0.5281	No	ns	0.75
ctrl-4 vs. TTL-L4	-0.6232	-1.039 to -0.2076	Yes	**	**0.0021**
ctrl-5 vs. VASH-L5	-0.04149	-0.9702 to 0.8872	No	ns	0.9905
ctrl-5 vs. TTL-L5	-0.5447	-1.435 to 0.3460	No	ns	0.2569
ctrl-L4+L5 vs. VASH-L4+L5	0.262	-0.7828 to 1.307	No	ns	0.7561
ctrl-L4+L5 vs. TTL-L4+L5	-1.03	-2.037 to -0.02347	Yes	*	**0.0449**

For Figure 5B:

Dunnett's multiple comparisons test	Mean Diff.	95.00% CI of diff.	Below threshold?	Summary	Adjusted P Value
control-L4 vs. TTL-sgRNA-L4	-0.249	-0.9866 to 0.4886	No	ns	0.5854
control-L4 vs. SVBP-sgRNA-L4	-0.7806	-1.555 to -0.006097	Yes	*	**0.0482**
control-L5 vs. TTL-sgRNA-L5	-0.2041	-1.356 to 0.9473	No	ns	0.8629
control-L5 vs. SVBP-sgRNA-L5	-0.06061	-1.271 to 1.149	No	ns	0.9877
control-L4+5 vs. TTL-sgRNA-L4+5	-0.4264	-1.805 to 0.9525	No	ns	0.6585
control-L4+5 vs. SVBP-sgRNA-L4+5	-0.5333	-1.977 to 0.9104	No	ns	0.5663

For Appendix Figure S6C

Dunnett's multiple comparisons test	Mean Diff.	95.00% CI of diff.	Below threshold?	Summary	Adjusted P Value
Total tubulin vs. Tyr-T	-0.7433	-1.222 to -0.2641	Yes	***	**0.001**
Total tubulin vs. detyr-T	-0.2305	-0.7097 to 0.2486	No	ns	0.5668
Total tubulin vs. PolyE-T	0.06405	-0.4151 to 0.5432	No	ns	0.9916
Total tubulin vs. Acet-T	0.1401	-0.3927 to 0.6730	No	ns	0.91

For Appendix Figure S10

Tukey's multiple comparisons test	Mean Diff.	95.00% CI of diff.	Below threshold?	Summary	Adjusted P Value
control-L4 vs. Katanin GOF-L4	-0.1062	-0.9304 to 0.7181	No	ns	0.9954
control-L4 vs. Katanin_mut GOF-L4	0.08329	-0.8566 to 1.023	No	ns	0.9989
control-L4 vs. Katanin+TTL GOF-L4	-0.3884	-1.233 to 0.4566	No	ns	0.6638
control-L4 vs. TTL-GOF-L4	-0.9812	-1.917 to -0.04522	Yes	*	**0.0366**
Katanin GOF-L4 vs. Katanin_mut GO	0.1895	-0.5839 to 0.9628	No	ns	0.95
Katanin GOF-L4 vs. Katanin+TTL GC	-0.2822	-0.9371 to 0.3726	No	ns	0.7138
Katanin GOF-L4 vs. TTL-GOF-L4	-0.875	-1.644 to -0.1064	Yes	*	**0.02**
Katanin_mut GOF-L4 vs. Katanin+TT	-0.4717	-1.267 to 0.3238	No	ns	0.4283
Katanin_mut GOF-L4 vs. TTL-GOF-L4	-1.065	-1.956 to -0.1730	Yes	*	**0.0137**
Katanin+TTL GOF-L4 vs. TTL-GOF-L4	-0.5928	-1.384 to 0.1981	No	ns	0.212

Tukey's multiple comparisons test	Mean Diff.	95.00% CI of diff.	Below threshold?	Summary	Adjusted P Value
control-L5 vs. Katanin GOF-L5	-0.0149	-1.050 to 1.021	No	ns	>0.9999
control-L5 vs. Katanin_mut GOF-L5	0.3383	-0.8322 to 1.509	No	ns	0.9125
control-L5 vs. Katanin+TTL GOF-L5	-0.4805	-1.561 to 0.5996	No	ns	0.6897
control-L5 vs. TTL-GOF-L5	-0.4182	-1.615 to 0.7790	No	ns	0.841
Katanin GOF-L5 vs. Katanin_mut GO	0.3532	-0.5452 to 1.252	No	ns	0.7763
Katanin GOF-L5 vs. Katanin+TTL GC	-0.4656	-1.243 to 0.3115	No	ns	0.418
Katanin GOF-L5 vs. TTL-GOF-L5	-0.4033	-1.336 to 0.5297	No	ns	0.7115
Katanin_mut GOF-L5 vs. Katanin+TT	-0.8188	-1.768 to 0.1307	No	ns	0.1152
Katanin_mut GOF-L5 vs. TTL-GOF-L5	-0.7565	-1.837 to 0.3243	No	ns	0.2702
Katanin+TTL GOF-L5 vs. TTL-GOF-L5	0.06231	-0.9201 to 1.045	No	ns	0.9997

Tukey's multiple comparisons test	Mean Diff.	95.00% CI of diff.	Below threshold?	Summary	Adjusted P Value
control-L4+5 vs. Katanin GOF-L4+5	-0.0115	-1.484 to 1.461	No	ns	>0.9999
control-L4+5 vs. Katanin_mut GOF-L4	0.5733	-1.091 to 2.237	No	ns	0.8475
control-L4+5 vs. Katanin+TTL GOF-L4	-0.6235	-2.161 to 0.9139	No	ns	0.7563
control-L4+5 vs. TTL-GOF-L4+5	-1.13	-2.831 to 0.5707	No	ns	0.3179
Katanin GOF-L4+5 vs. Katanin_mut (	0.5848	-0.6893 to 1.859	No	ns	0.6649
Katanin GOF-L4+5 vs. Katanin+TTL (	-0.612	-1.715 to 0.4915	No	ns	0.4938
Katanin GOF-L4+5 vs. TTL-GOF-L4+5	-1.119	-2.440 to 0.2031	No	ns	0.1262
Katanin_mut GOF-L4+5 vs. Katanin+	-1.197	-2.545 to 0.1513	No	ns	0.0994
Katanin_mut GOF-L4+5 vs. TTL-GOF	-1.703	-3.235 to -0.1716	Yes	*	**0.024**
Katanin+TTL GOF-L4+5 vs. TTL-GOF	-0.5066	-1.900 to 0.8866	No	ns	0.8209
