# Supplementary material for: Microtubule-binding protein MAP1B regulates interstitial axon branching of cortical neurons via the tubulin tyrosination cycle
Source: EMBO J. 2024 Feb 22;43(7):5. doi: 10.1038/s44318-024-00050-3 (PMC10987652; doi:10.1038/s44318-024-00050-3)
Supplement: Supplementary file 3 — Table EV2 [file 44318_2024_50_MOESM3_ESM.pdf]

Table EV2.
Oligonucleotide sequences related to all experiments in this study.

REAGENT or RESOURCE	SOURCE	IDENTIFIER
chicken anti-GFP, 1:1000	AVES	GFP-1010
Living Colors DsRed Polyclonal antibody, 1:1000	Clontech	632496
rabbit anti-RFP, 1:1000	Thermo Fisher	R10367
rat anti-Tubulin-tyrosinated (YL1/2), 1:500	Millipore	MAB1864
mouse anti-Flag, 1:200	Sigma	F1804
rabbit anti-Flag, 1:200	Cell signaling	D6W5B
Anti-phospho MAP1B (Thr1265) Antibody, 1:250	Sigma	ABN58
Anti-α-Tubulin Antibody, clone DM1A, 1:500	Millipore	05-829
Anti-Acetylated Tubulin antibody, Mouse monoclonal, 1:500	Sigma	T7451-25UL
Phospho-GSK-3β (Ser9) (5B3) Rabbit mAb, 1:150	Cell Signaling Technology	9323S
Anti-Detyrosinated alpha-Tubulin Rabbit Monoclonal Antibody, Clone R	Revmab Biosciences	31-1335-00
anti-Polyglutamate chain (polyE), pAb (IN105), 1:500	Adipogen Lifesciences	AG-25B-0030-C050
anti-GSK3B-nonphospho, 1:250	Nicholas M. Kanaan	ref: Grabinski&Kanaan, 2016
anti-MAP1B (AA6), 1:50	Santa Cruz	sc-58784
anti-Hemaglutinin antibody (Rb), C29F4, 1:500	Cell signaling	C29F4
Goat anti-Chicken IgY (H+L), Alexa Fluor 488, 1:1000	Fisher	A11039
Goat anti-Rabbit IgG (H+L) Highly Cross-Adsorbed, Alexa Fluor 555, 1:	Fisher	A21429
Alexa Fluor 647 goat anti-mouse IgG (H+L), 1:1000	Fisher	A21236
Alexa Fluor 647 goat anti-rat IgG (H+L), 1:1000	Fisher	A21247
DH5α competent bacteria	N/A	N/A
OmniPur® PIPES, Sodium Salt	Millipore Sigma	6910-OP
Tamoxifen	Sigma	T5648-1G
Corn oil	Sigma	C8267
DPBS 1x, no calcium, no magnesium	Fisher	14190250
Bupivicaine hydrochloride	Sigma	B5274-5G
Buprenorphine ER-LAB 5ml (1mg/ml)	ZooPharm, LLC	N/A
DAPI (4',6-Diamidino-2-Phenylindole, Dihydrochloride)	Fisher	D1306
FluoroGel with DABCO	Electron microscopy sciences	17985-01
REDExtract-N-Amp(TM)	Sigma	R4775-125ML
QuickExtract DNA extraction solution	MRC	DN 131- 25 ml
T4 DNA ligase	NEB	M0202S
T4 DNA ligase buffer	NEB	B0202S
ATP solution	Sigma	A2383-1G
Dinucleotide phosphates	Fisher	10297018
NEBuilder® HiFi DNA Assembly Cloning Kit	NEB	E5520S
NucleoBond® Xtra Midi EF	Clontech	740420.5
**PLASMIDS**	**SOURCE**	**IDENTIFIER**
pCAG-CreERT2	Dorskind et al.	
pEF1-Flex-FlpO	Dorskind et al.	
pCAG-FSF-GFP	Dorskind et al.	
pCAG-FSF-mCherry	Dorskind et al.	
pAAV-Tre-Flex-FlpO	Lin et al. 2018	Addgene no. 118027
CAG-FSF-RFP-ires-tTA-WPRE	Luo et al. 2016	Addgene no. 85038
pCAG-GFP	Dorskind et al.	
pCAG-FSF-GSK3BCA-IRES-GFP human	Dorskind et al.	derived from Addgene no. 14754, Stambolic et al. 1994
pCAG-FSF-GSK3BCA-IRES-GFP mouse	this paper	
pCAG-FSF-GSK3BDN-IRES-GFP mouse	this paper	
pPrime-dsRed-miR30-shRNA-MAP1B	this paper	
pPrime-dsRed-miR30-shRNA-CLASP1	this paper	
pPrime-dsRed-miR30-shRNA-CLASP2	this paper	
pPrime-dsRed-miR30-shRNA-MACF1	this paper	
pPrime-dsRed-miR30-shRNA-scrambled	this paper	
pX458-sgRNA-control2x-Cas9-T2A-GFP-A	this paper	derived from Addgene no. 48138, Run et al. 2013
pX458-sgRNA-control2x-Cas9-T2A-GFP-B	this paper	
pX458-sgRNA-control2x-Cas9-T2A-GFP-C	this paper	
pX458-sgRNA-control2x-Cas9-T2A-GFP-D	this paper	
pX458-sgRNA-MAP1B2x-Cas9-T2A-GFP-A	this paper	
pX458-sgRNA-MAP1B2x-Cas9-T2A-GFP-B	this paper	
pCAG-FSF-MAP1B-Flag	this paper	
pCAG-FSF-MAP1B-P-Flag	this paper	
pCAG-FSF-MAP1B-deltaP-Flag	this paper	
pCAG-EB3-GFP	this paper	derived from Addgene no. 190164, Stepanova et al. 2003
pMini-donor-Actin	this paper	
pX330-sgRNA-Actin-Cas9	this paper	derived from Addgene no. 42230, Cong et al. 2013
pMini-donor-MAP1B	this paper	derived from pMiniT Vector (NEB, E1202)
pX330-sgRNA-MAP1B-Cas9	this paper	
pMini-donor-GSK3B	this paper	
pX330-sgRNA-GSK3B-Cas9	this paper	
pMini-TagRFP-T_A1aY1	this paper	derived from Addgene no. 158751, Kesarwani et al. 2020
pX330-2xsgRNA-Rosa26-Cas9	this paper	
pMini-donor-Tuba1b	this paper	
pX330-sgRNA-Tuba1b-HA	this paper	
pCAG-FSF-VASH1-IRES-GFP	this paper	
pCAG-FSF-VASH2-IRES-GFP	this paper	
pCAG-FSF-SVBP-IRES-GFP	this paper	
pCAG-FSF-TTL-IRES-GFP	this paper	
pCAG-FSF-TTLDN-IRES-GFP	this paper	
pX458-sgRNA-TTL2x-Cas9-T2A-GFP-A	this paper	
pX458-sgRNA-TTL2x-Cas9-T2A-GFP-B	this paper	
pX458-sgRNA-SVBP2x-Cas9-T2A-GFP-A	this paper	
pX458-sgRNA-SVBP2x-Cas9-T2A-GFP-B	this paper	
pX458-sgRNA-KATNA1 2x-Cas9-T2A-GFP-B	this paper	
pX458-sgRNA-KATNA1 2x-Cas9-T2A-GFP-B	this paper	
pCAG-FSF-Katanin(p60)-IRES-GFP	this paper	
pCAG-FSF-Katanin(p60)–mutE306Q-IRES-GFP	this paper	
pX458-sgRNA-MATCAP2x-Cas9-T2A-GFP	this paper	

OLIGONUCLEOTIDES	NAME	SEQUENCE	NOTES
genotyping	GSK3A-KO-F	CCC CCA CCA AGT GAT TTC ACT GCT A	Feng-Quan Zhou lab
genotyping	GSK3A-KO-R	AAC ATG AAA TTC CGG GCT CCA ACT CT	Feng-Quan Zhou lab
genotyping	GSK3B-fl-F	ACA GGC CAC AGG AAG TCA GT	Feng-Quan Zhou lab
genotyping	GSK3B-fl-R	TCT GGG CTA TAG CTA TCT AGT AAC	Feng-Quan Zhou lab
genotyping	Bcat-fl-F	AAG GTA GAG TGA TGA AAG TTG TT	Jeremy Nathans lab
genotyping	Bcat-fl-R	CAC CAT GTC CTC TGT CTA TTC	Jeremy Nathans lab
genotyping	APC-fl-F	GTT CTG TAT CAT GGA AAG ATA GGT GGT C	Bart Williams lab
genotyping	APC-fl-R	CAC TCA AAA CGC TTT TGA GGG TTG ATT C	Bart Williams lab
knockdown	MAP1B-shRNA	GCCCAAGAAAGAAGTGGTTAA	Benoist et al. 2013
knockdown	CLASP1-shRNA	GCCATTATGCCAACTATCTTT	Mimori-Kiyosue et al. 2005
knockdown	CLASP2-shRNA	GTTCAGAAAGCCCTTGATATT	Mimori-Kiyosue et al. 2005
knockdown	MACF1-shRNA	GCAGAGATGTATCATCCATCAA	Ka et al. 2014
CRISPR-knockdown	MAP1B-sgRNA-1	GGAGCCATCGGGCAGCAT	Bassik Mouse CRISPR Knockout Library, see ref. Morgens et al. 2017
CRISPR-knockdown	MAP1B-sgRNA-2	GGTTTGTGTCCCACGAT	
CRISPR-knockdown	MAP1B-sgRNA-3	GGAACCCCCAACCTCGGG	
CRISPR-knockdown	MAP1B-sgRNA-4	GTTTCTAAGACGTCACTT	
CRISPR-knockdown	control-sgRNA-1	ggcccccatatatgcca	
CRISPR-knockdown	control-sgRNA-2	aaCCTCAACCATAACCCT	
CRISPR-knockdown	control-sgRNA-3	gccccacccacaataggct	
CRISPR-knockdown	control-sgRNA-4	ATATCCAACTTagccaggcg	
CRISPR-knockin	actin-sgRNA-F	AGAAGGCTATAGTCACCTCG	designed in benchling, On- and Off-target score calculated online according to The Doench, Fusi et al. (2016)
CRISPR-knockin	actin-sgRNA-R	TCGATCCCCAAGAAAACCCC	
CRISPR-knockin	MAP1B-sgRNA-F	ttttttGGGATGTCATTGAA	
CRISPR-knockin	MAP1B-sgRNA-R	gggaaagagagtcagcggga	
CRISPR-knockin	GSK3B-sgRNA-F	CTGTGTGTTTGAACCCACAT	
CRISPR-knockin	GSK3B-sgRNA-R	CACTTTTCAGGAGATCCAAG	
CRISPR-knockin	Rosa26-sgRNA-F	cacaagagtagttacttggc	
CRISPR-knockin	Rosa26-sgRNA-R	ATATCCAACTTagccaggcg	
CRISPR-knockin	Tuba1b-sgRNA-F	AGGTAAGTAACGCAATGAGG	
CRISPR-knockin	Tuba1b-sgRNA-R	CCTGCACGGAACGTGCAGCA	
CRISPR-knockdown	TTL-sgRNA-1	GGGCATCCTCATCTCCTCAG	Bassik Mouse CRISPR Knockout Library
CRISPR-knockdown	TTL-sgRNA-2	GCCTCTTCCAGTAGCCGG	
CRISPR-knockdown	TTL-sgRNA-3	GGATTGCAAAGTCATCAGC	
CRISPR-knockdown	TTL-sgRNA-4	GCTGGTGAATTACTACCGGG	
CRISPR-knockdown	SVBP-sgRNA-1	GTCTCAGTTTCCTGCACAA	
CRISPR-knockdown	SVBP-sgRNA-2	GCATTCAGAAGCCAAACCA	
CRISPR-knockdown	SVBP-sgRNA-3	TAAAGAACCAGCCTTCAGAG	
CRISPR-knockdown	SVBP-sgRNA-4	GCTCTCAACAGAGTCATGA	
CRISPR-knockdown	MATCAP-sgRNA-1	GGTGCTGGACTCGGGGACAC	Bassik Mouse CRISPR Knockout Library, reference gene name: 4931428F04Rik, see ref. Landskron et al. 2022
CRISPR-knockdown	MATCAP-sgRNA-2	GCATCCCCGTCCAATCG	
CRISPR-knockdown	MATCAP-sgRNA-3	GCATGTATTTGCGCACGG	
CRISPR-knockdown	MATCAP-sgRNA-4	GAAGGGACTGCCAGAGCCAA	
pX plasmid construction	insert1 - F	tggccttttgctggccttttgctcacatgtGAGGGCCTATTTCCCATG	TKIT method reference: Fang et al. 2021
pX plasmid construction	insert1 - R	tagctctaaaacNNNNNNNNNNNNNNNCGGTGTTTCGTCCTTTCCAC	
pX plasmid construction	insert2 - F	aggacgaaacaccGNNNNNNNNNNNNNGTTTTAGAGCTAGAAATAGCAAGTTaaa[illegible]	
pX plasmid construction	insert2 - R	tagctctaaaac**NNNNNNNNNNNNNNN**CGGTGTTTCGTCCTTTCCAC	
pX plasmid construction	insert3 - F	aggacgaaacaccG**NNNNNNNNNNNNNNNNNNNNN**GTTTTAGAGCTAGAAATA[illegible]	
pX plasmid construction	insert3 - R	ccatttaccgtaagttatgtaacgggtaccgatatctagaaaaaagcaccg	
