# Supplementary material for: Microtubule-binding protein MAP1B regulates interstitial axon branching of cortical neurons via the tubulin tyrosination cycle
Source: EMBO J. 2024 Feb 22;43(7):5. doi: 10.1038/s44318-024-00050-3 (PMC10987652; doi:10.1038/s44318-024-00050-3)
Supplement: Supplementary file 4 — Movie EV1 [file 44318_2024_50_MOESM4_ESM.zip › Movie EV1/Movie EV1-legend.docx]

**Movie EV1.**

Time-lapse imaging of EB3-GFP particles in DIV4 cultured layer 2/3 CPNs.
