# Supplementary figures and images for: Microtubule-binding protein MAP1B regulates interstitial axon branching of cortical neurons via the tubulin tyrosination cycle (part 1 of 2)
Source: EMBO J. 2024 Feb 22;43(7):5. doi: 10.1038/s44318-024-00050-3 (PMC10987652; doi:10.1038/s44318-024-00050-3)

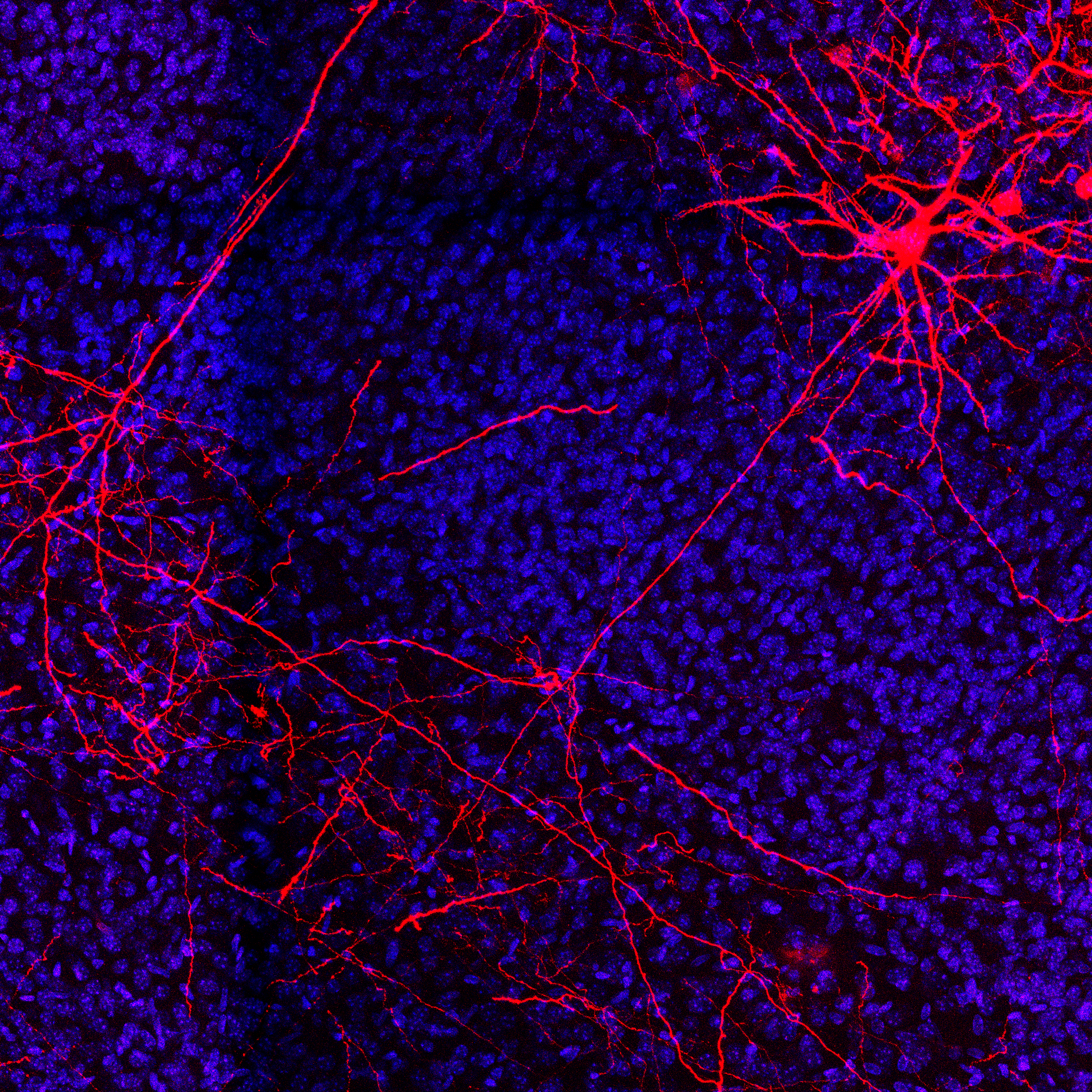

Supplement: Supplementary file 5 — Source Data Fig. 1 [file 44318_2024_50_MOESM5_ESM.zip › Figure1-source files/Figure1B-control.tif]

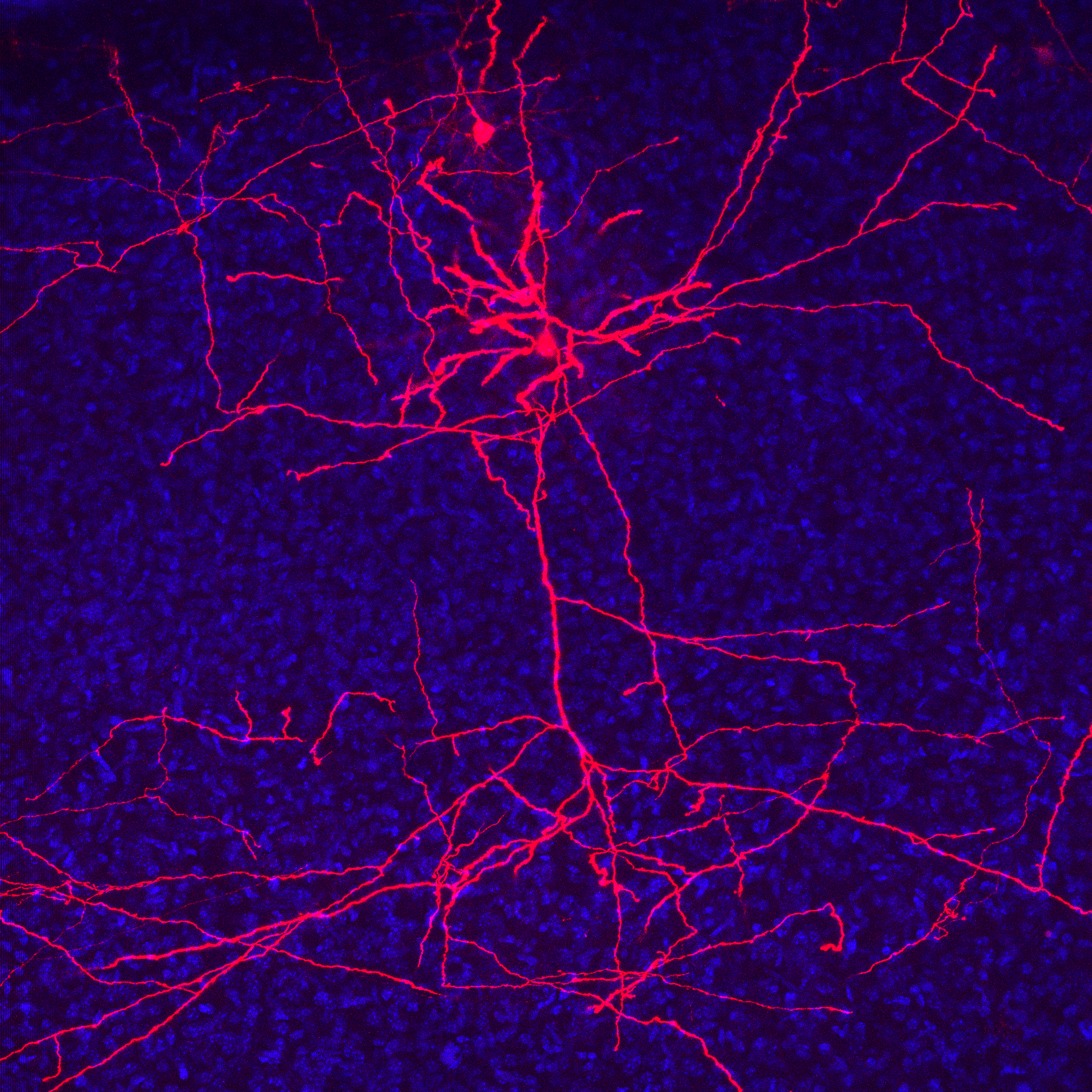

Supplement: Supplementary file 5 — Source Data Fig. 1 [file 44318_2024_50_MOESM5_ESM.zip › Figure1-source files/Figure1D-control.tif]

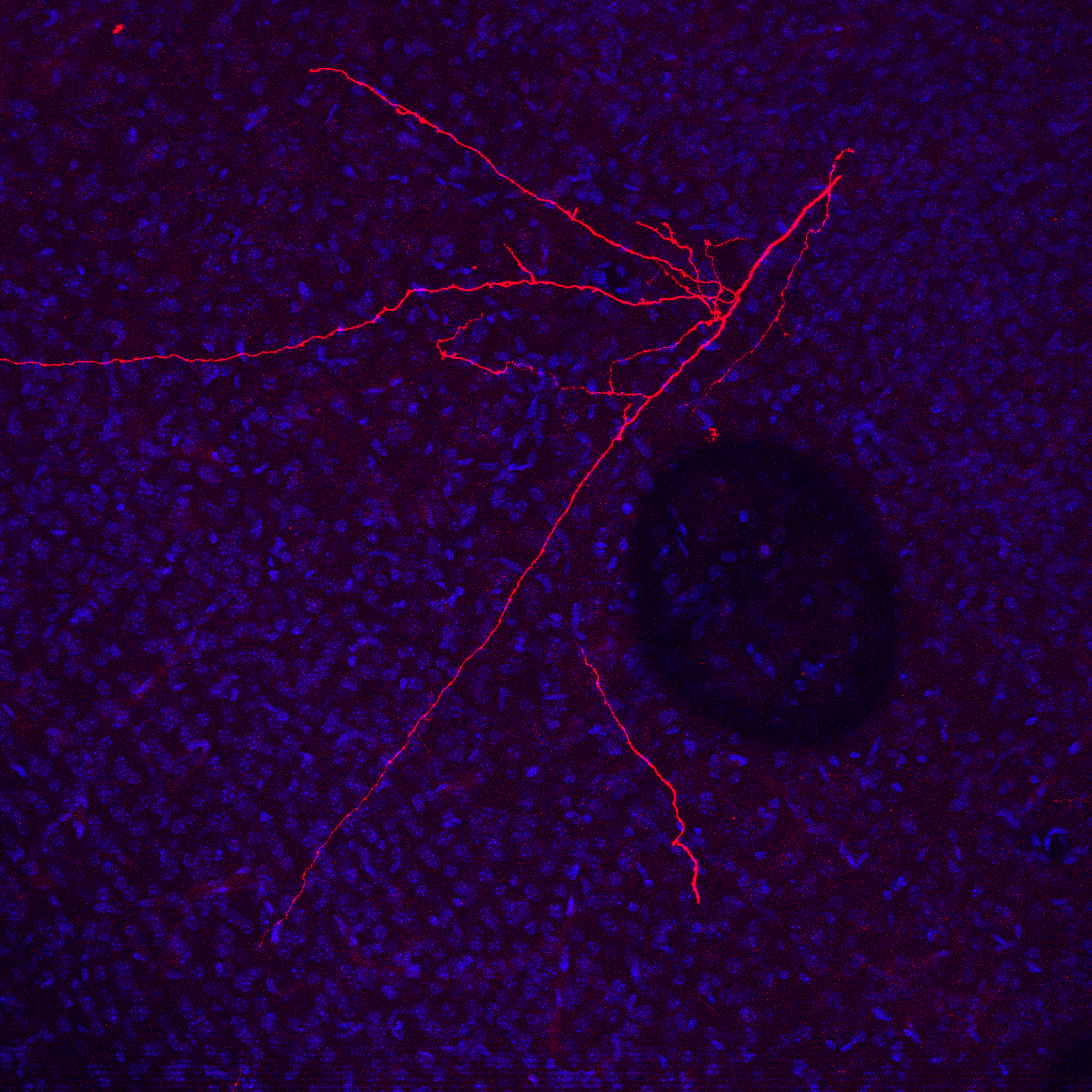

Supplement: Supplementary file 5 — Source Data Fig. 1 [file 44318_2024_50_MOESM5_ESM.zip › Figure1-source files/Figure1D-double KO.tif]

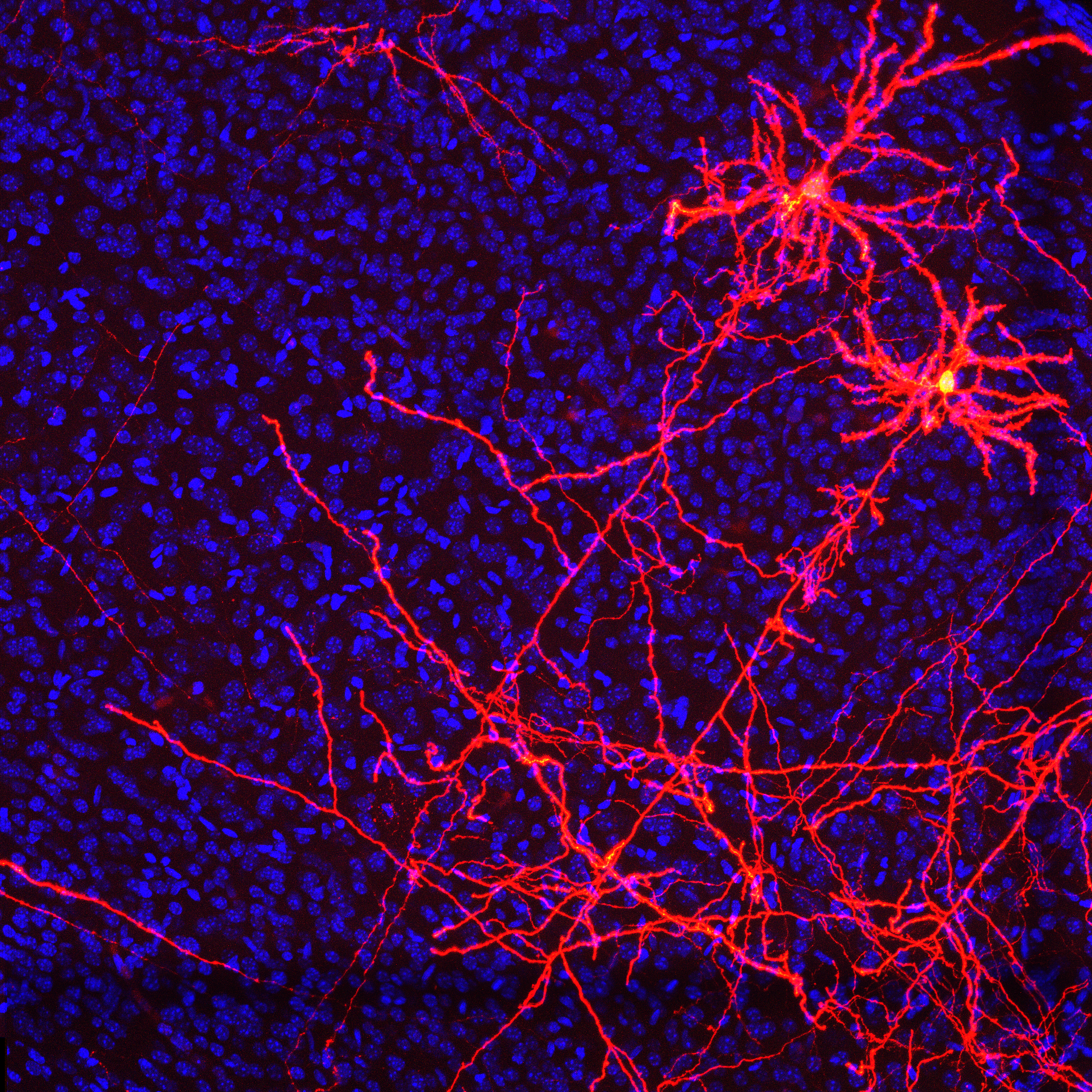

Supplement: Supplementary file 5 — Source Data Fig. 1 [file 44318_2024_50_MOESM5_ESM.zip › Figure1-source files/Figure1B-GSK3B-CA.tif]

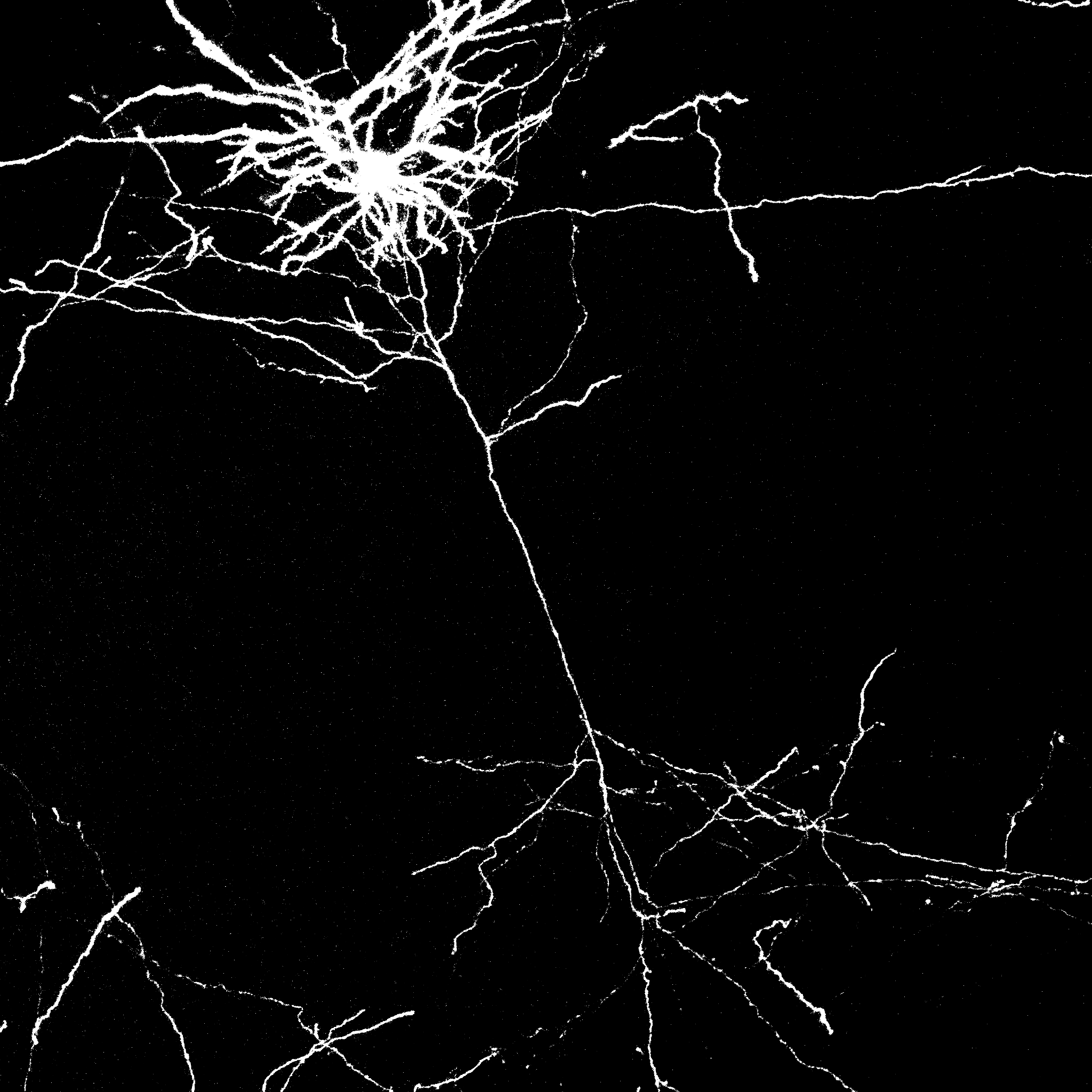

Supplement: Supplementary file 5 — Source Data Fig. 1 [file 44318_2024_50_MOESM5_ESM.zip › Figure1-source files/Figure1A-single channel.tif]

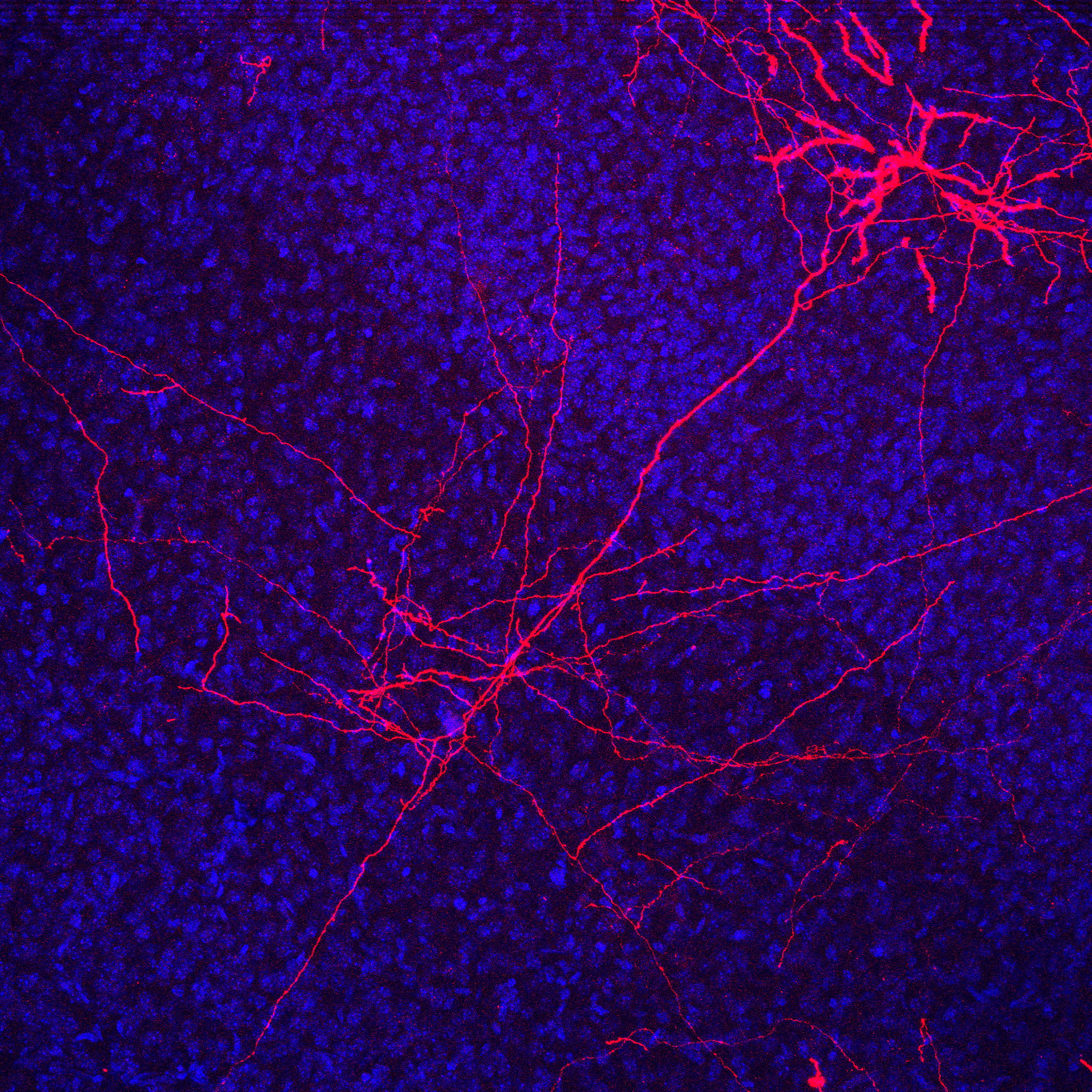

Supplement: Supplementary file 5 — Source Data Fig. 1 [file 44318_2024_50_MOESM5_ESM.zip › Figure1-source files/Figure1D-GSK3B KO.tif]

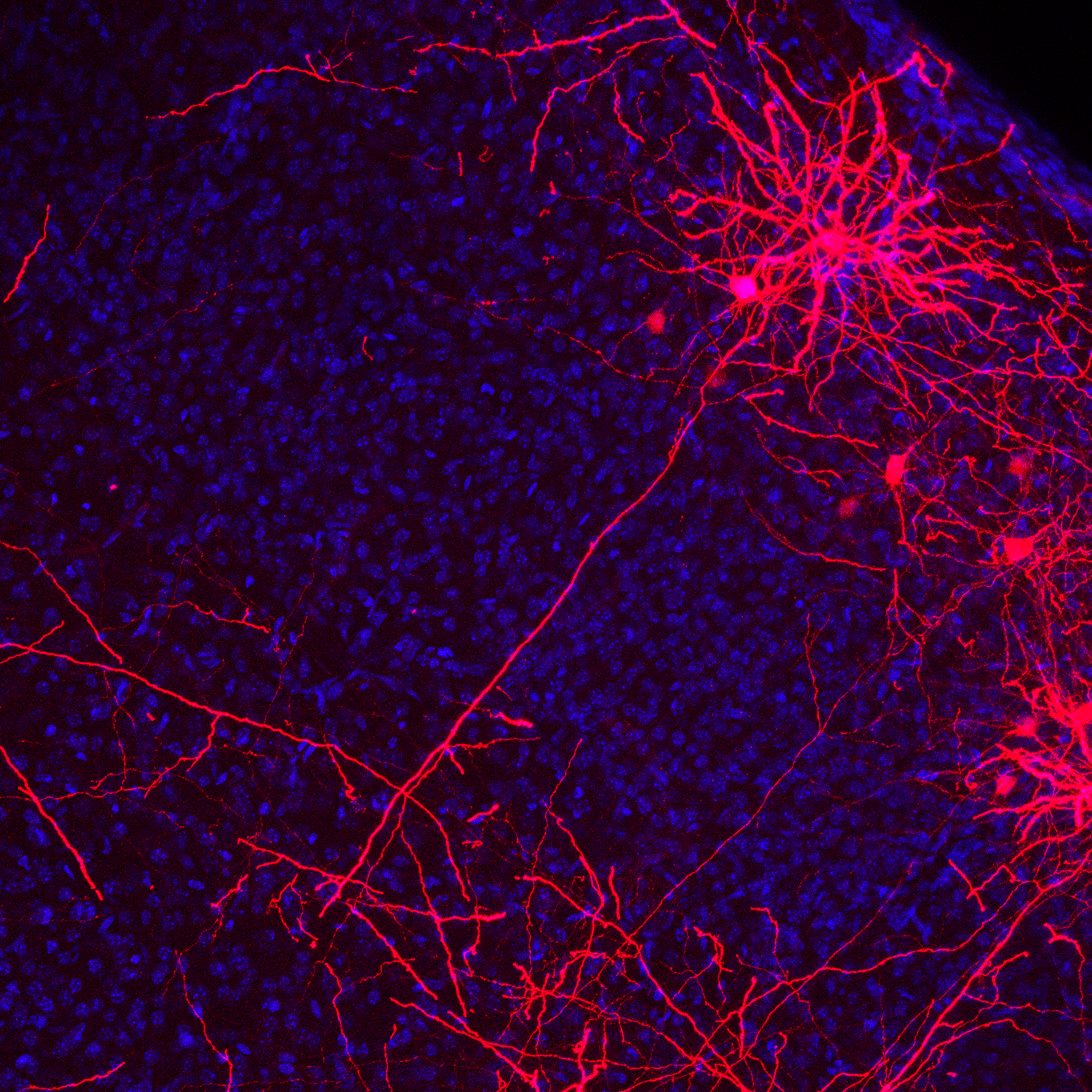

Supplement: Supplementary file 5 — Source Data Fig. 1 [file 44318_2024_50_MOESM5_ESM.zip › Figure1-source files/Figure1C-control.tif]

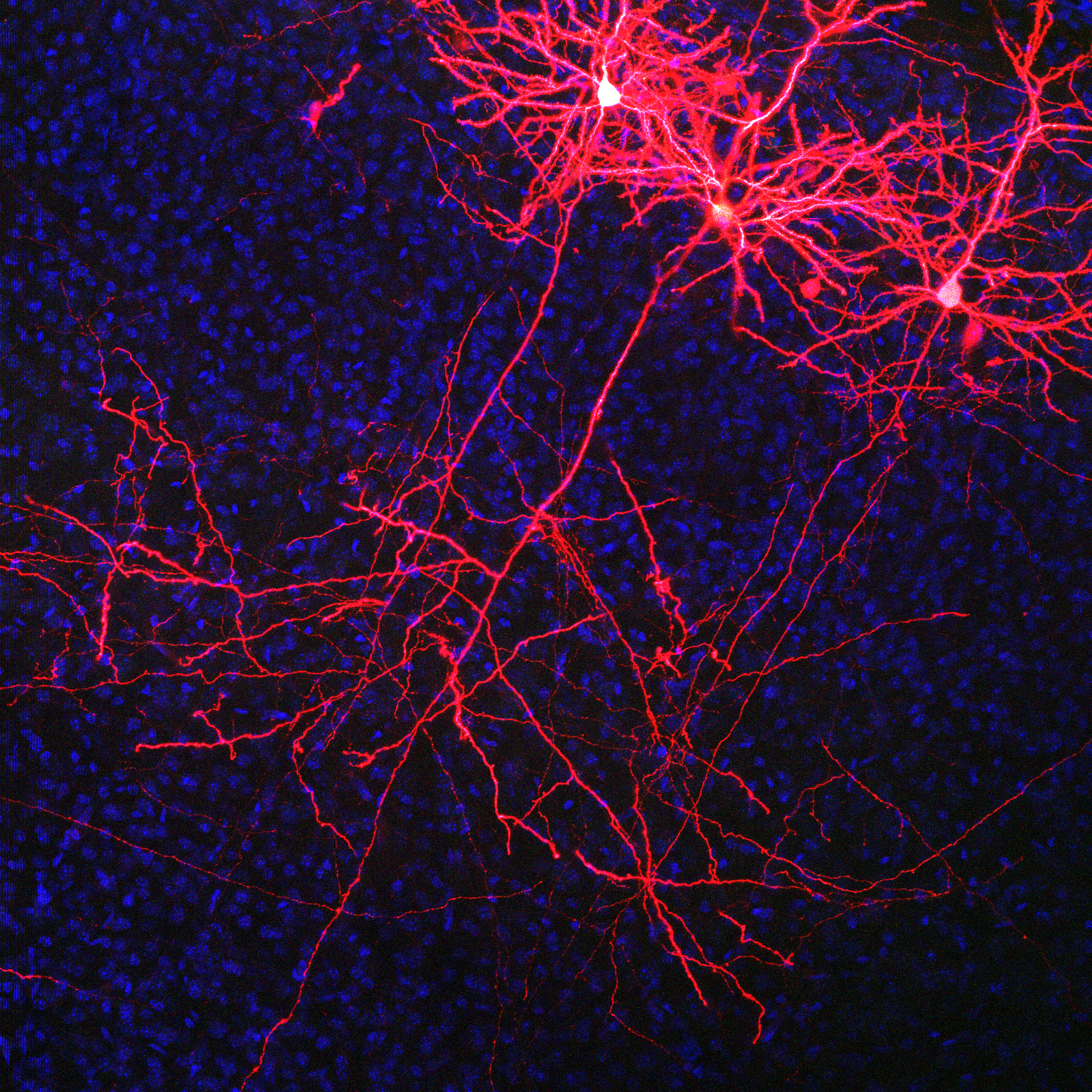

Supplement: Supplementary file 5 — Source Data Fig. 1 [file 44318_2024_50_MOESM5_ESM.zip › Figure1-source files/Figure1C-GSK3B-DN.tif]

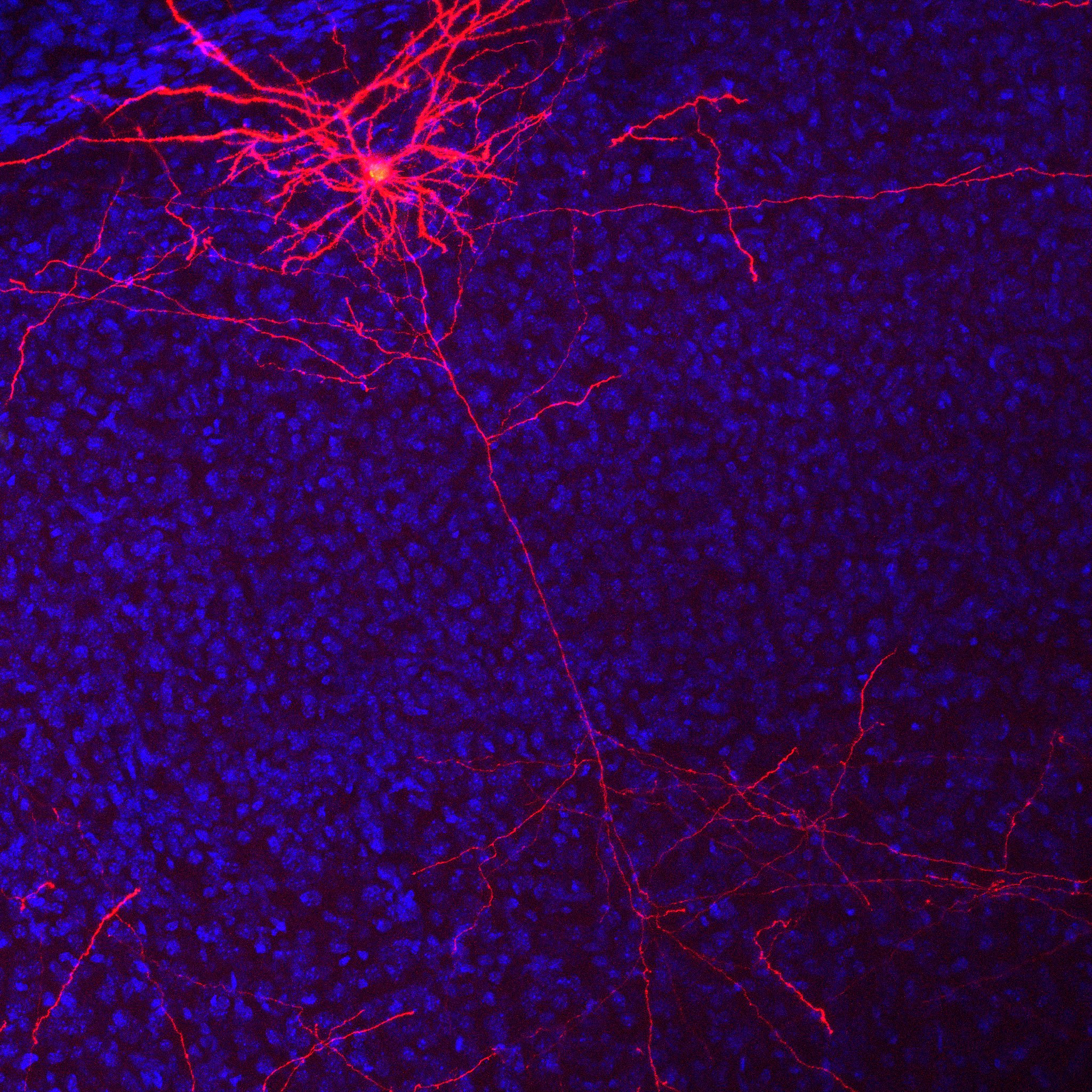

Supplement: Supplementary file 5 — Source Data Fig. 1 [file 44318_2024_50_MOESM5_ESM.zip › Figure1-source files/Figure1A.jpg]

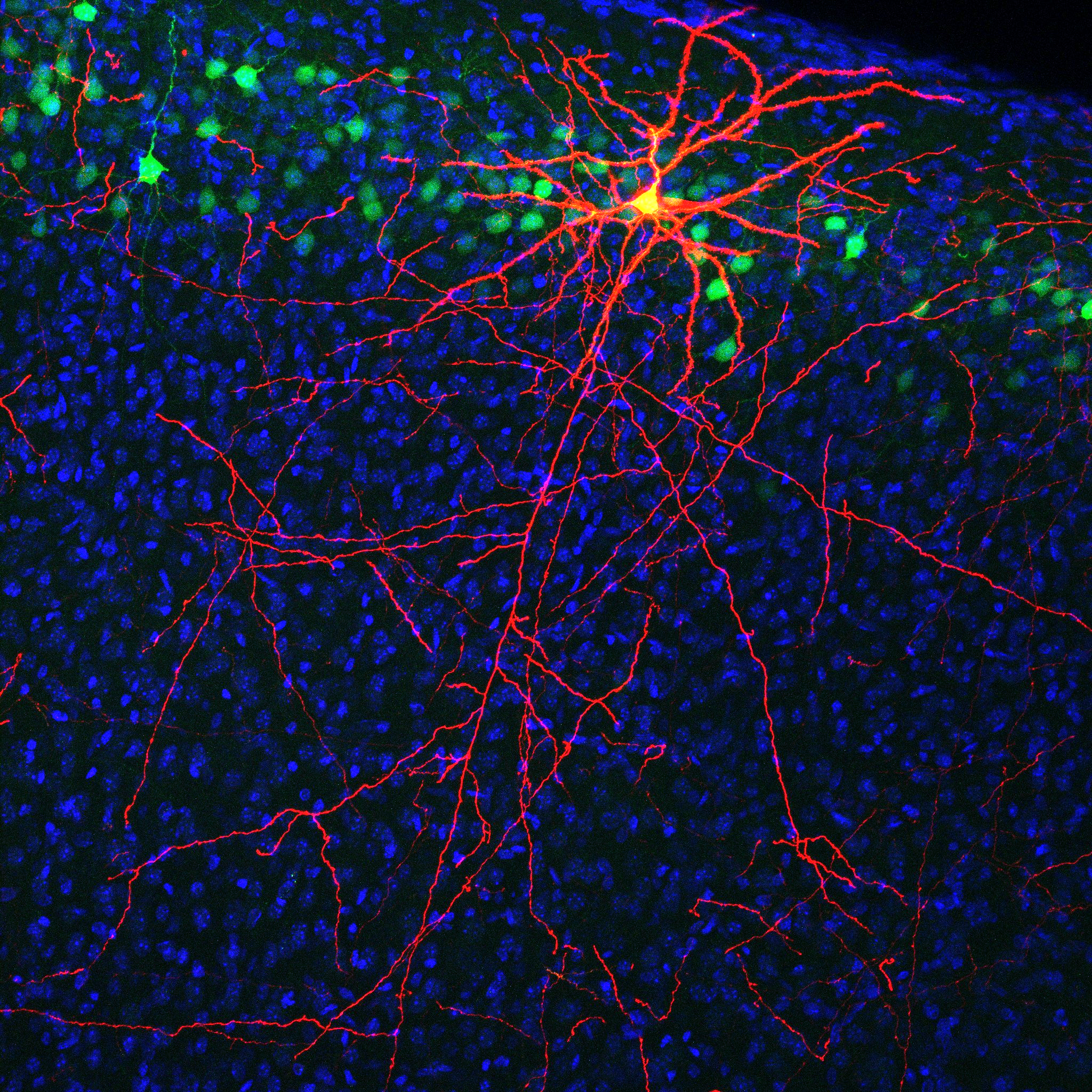

Supplement: Supplementary file 6 — Source Data Fig. 2 [file 44318_2024_50_MOESM6_ESM.zip › Figure2-source files/Figure2C-MAP1BsgRNA.tif]

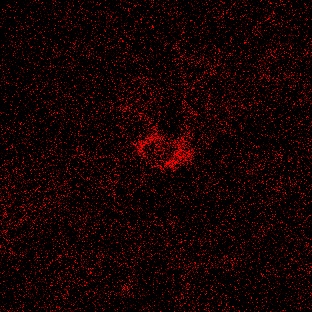

Supplement: Supplementary file 6 — Source Data Fig. 2 [file 44318_2024_50_MOESM6_ESM.zip › Figure2-source files/Figure2E-C2-MAX.tif]

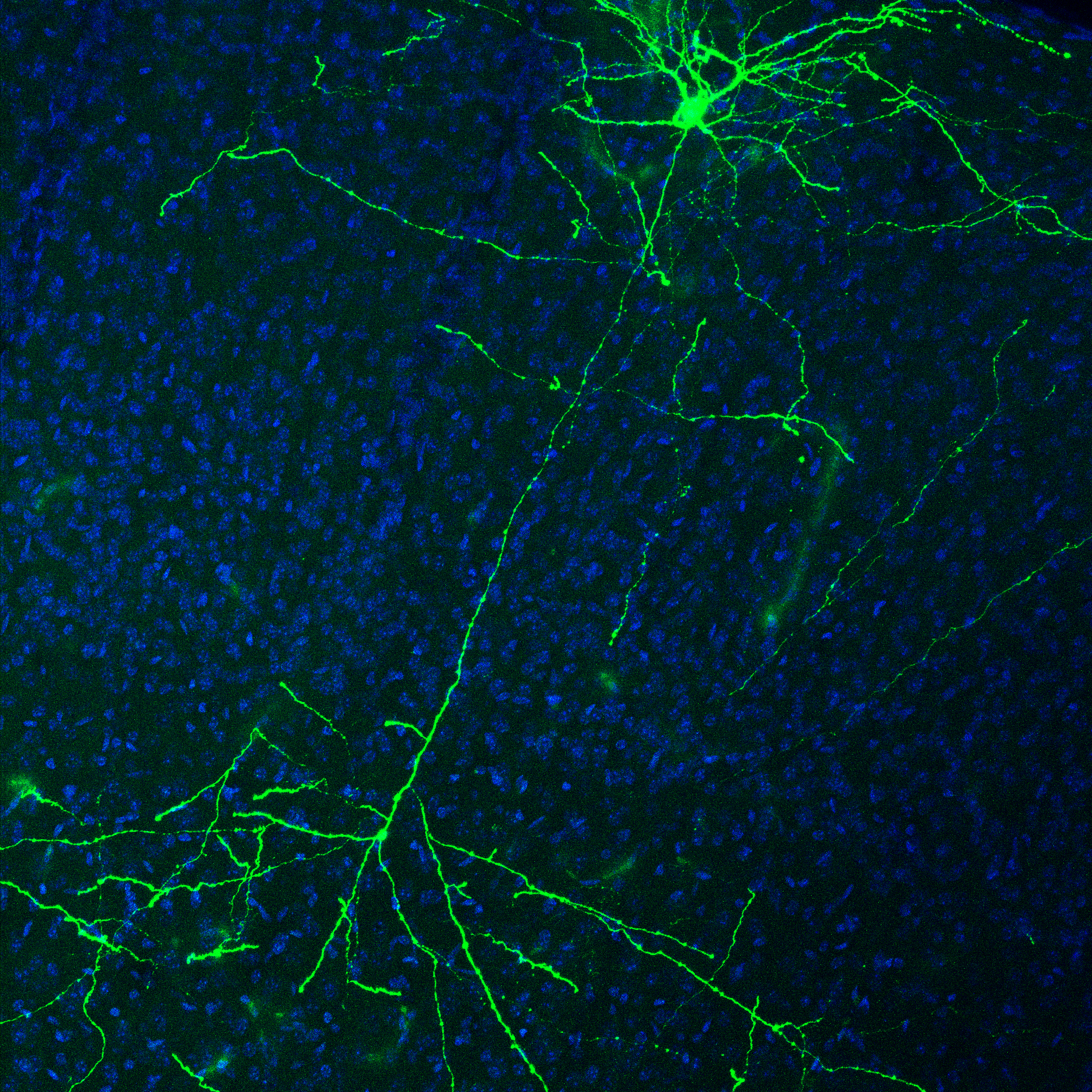

Supplement: Supplementary file 6 — Source Data Fig. 2 [file 44318_2024_50_MOESM6_ESM.zip › Figure2-source files/Figure2E-control.tif]

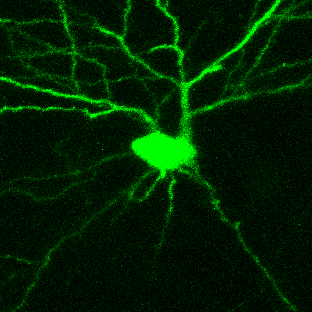

Supplement: Supplementary file 6 — Source Data Fig. 2 [file 44318_2024_50_MOESM6_ESM.zip › Figure2-source files/Figure2E-C1-MAX.tif]

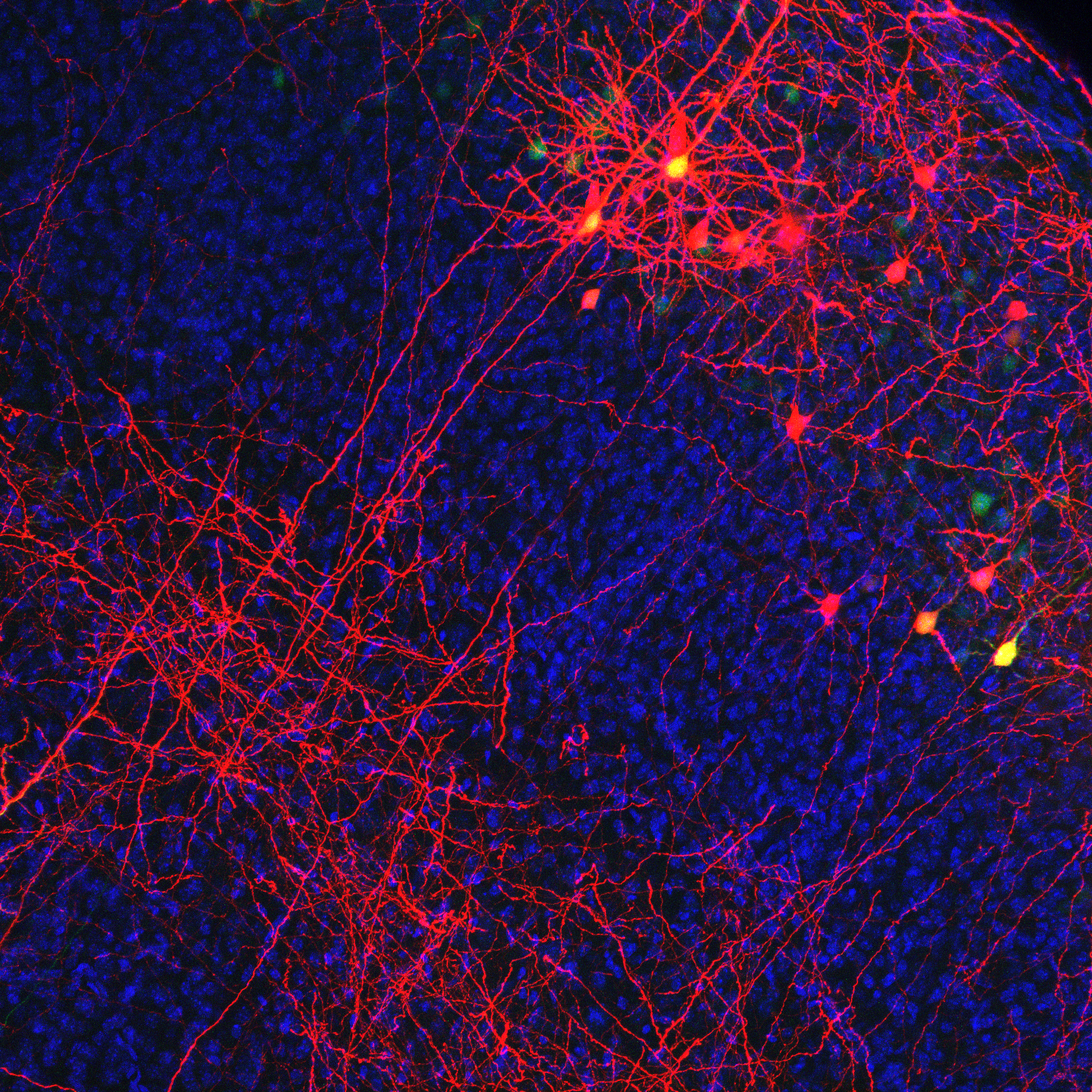

Supplement: Supplementary file 6 — Source Data Fig. 2 [file 44318_2024_50_MOESM6_ESM.zip › Figure2-source files/Figure2C-control.tif]

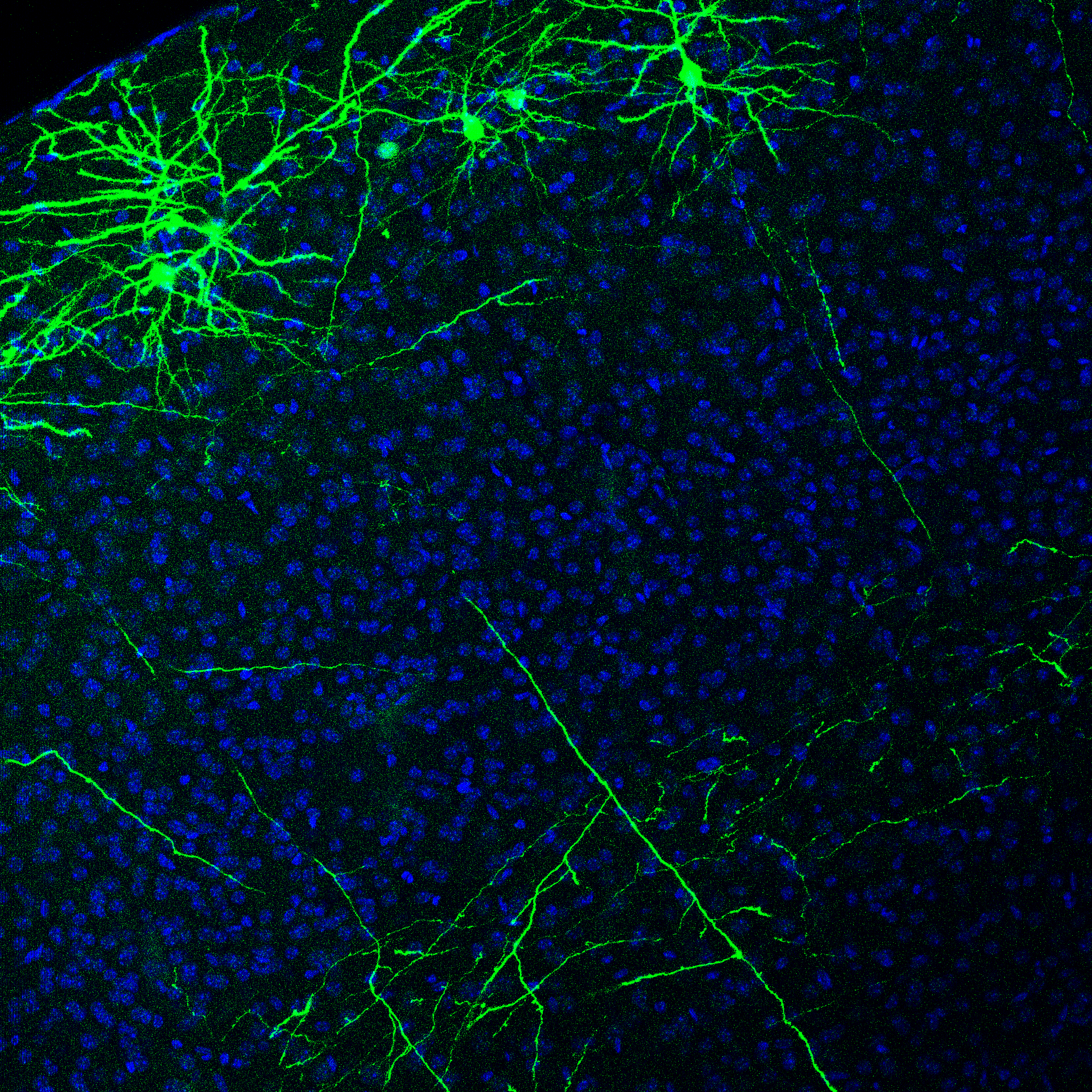

Supplement: Supplementary file 6 — Source Data Fig. 2 [file 44318_2024_50_MOESM6_ESM.zip › Figure2-source files/Figure2E-MAP1B-Flag.tif]

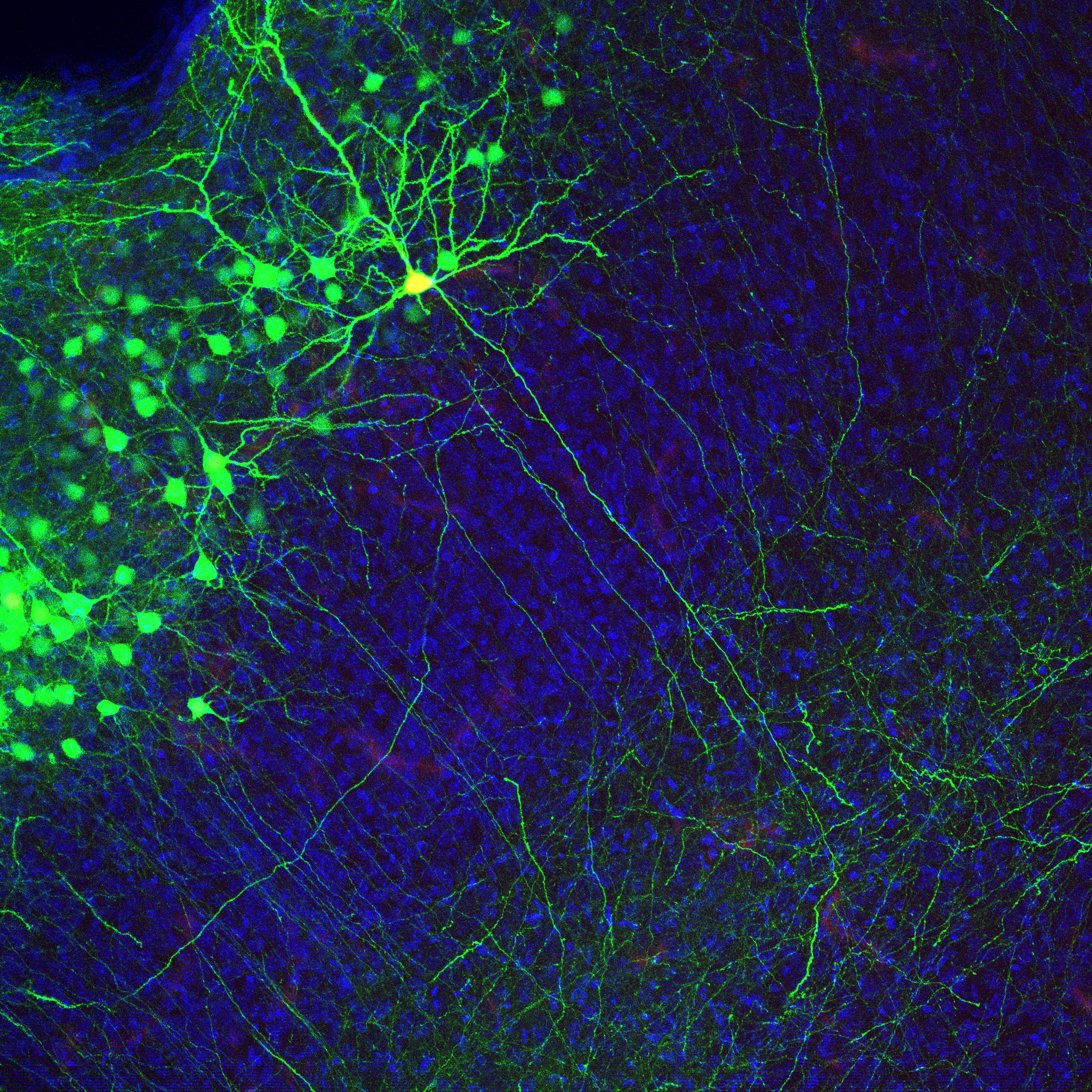

Supplement: Supplementary file 6 — Source Data Fig. 2 [file 44318_2024_50_MOESM6_ESM.zip › Figure2-source files/Figure2A-control.tif]

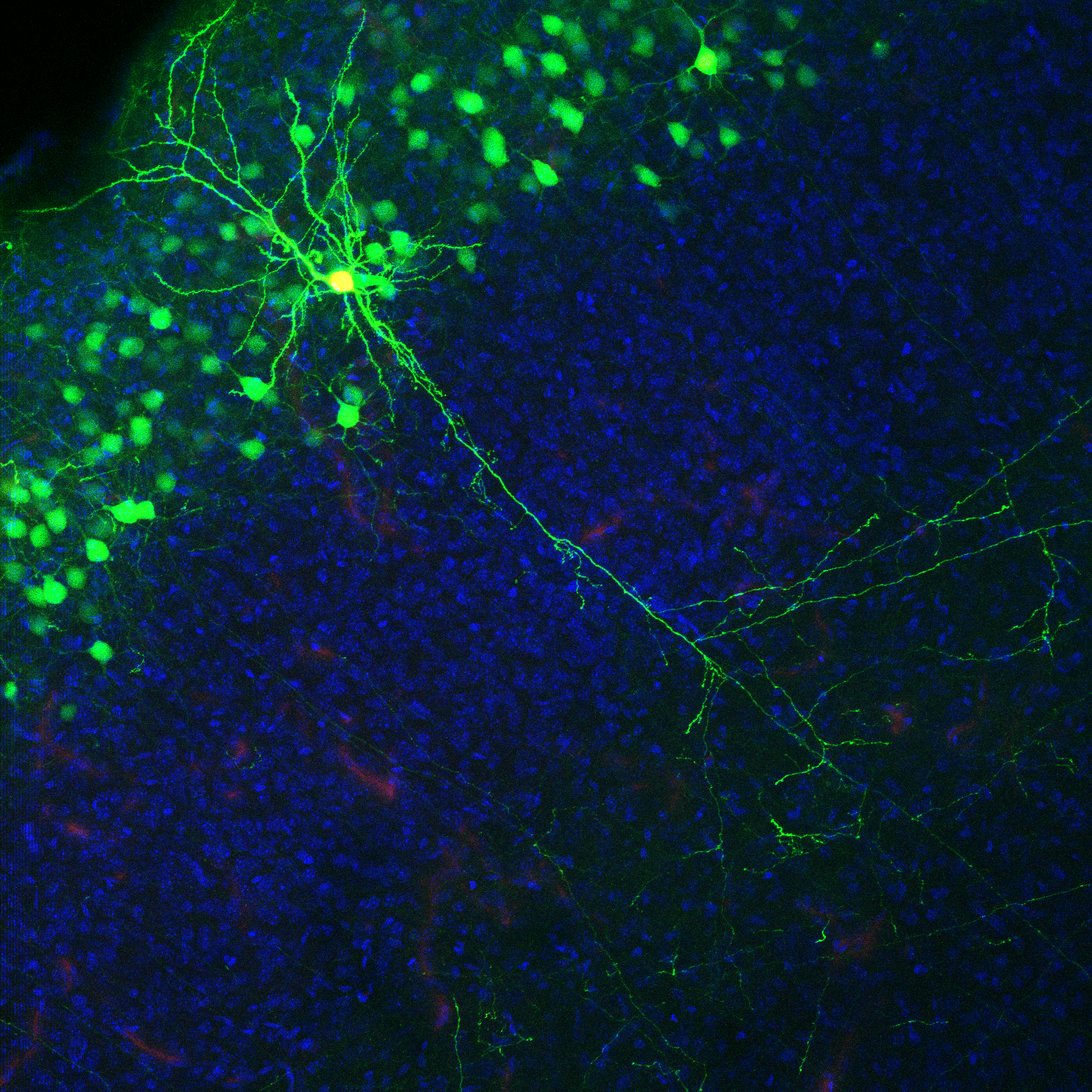

Supplement: Supplementary file 6 — Source Data Fig. 2 [file 44318_2024_50_MOESM6_ESM.zip › Figure2-source files/Figure2A-MAP1BshRNA.tif]

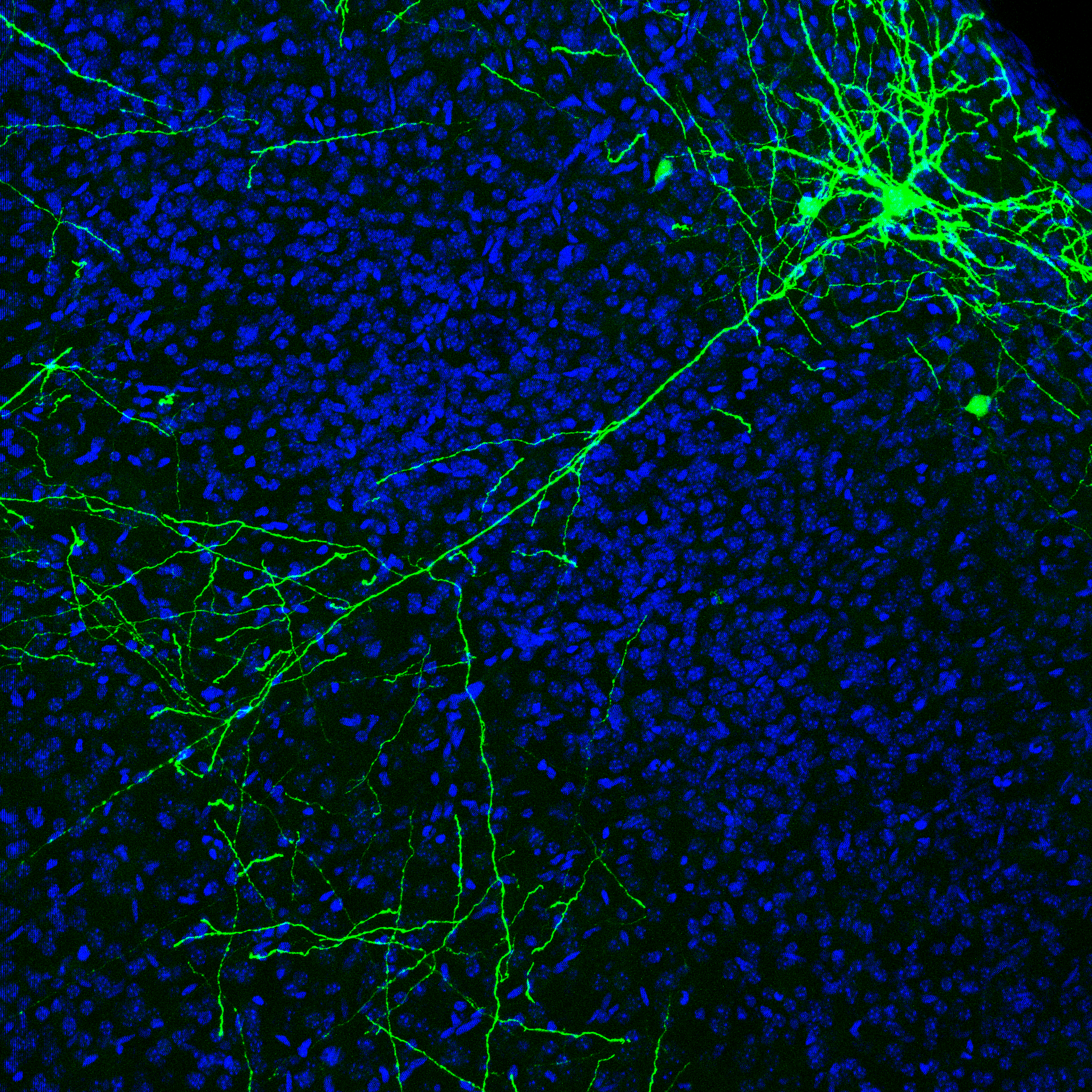

Supplement: Supplementary file 7 — Source Data Fig. 3 [file 44318_2024_50_MOESM7_ESM.zip › Figure3-source files/Figure3A-MAX_MAP1B-P.tif]

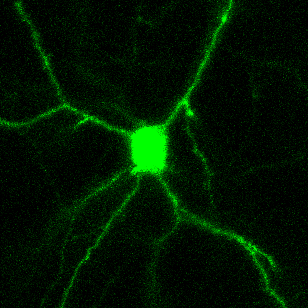

Supplement: Supplementary file 7 — Source Data Fig. 3 [file 44318_2024_50_MOESM7_ESM.zip › Figure3-source files/C1-MAX_MAP1B-dP-GFP.tif]

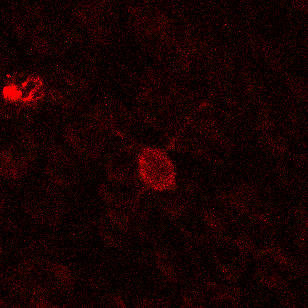

Supplement: Supplementary file 7 — Source Data Fig. 3 [file 44318_2024_50_MOESM7_ESM.zip › Figure3-source files/C2-MAX_MAP1B-P-Flag.tif]

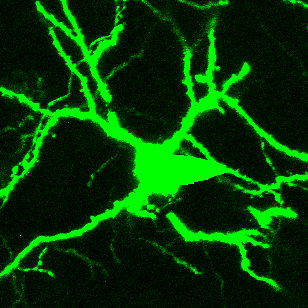

Supplement: Supplementary file 7 — Source Data Fig. 3 [file 44318_2024_50_MOESM7_ESM.zip › Figure3-source files/C1-MAX_MAP1B-P-GFP.tif]

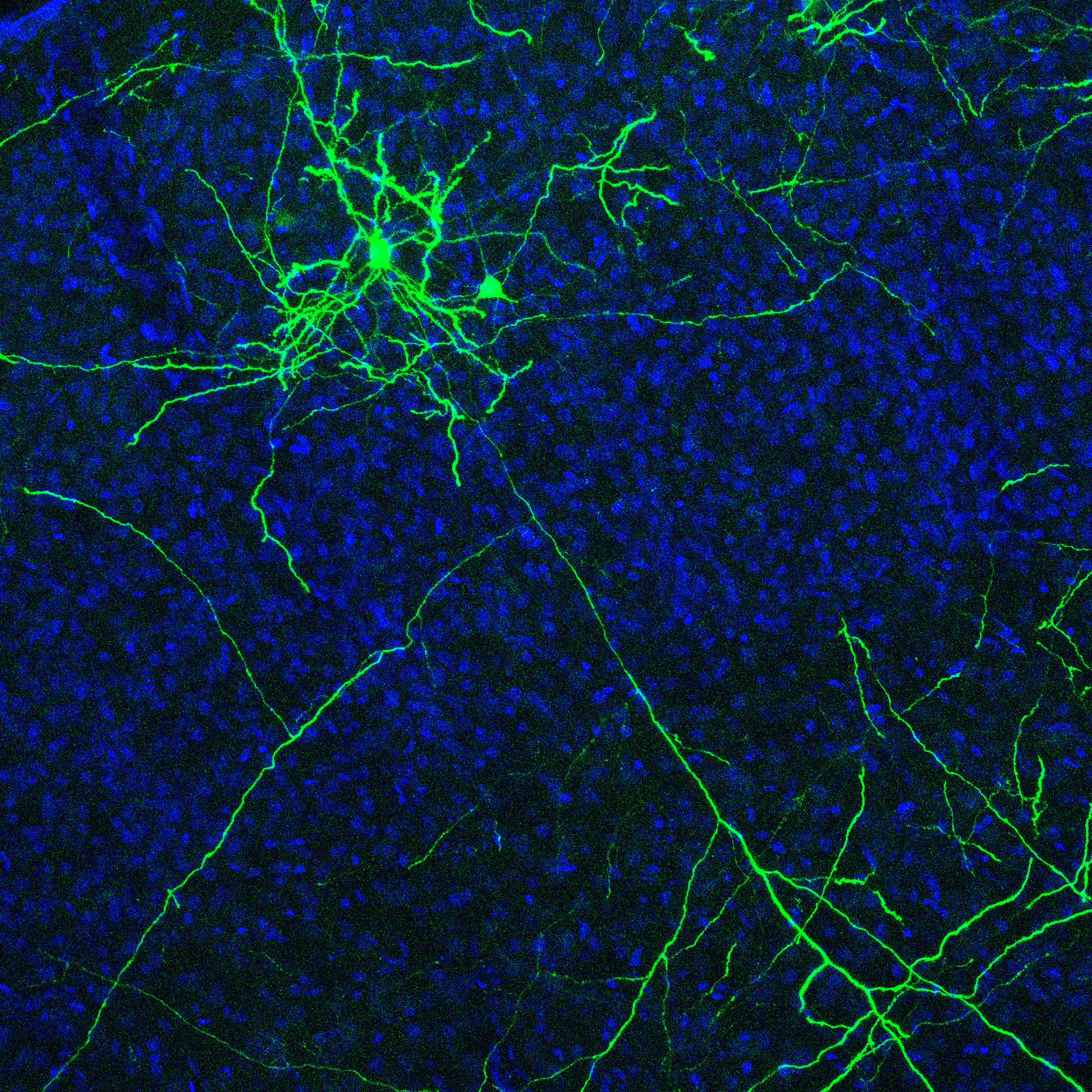

Supplement: Supplementary file 7 — Source Data Fig. 3 [file 44318_2024_50_MOESM7_ESM.zip › Figure3-source files/Figure3A-MAX_MAP1Bdephospho.tif]

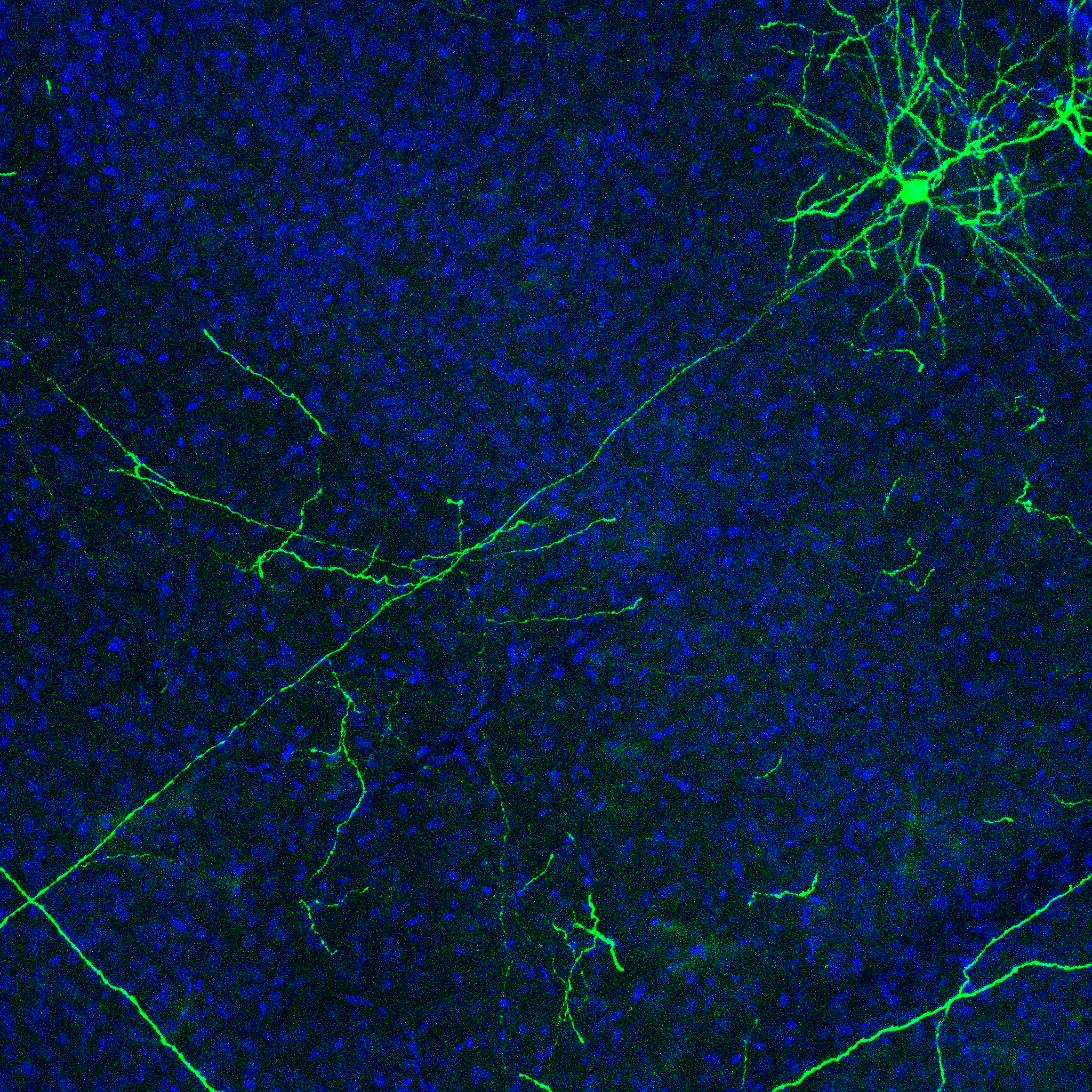

Supplement: Supplementary file 7 — Source Data Fig. 3 [file 44318_2024_50_MOESM7_ESM.zip › Figure3-source files/Figure3A-MAX_MAP1B-GOF.tif]

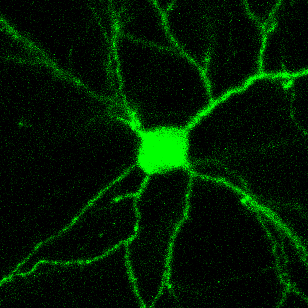

Supplement: Supplementary file 7 — Source Data Fig. 3 [file 44318_2024_50_MOESM7_ESM.zip › Figure3-source files/C1-MAX_MAP1B-GFP.tif]

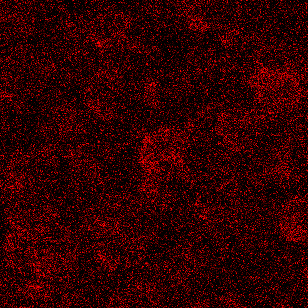

Supplement: Supplementary file 7 — Source Data Fig. 3 [file 44318_2024_50_MOESM7_ESM.zip › Figure3-source files/C3-MAX_MAP1B-Flag.tif]

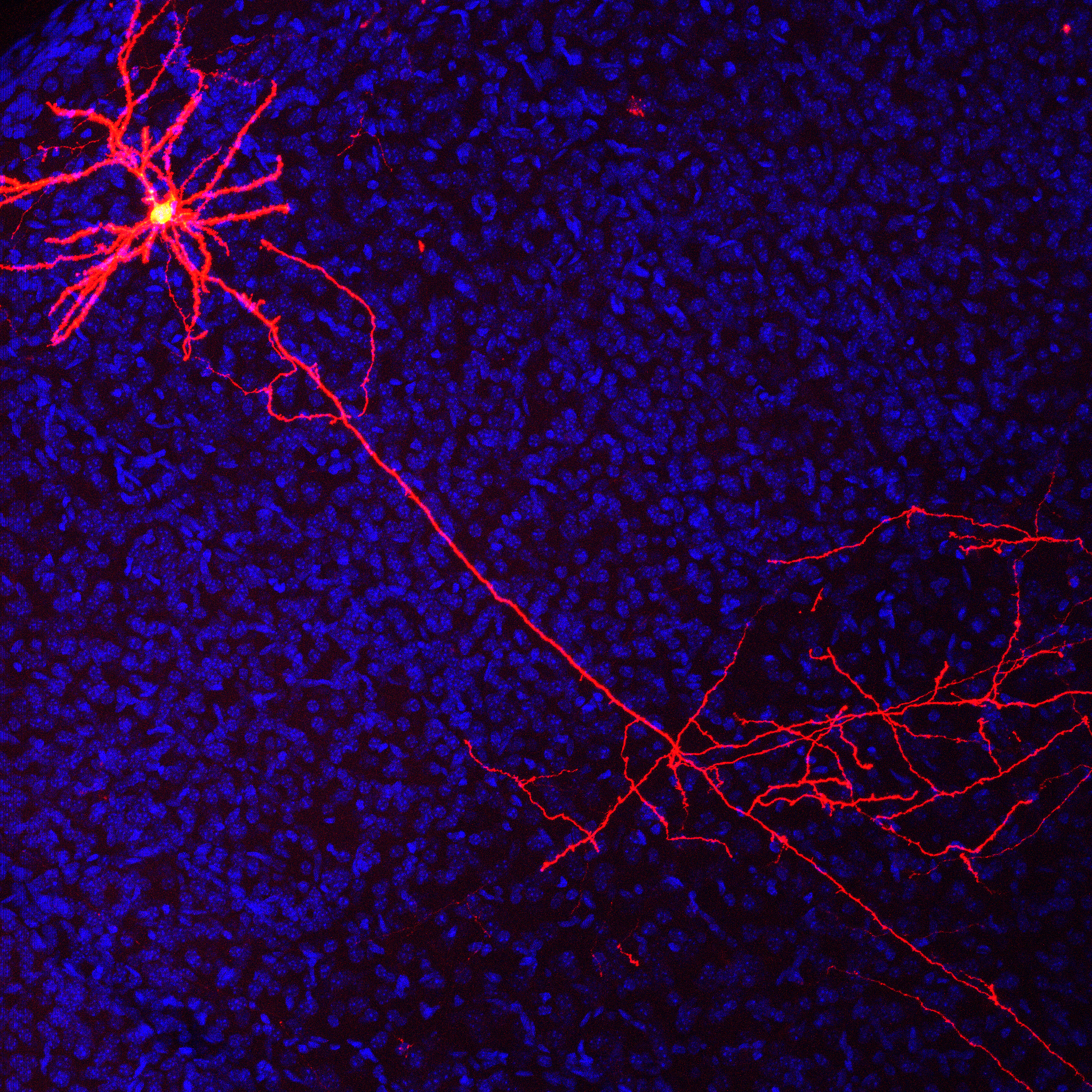

Supplement: Supplementary file 7 — Source Data Fig. 3 [file 44318_2024_50_MOESM7_ESM.zip › Figure3-source files/Figure3C-GSK3Btif.tif]

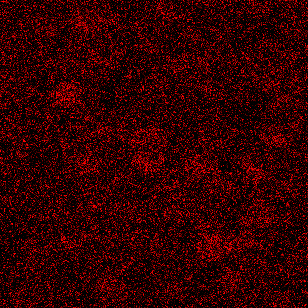

Supplement: Supplementary file 7 — Source Data Fig. 3 [file 44318_2024_50_MOESM7_ESM.zip › Figure3-source files/C2-MAX_MAP1B-dP-Flag.tif]

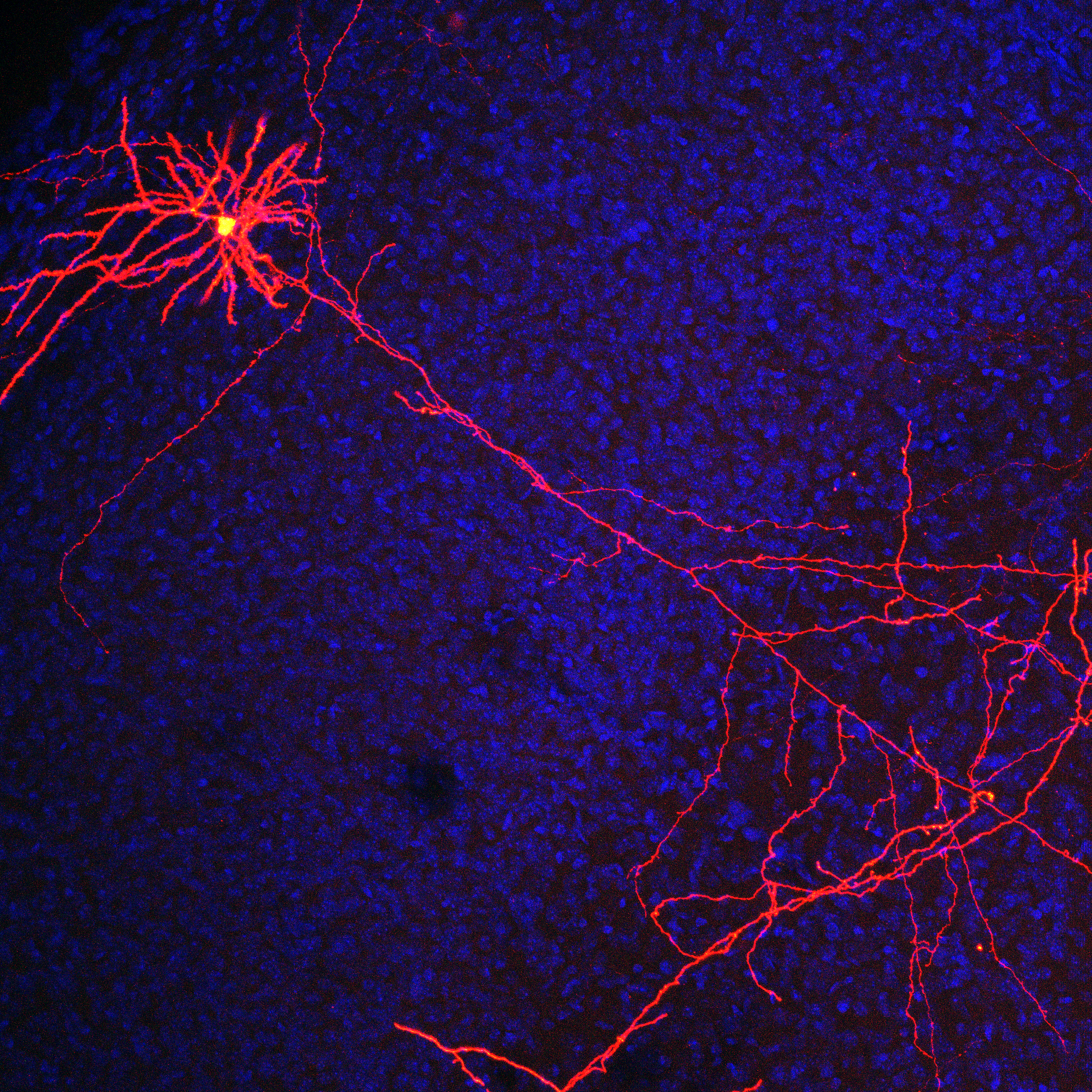

Supplement: Supplementary file 7 — Source Data Fig. 3 [file 44318_2024_50_MOESM7_ESM.zip › Figure3-source files/Figure3C-GSK3B-MAP1B.tif]

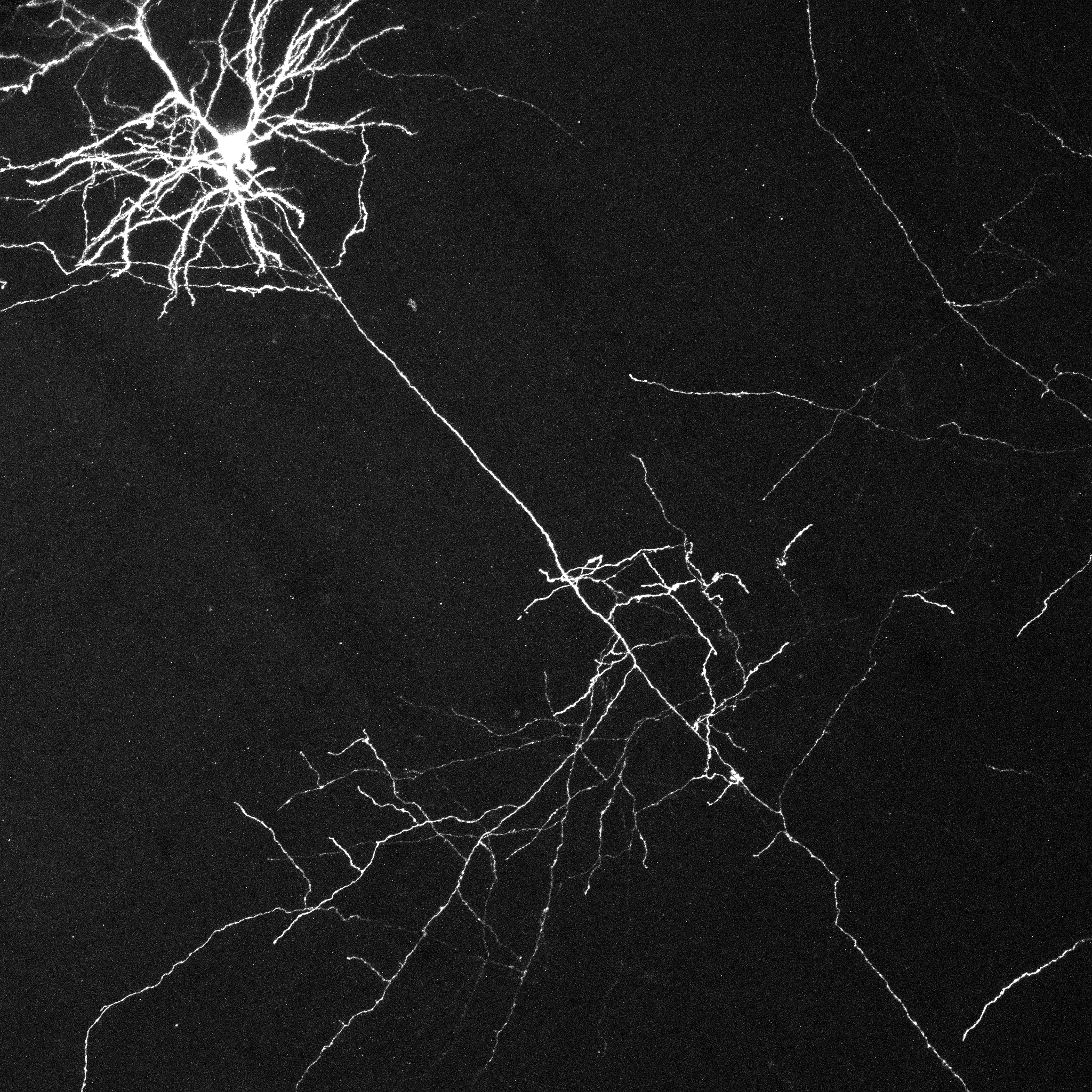

Supplement: Supplementary file 8 — Source Data Fig. 4 [file 44318_2024_50_MOESM8_ESM.zip › Figure4-source files/Figure4E-C2-MAX_Vash1-SVBP.tif]

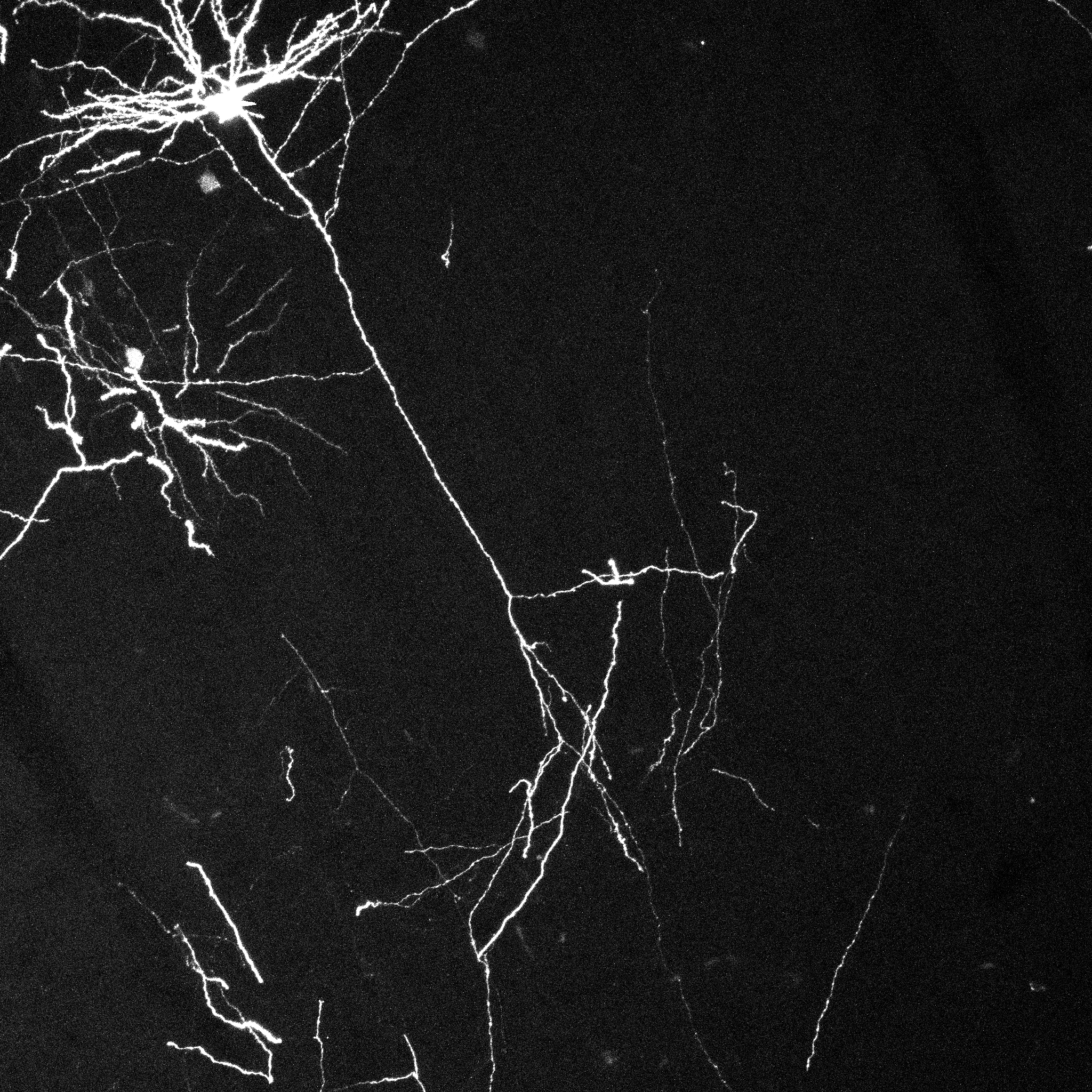

Supplement: Supplementary file 8 — Source Data Fig. 4 [file 44318_2024_50_MOESM8_ESM.zip › Figure4-source files/Figure4E-C2-MAX_control.tif]

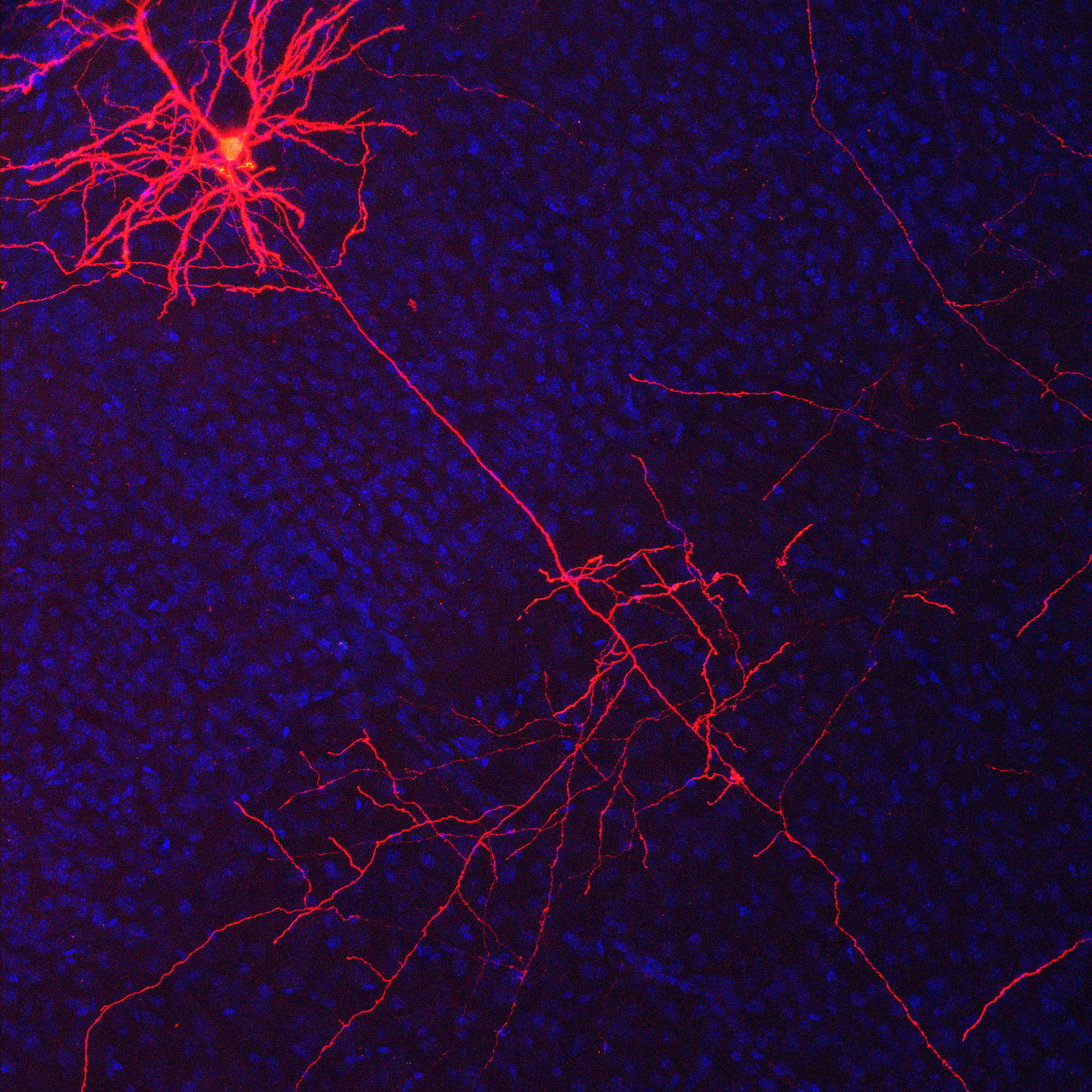

Supplement: Supplementary file 8 — Source Data Fig. 4 [file 44318_2024_50_MOESM8_ESM.zip › Figure4-source files/Figure4C-VASH1-SVBP.tif]

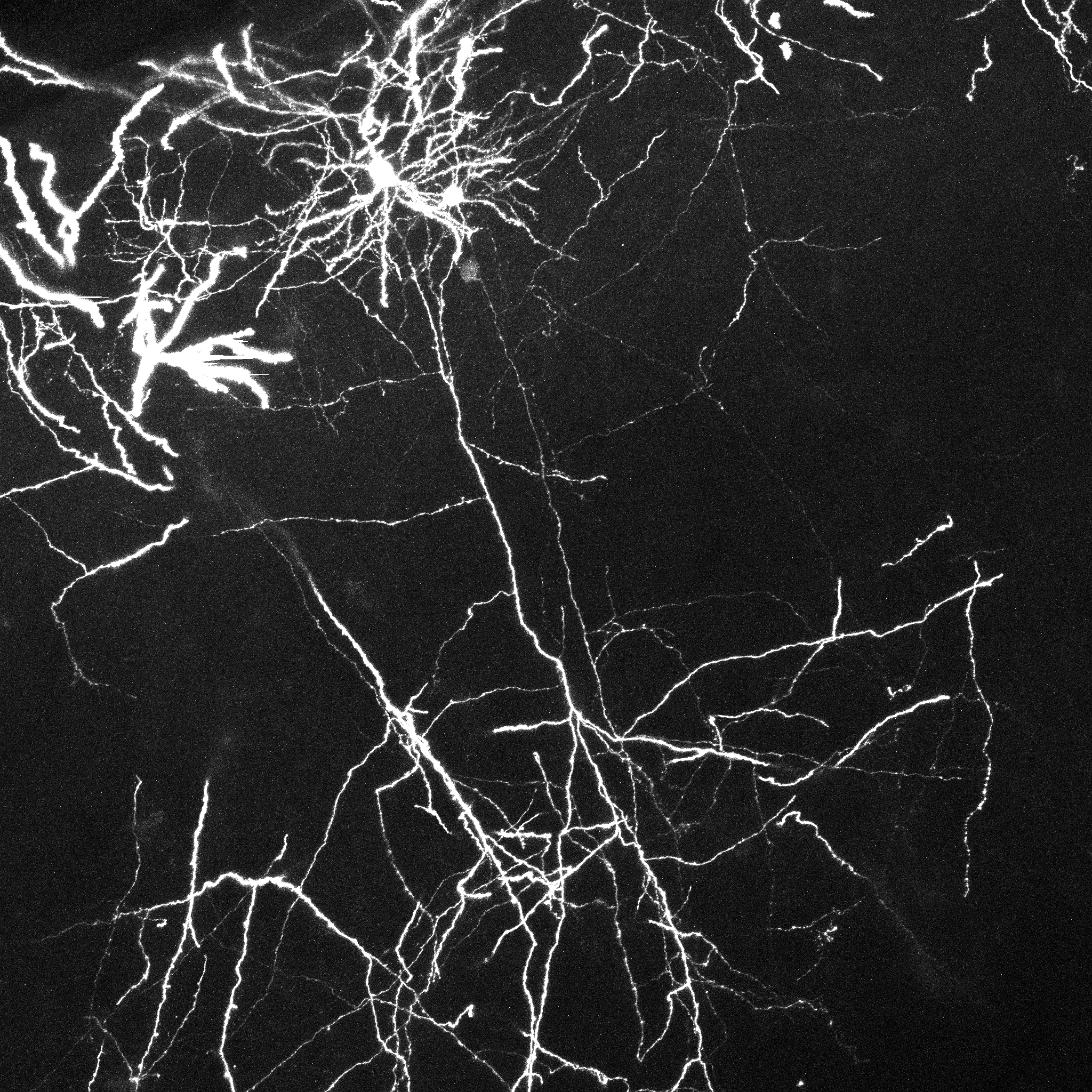

Supplement: Supplementary file 8 — Source Data Fig. 4 [file 44318_2024_50_MOESM8_ESM.zip › Figure4-source files/Figure4E-C2-MAX_TTL.tif]

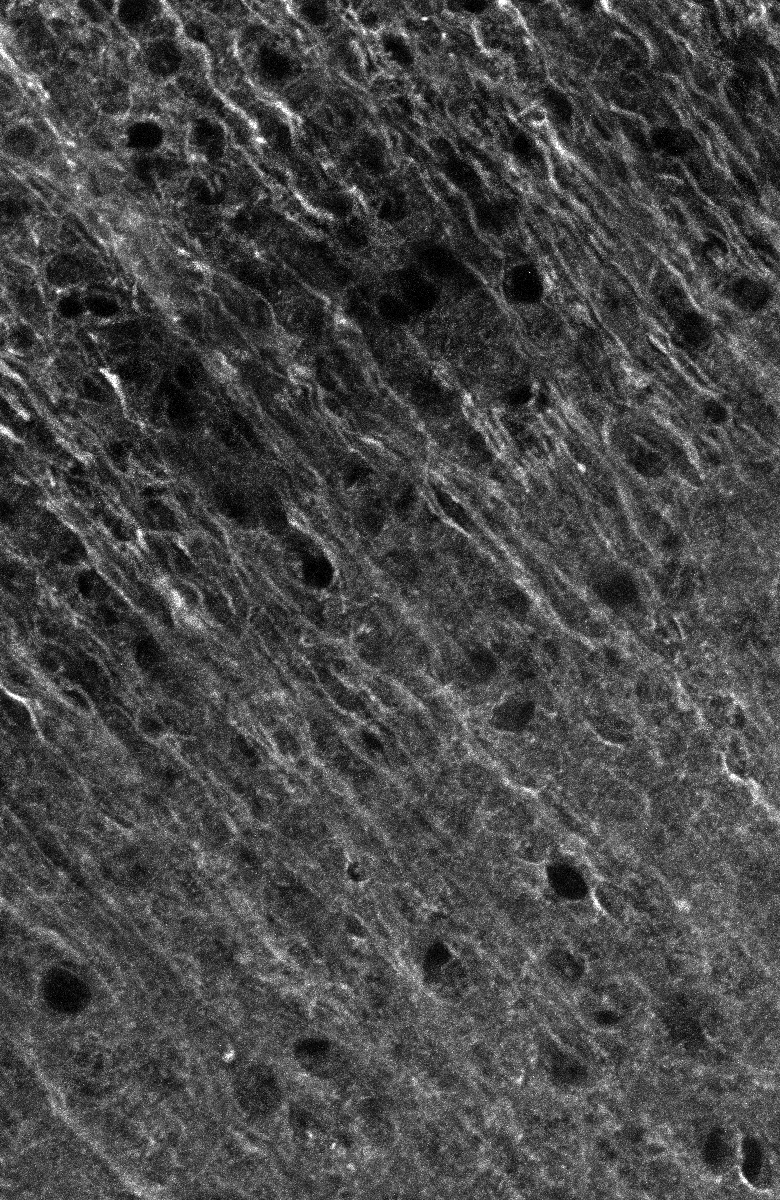

Supplement: Supplementary file 8 — Source Data Fig. 4 [file 44318_2024_50_MOESM8_ESM.zip › Figure4-source files/MAX_exp418-RFP-tubulin-detail.tif]

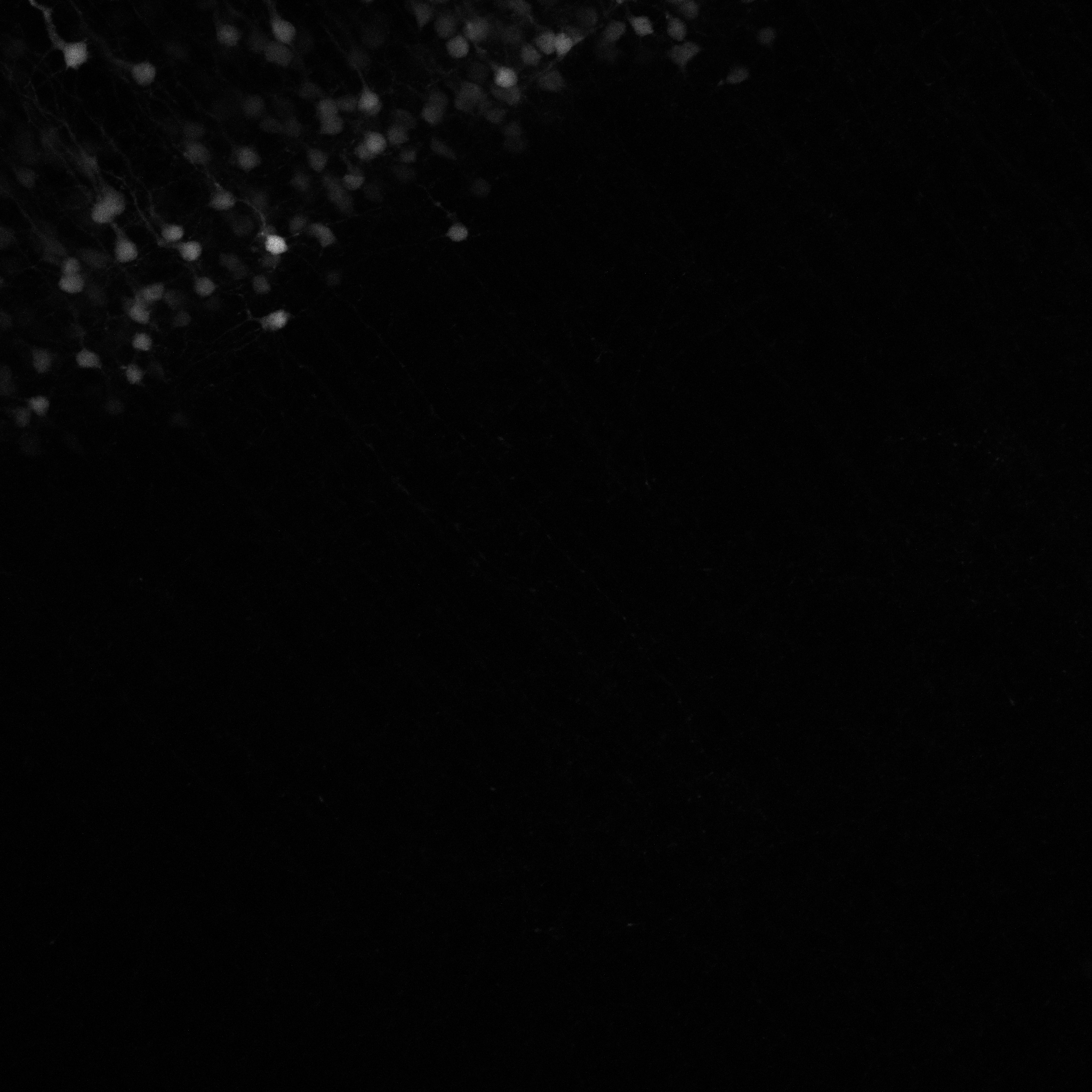

Supplement: Supplementary file 8 — Source Data Fig. 4 [file 44318_2024_50_MOESM8_ESM.zip › Figure4-source files/MAX_exp418-RFP-tubulin.tif]

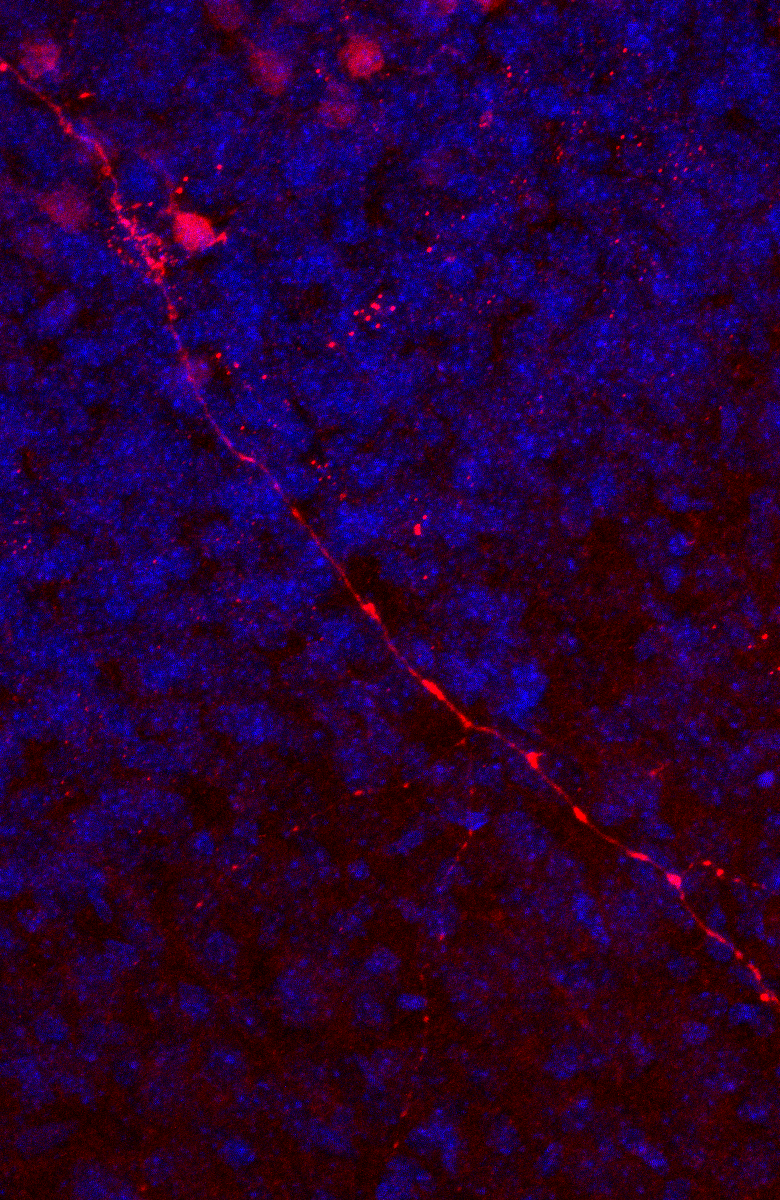

Supplement: Supplementary file 8 — Source Data Fig. 4 [file 44318_2024_50_MOESM8_ESM.zip › Figure4-source files/MAX_exp418-RFP-tubulin-RFPdetail.tif]

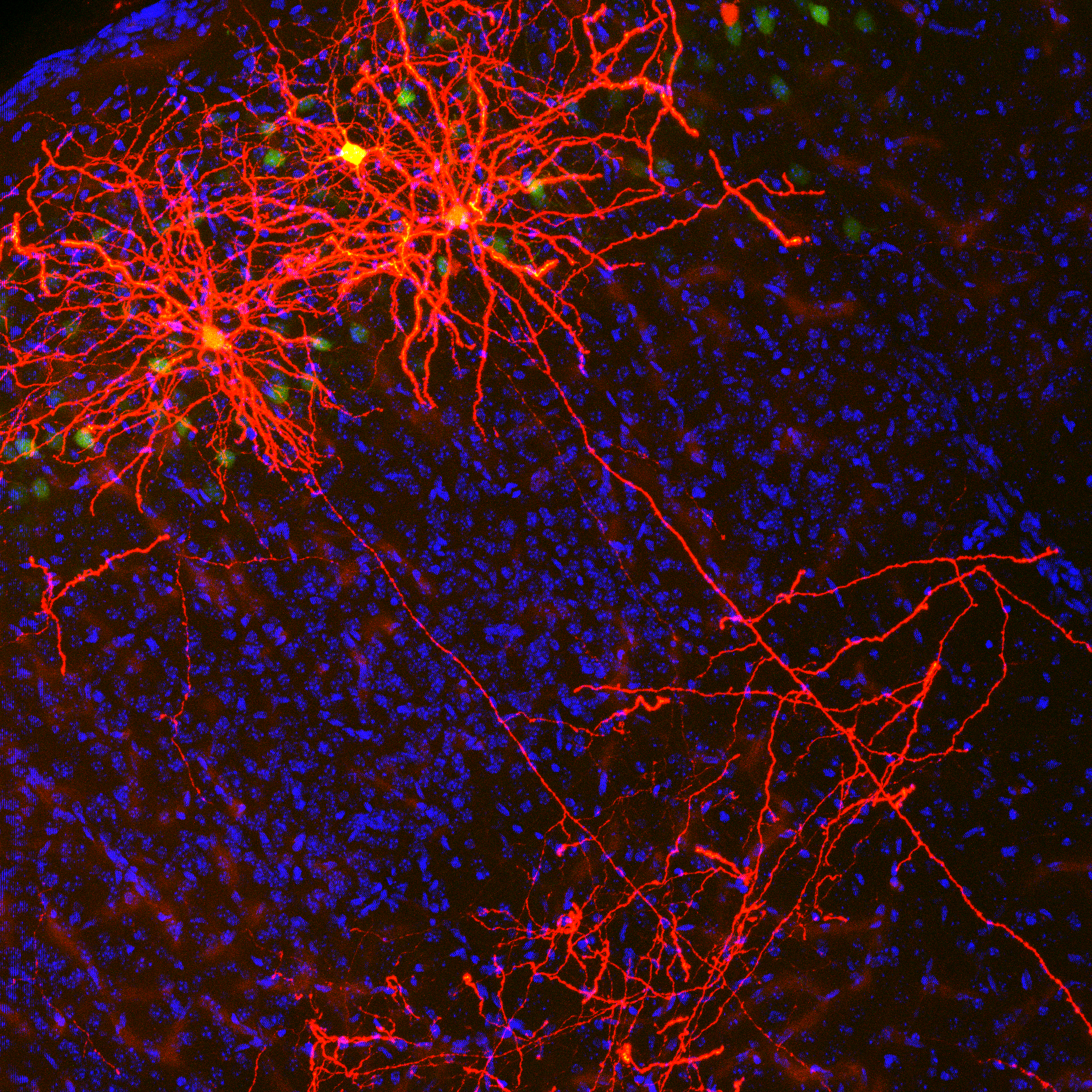

Supplement: Supplementary file 9 — Source Data Fig. 5 [file 44318_2024_50_MOESM9_ESM.zip › Figure5-source files/Figure5A-TTL-gRNA.tif]

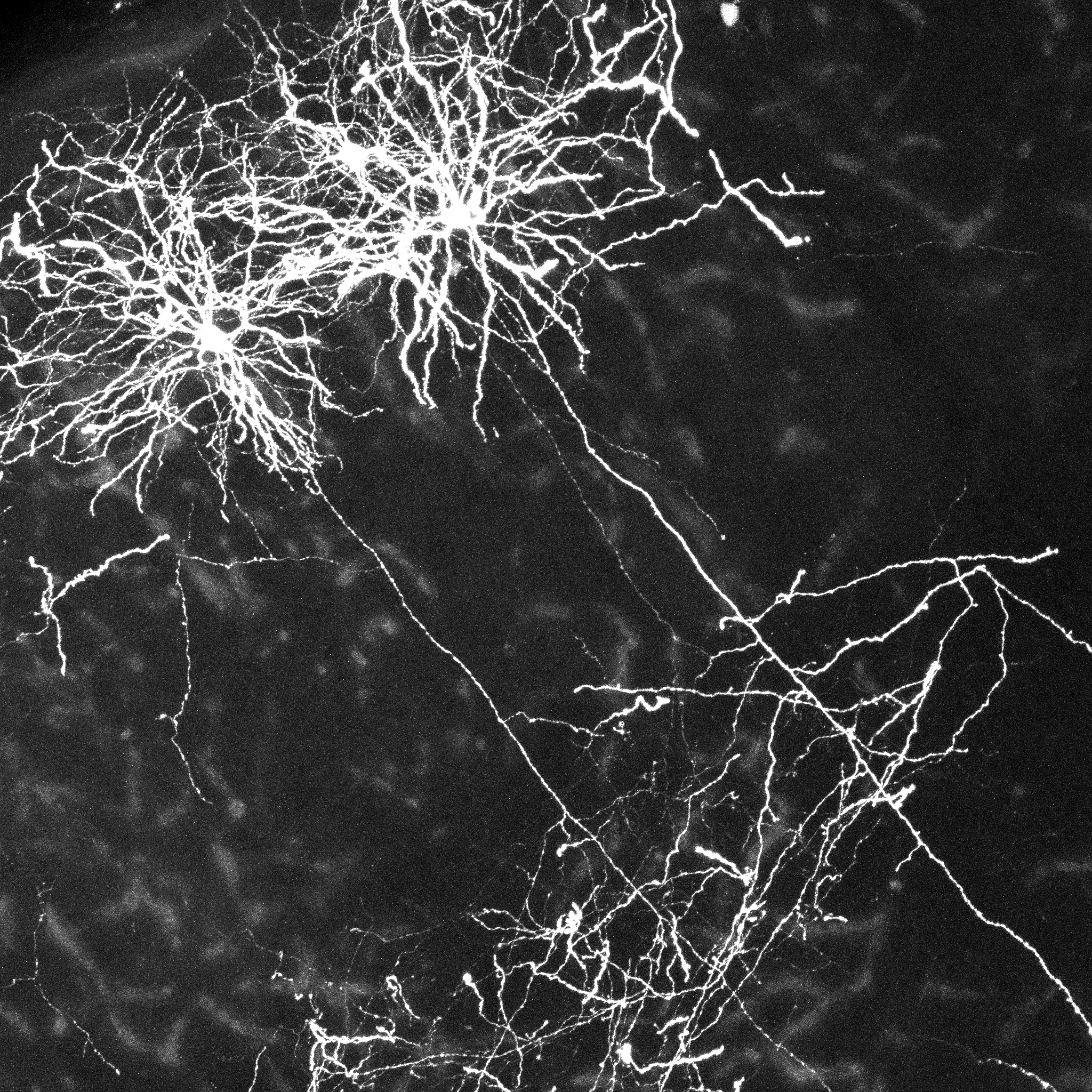

Supplement: Supplementary file 9 — Source Data Fig. 5 [file 44318_2024_50_MOESM9_ESM.zip › Figure5-source files/Figure5A-C2-MAX_TTL.tif]

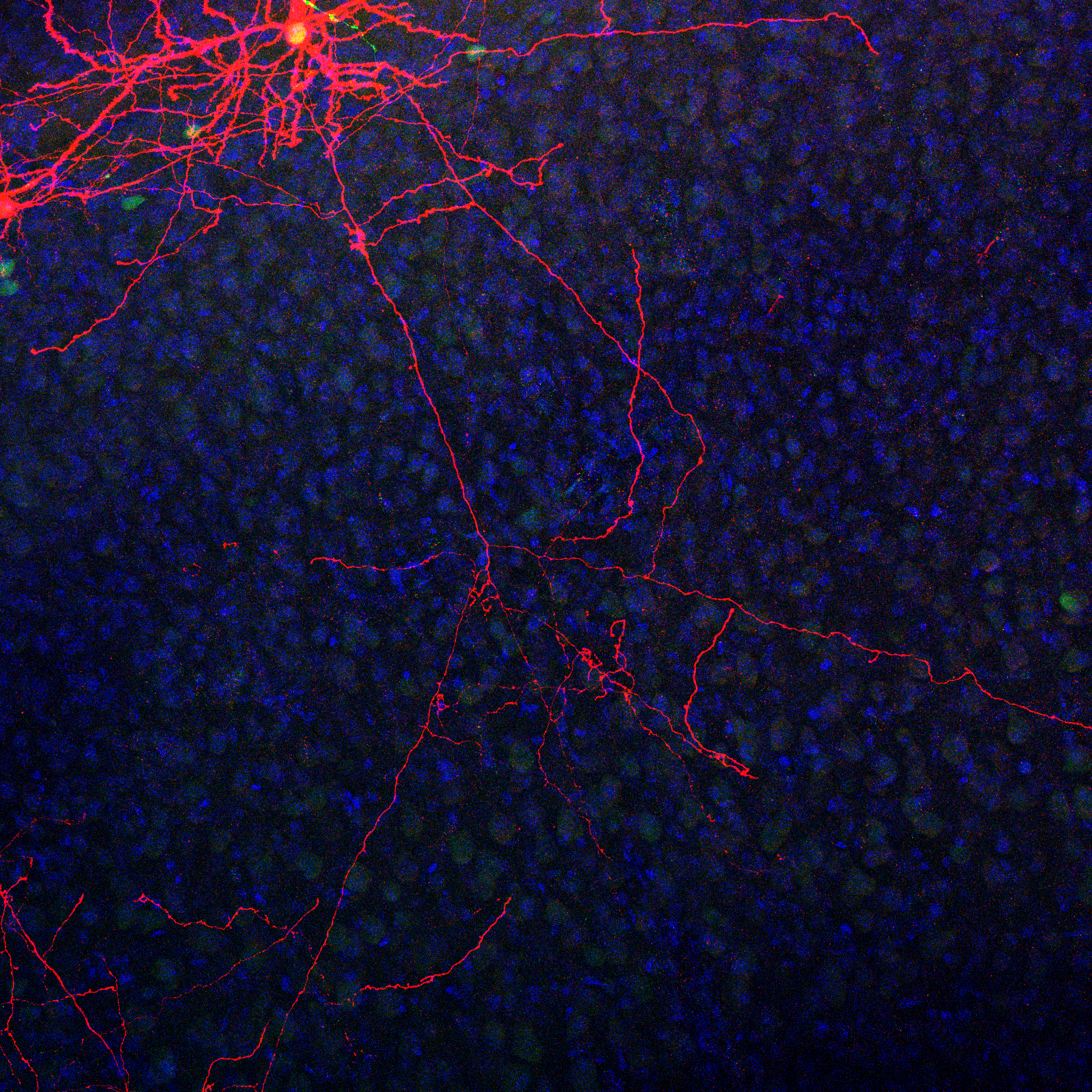

Supplement: Supplementary file 9 — Source Data Fig. 5 [file 44318_2024_50_MOESM9_ESM.zip › Figure5-source files/Figure5A-control-gRNA.tif]

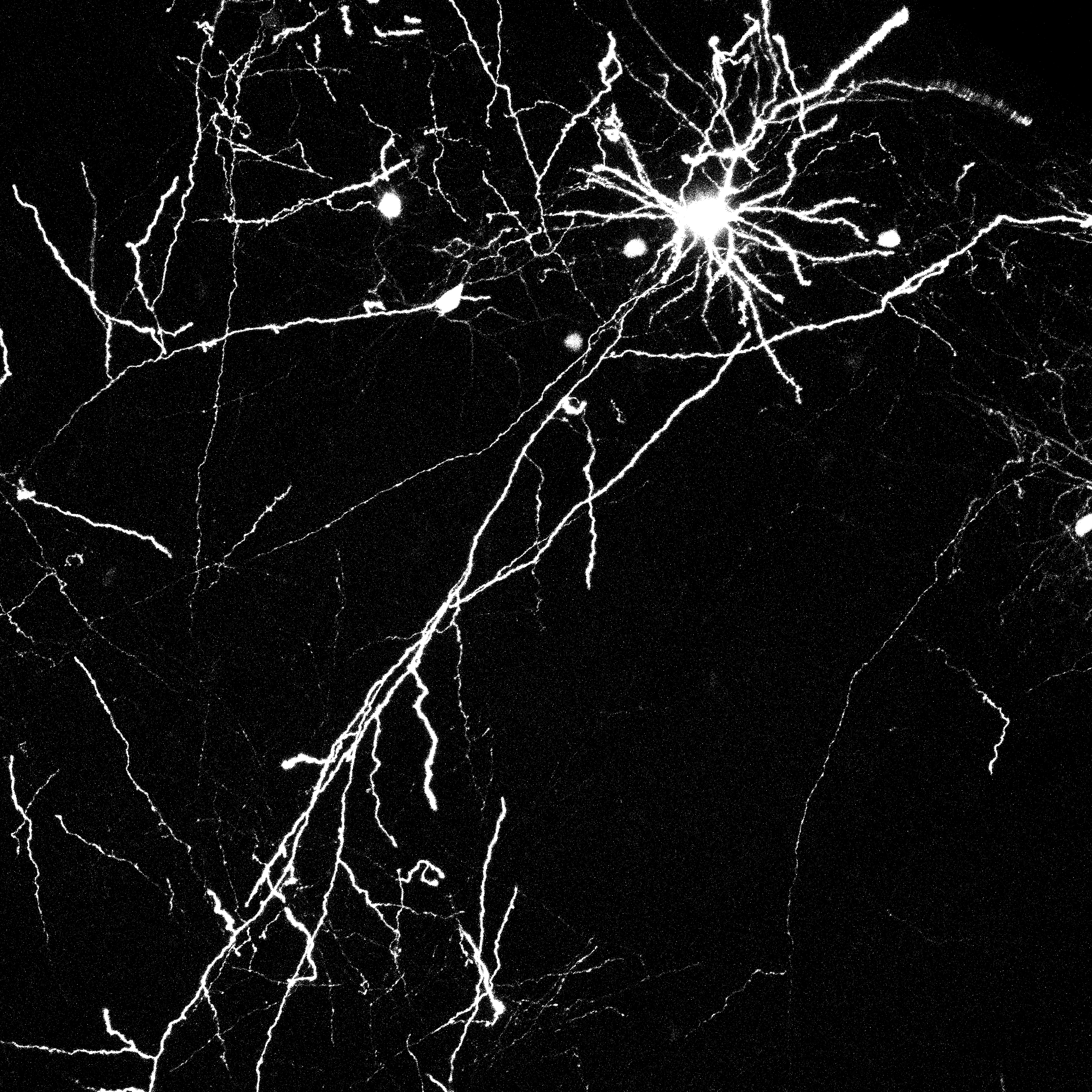

Supplement: Supplementary file 9 — Source Data Fig. 5 [file 44318_2024_50_MOESM9_ESM.zip › Figure5-source files/Figure5A-C2-MAX_SVBP.tif]

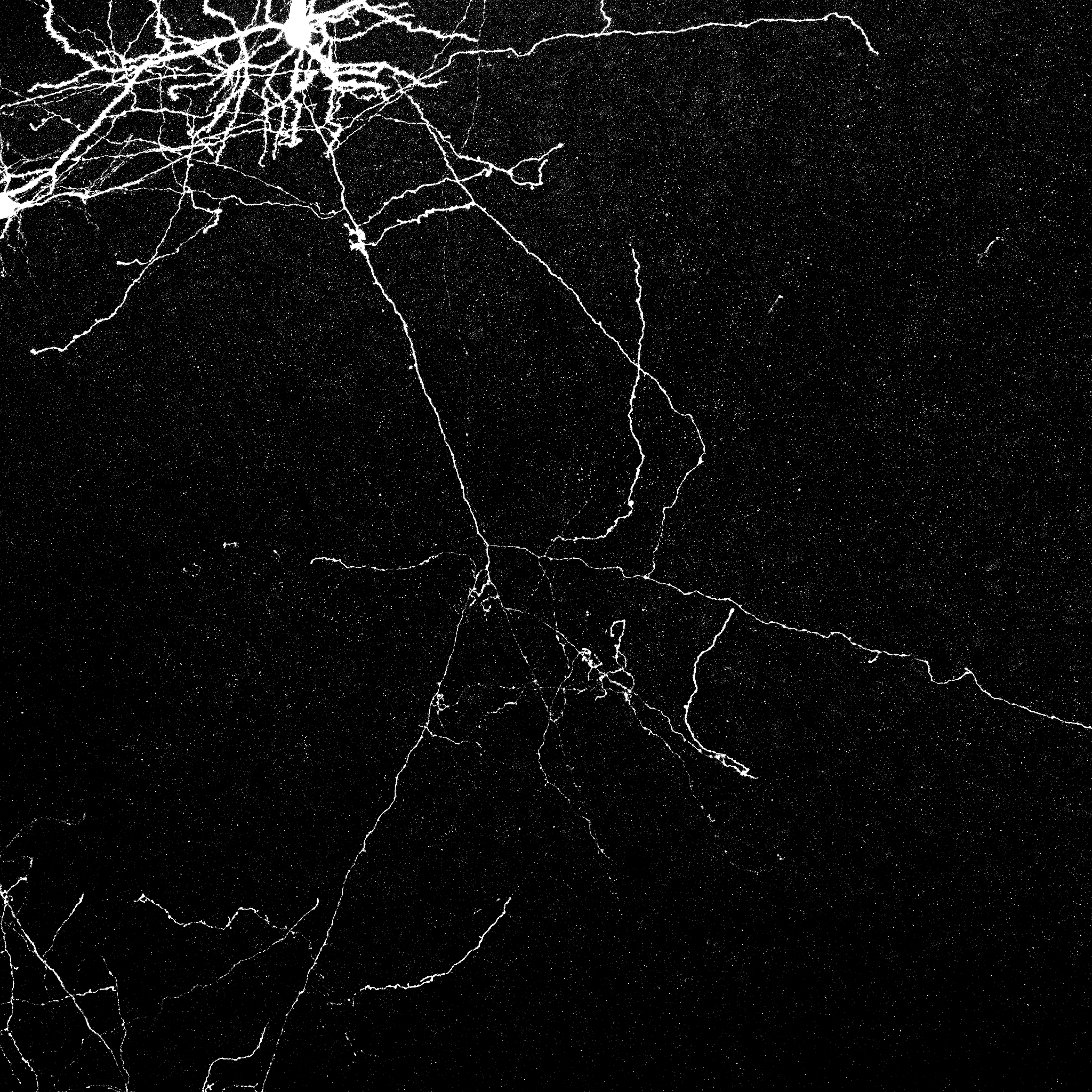

Supplement: Supplementary file 9 — Source Data Fig. 5 [file 44318_2024_50_MOESM9_ESM.zip › Figure5-source files/Figure5A-C2-MAX_control.tif]

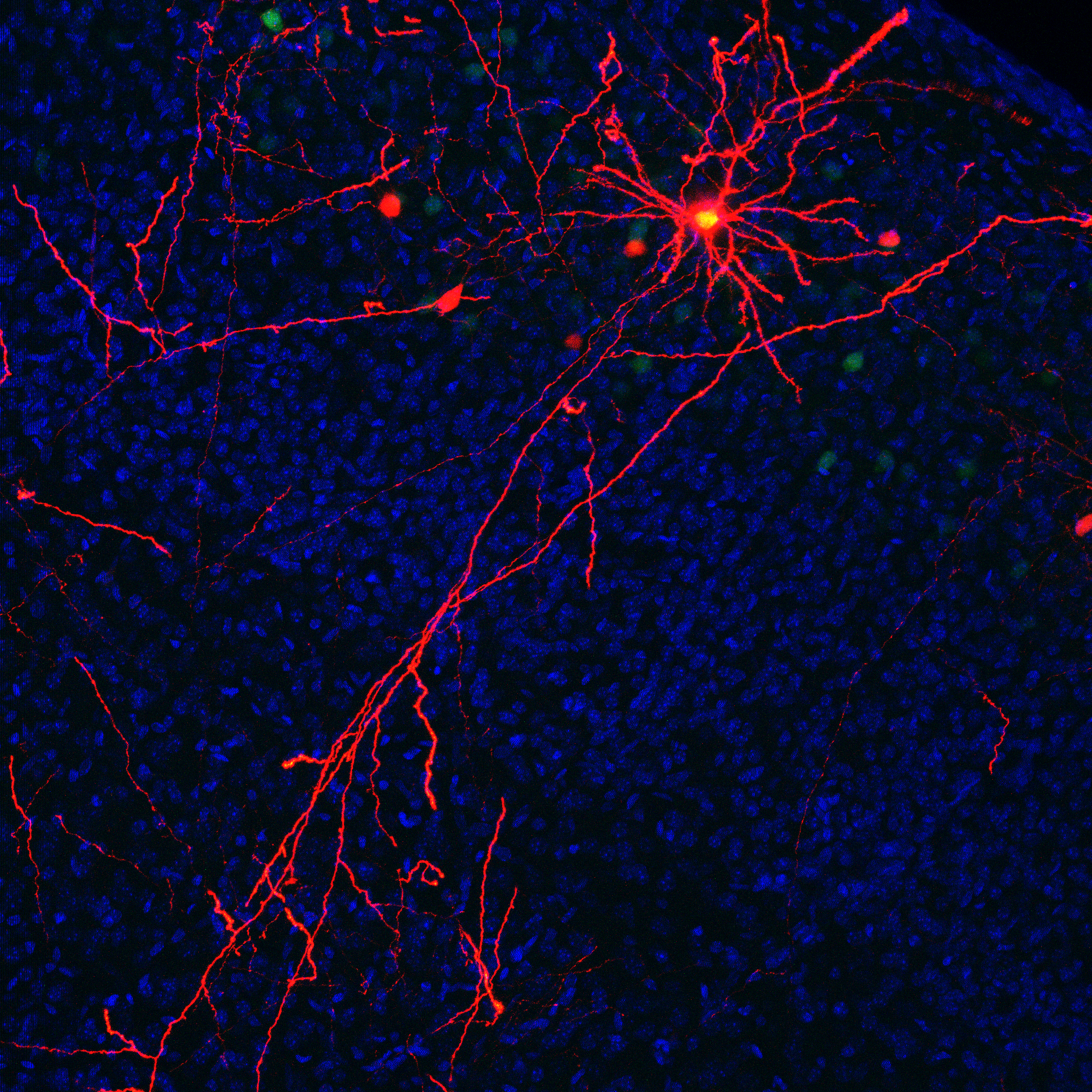

Supplement: Supplementary file 9 — Source Data Fig. 5 [file 44318_2024_50_MOESM9_ESM.zip › Figure5-source files/Figure5A-SVBP-gRNA.tif]

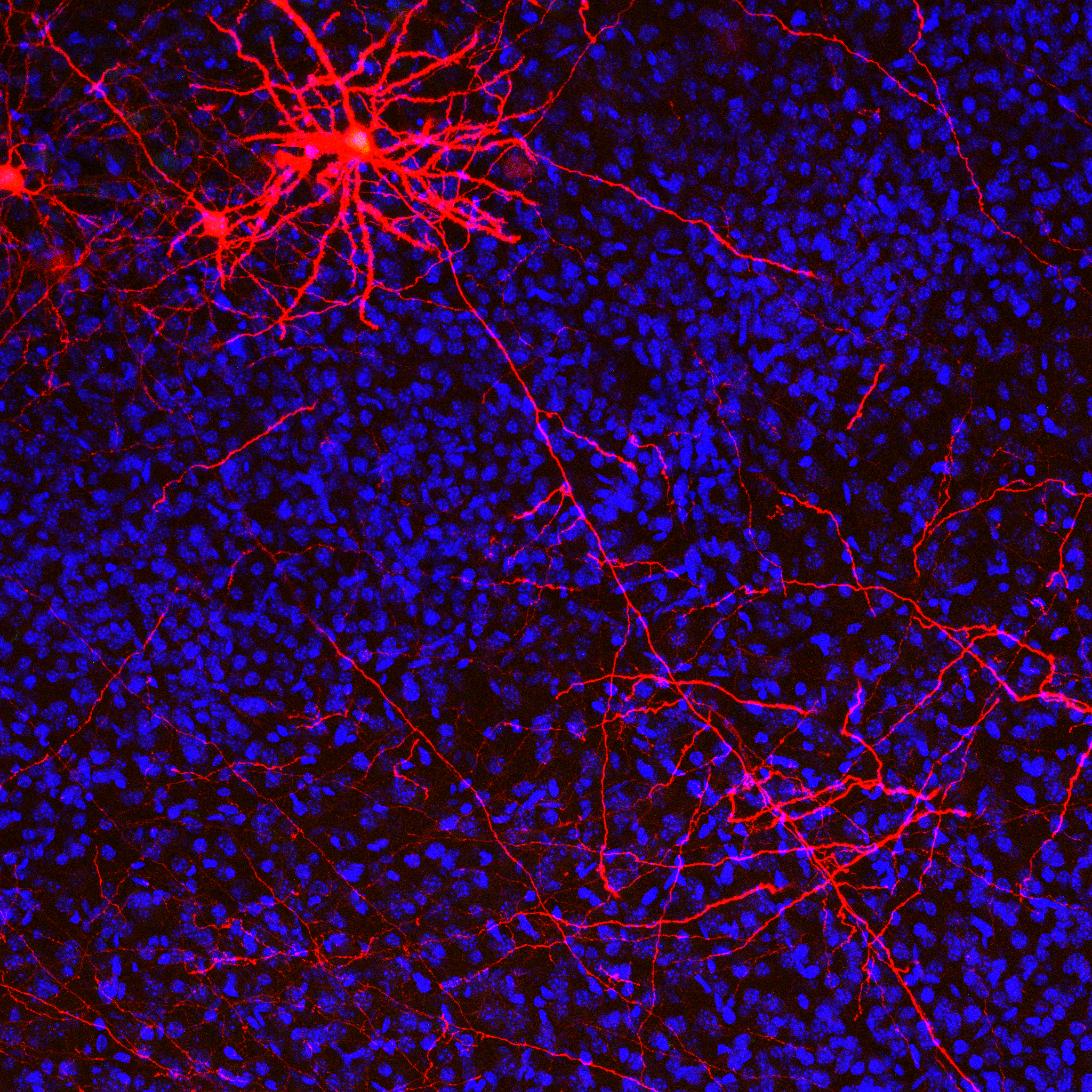

Supplement: Supplementary file 10 — Source Data Fig. 6 [file 44318_2024_50_MOESM10_ESM.zip › Figure6-source files/Figure6E-Map1bKO.tif]

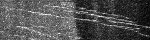

Supplement: Supplementary file 10 — Source Data Fig. 6 [file 44318_2024_50_MOESM10_ESM.zip › Figure6-source files/Figure6B-GSK3Binhib-kymograph2.tif]

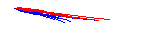

Supplement: Supplementary file 10 — Source Data Fig. 6 [file 44318_2024_50_MOESM10_ESM.zip › Figure6-source files/Figure6B-comparison-traces.tif]

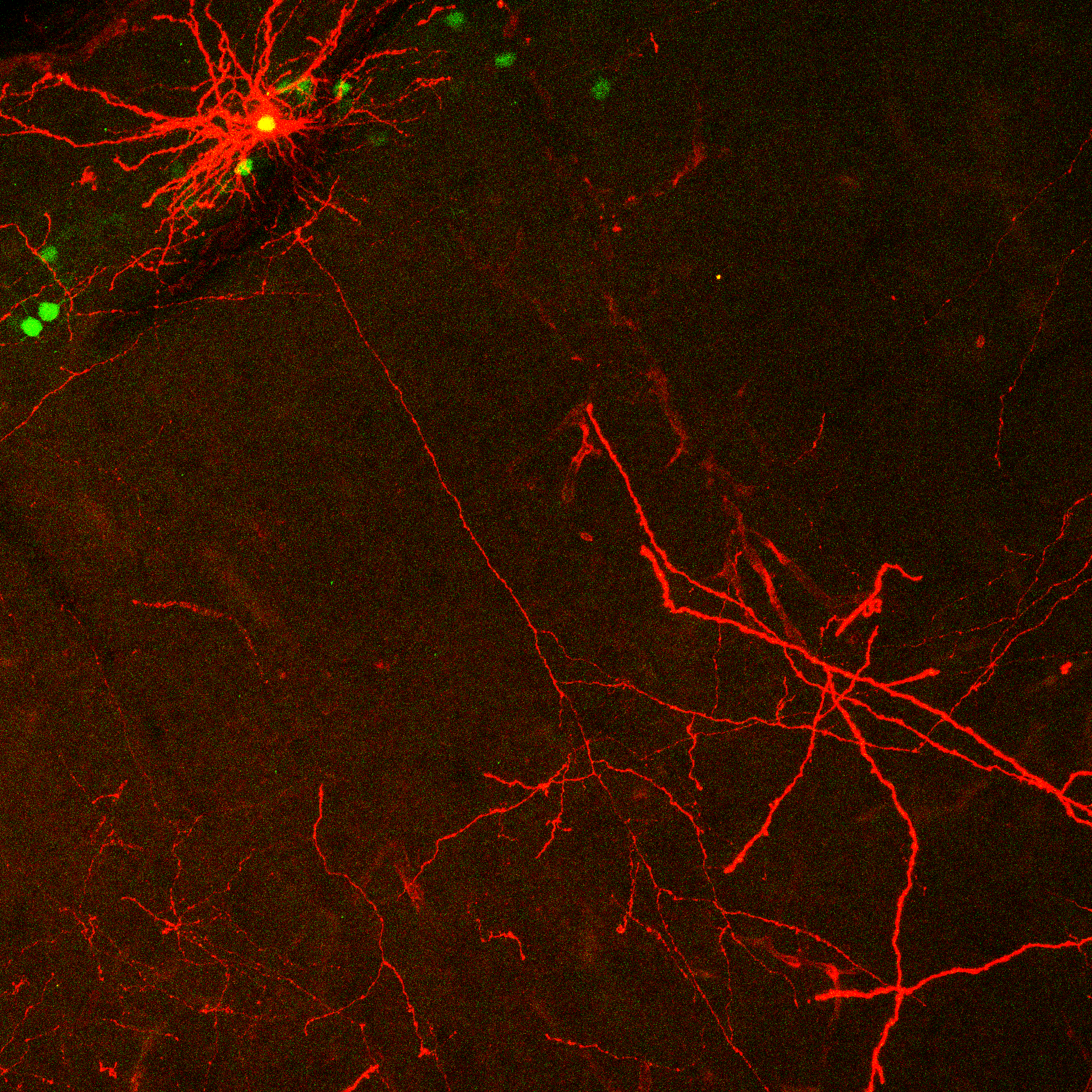

Supplement: Supplementary file 10 — Source Data Fig. 6 [file 44318_2024_50_MOESM10_ESM.zip › Figure6-source files/Figure6C-TtlgRNA-GSK3B.tif]

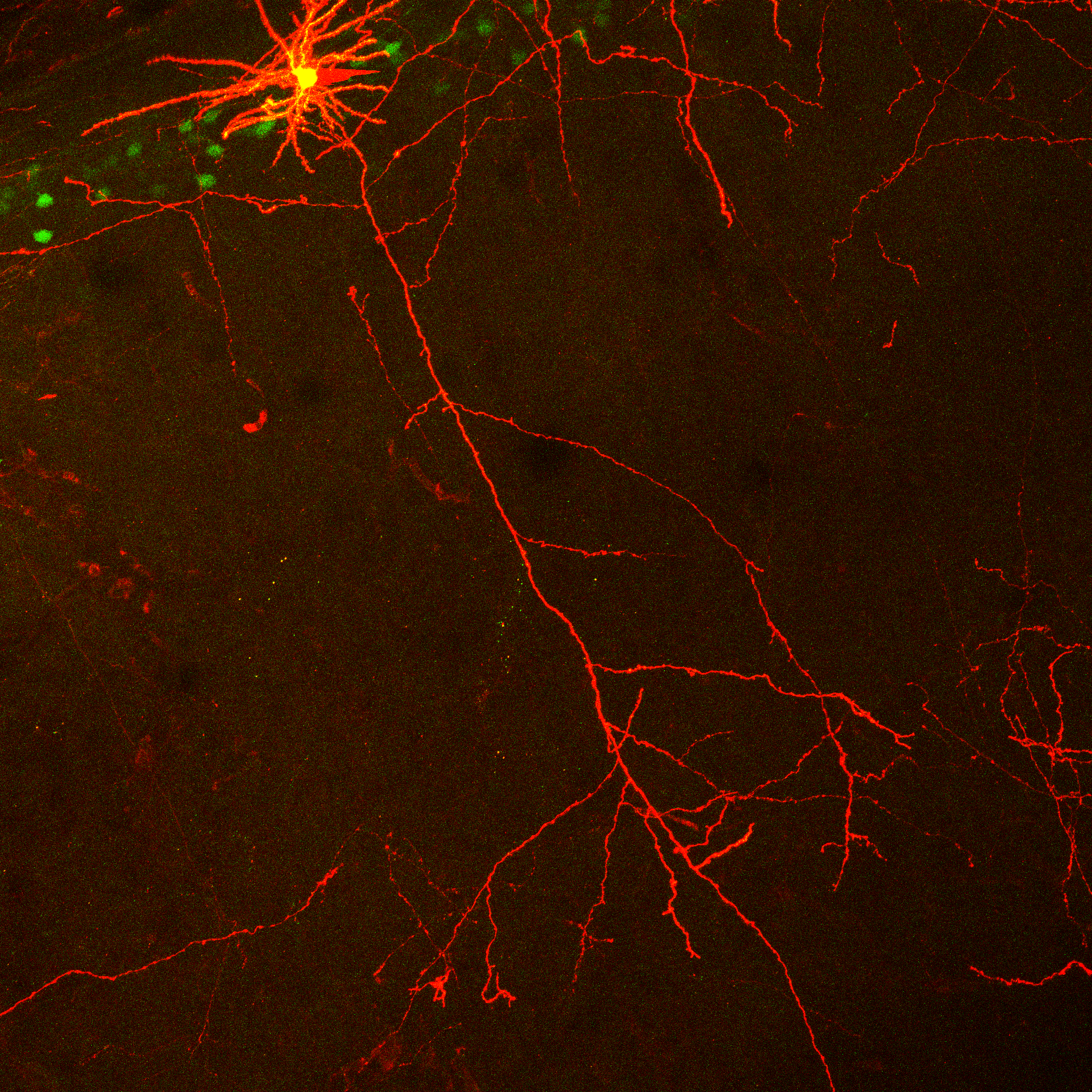

Supplement: Supplementary file 10 — Source Data Fig. 6 [file 44318_2024_50_MOESM10_ESM.zip › Figure6-source files/Figure6C-control-GSK3B.tif]

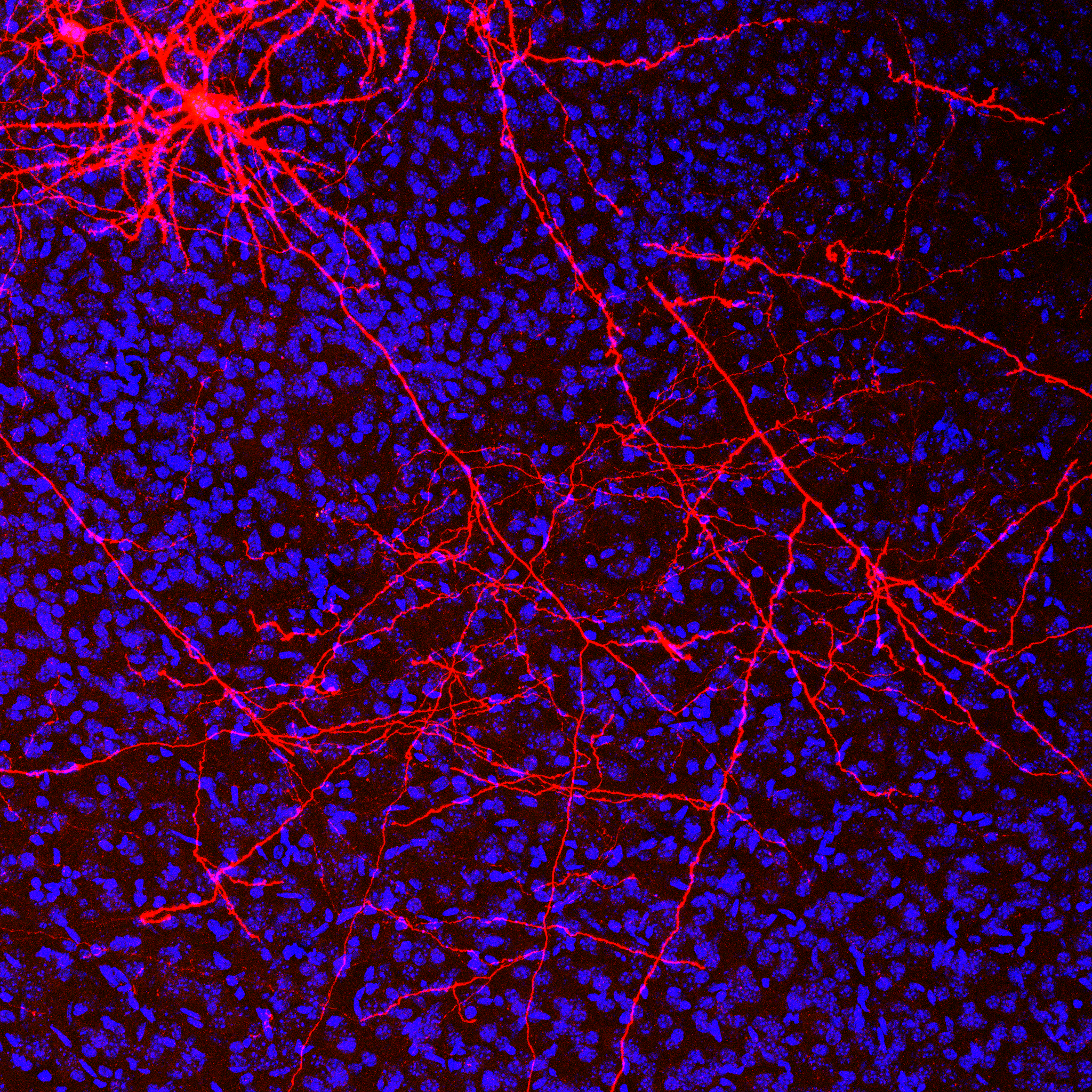

Supplement: Supplementary file 10 — Source Data Fig. 6 [file 44318_2024_50_MOESM10_ESM.zip › Figure6-source files/Figure6E-Map1b-TtlKO.tif]

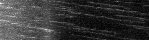

Supplement: Supplementary file 10 — Source Data Fig. 6 [file 44318_2024_50_MOESM10_ESM.zip › Figure6-source files/Figure6B-control-kymograph3.tif]

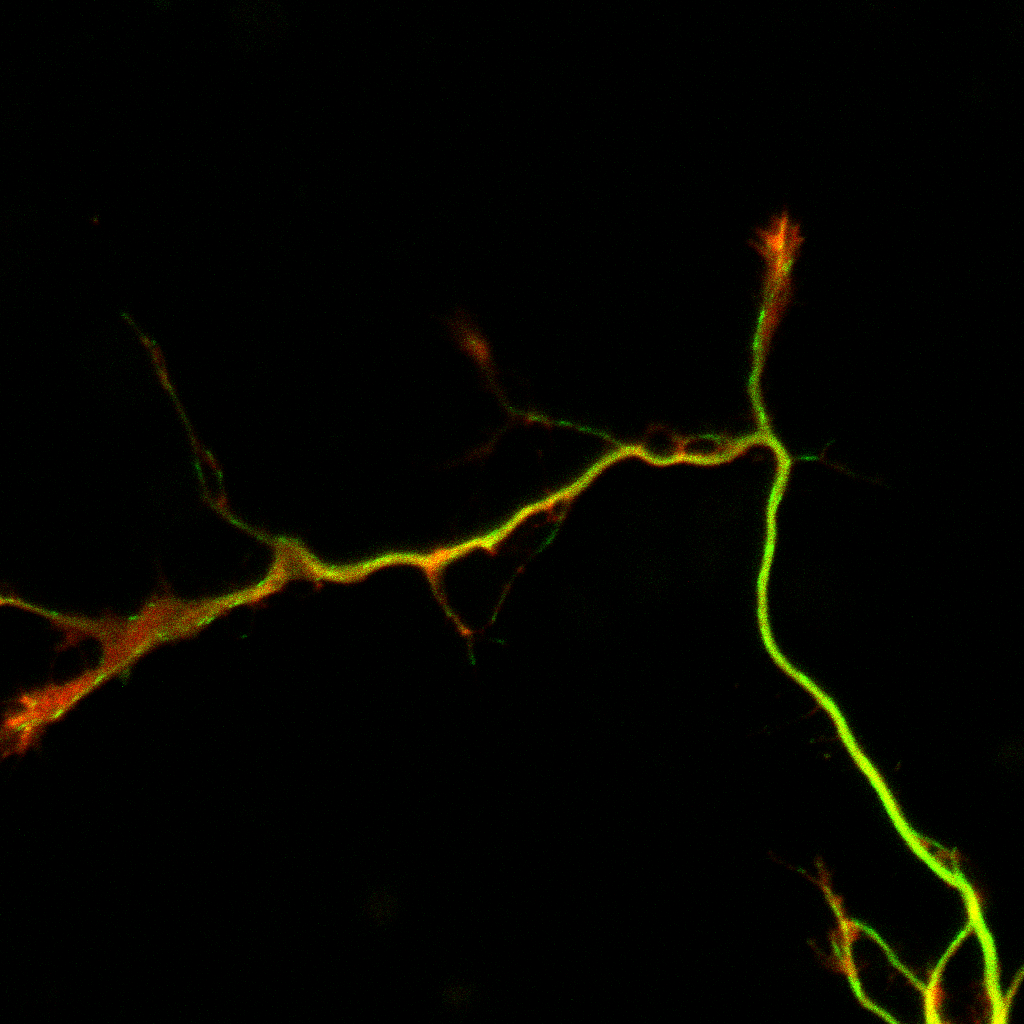

Supplement: Supplementary file 10 — Source Data Fig. 6 [file 44318_2024_50_MOESM10_ESM.zip › Figure6-source files/Figure6A-63x-EB3-control.tif]

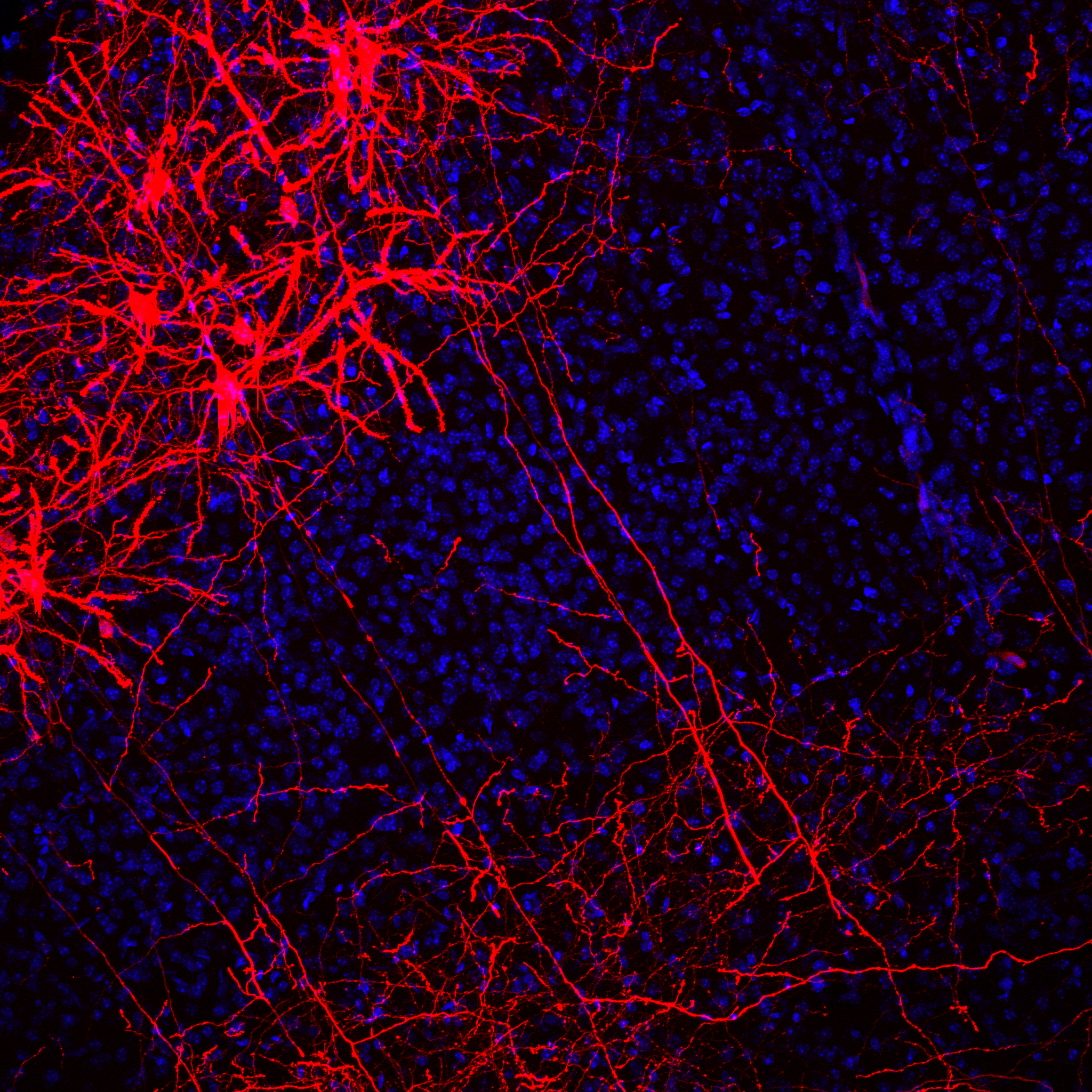

Supplement: Supplementary file 11 — EV Figures Source Data [file 44318_2024_50_MOESM11_ESM.zip › EV Figures-source files/FigureEV2-source files/MAX_777-mouse4-slice1-APC.tif]

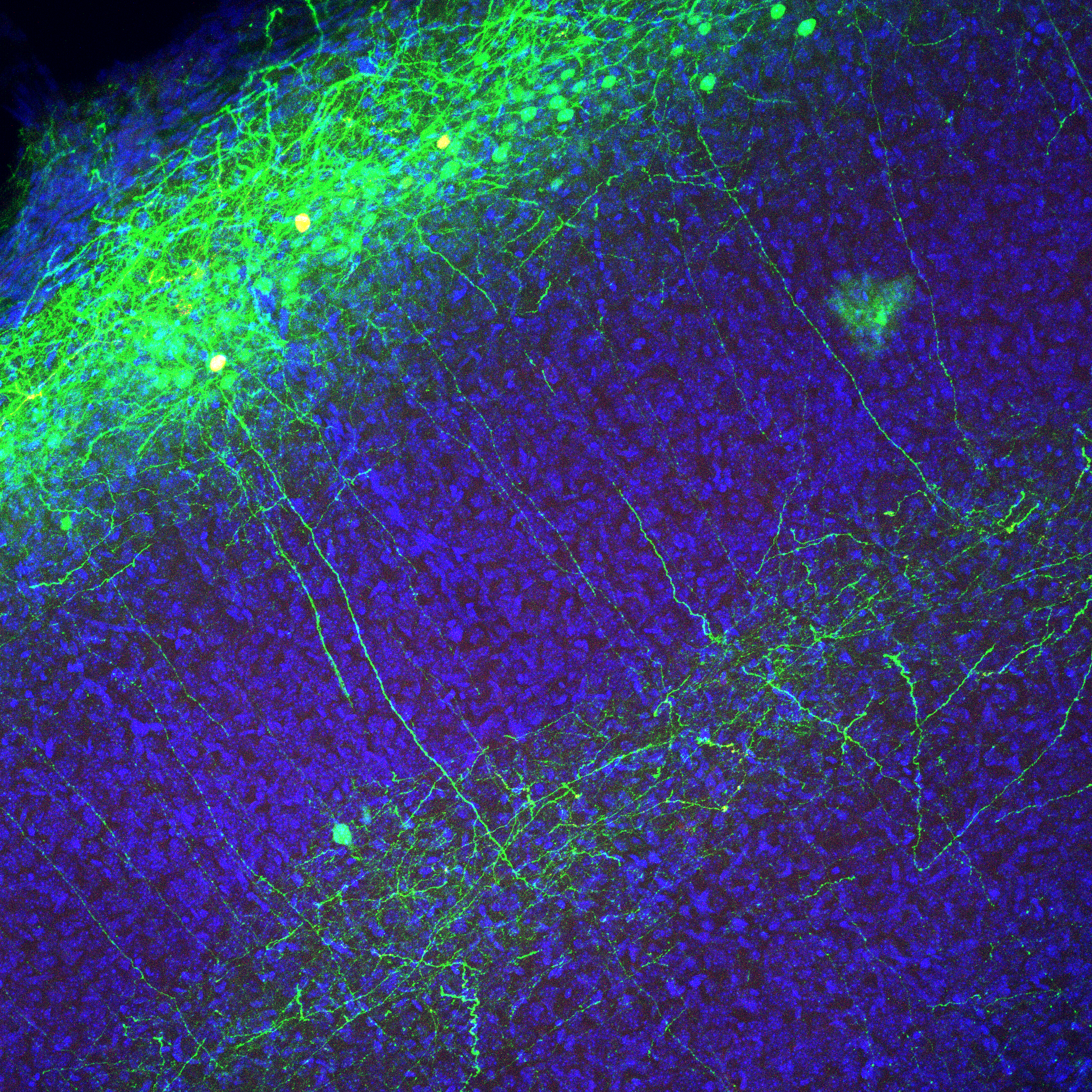

Supplement: Supplementary file 11 — EV Figures Source Data [file 44318_2024_50_MOESM11_ESM.zip › EV Figures-source files/FigureEV2-source files/MAX_exp82-R-mouse2-slice3-Clasp1.tif]

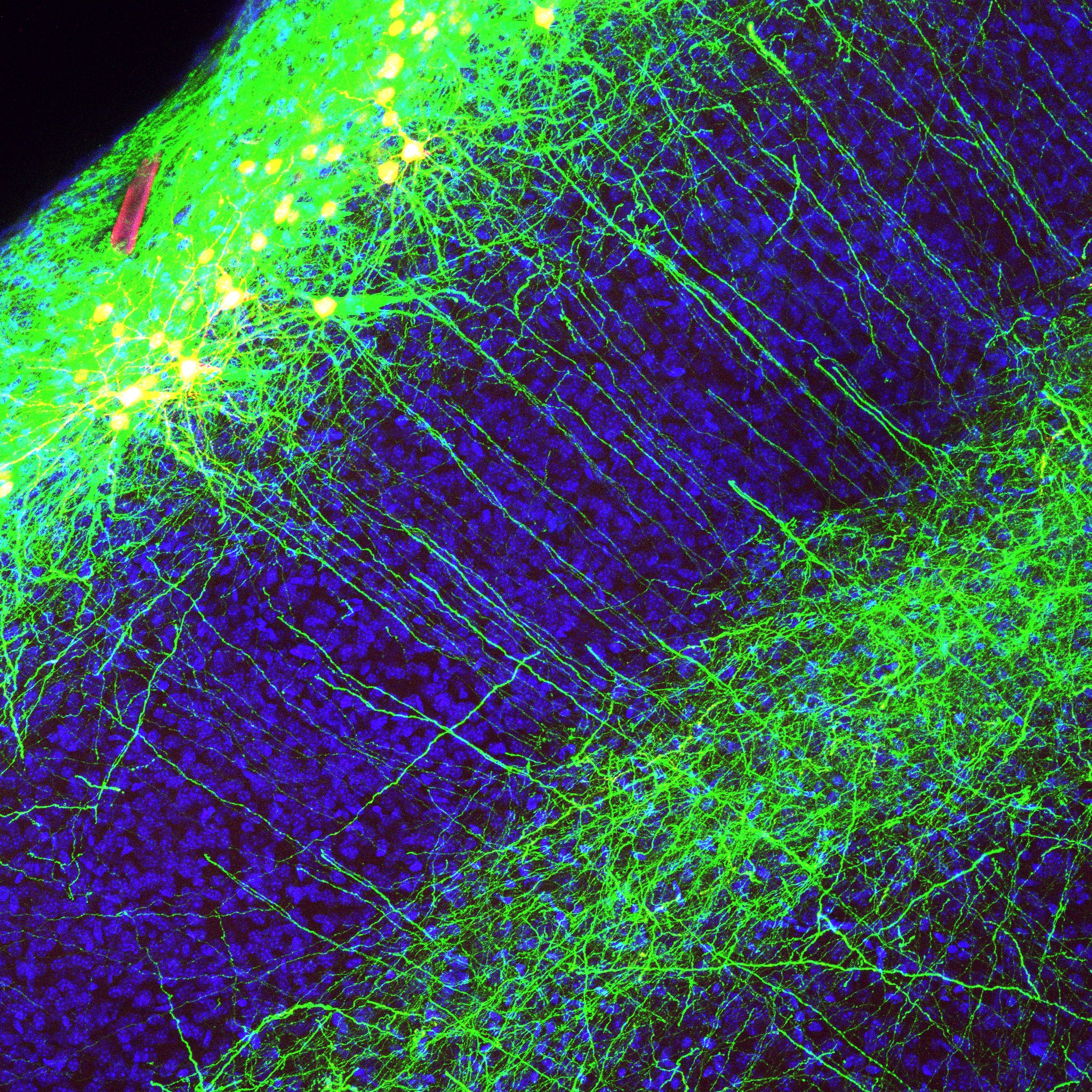

Supplement: Supplementary file 11 — EV Figures Source Data [file 44318_2024_50_MOESM11_ESM.zip › EV Figures-source files/FigureEV2-source files/MACF1-control-1shRNA.tif]

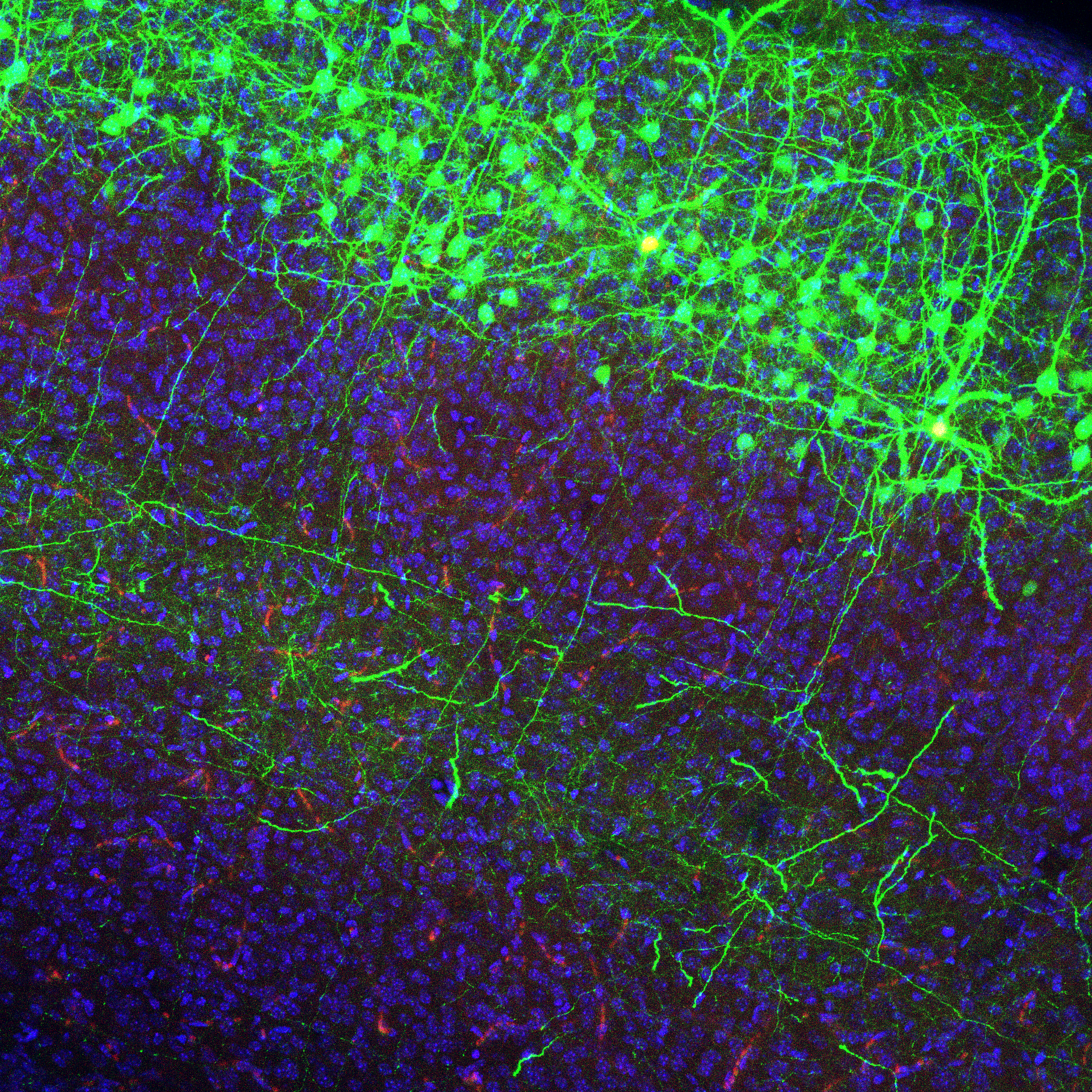

Supplement: Supplementary file 11 — EV Figures Source Data [file 44318_2024_50_MOESM11_ESM.zip › EV Figures-source files/FigureEV2-source files/MAX_exp83-R-mouse2-slice1-Clasp2.tif]

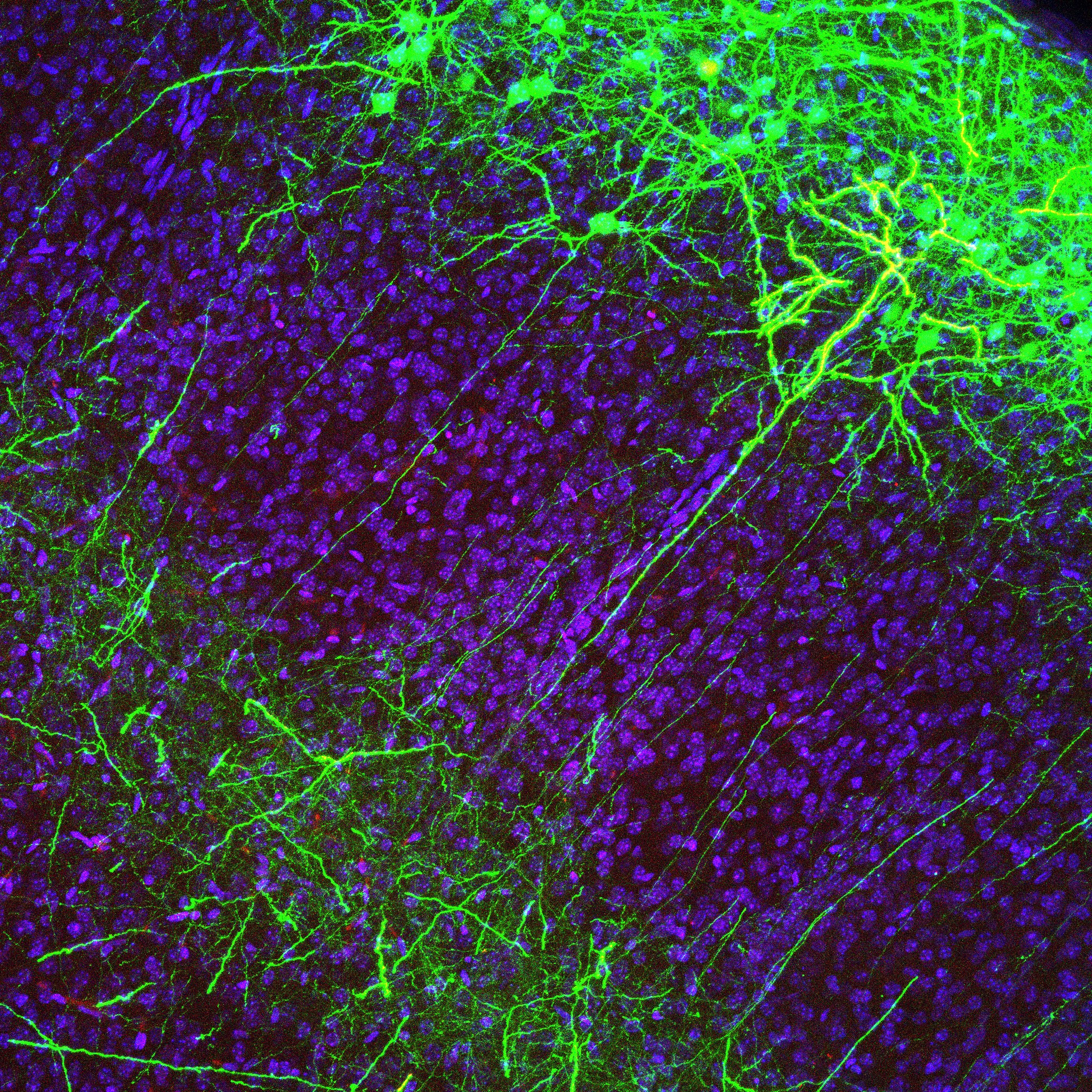

Supplement: Supplementary file 11 — EV Figures Source Data [file 44318_2024_50_MOESM11_ESM.zip › EV Figures-source files/FigureEV2-source files/MACF1-1shRNA.tif]

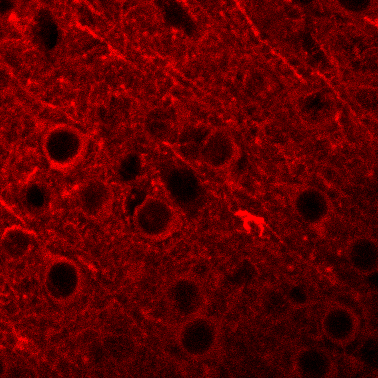

Supplement: Supplementary file 11 — EV Figures Source Data [file 44318_2024_50_MOESM11_ESM.zip › EV Figures-source files/FigureEV5-source files/FigEV5A-C2-exp285-L-example1-1.tif]

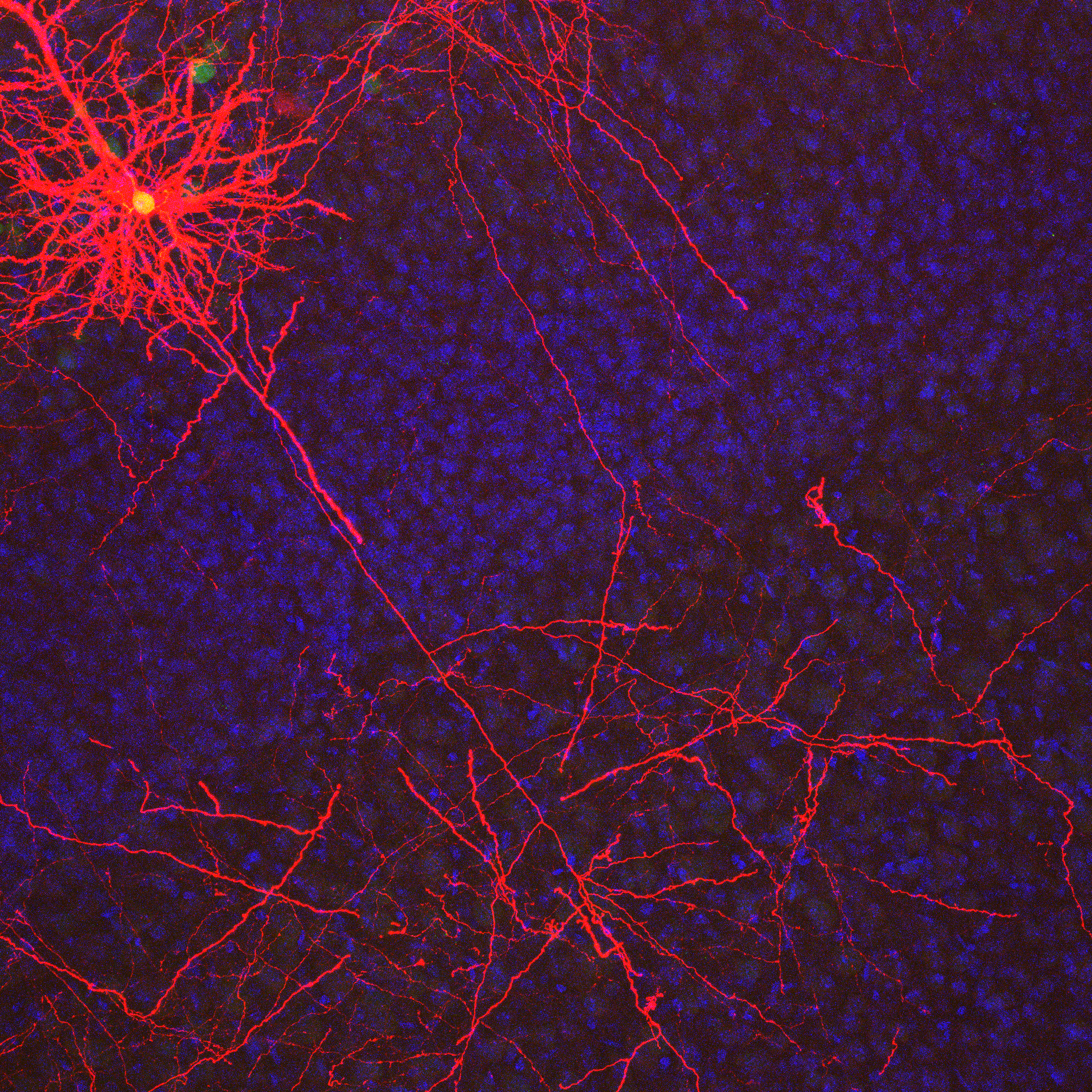

Supplement: Supplementary file 11 — EV Figures Source Data [file 44318_2024_50_MOESM11_ESM.zip › EV Figures-source files/FigureEV5-source files/FigureEV5C-control.tif]

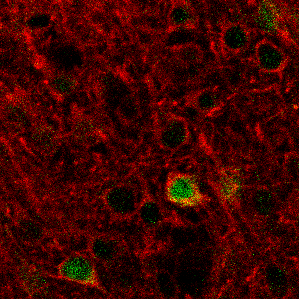

Supplement: Supplementary file 11 — EV Figures Source Data [file 44318_2024_50_MOESM11_ESM.zip › EV Figures-source files/FigureEV5-source files/FigueEV5B-SvbpMAX_exp327-P7-L-mouse2-slice1detail-1.tif]

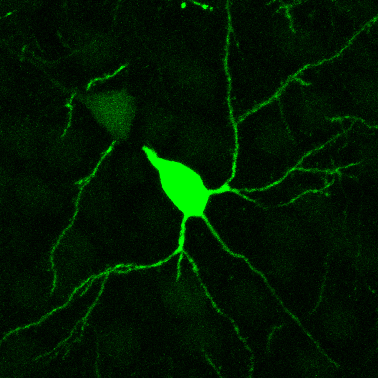

Supplement: Supplementary file 11 — EV Figures Source Data [file 44318_2024_50_MOESM11_ESM.zip › EV Figures-source files/FigureEV5-source files/FigEV5A-C1-exp285-L-example1-1.tif]

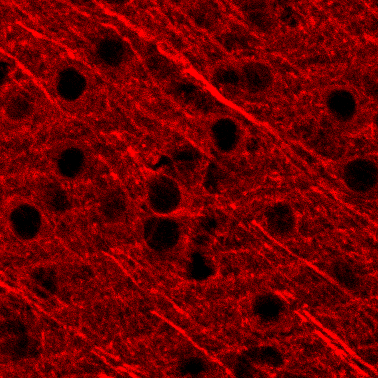

Supplement: Supplementary file 11 — EV Figures Source Data [file 44318_2024_50_MOESM11_ESM.zip › EV Figures-source files/FigureEV5-source files/FigEV5A-C2-exp285-R-example1-1.tif]

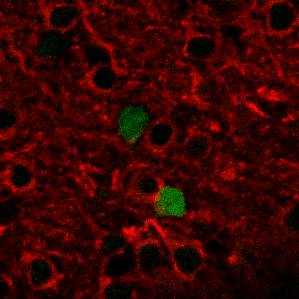

Supplement: Supplementary file 11 — EV Figures Source Data [file 44318_2024_50_MOESM11_ESM.zip › EV Figures-source files/FigureEV5-source files/FigureEV5B-TtlMAX_exp327-P7-R-mouse2-slice1detail-1.tif]

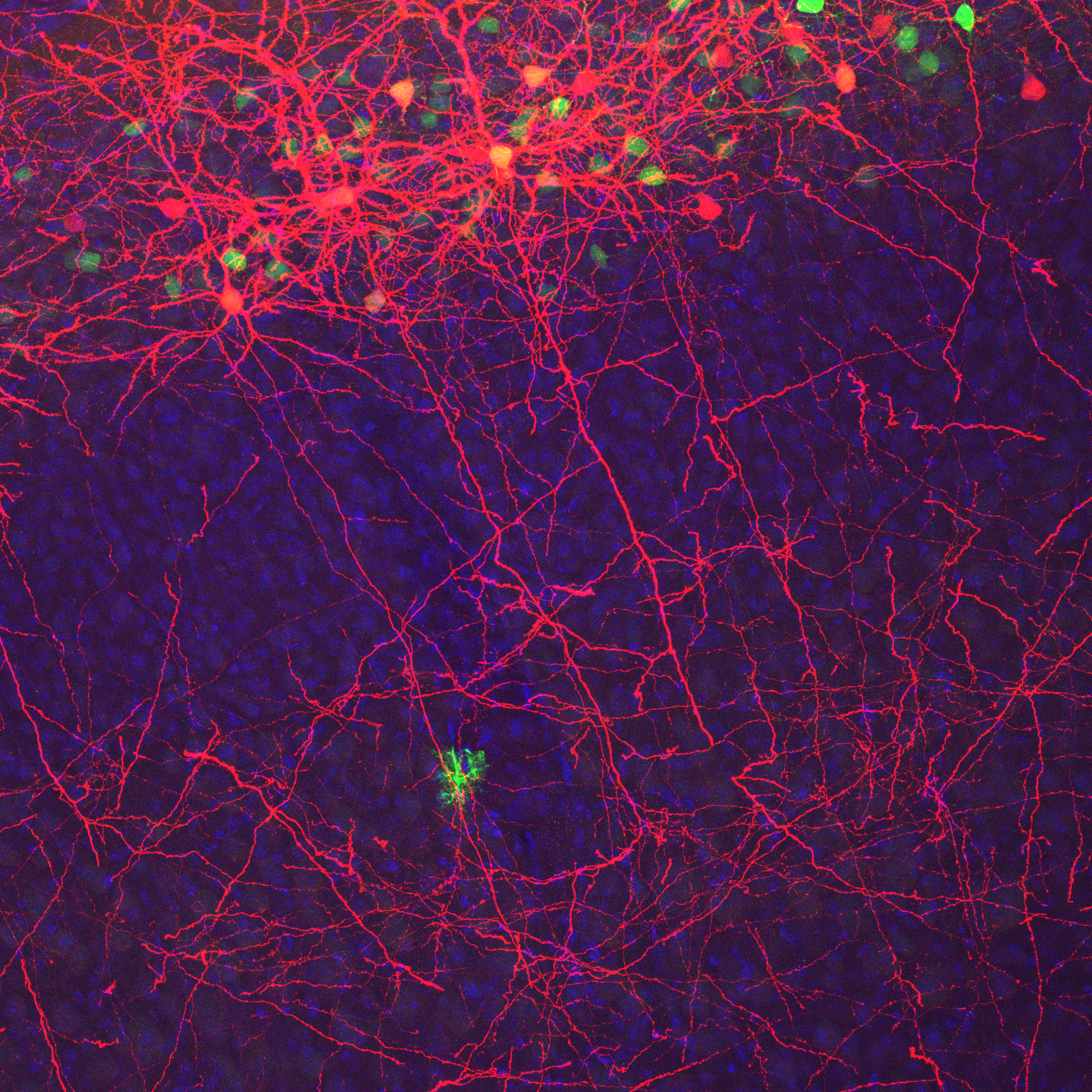

Supplement: Supplementary file 11 — EV Figures Source Data [file 44318_2024_50_MOESM11_ESM.zip › EV Figures-source files/FigureEV5-source files/FigureEV5C-doubleKO.tif]

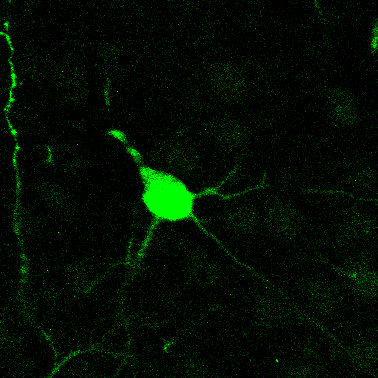

Supplement: Supplementary file 11 — EV Figures Source Data [file 44318_2024_50_MOESM11_ESM.zip › EV Figures-source files/FigureEV5-source files/FigEV5A-C1-exp285-R-example1-1.tif]

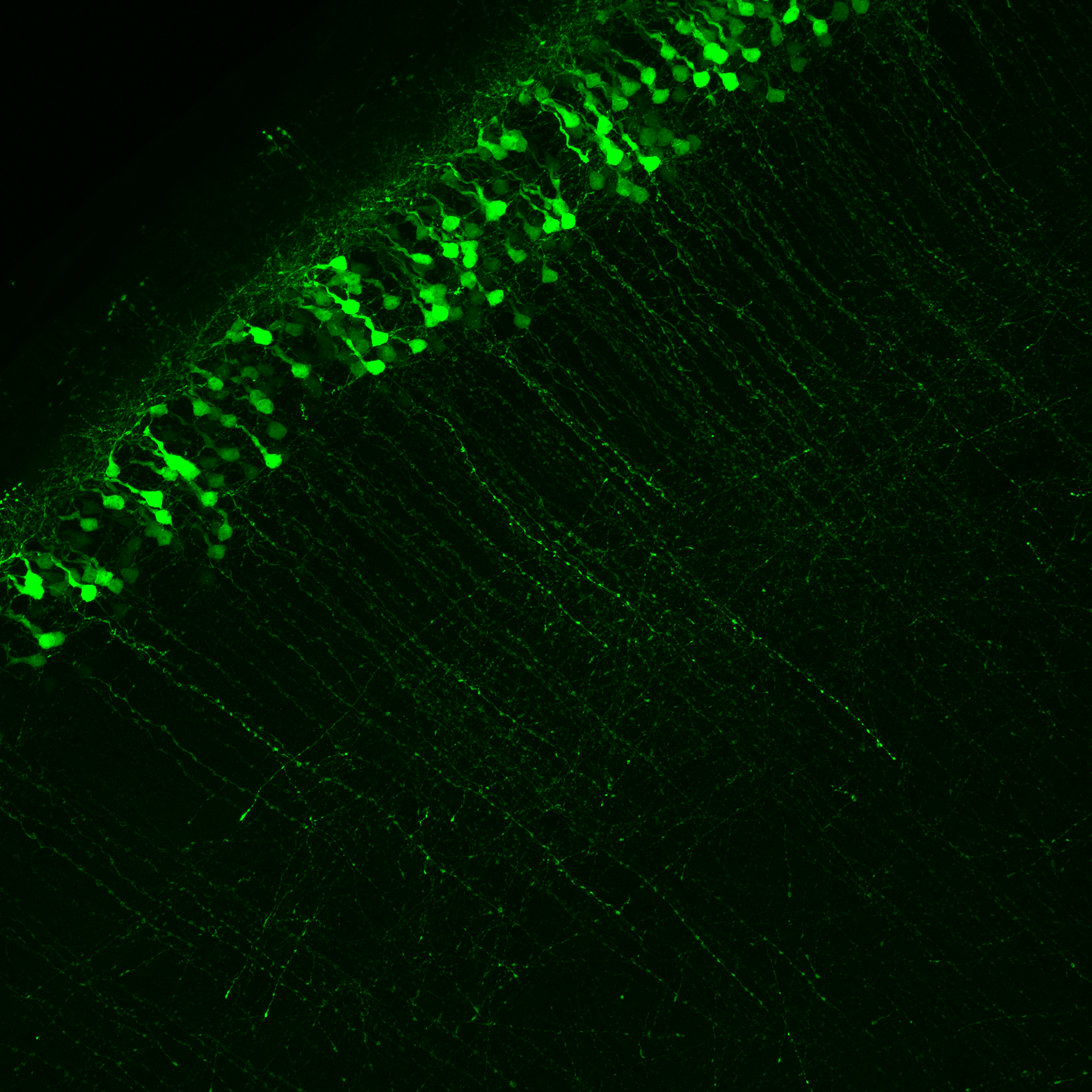

Supplement: Supplementary file 11 — EV Figures Source Data [file 44318_2024_50_MOESM11_ESM.zip › EV Figures-source files/FigureEV4-source files/FigEV4C-C2-MAX_exp322-P4-mouse1-slice3-1.tif]

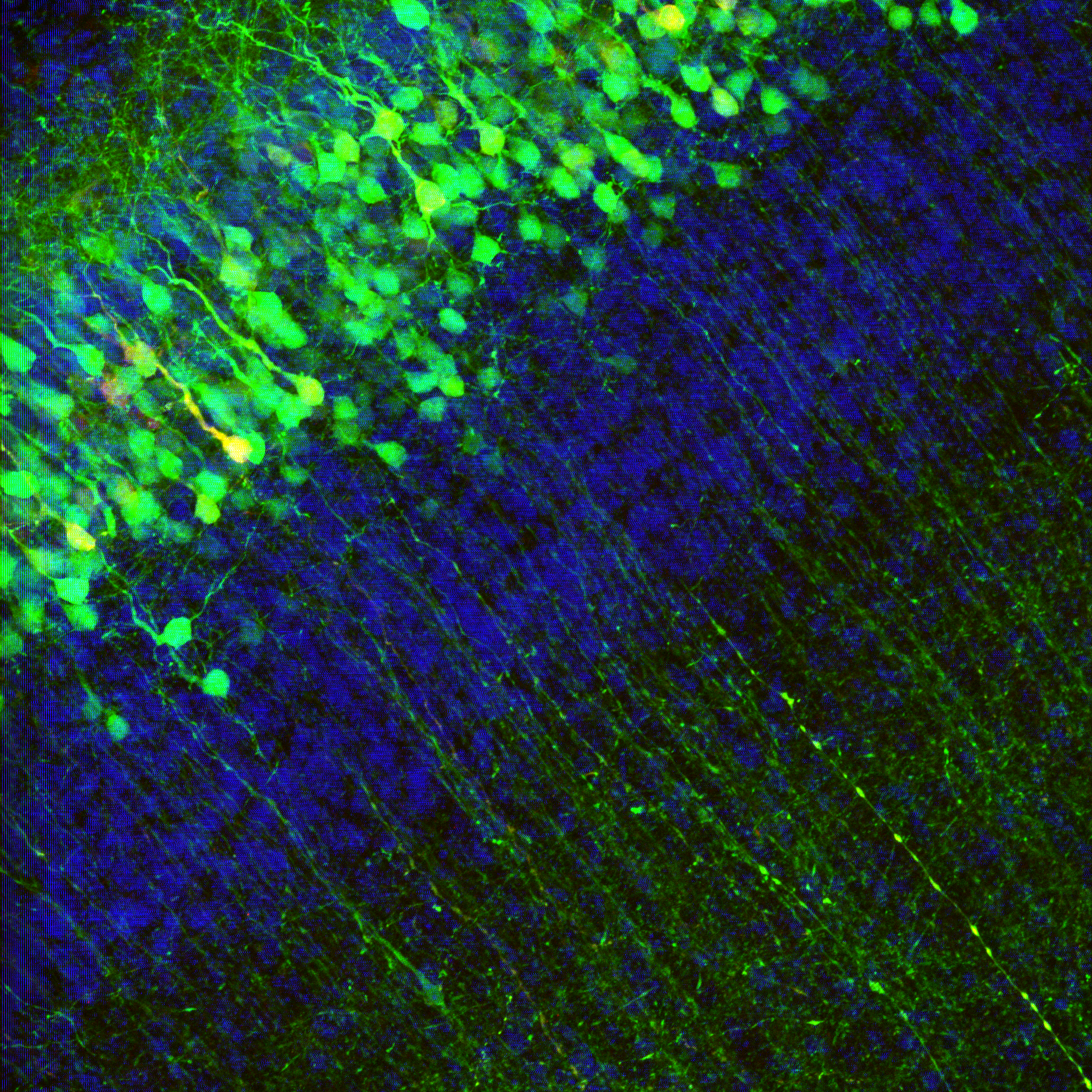

Supplement: Supplementary file 11 — EV Figures Source Data [file 44318_2024_50_MOESM11_ESM.zip › EV Figures-source files/FigureEV4-source files/FigEV4B-MAX_exp384-L-mouse1-slice5-merge.tif]

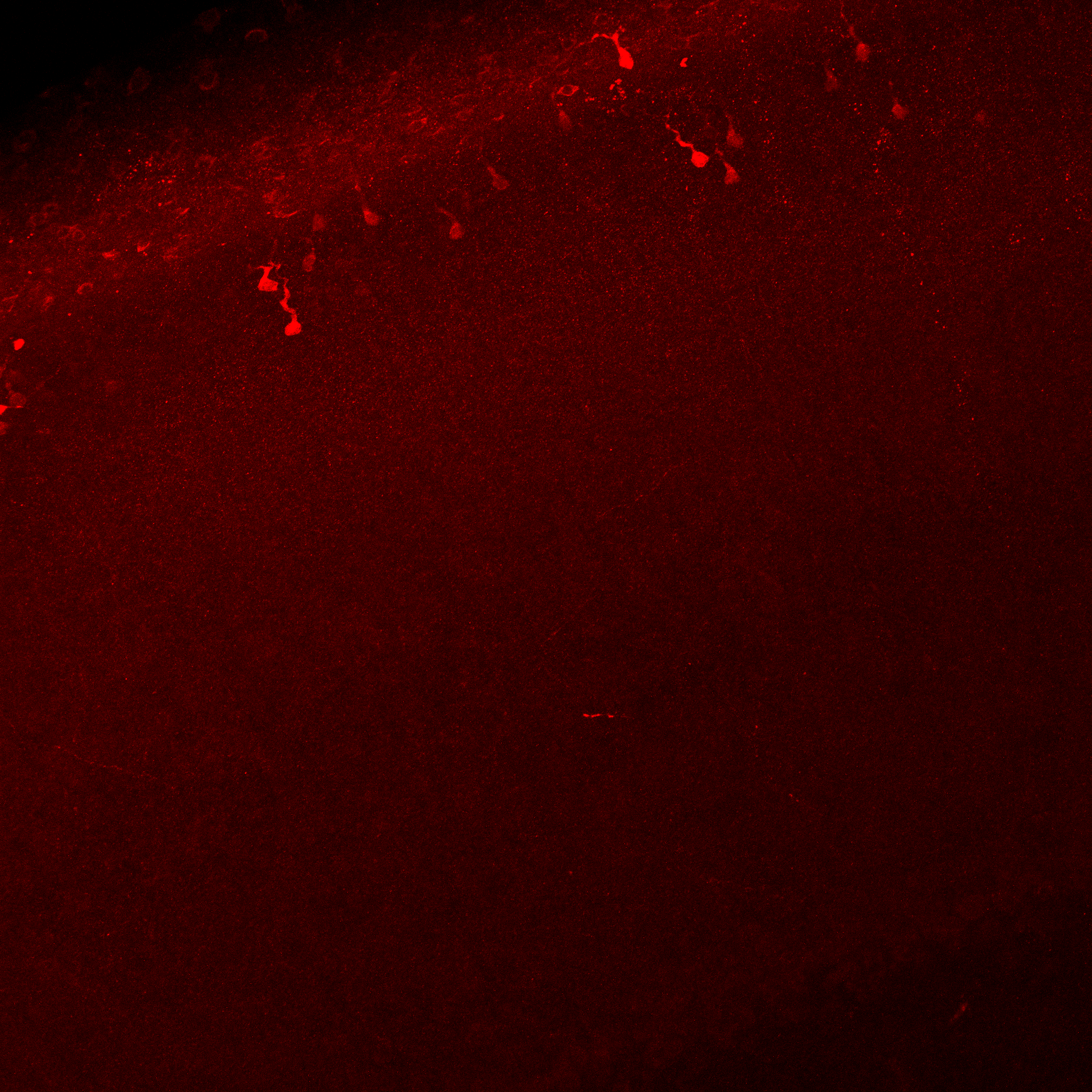

Supplement: Supplementary file 11 — EV Figures Source Data [file 44318_2024_50_MOESM11_ESM.zip › EV Figures-source files/FigureEV4-source files/FigEV4C-C1-MAX_exp322-mouse2-slice2-1.tif]

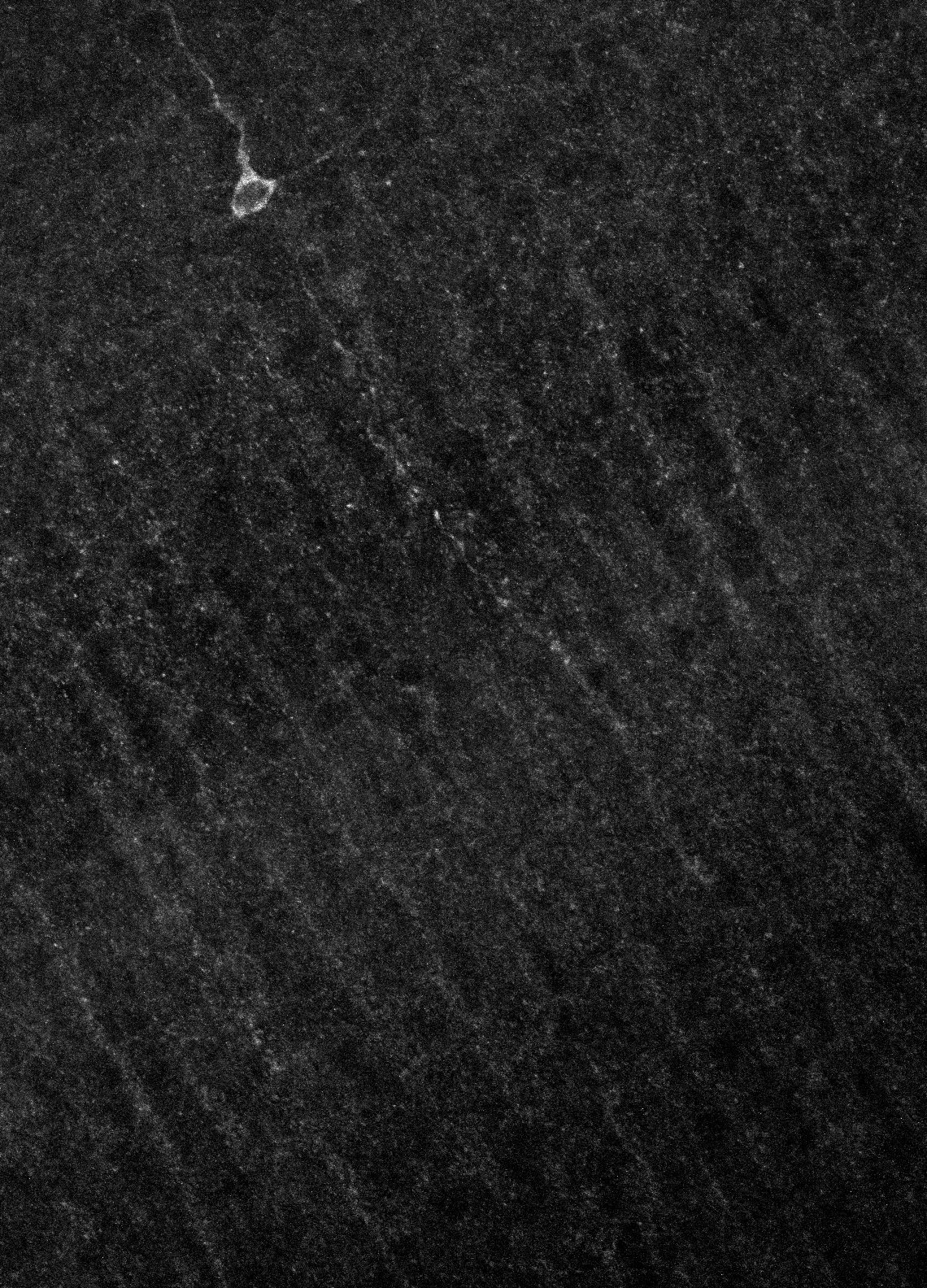

Supplement: Supplementary file 11 — EV Figures Source Data [file 44318_2024_50_MOESM11_ESM.zip › EV Figures-source files/FigureEV4-source files/FigEV4A-C1-MAX_exp389-mouse4-slice1-1.tif]

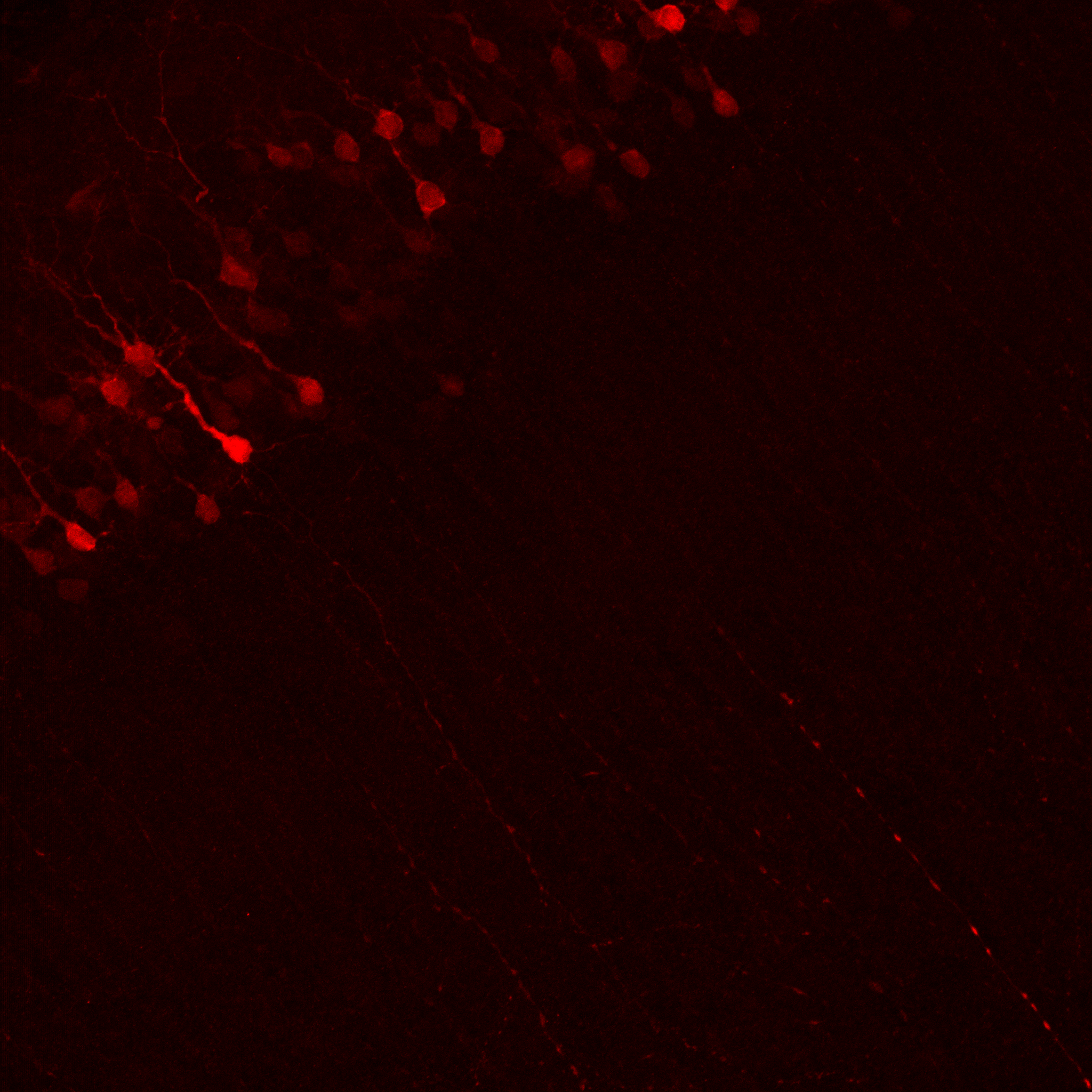

Supplement: Supplementary file 11 — EV Figures Source Data [file 44318_2024_50_MOESM11_ESM.zip › EV Figures-source files/FigureEV4-source files/FigEV4B-C3-TEST-MAX_exp384-L-mouse1-slice5-1.tif]

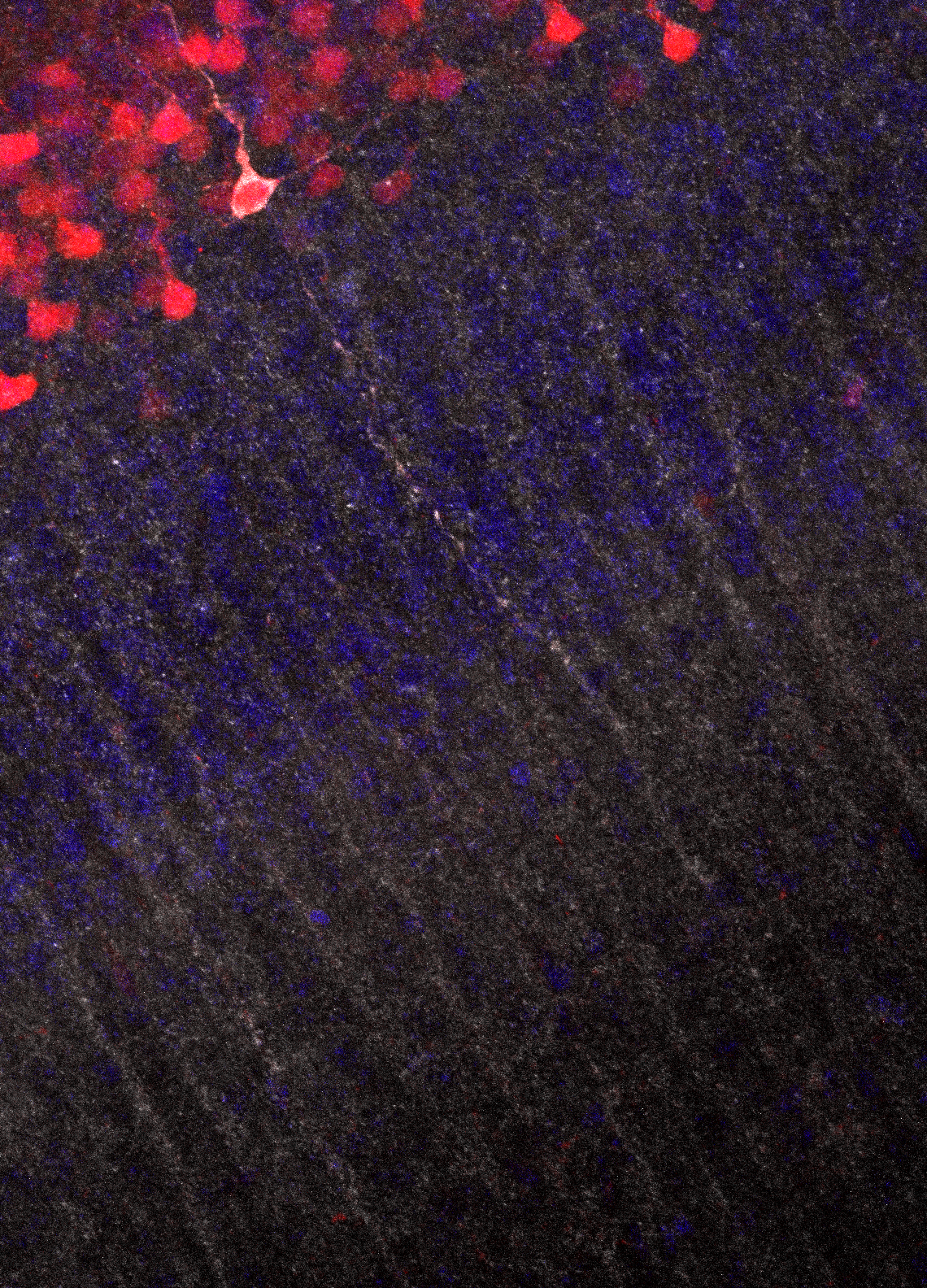

Supplement: Supplementary file 11 — EV Figures Source Data [file 44318_2024_50_MOESM11_ESM.zip › EV Figures-source files/FigureEV4-source files/FigEV4A-MAX_exp389-mouse4-slice1-merge.tif]

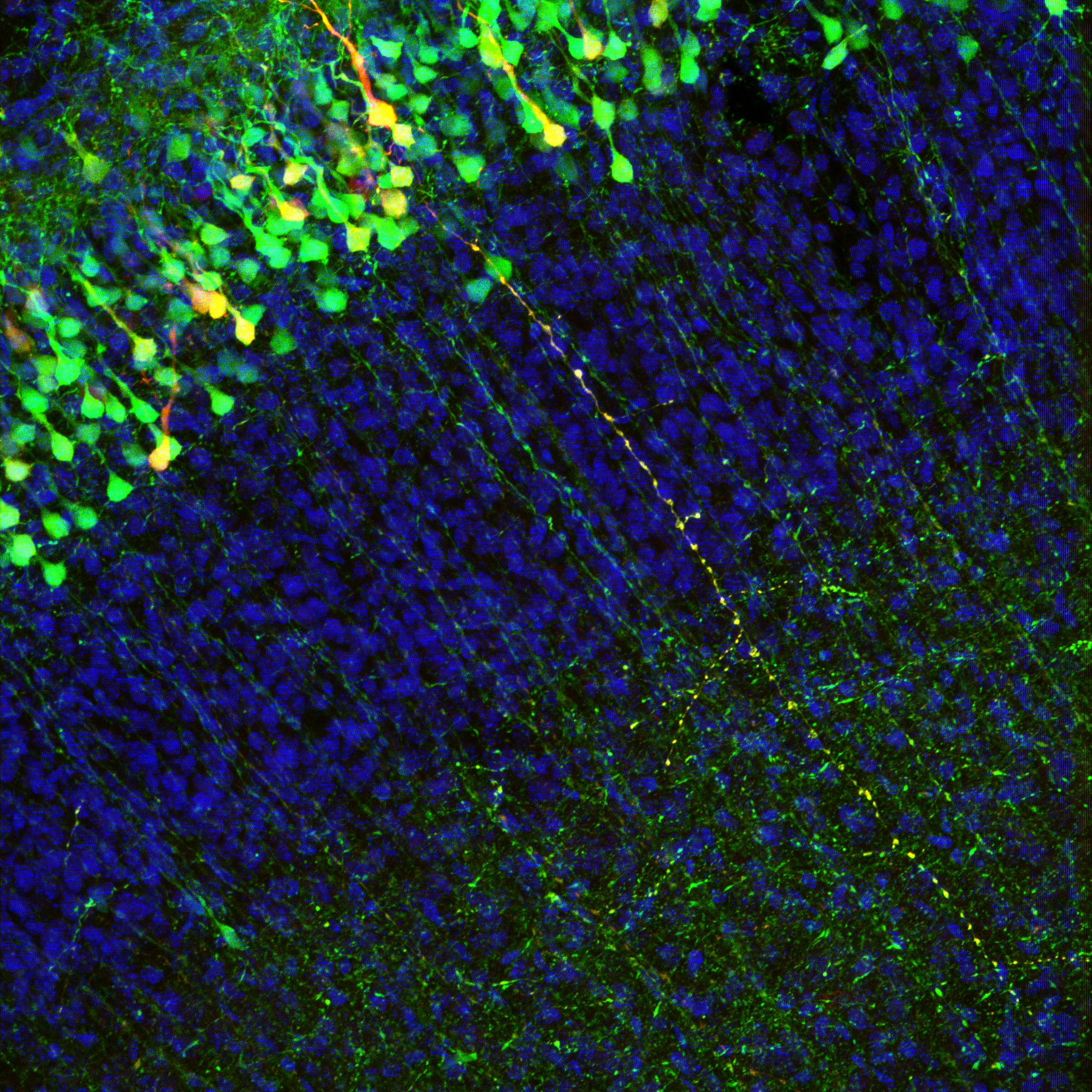

Supplement: Supplementary file 11 — EV Figures Source Data [file 44318_2024_50_MOESM11_ESM.zip › EV Figures-source files/FigureEV4-source files/FigEV4B-MAX_exp384-R-mouse2-slice1-merge.tif]

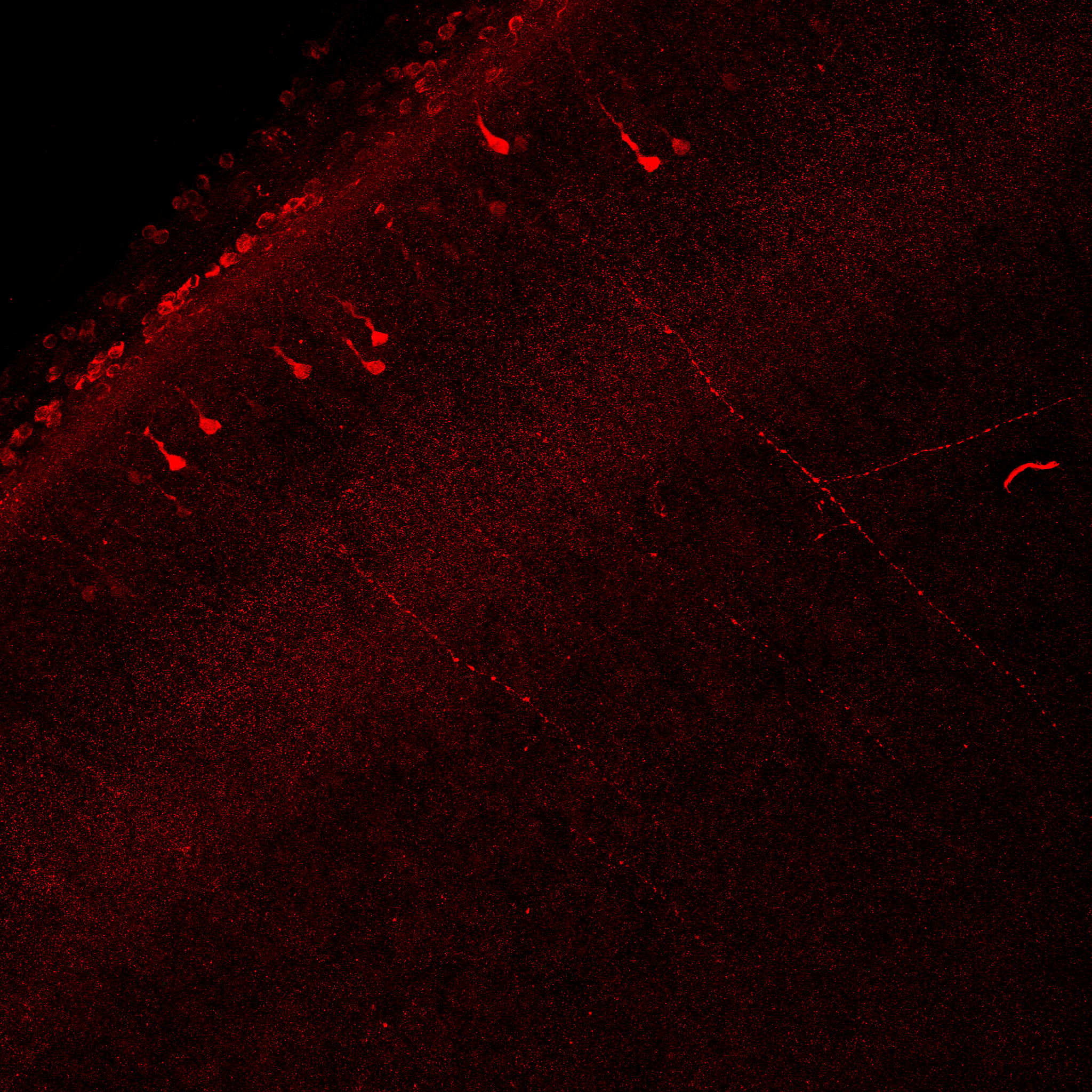

Supplement: Supplementary file 11 — EV Figures Source Data [file 44318_2024_50_MOESM11_ESM.zip › EV Figures-source files/FigureEV4-source files/FigEV4C-C1-MAX_exp322-P4-mouse1-slice3-1.tif]

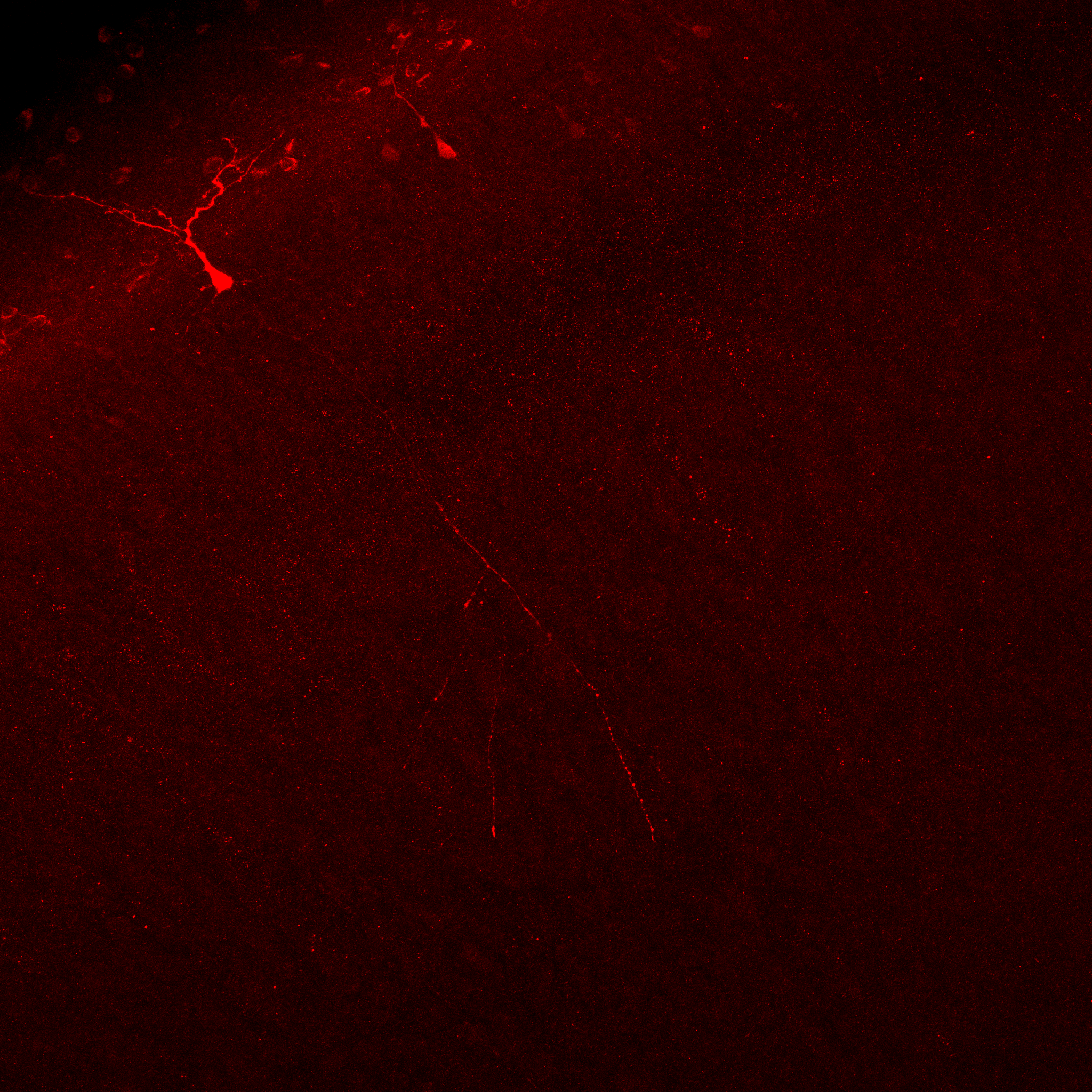

Supplement: Supplementary file 11 — EV Figures Source Data [file 44318_2024_50_MOESM11_ESM.zip › EV Figures-source files/FigureEV4-source files/FigEV4C-C1-MAX_exp322-P4-mouse2-slice5-1.tif]

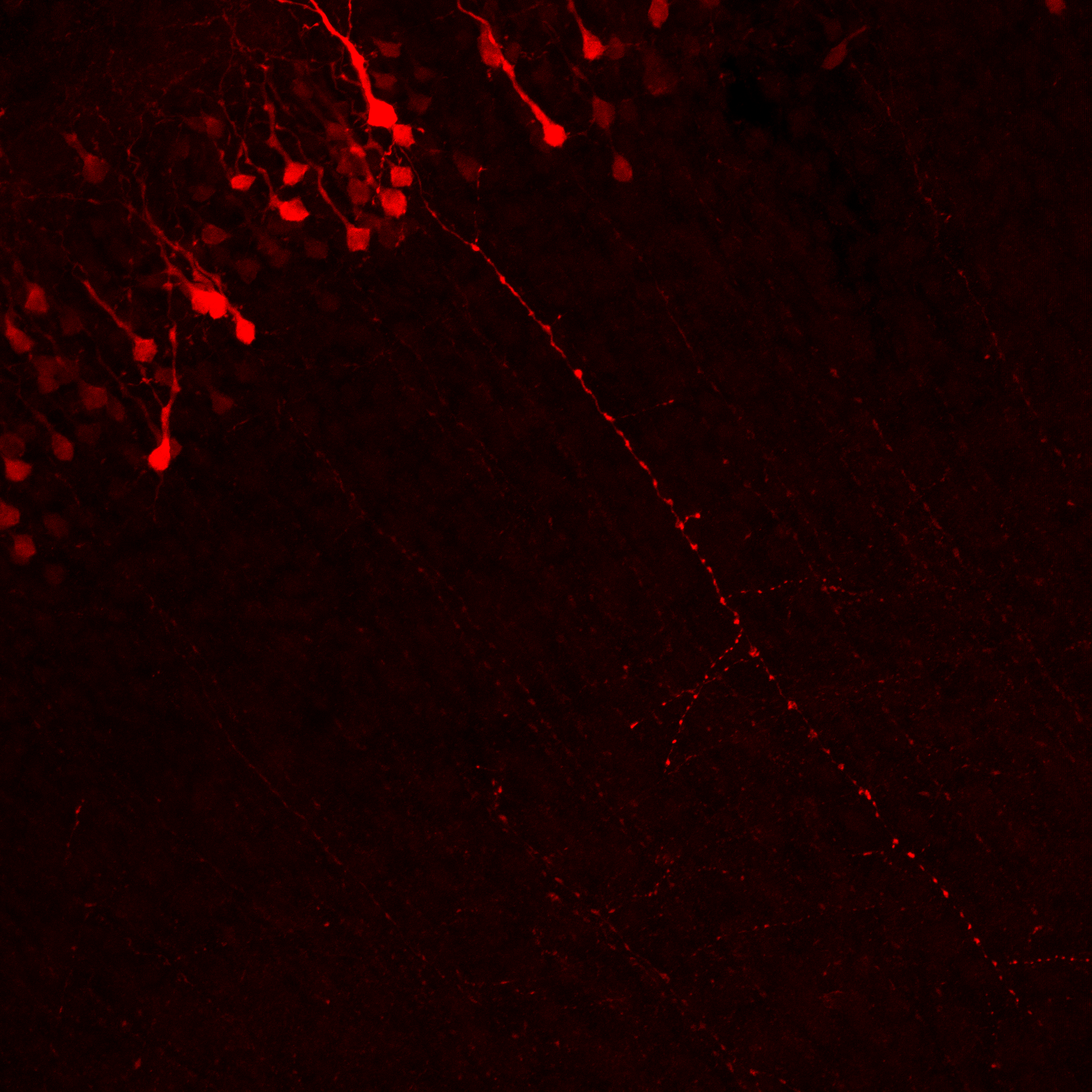

Supplement: Supplementary file 11 — EV Figures Source Data [file 44318_2024_50_MOESM11_ESM.zip › EV Figures-source files/FigureEV4-source files/FigEV4B-C3-TEST-MAX_exp384-R-mouse2-slice1-1.tif]

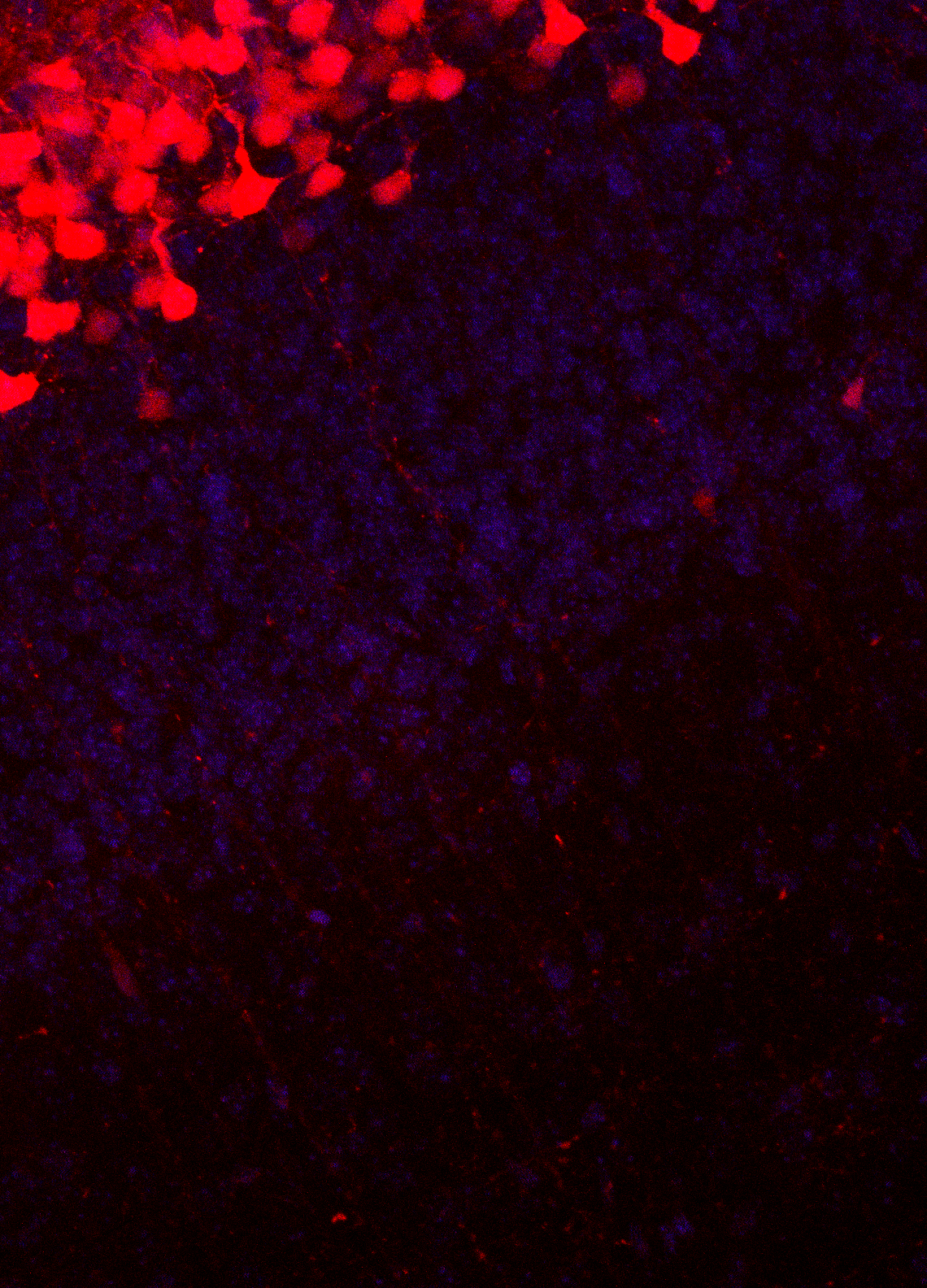

Supplement: Supplementary file 11 — EV Figures Source Data [file 44318_2024_50_MOESM11_ESM.zip › EV Figures-source files/FigureEV4-source files/FigEV4A-MAX_exp389-mouse4-slice1-1.tif]

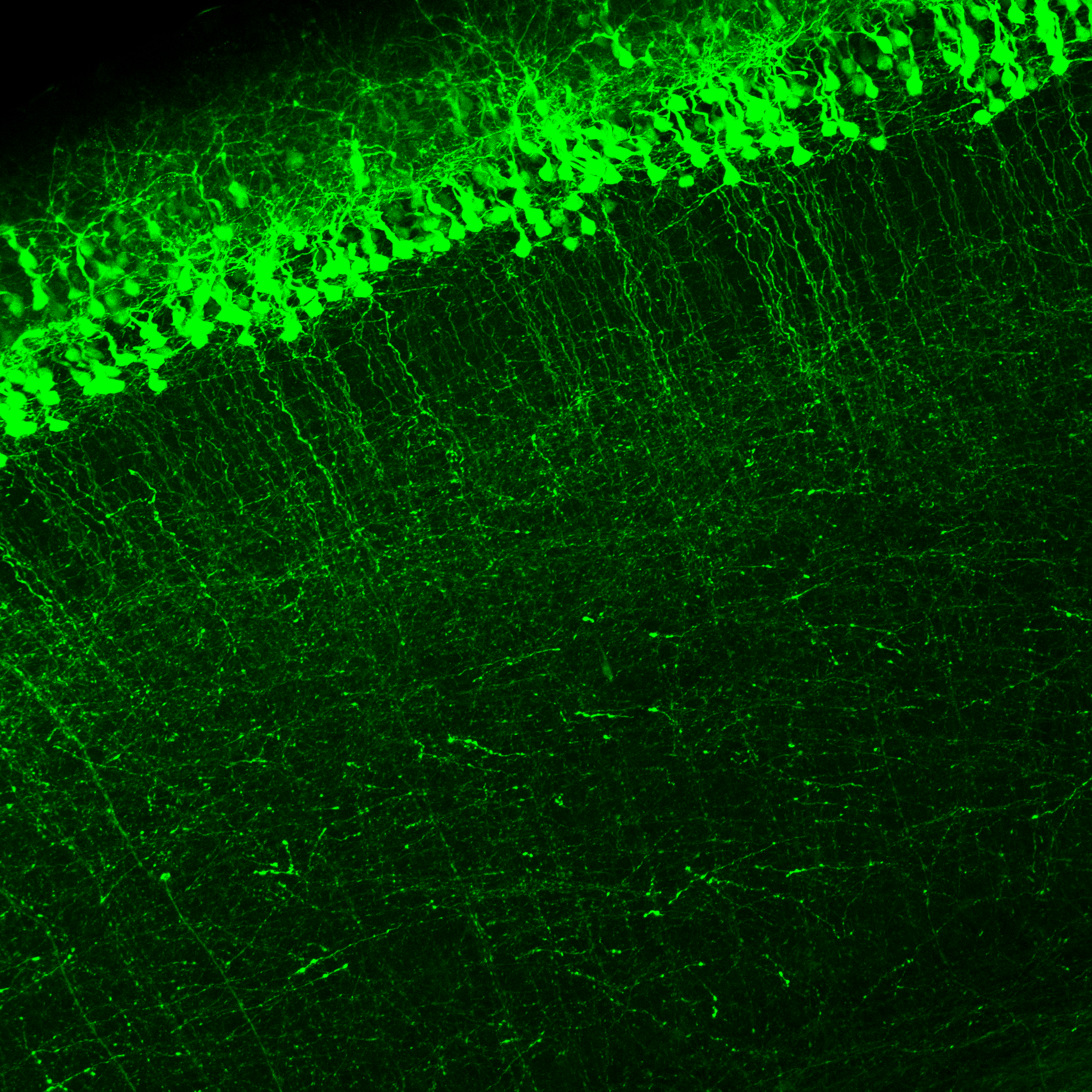

Supplement: Supplementary file 11 — EV Figures Source Data [file 44318_2024_50_MOESM11_ESM.zip › EV Figures-source files/FigureEV4-source files/FigEV4C-C2-MAX_exp322-mouse2-slice2-1.tif]

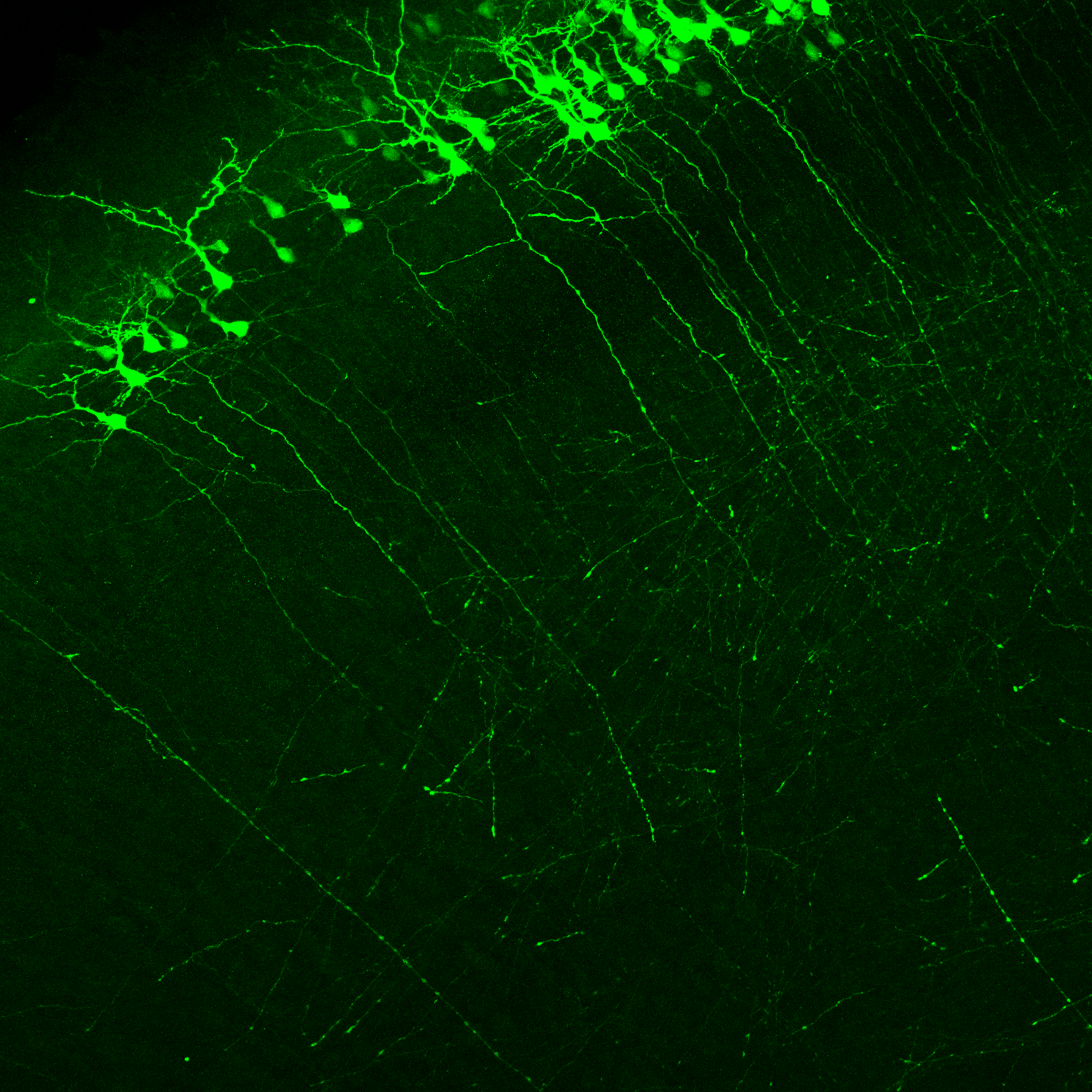

Supplement: Supplementary file 11 — EV Figures Source Data [file 44318_2024_50_MOESM11_ESM.zip › EV Figures-source files/FigureEV4-source files/FigEV4C-C2-MAX_exp322-P4-mouse2-slice5-1.tif]

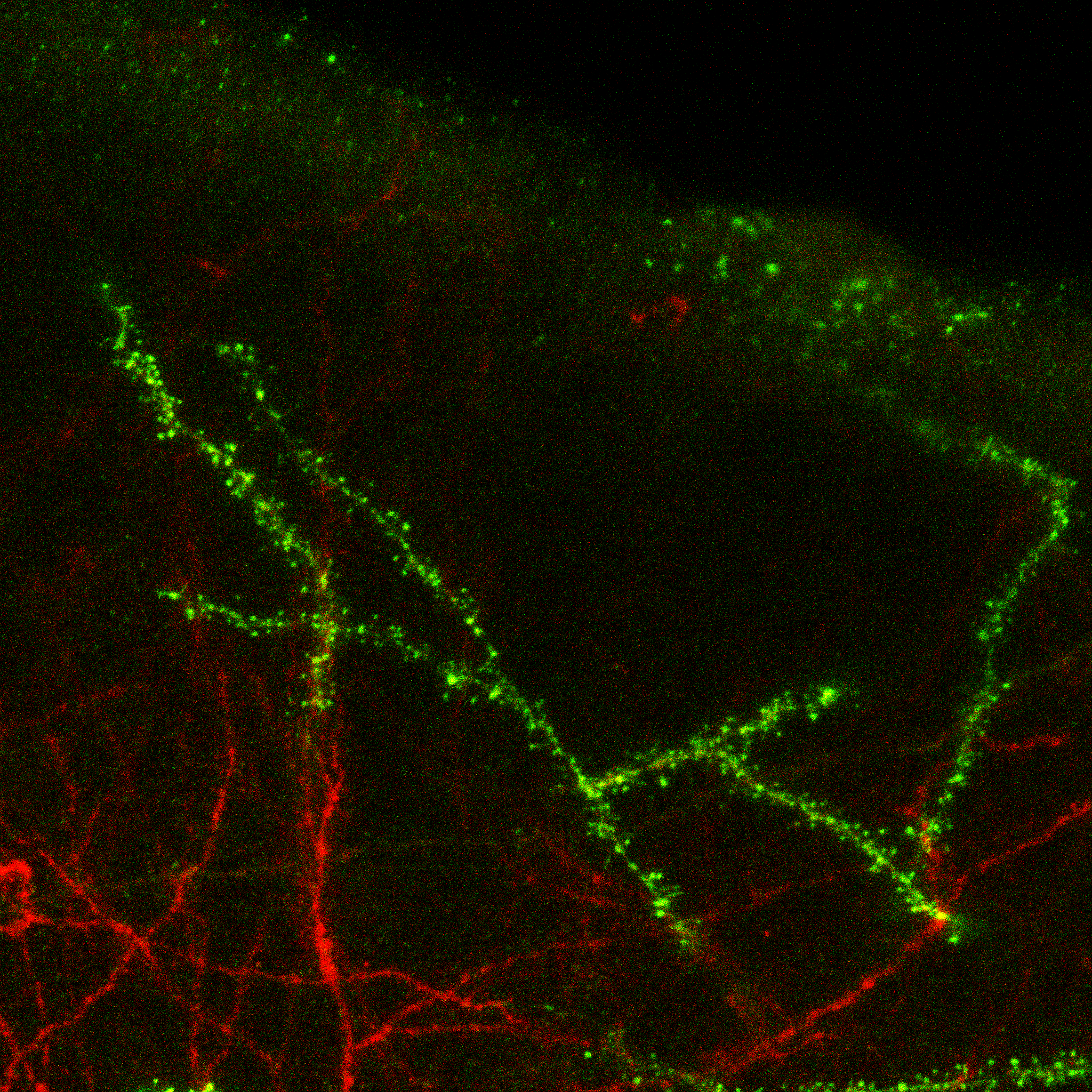

Supplement: Supplementary file 11 — EV Figures Source Data [file 44318_2024_50_MOESM11_ESM.zip › EV Figures-source files/FigureEV3-source files/FigureEV3B-63x-actin.tif]

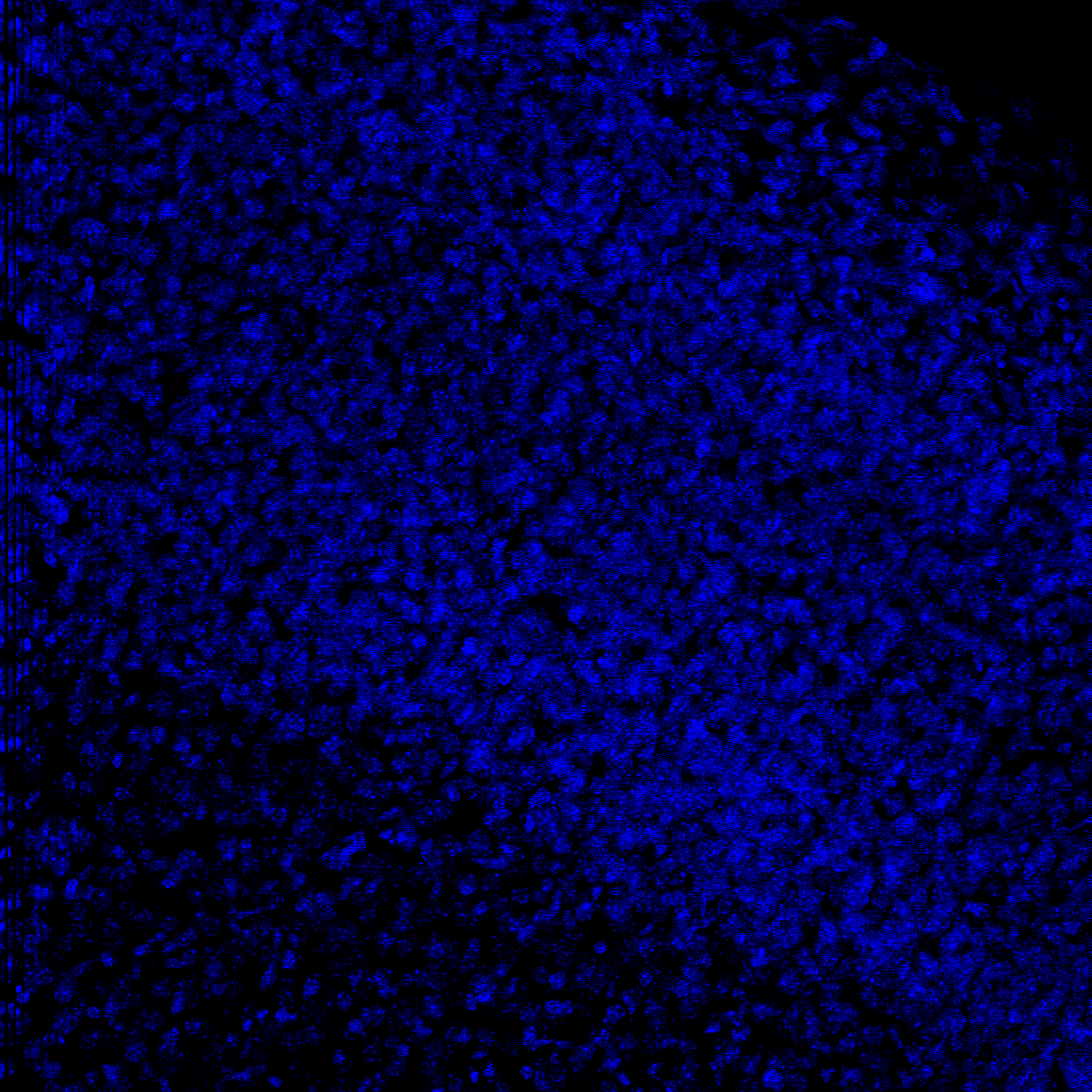

Supplement: Supplementary file 11 — EV Figures Source Data [file 44318_2024_50_MOESM11_ESM.zip › EV Figures-source files/FigureEV3-source files/FigureEV3F-DAPI-MAP1B panel.tif]

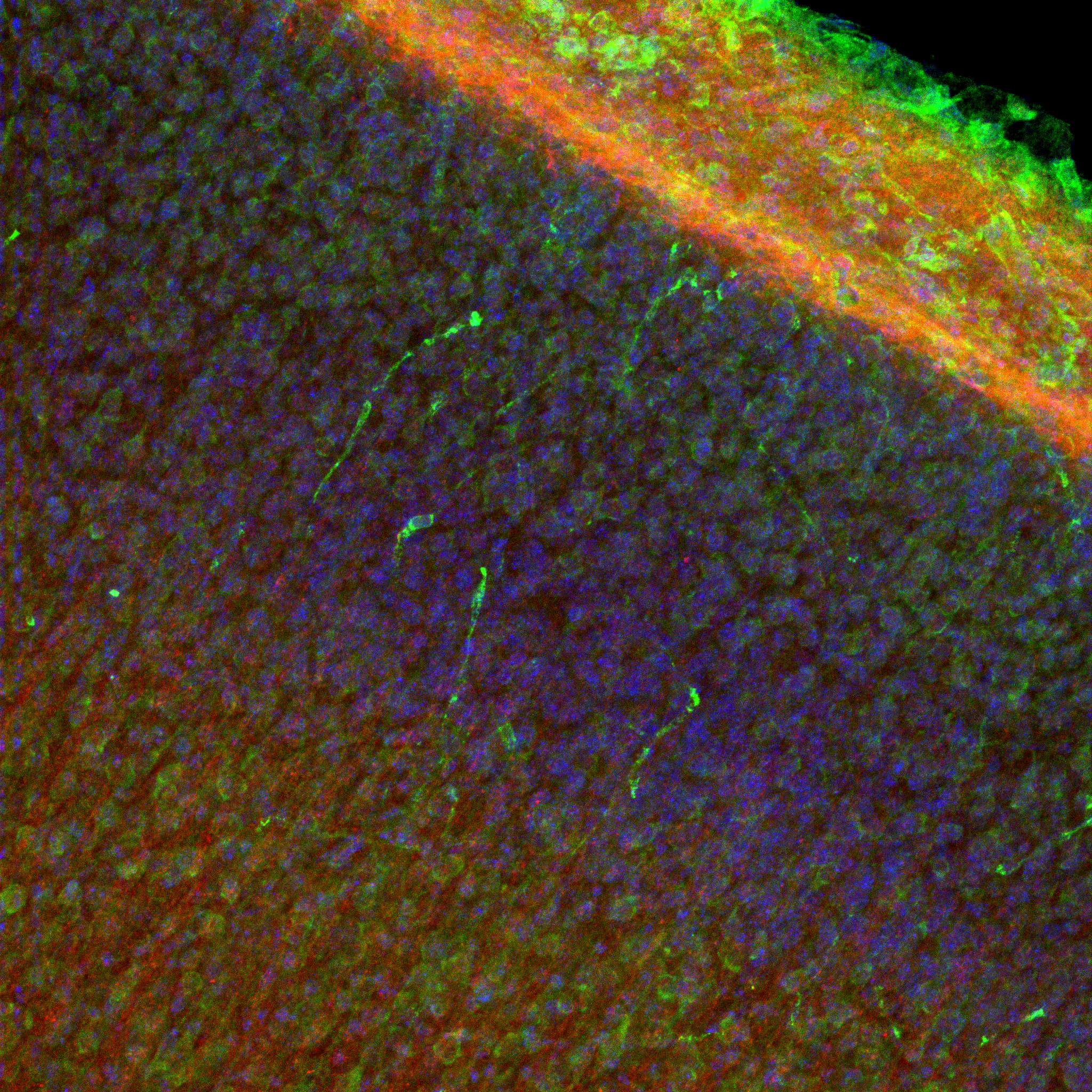

Supplement: Supplementary file 11 — EV Figures Source Data [file 44318_2024_50_MOESM11_ESM.zip › EV Figures-source files/FigureEV3-source files/FigureEV3F-MAP1B-GREEN-pMAP1B-red-merge.tif]

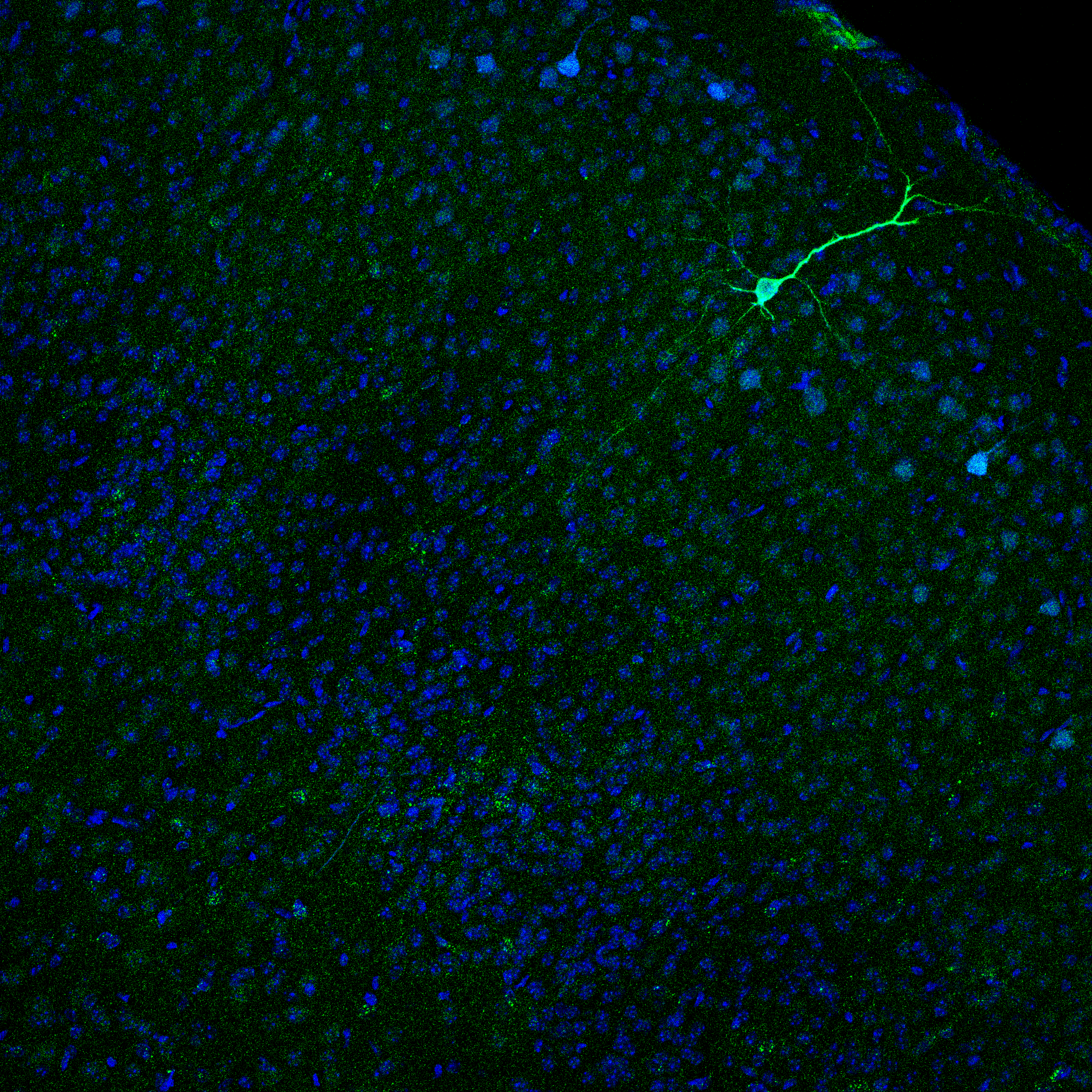

Supplement: Supplementary file 11 — EV Figures Source Data [file 44318_2024_50_MOESM11_ESM.zip › EV Figures-source files/FigureEV3-source files/FigureEV3C-MAP1B-P14.tif]

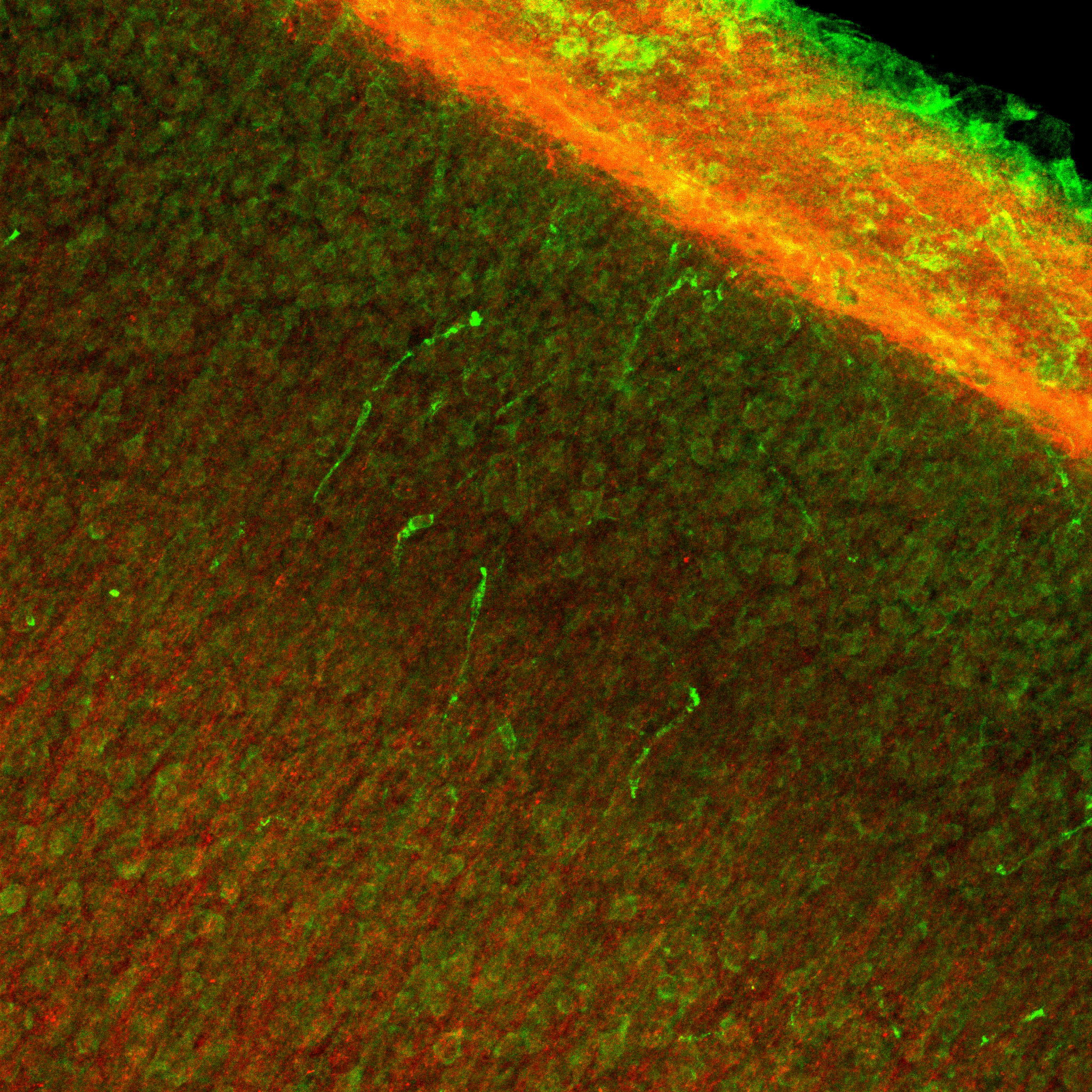

Supplement: Supplementary file 11 — EV Figures Source Data [file 44318_2024_50_MOESM11_ESM.zip › EV Figures-source files/FigureEV3-source files/FigureEV3F-MAP1B-merge-detail.tif]

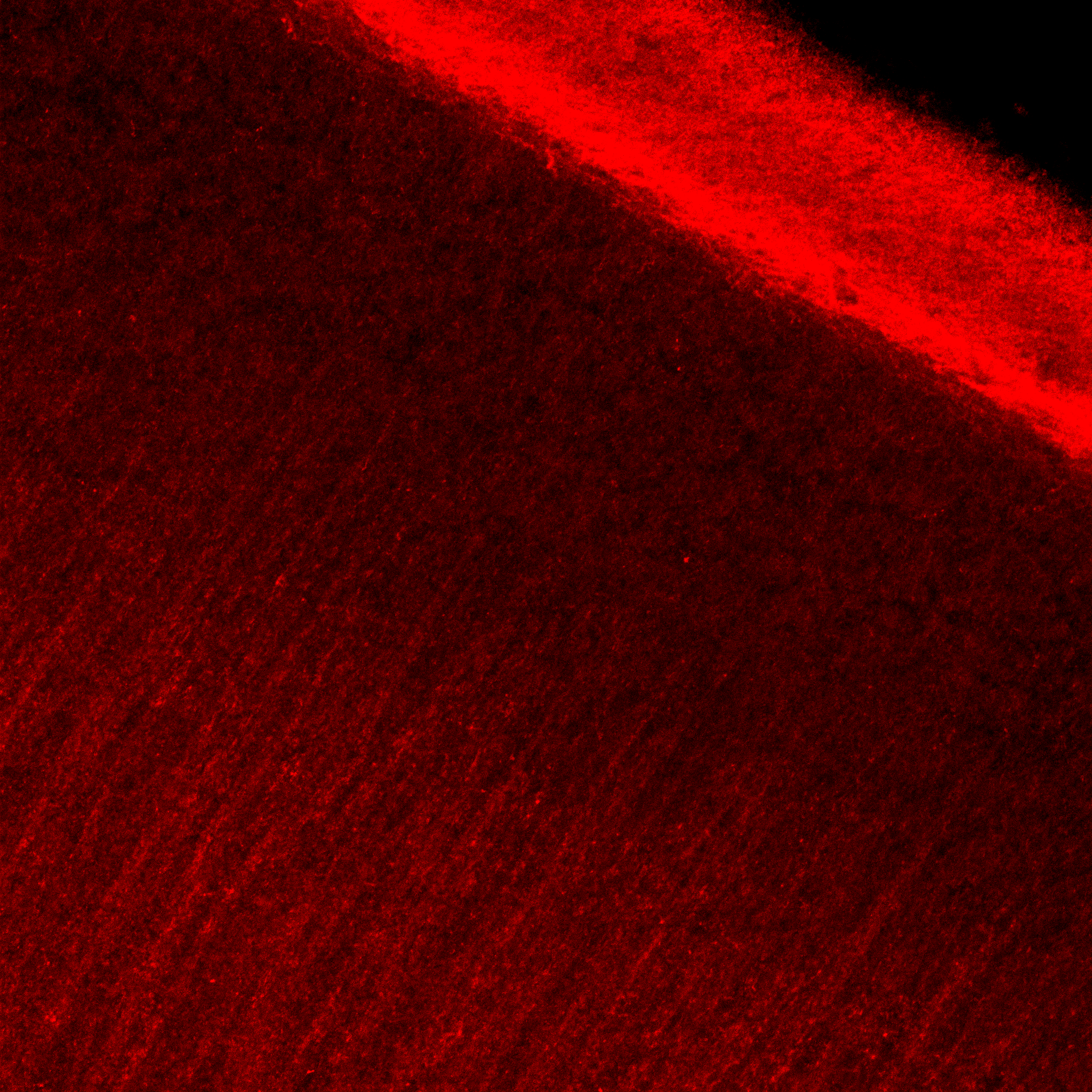

Supplement: Supplementary file 11 — EV Figures Source Data [file 44318_2024_50_MOESM11_ESM.zip › EV Figures-source files/FigureEV3-source files/FigureEV3F-phosphoMAP1B-Map1b panel.tif]

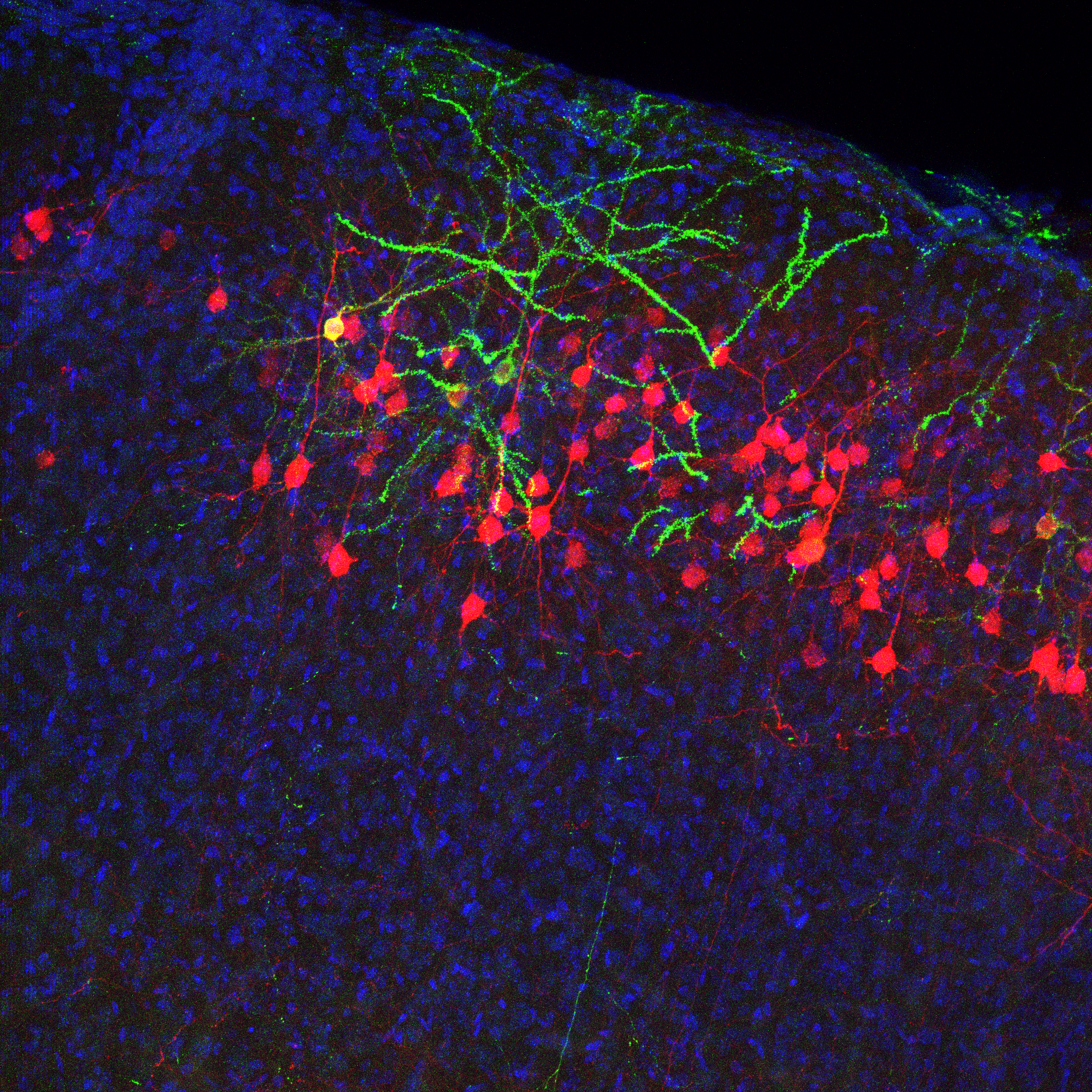

Supplement: Supplementary file 11 — EV Figures Source Data [file 44318_2024_50_MOESM11_ESM.zip › EV Figures-source files/FigureEV3-source files/FigureEV3B-actin-1.tif]

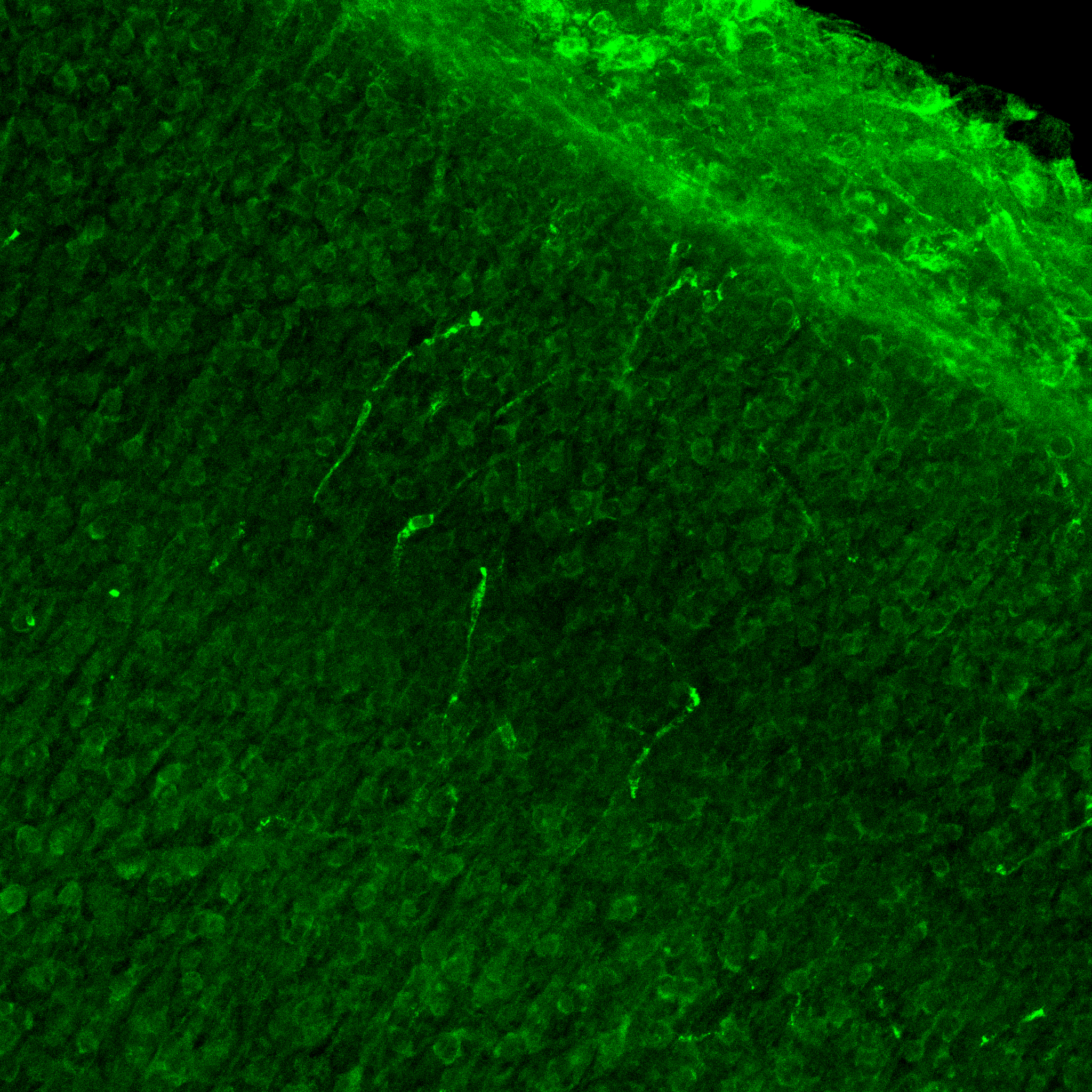

Supplement: Supplementary file 11 — EV Figures Source Data [file 44318_2024_50_MOESM11_ESM.zip › EV Figures-source files/FigureEV3-source files/FigureEV3F-MAP1B-Map1B panel.tif]

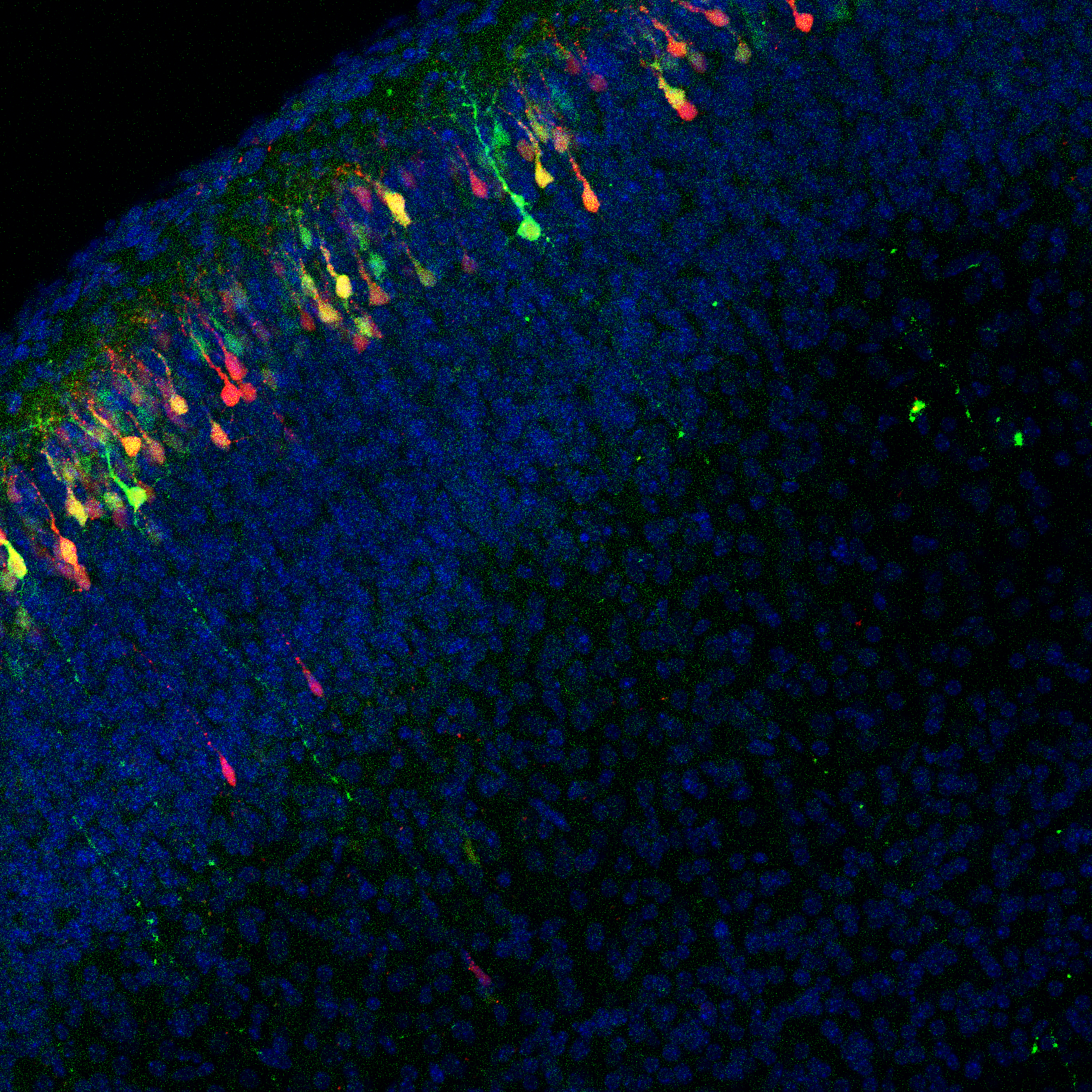

Supplement: Supplementary file 11 — EV Figures Source Data [file 44318_2024_50_MOESM11_ESM.zip › EV Figures-source files/FigureEV3-source files/FigureEV3D-GSK3B.tif]

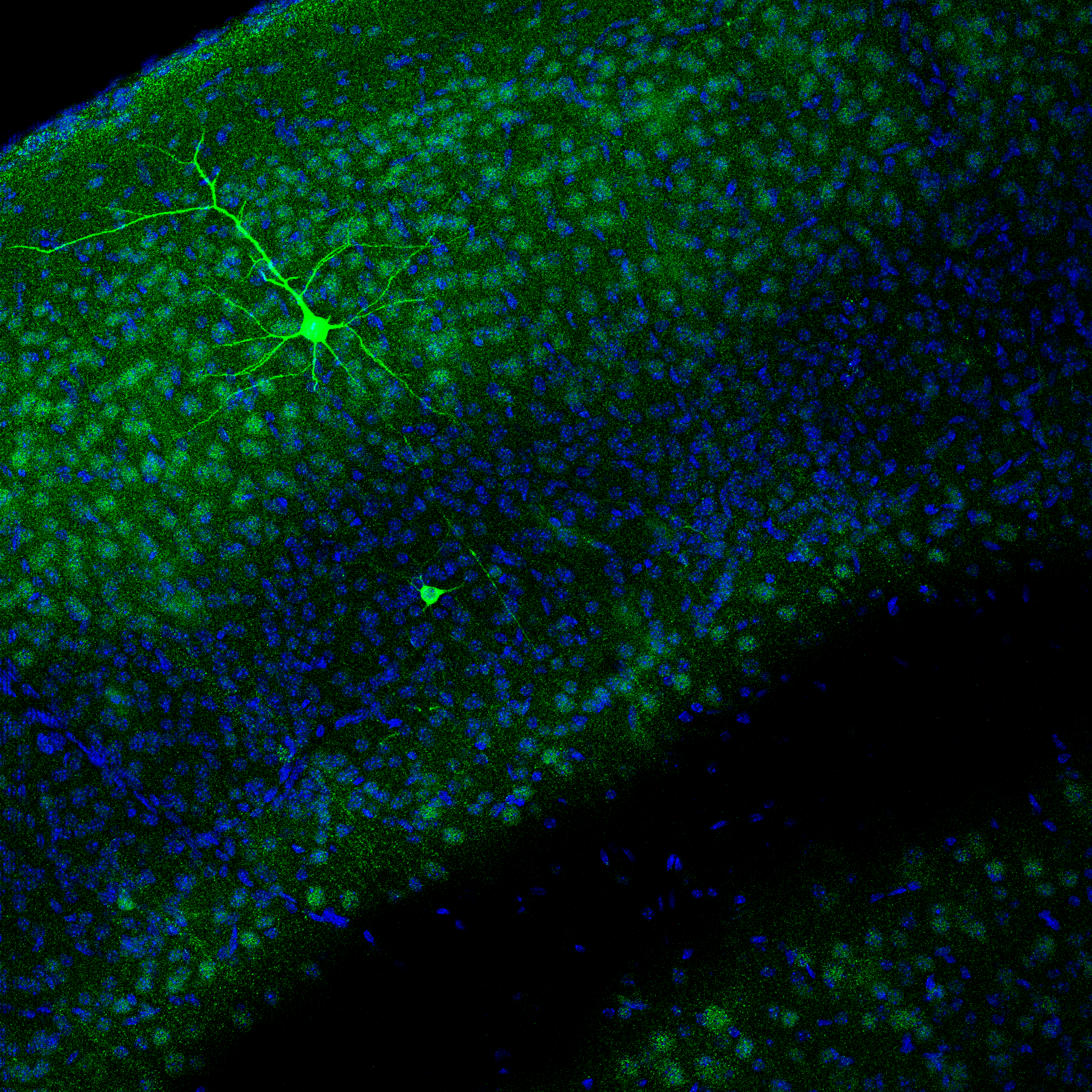

Supplement: Supplementary file 11 — EV Figures Source Data [file 44318_2024_50_MOESM11_ESM.zip › EV Figures-source files/FigureEV3-source files/FigureEV3D-GSK3B-P14.tif]

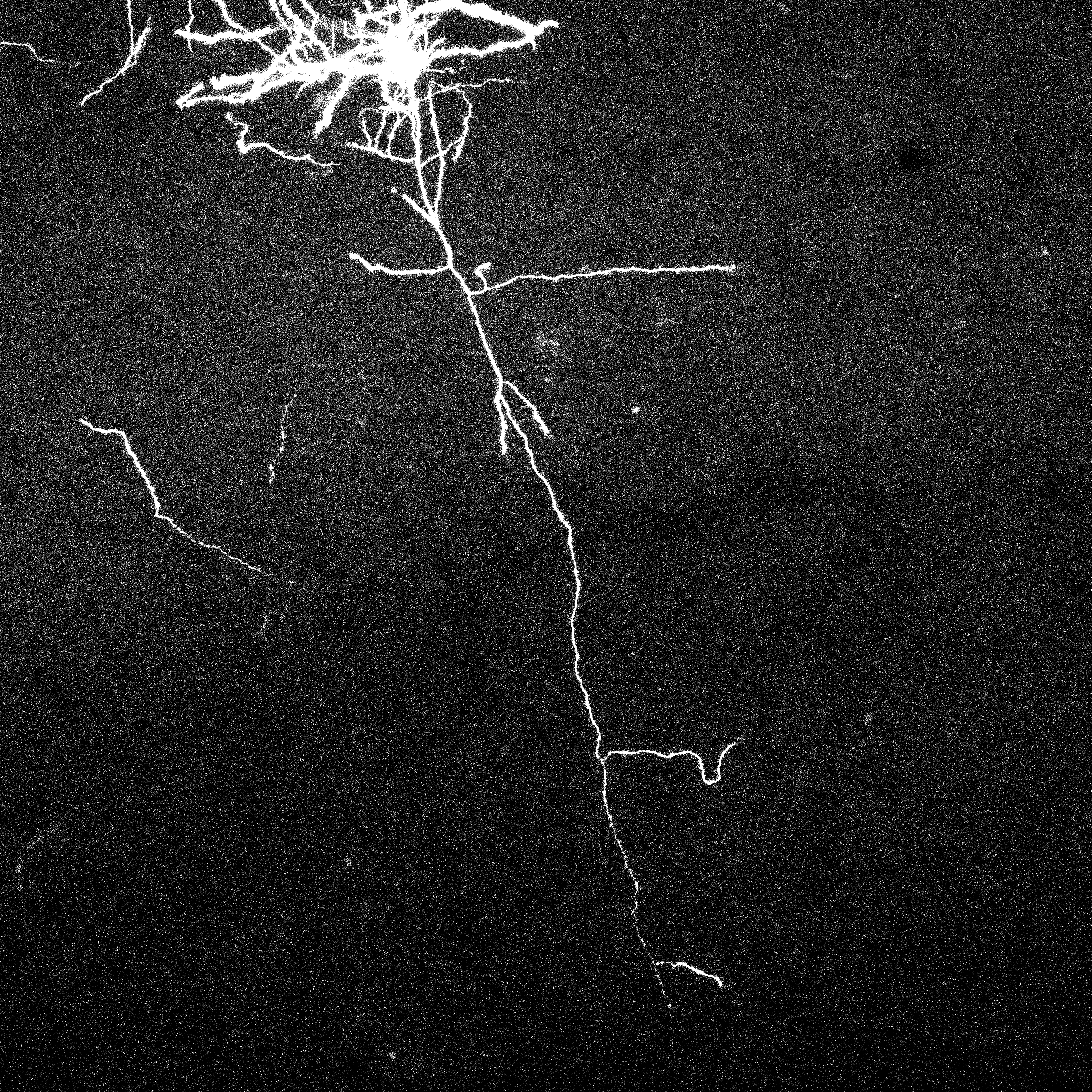

Supplement: Supplementary file 11 — EV Figures Source Data [file 44318_2024_50_MOESM11_ESM.zip › EV Figures-source files/FigureEV1-source files/FigureEV1E.tif]

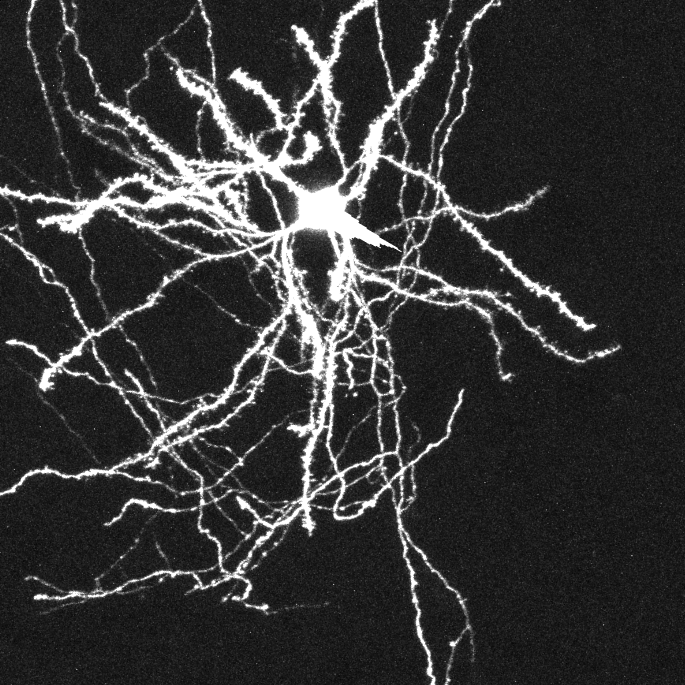

Supplement: Supplementary file 11 — EV Figures Source Data [file 44318_2024_50_MOESM11_ESM.zip › EV Figures-source files/FigureEV1-source files/MAX_exp1-mouse2-1-dendrite-doubleKO-1.tif]

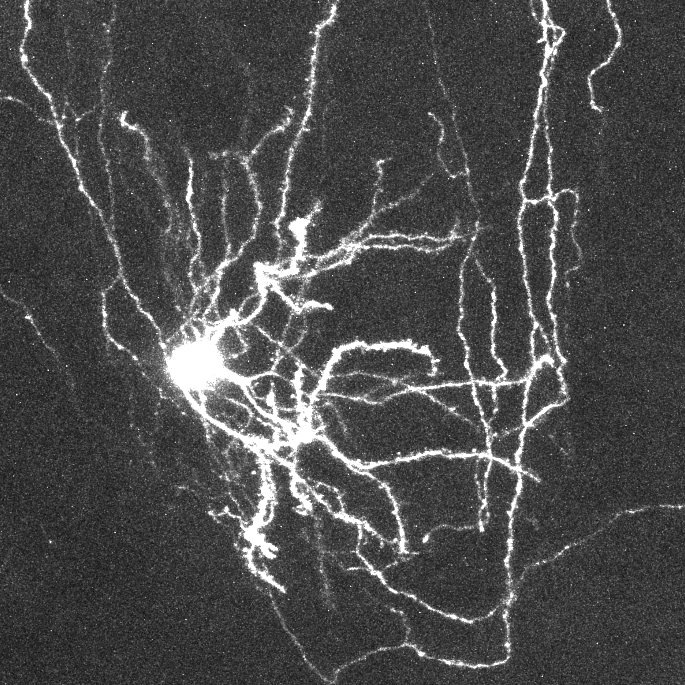

Supplement: Supplementary file 11 — EV Figures Source Data [file 44318_2024_50_MOESM11_ESM.zip › EV Figures-source files/FigureEV1-source files/MAX_exp1-mouse2-2-dendrite-doubleKO-1.tif]

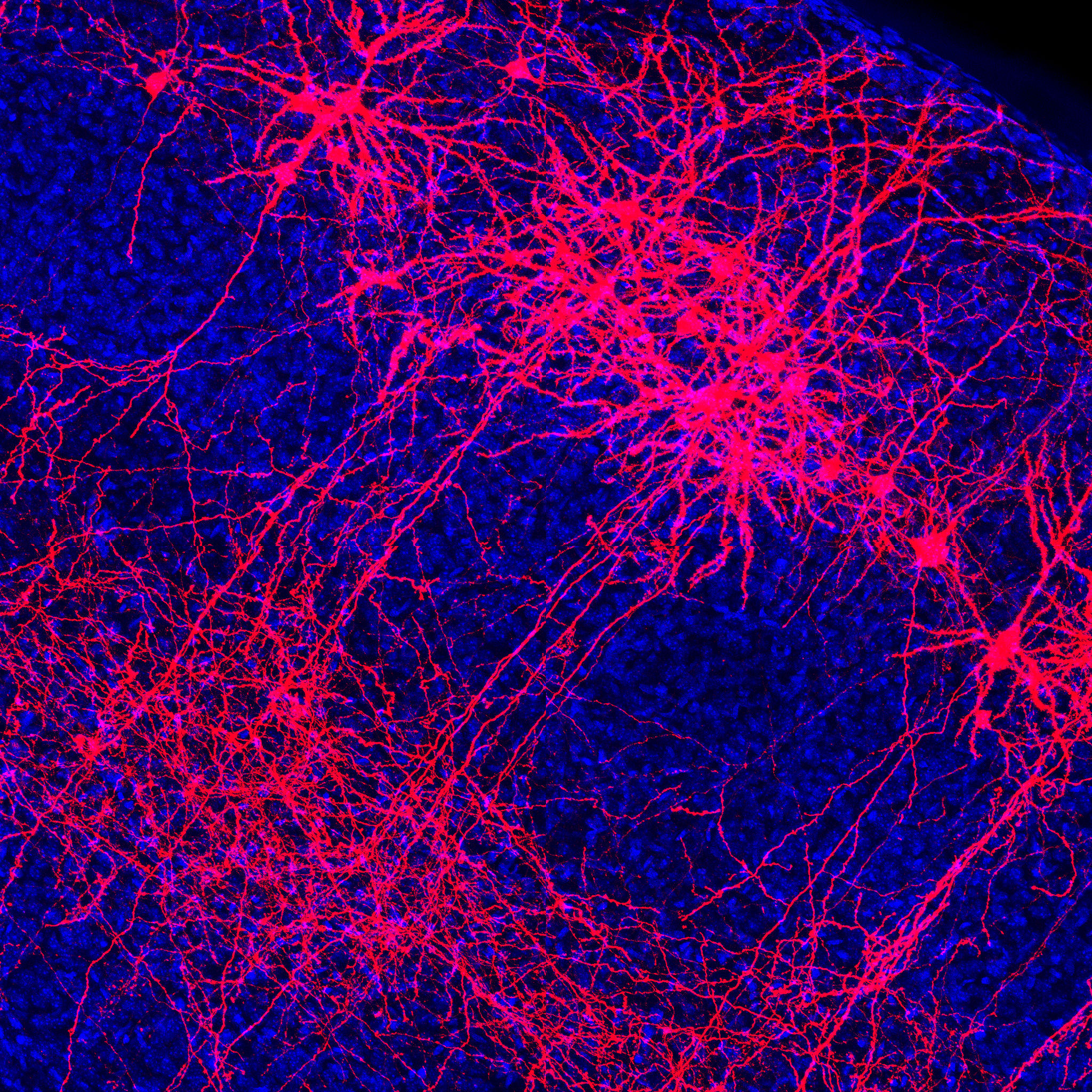

Supplement: Supplementary file 11 — EV Figures Source Data [file 44318_2024_50_MOESM11_ESM.zip › EV Figures-source files/FigureEV1-source files/FigureEV1B-GSK3BCA.tif]

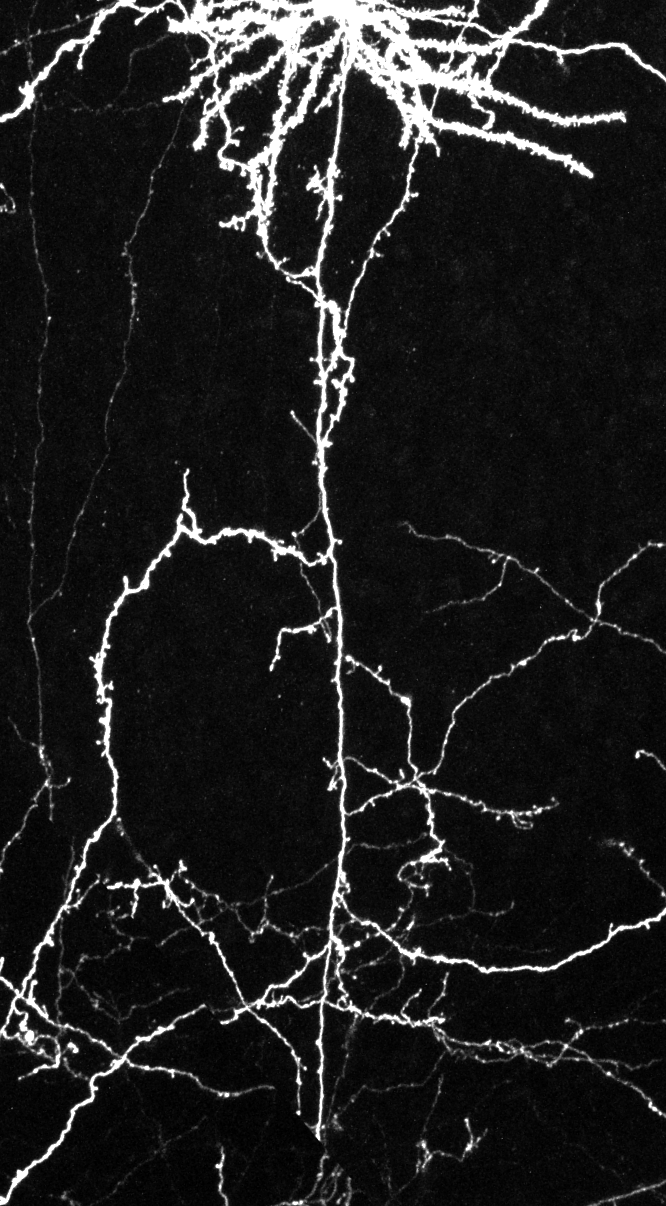

Supplement: Supplementary file 11 — EV Figures Source Data [file 44318_2024_50_MOESM11_ESM.zip › EV Figures-source files/FigureEV1-source files/FigureEV1C-protrusions.tif]

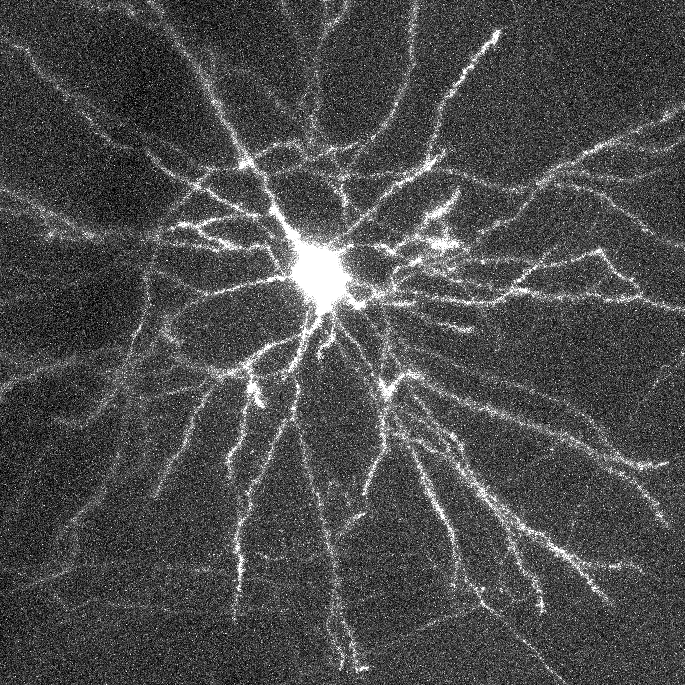

Supplement: Supplementary file 11 — EV Figures Source Data [file 44318_2024_50_MOESM11_ESM.zip › EV Figures-source files/FigureEV1-source files/C2-MAX_exp9-mouse2-slice1-gsk3bKO-1.tif]

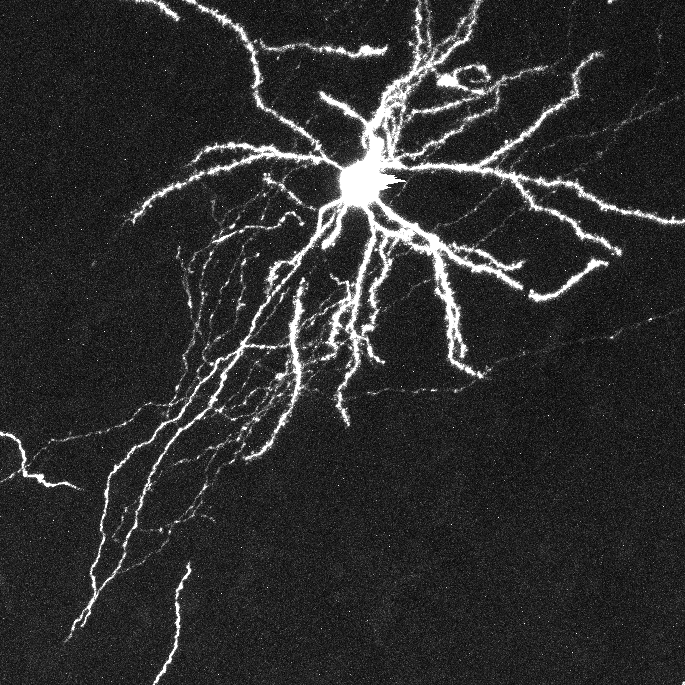

Supplement: Supplementary file 11 — EV Figures Source Data [file 44318_2024_50_MOESM11_ESM.zip › EV Figures-source files/FigureEV1-source files/C2-MAX_exp9-mouse5-slice5-doubleKO-1.tif]

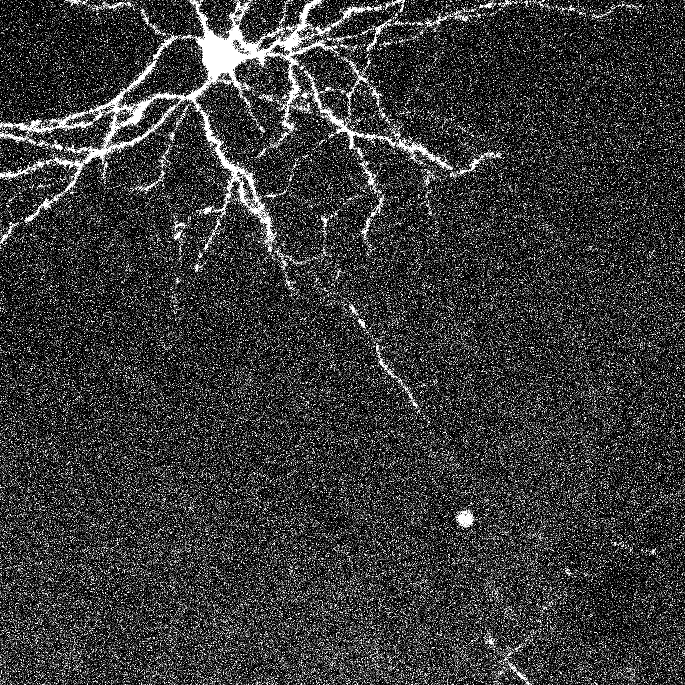

Supplement: Supplementary file 11 — EV Figures Source Data [file 44318_2024_50_MOESM11_ESM.zip › EV Figures-source files/FigureEV1-source files/C2-MAX_exp9-mouse1-slice4-gsk3bKO-1.tif]

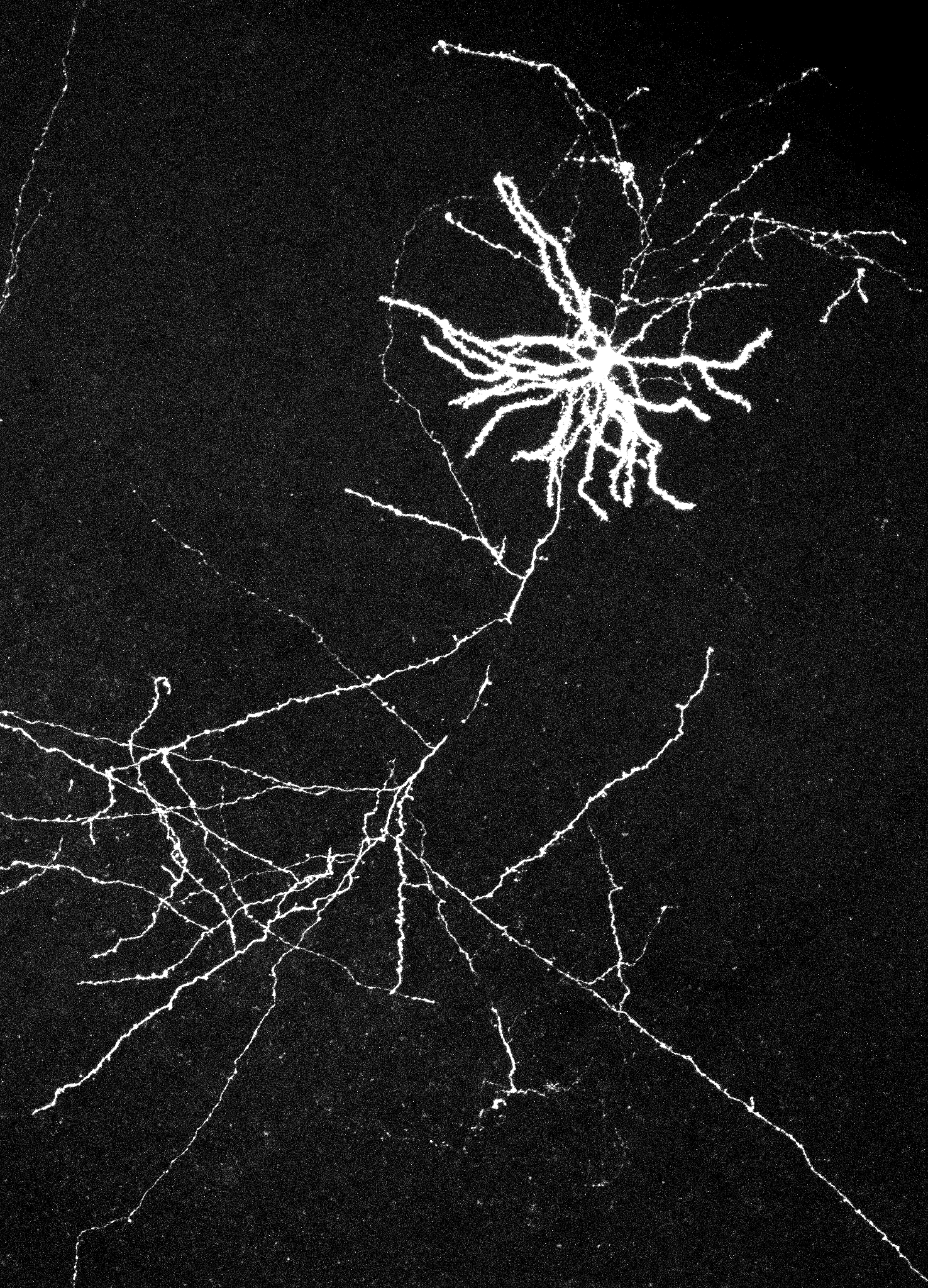

Supplement: Supplementary file 11 — EV Figures Source Data [file 44318_2024_50_MOESM11_ESM.zip › EV Figures-source files/FigureEV1-source files/FigureEV1D-adult-single.tif]

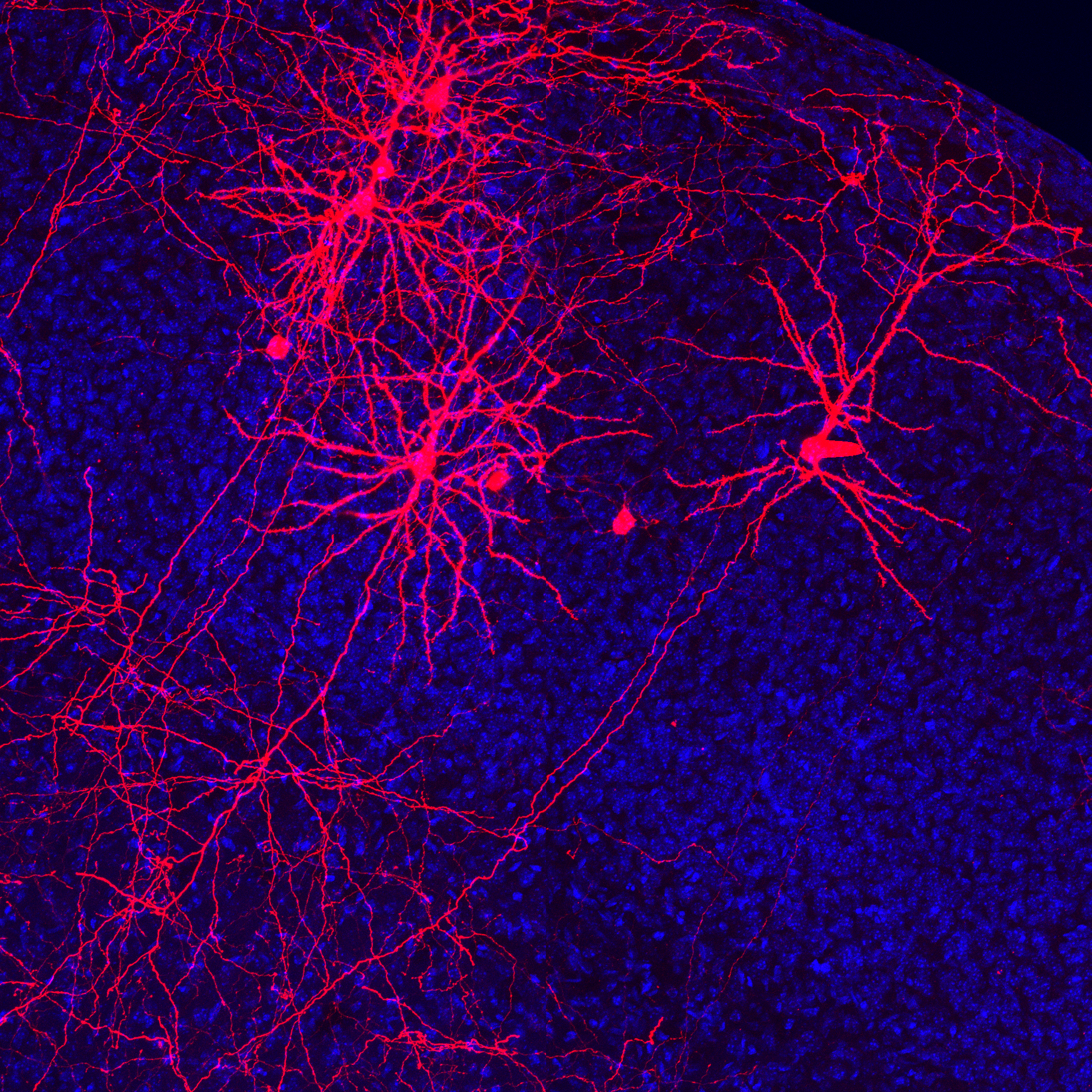

Supplement: Supplementary file 11 — EV Figures Source Data [file 44318_2024_50_MOESM11_ESM.zip › EV Figures-source files/FigureEV1-source files/FigureEV1B_control.tif]

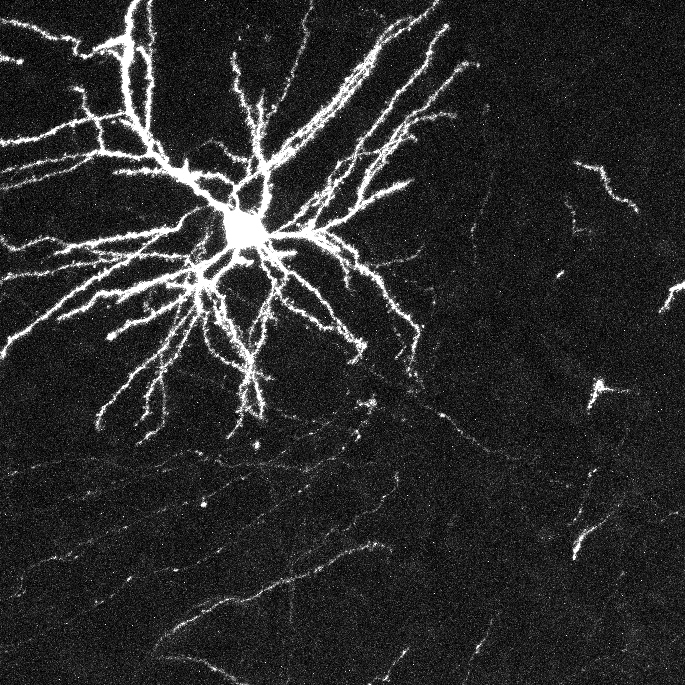

Supplement: Supplementary file 11 — EV Figures Source Data [file 44318_2024_50_MOESM11_ESM.zip › EV Figures-source files/FigureEV1-source files/C2-MAX_exp9-mouse2-slice2-gsk3bKO-1.tif]

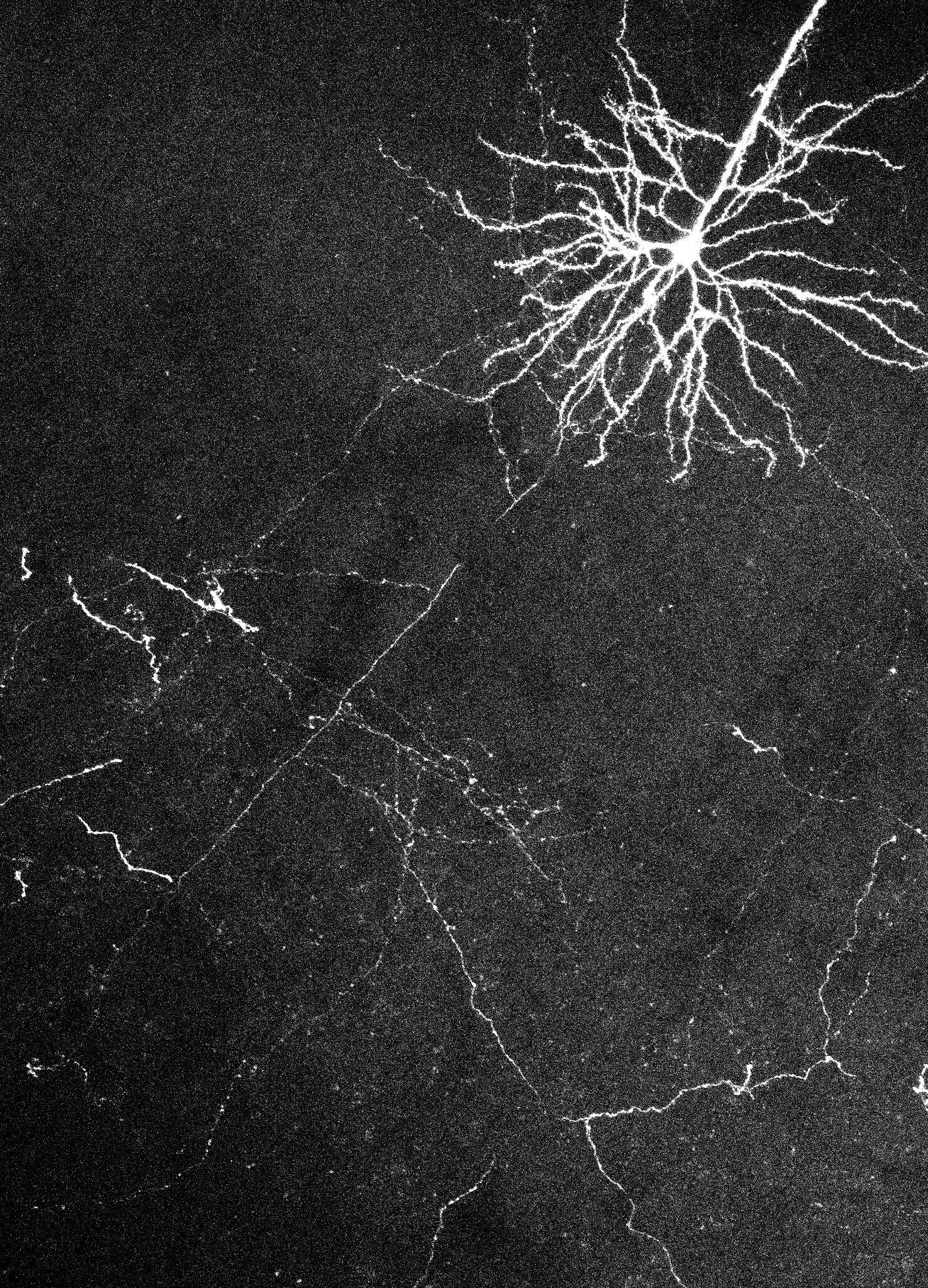

Supplement: Supplementary file 11 — EV Figures Source Data [file 44318_2024_50_MOESM11_ESM.zip › EV Figures-source files/FigureEV1-source files/FigureEV1D-control-single-1.tif]

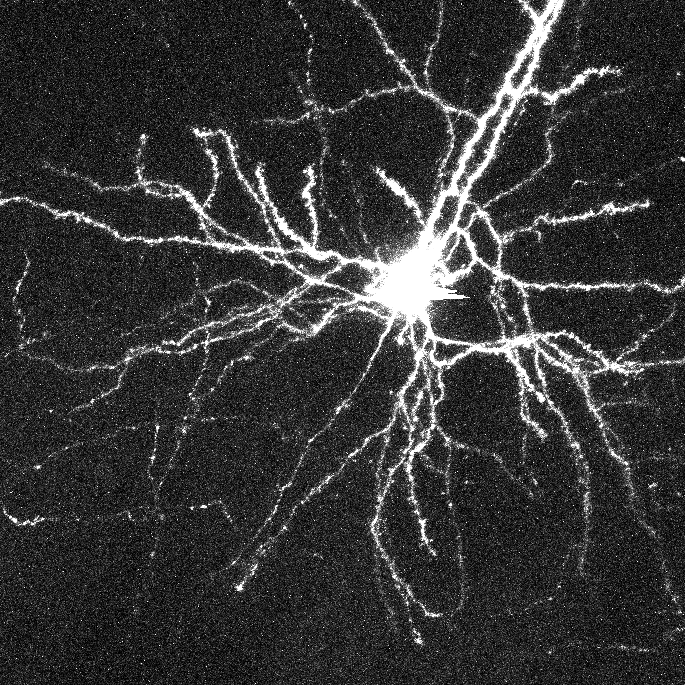

Supplement: Supplementary file 11 — EV Figures Source Data [file 44318_2024_50_MOESM11_ESM.zip › EV Figures-source files/FigureEV1-source files/C2-MAX_exp9-mouse5-slice1-doubleKO-1.tif]

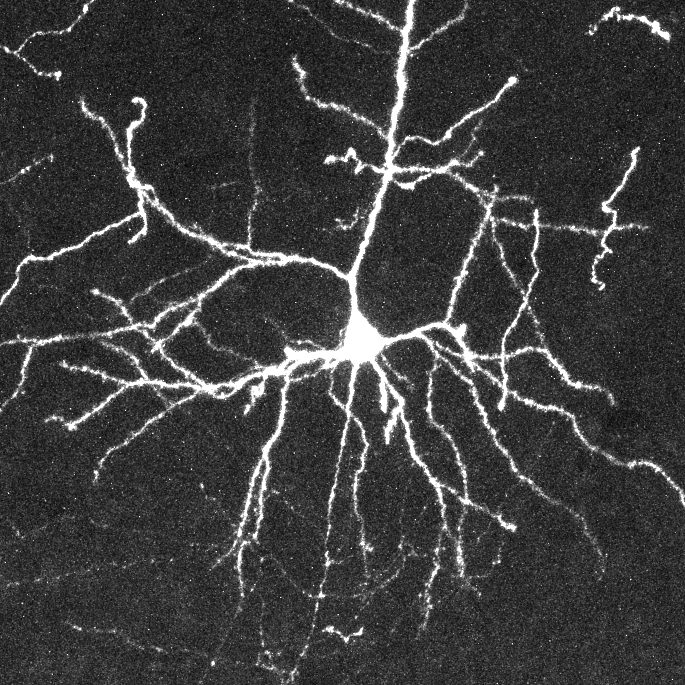

Supplement: Supplementary file 11 — EV Figures Source Data [file 44318_2024_50_MOESM11_ESM.zip › EV Figures-source files/FigureEV1-source files/C2-MAX_exp9-mouse5-slice6-doubleKO-1.tif]

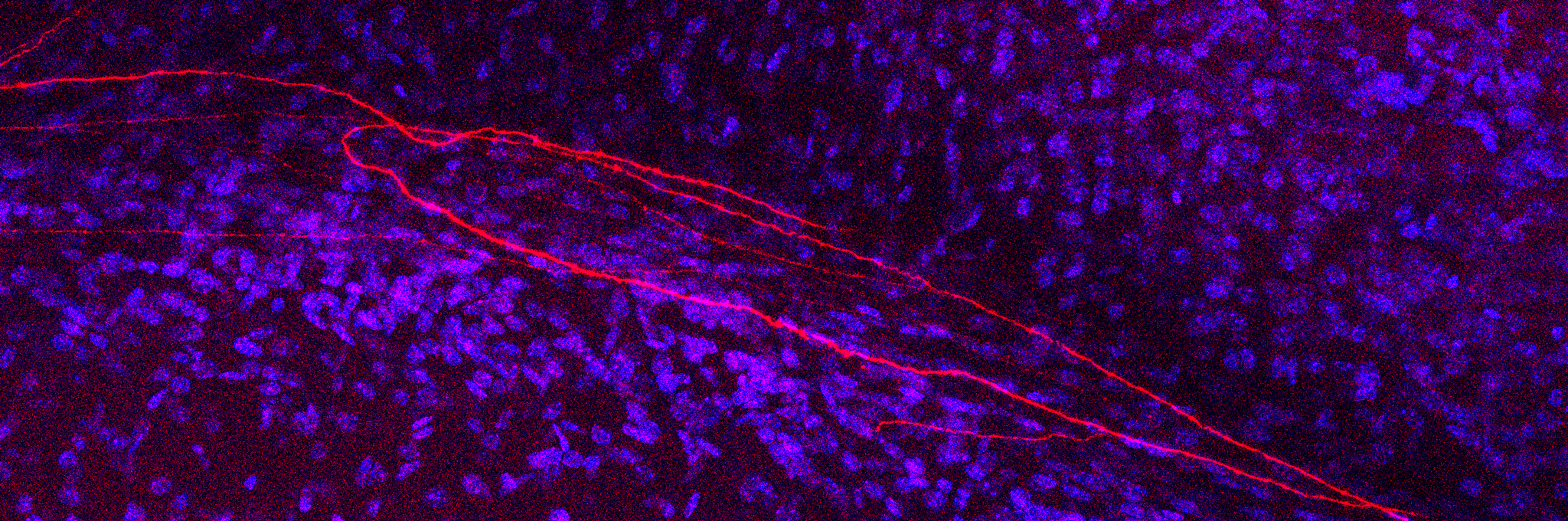

Supplement: Supplementary file 11 — EV Figures Source Data [file 44318_2024_50_MOESM11_ESM.zip › EV Figures-source files/FigureEV1-source files/FigureEV1C-loop2.tif]
