# Supplementary figures and images for: Microtubule-binding protein MAP1B regulates interstitial axon branching of cortical neurons via the tubulin tyrosination cycle (part 2 of 2)
Source: EMBO J. 2024 Feb 22;43(7):5. doi: 10.1038/s44318-024-00050-3 (PMC10987652; doi:10.1038/s44318-024-00050-3)

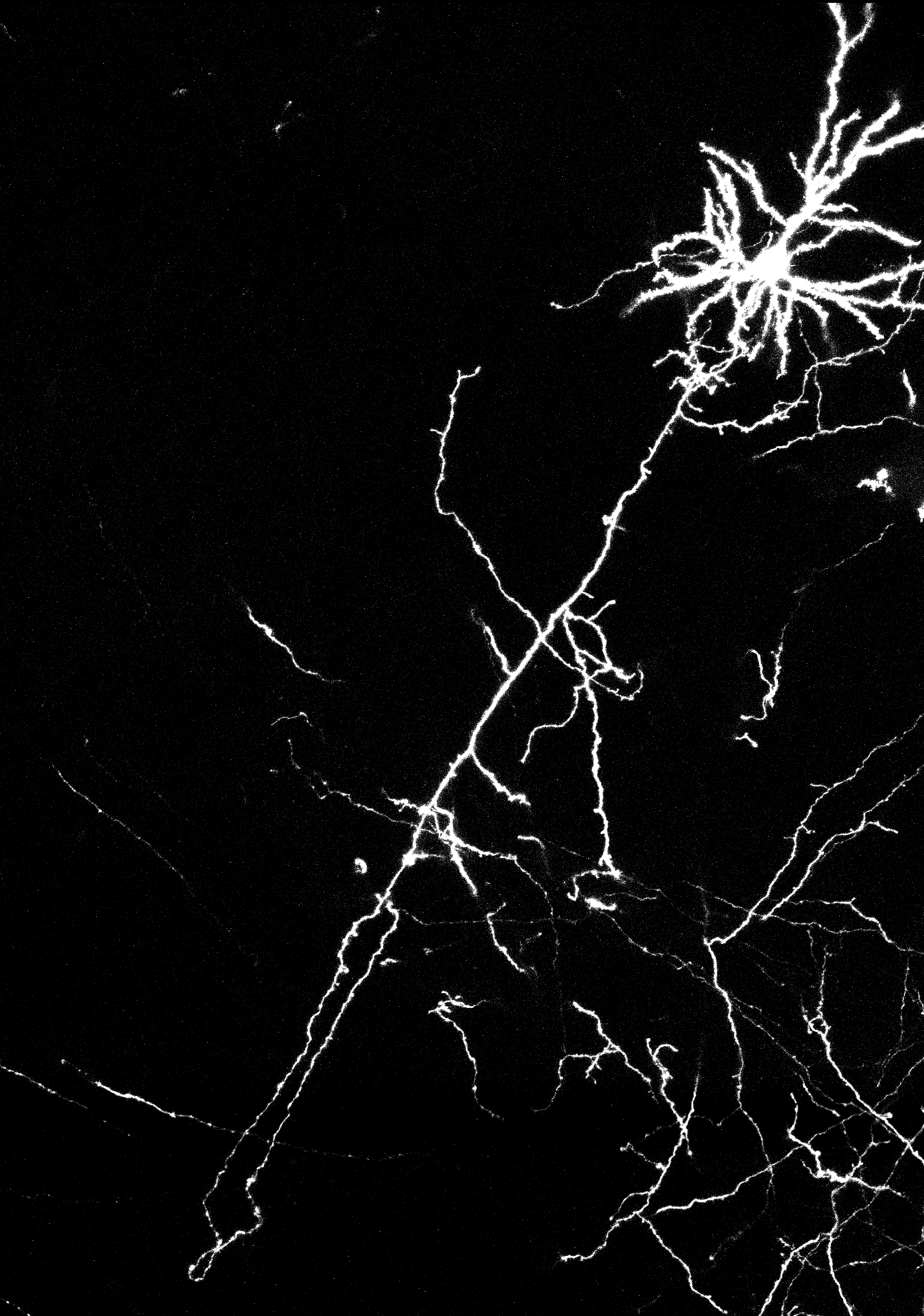

Supplement: Supplementary file 11 — EV Figures Source Data [file 44318_2024_50_MOESM11_ESM.zip › EV Figures-source files/FigureEV1-source files/FigureEV1C-looping primary axon.tif]

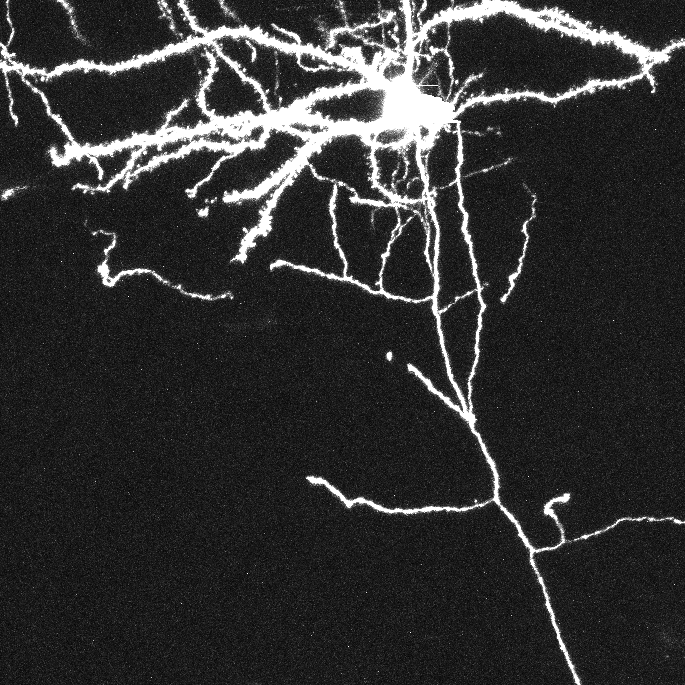

Supplement: Supplementary file 11 — EV Figures Source Data [file 44318_2024_50_MOESM11_ESM.zip › EV Figures-source files/FigureEV1-source files/MAX_exp1-mouse2-3-dendrite-doubleKO-1.tif]

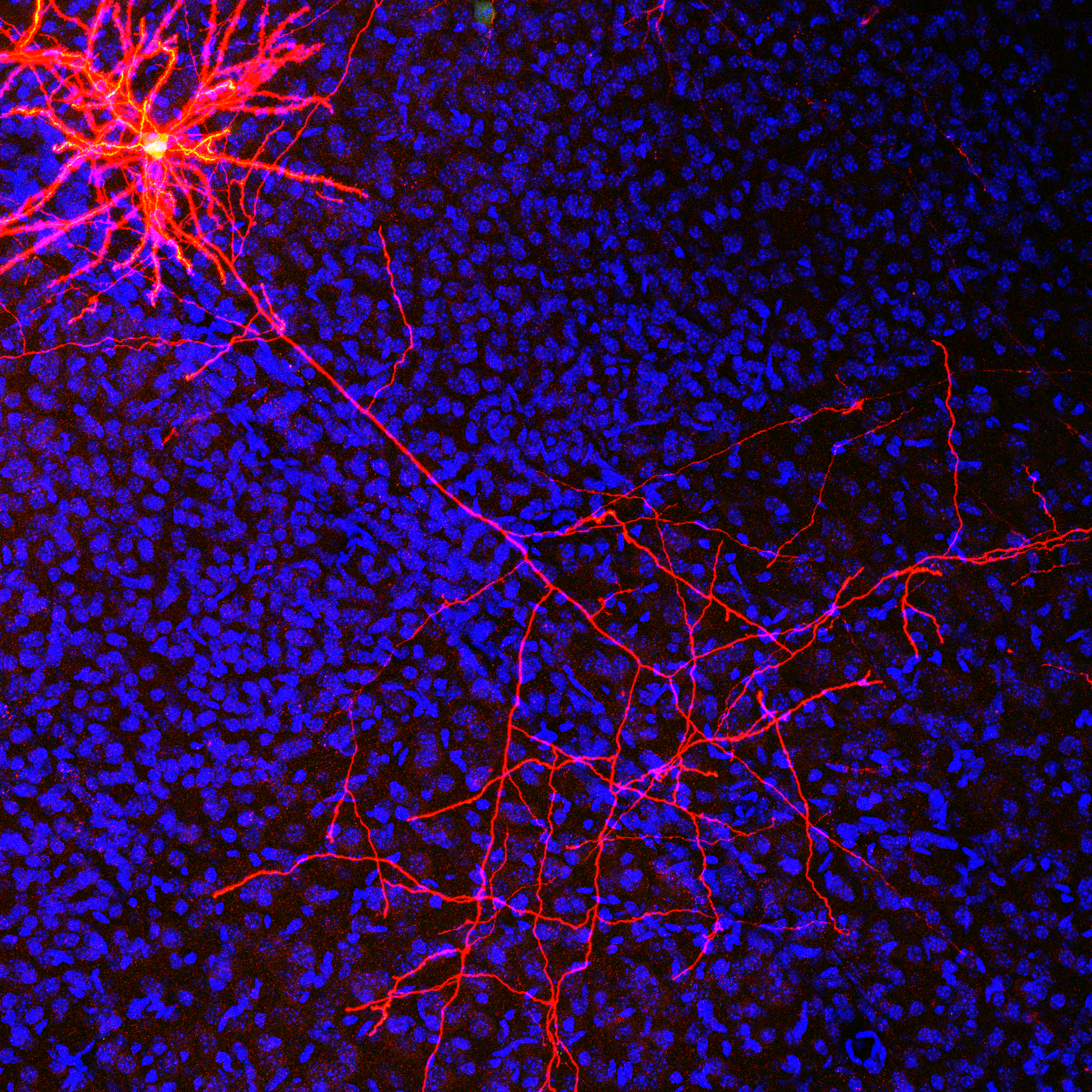

Supplement: Supplementary file 12 — Appendix Source Data [file 44318_2024_50_MOESM12_ESM.zip › Appendix-source files/Figure S10/S10B-control.tif]

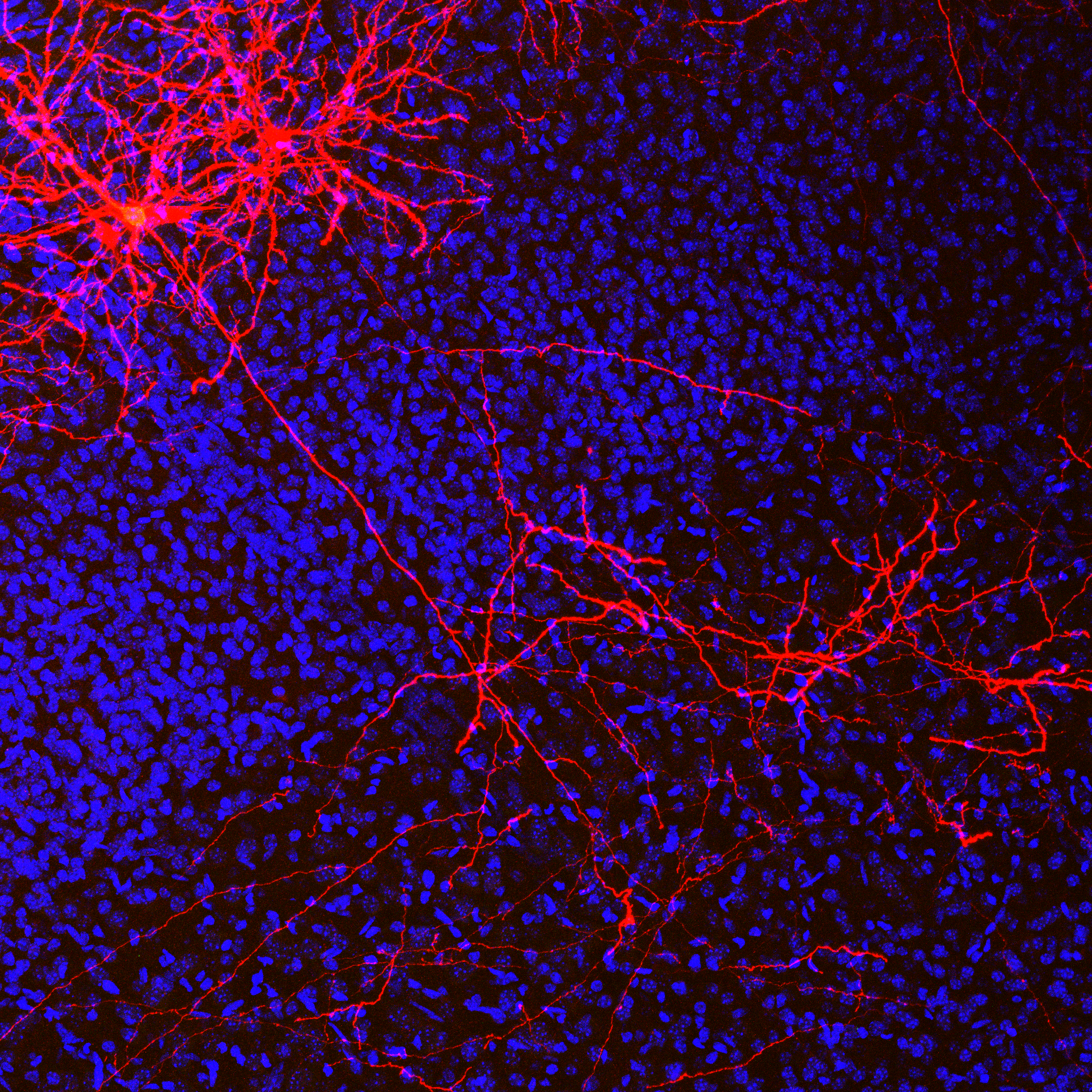

Supplement: Supplementary file 12 — Appendix Source Data [file 44318_2024_50_MOESM12_ESM.zip › Appendix-source files/Figure S10/S10B-Katanin-DN.tif]

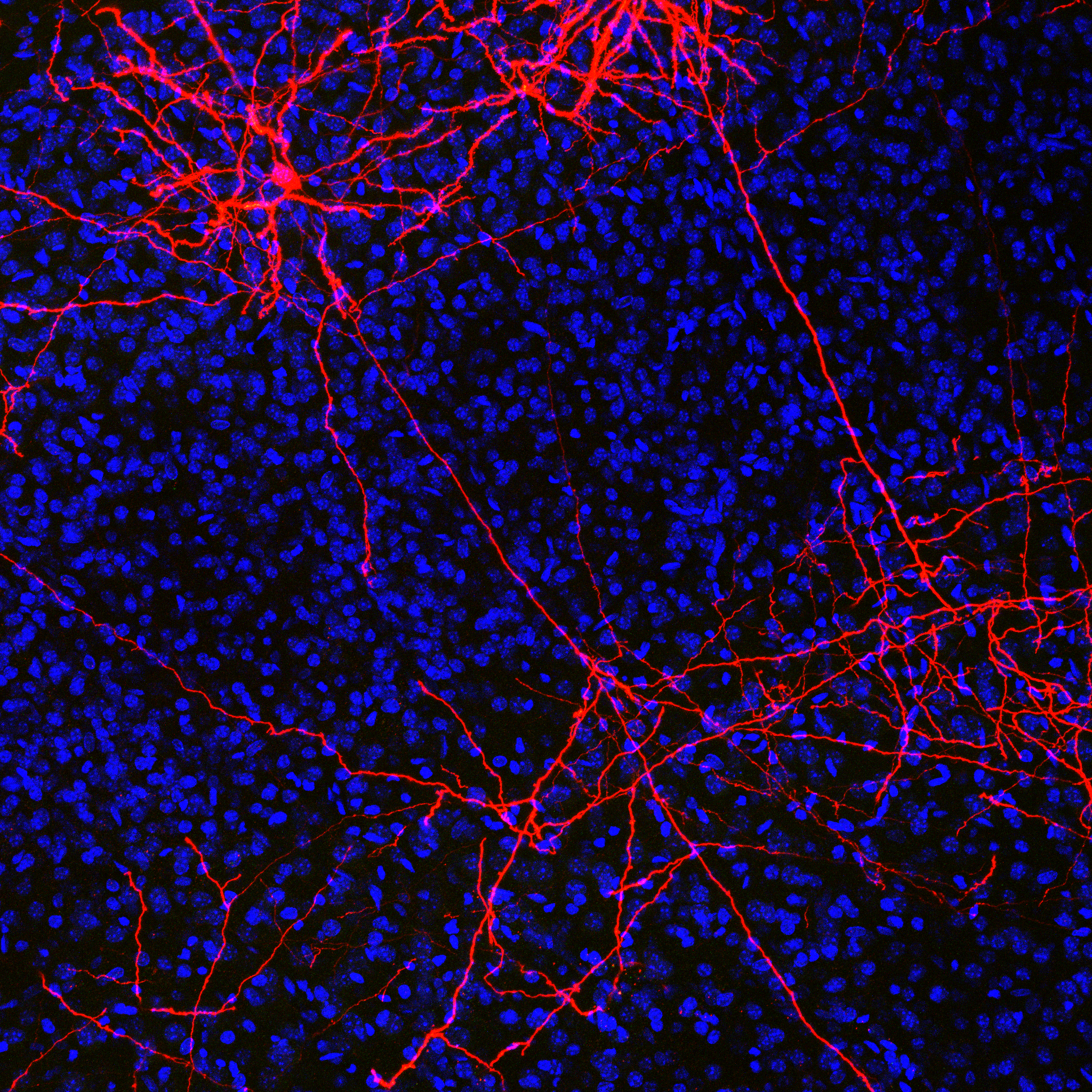

Supplement: Supplementary file 12 — Appendix Source Data [file 44318_2024_50_MOESM12_ESM.zip › Appendix-source files/Figure S10/S10B-Katanin.tif]

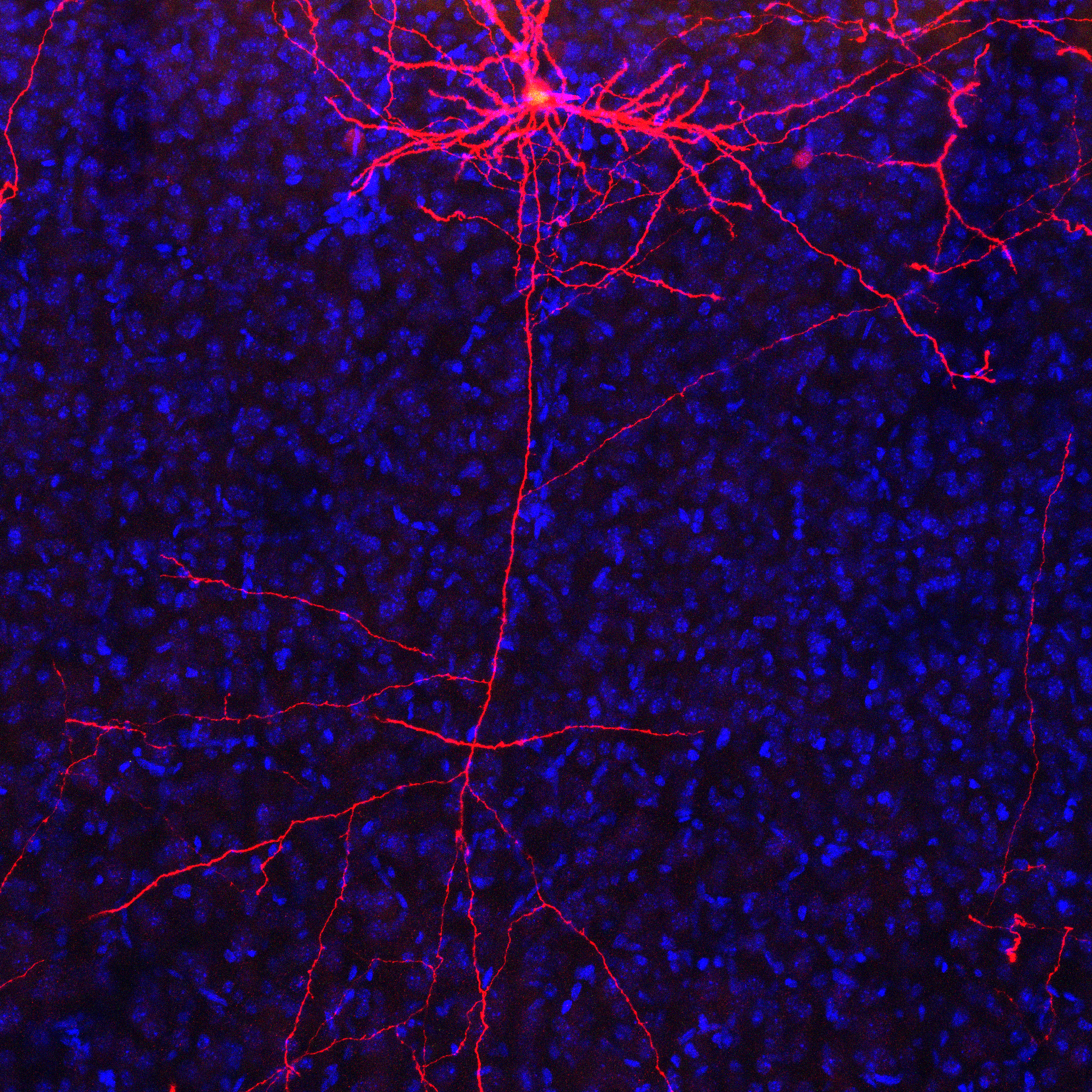

Supplement: Supplementary file 12 — Appendix Source Data [file 44318_2024_50_MOESM12_ESM.zip › Appendix-source files/Figure S10/S10A-Katna1 gRNA.tif]

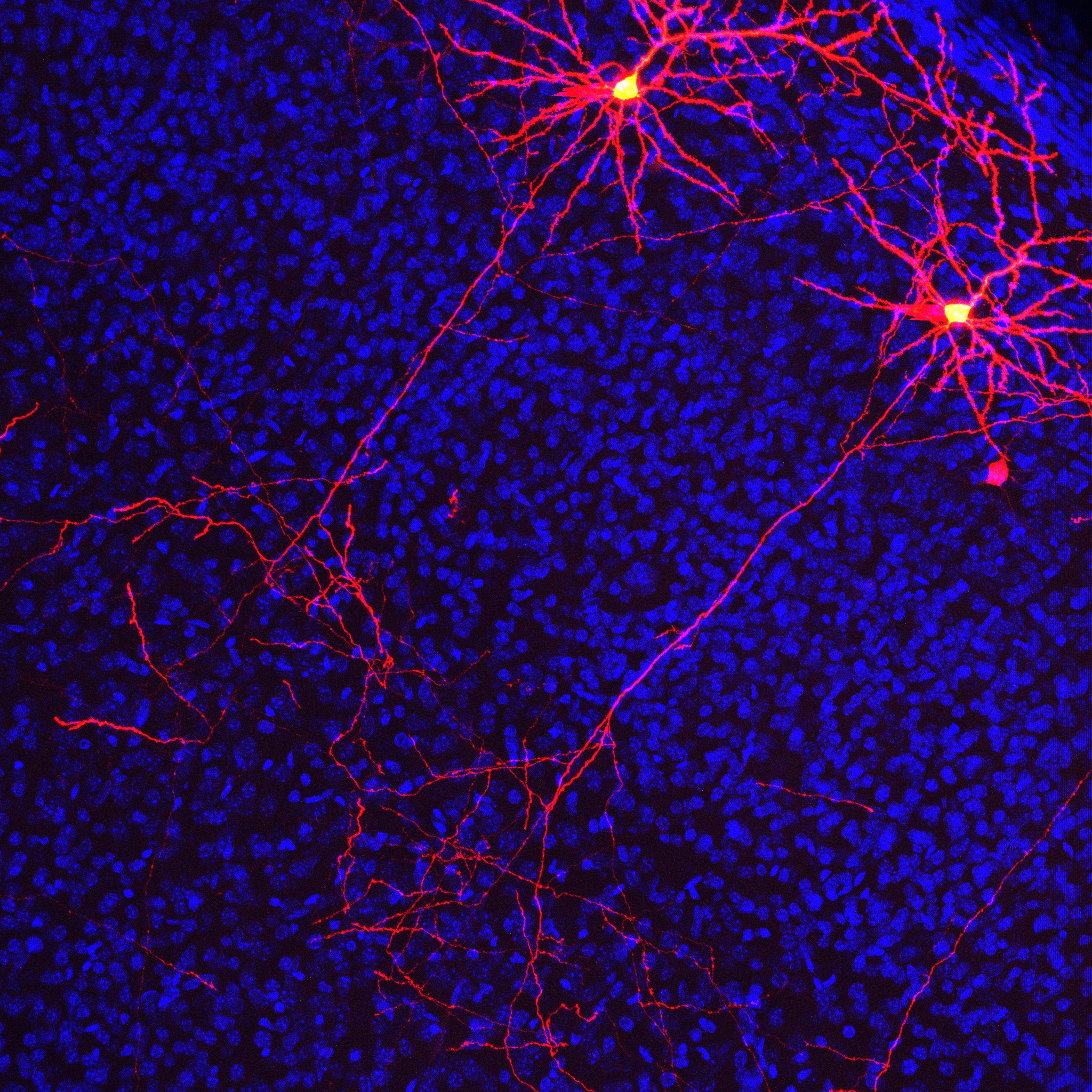

Supplement: Supplementary file 12 — Appendix Source Data [file 44318_2024_50_MOESM12_ESM.zip › Appendix-source files/Figure S10/S10A-control.tif]

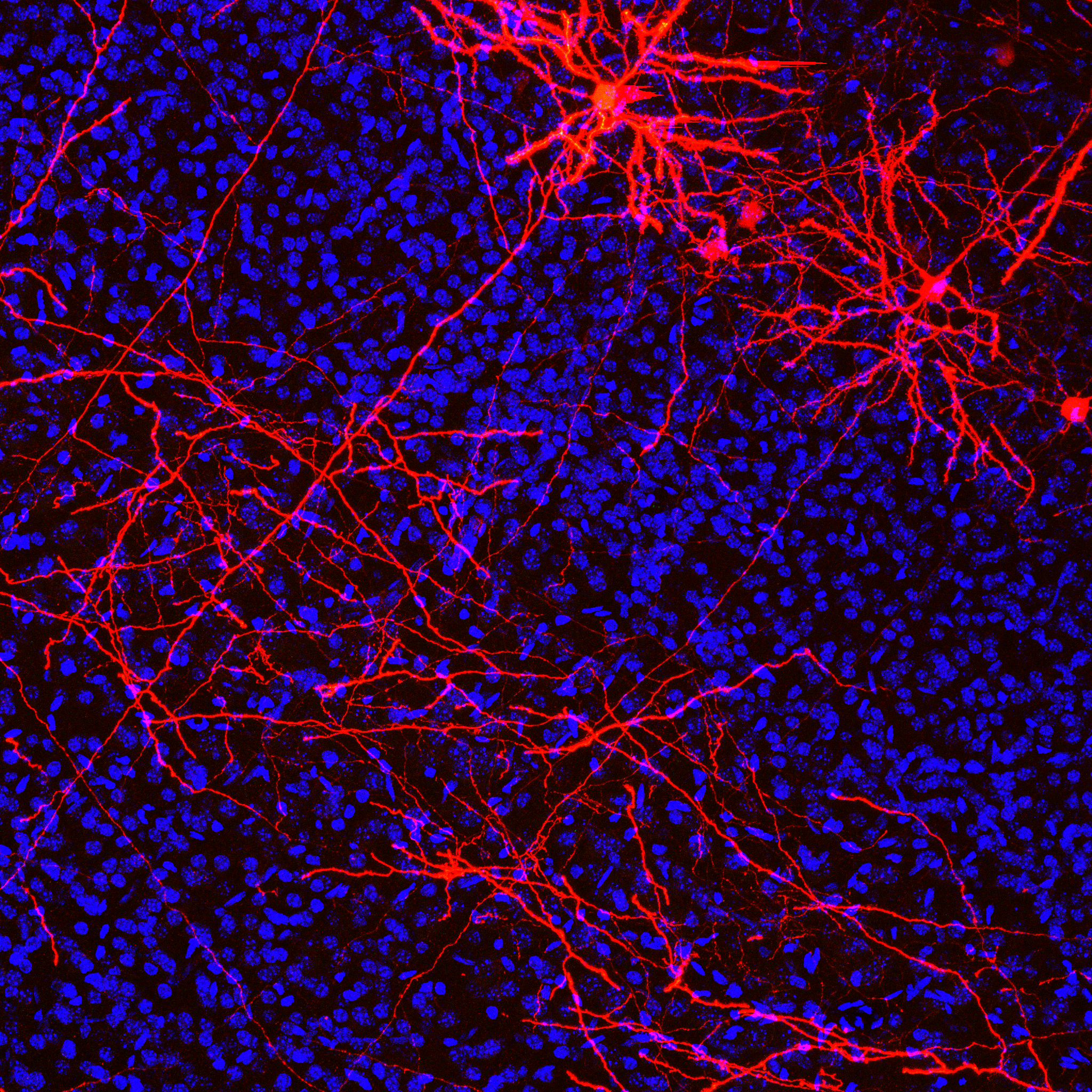

Supplement: Supplementary file 12 — Appendix Source Data [file 44318_2024_50_MOESM12_ESM.zip › Appendix-source files/Figure S10/S10B-Katanin&TTL.tif]

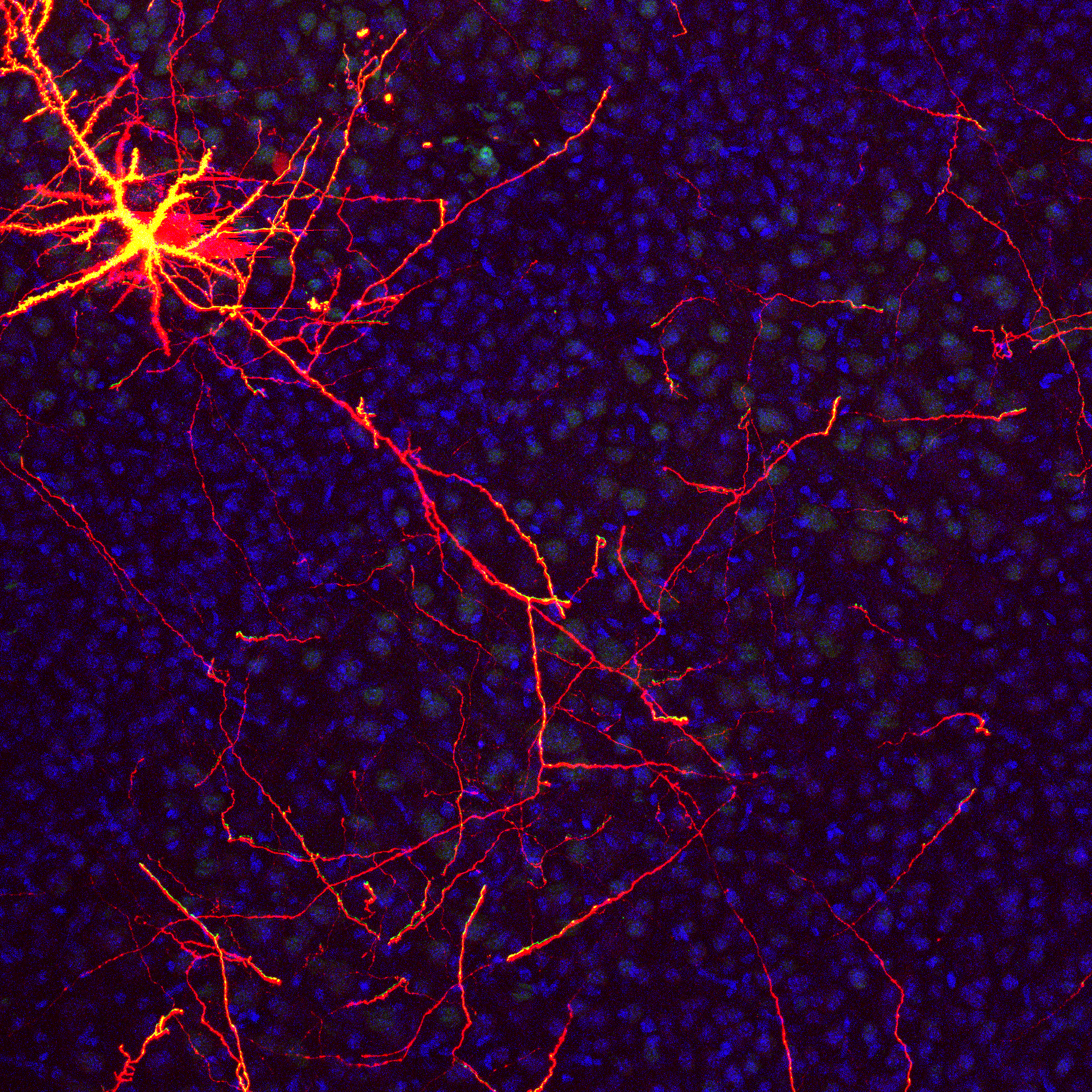

Supplement: Supplementary file 12 — Appendix Source Data [file 44318_2024_50_MOESM12_ESM.zip › Appendix-source files/Figure S10/S10B-TTL.tif]

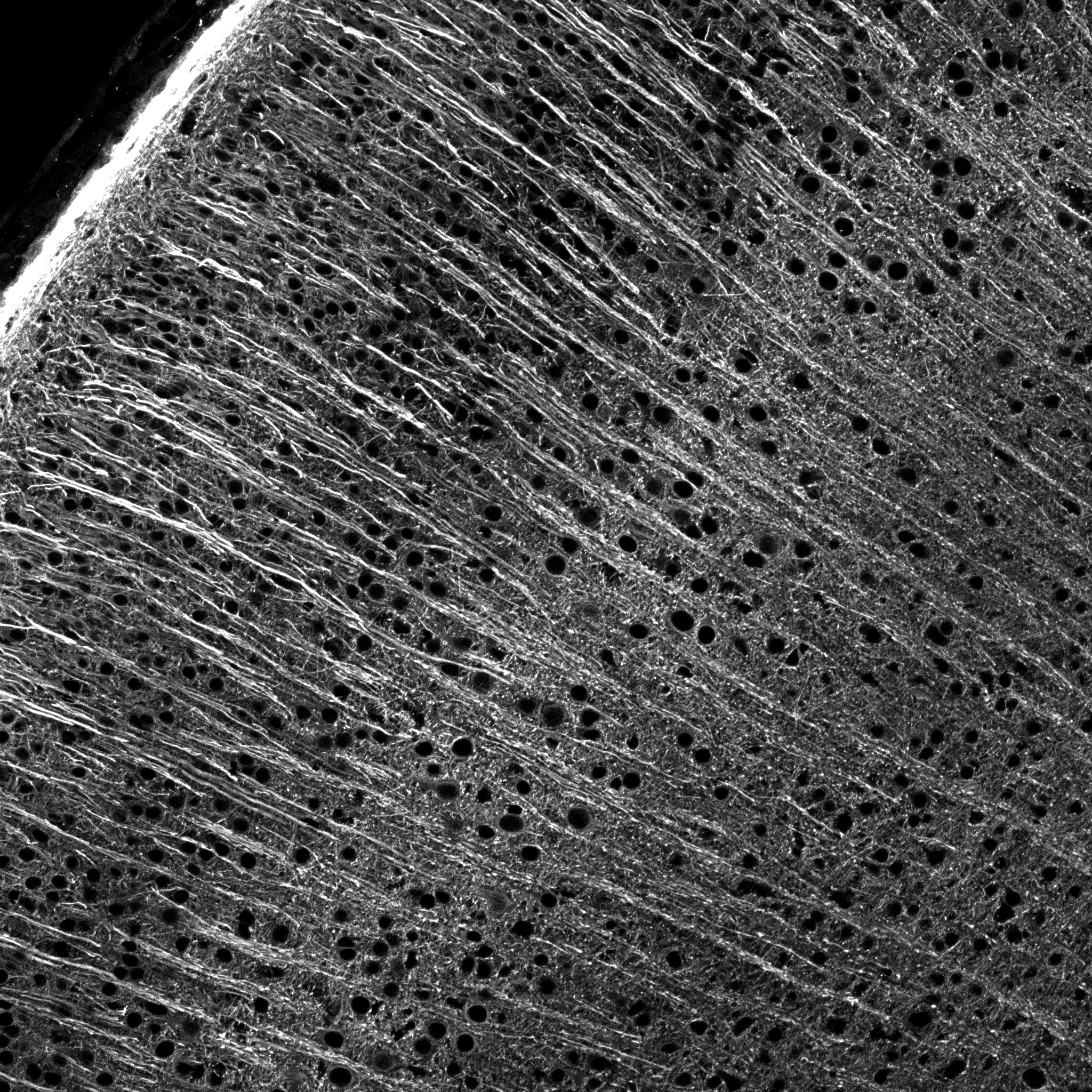

Supplement: Supplementary file 12 — Appendix Source Data [file 44318_2024_50_MOESM12_ESM.zip › Appendix-source files/Figure S6/S6B-Acetyl-Tubulin-example.tif]

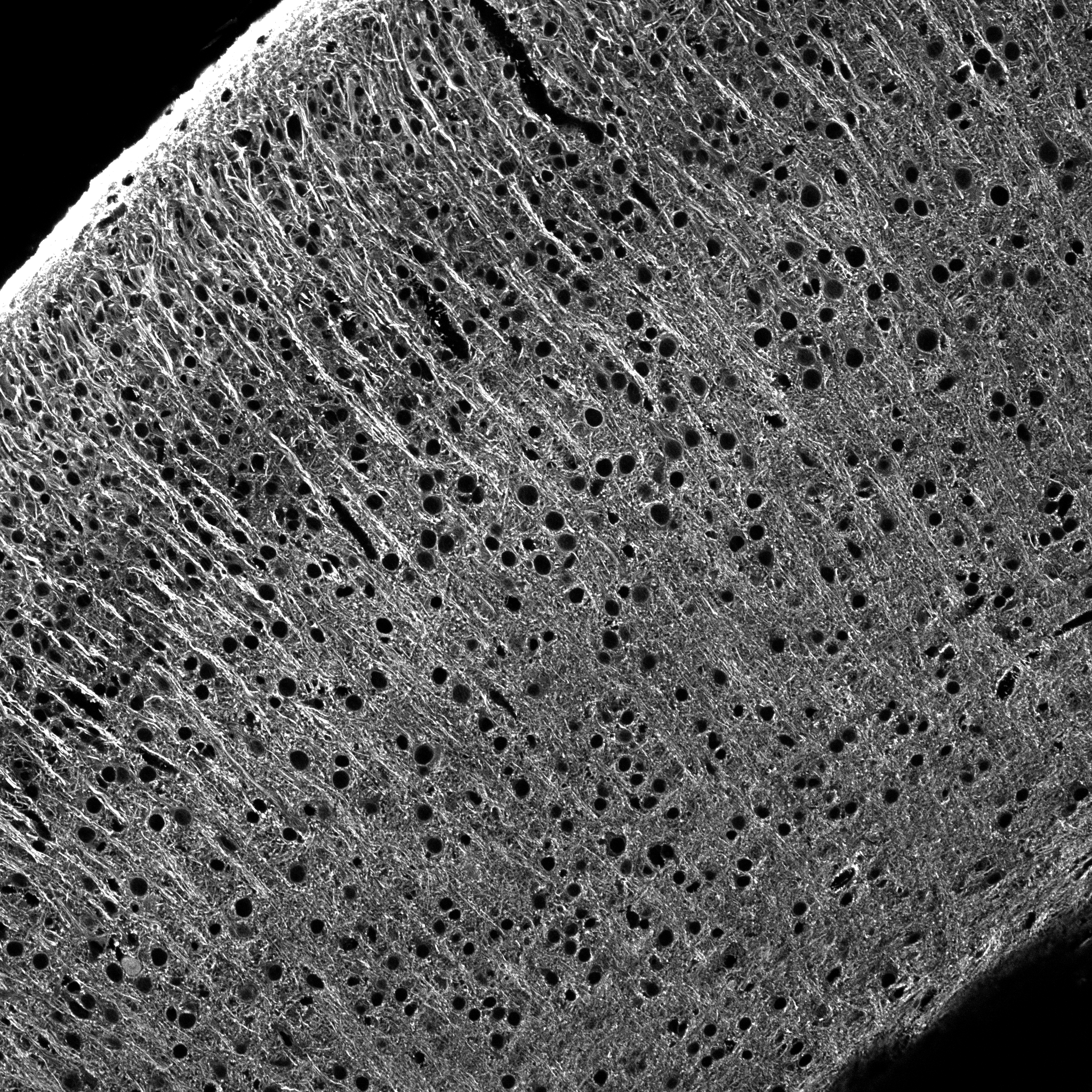

Supplement: Supplementary file 12 — Appendix Source Data [file 44318_2024_50_MOESM12_ESM.zip › Appendix-source files/Figure S6/S6B-Detyr-Tubulin-example.tif]

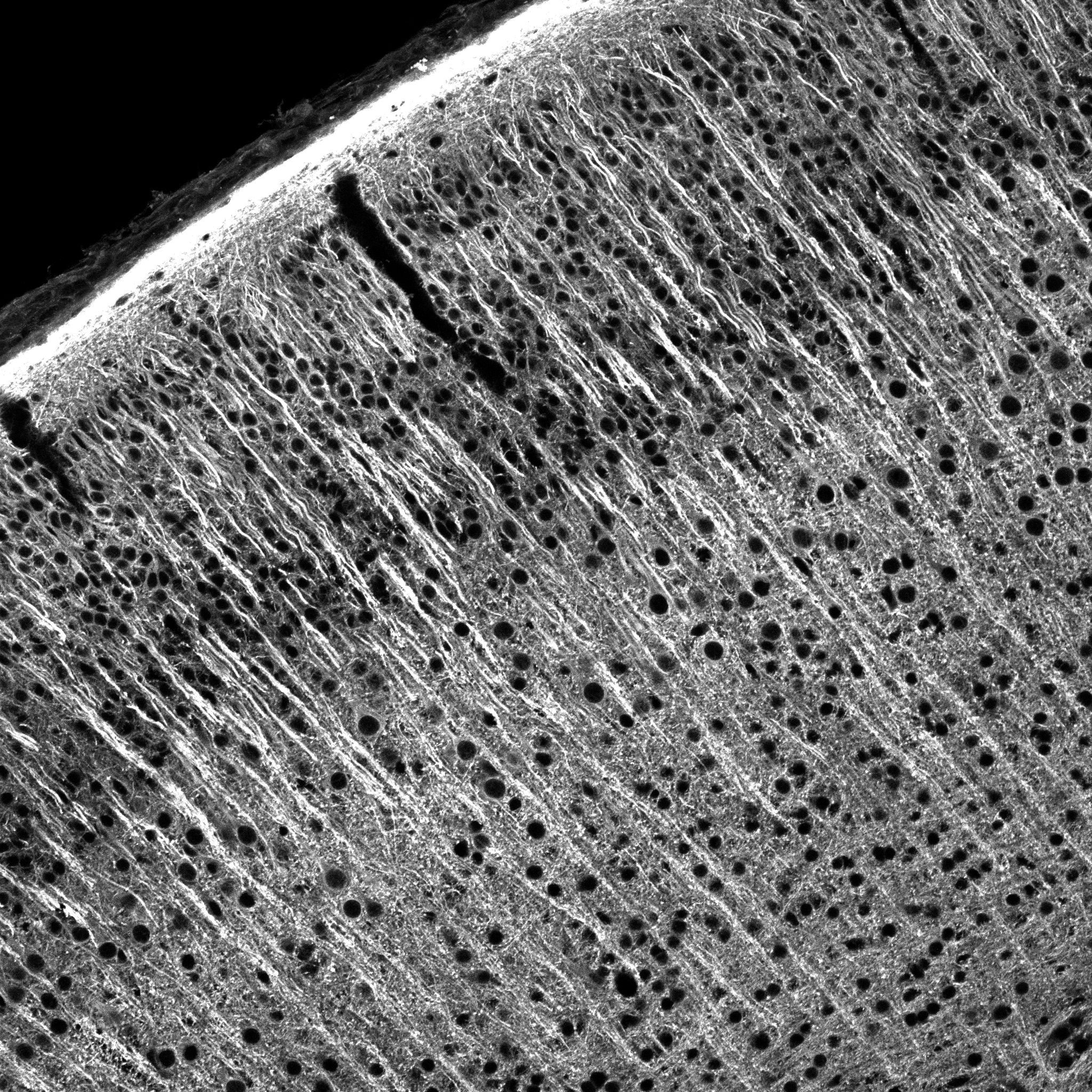

Supplement: Supplementary file 12 — Appendix Source Data [file 44318_2024_50_MOESM12_ESM.zip › Appendix-source files/Figure S6/S6B-Tubulin-example.tif]

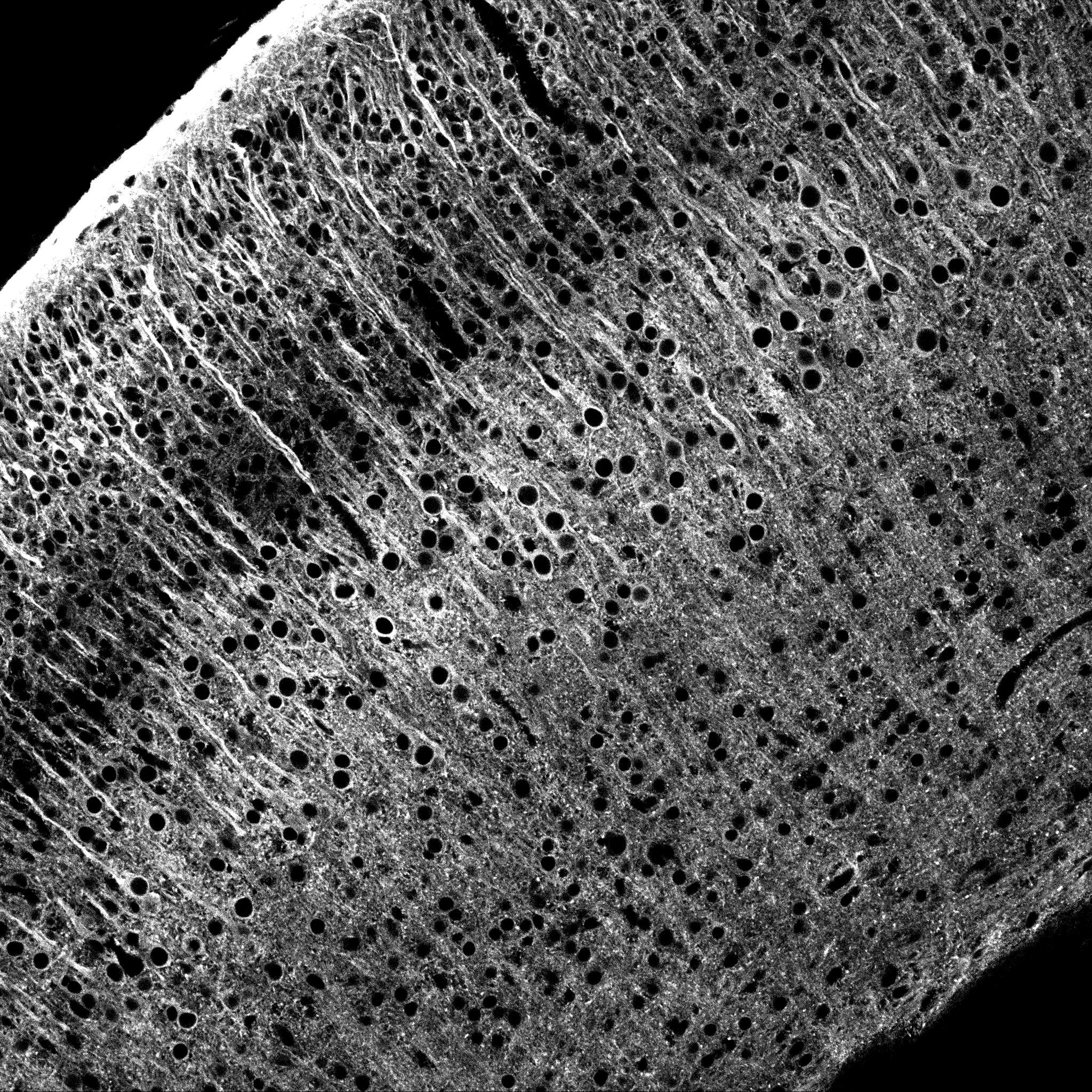

Supplement: Supplementary file 12 — Appendix Source Data [file 44318_2024_50_MOESM12_ESM.zip › Appendix-source files/Figure S6/S6B-Tyr-Tubulin-example.tif]

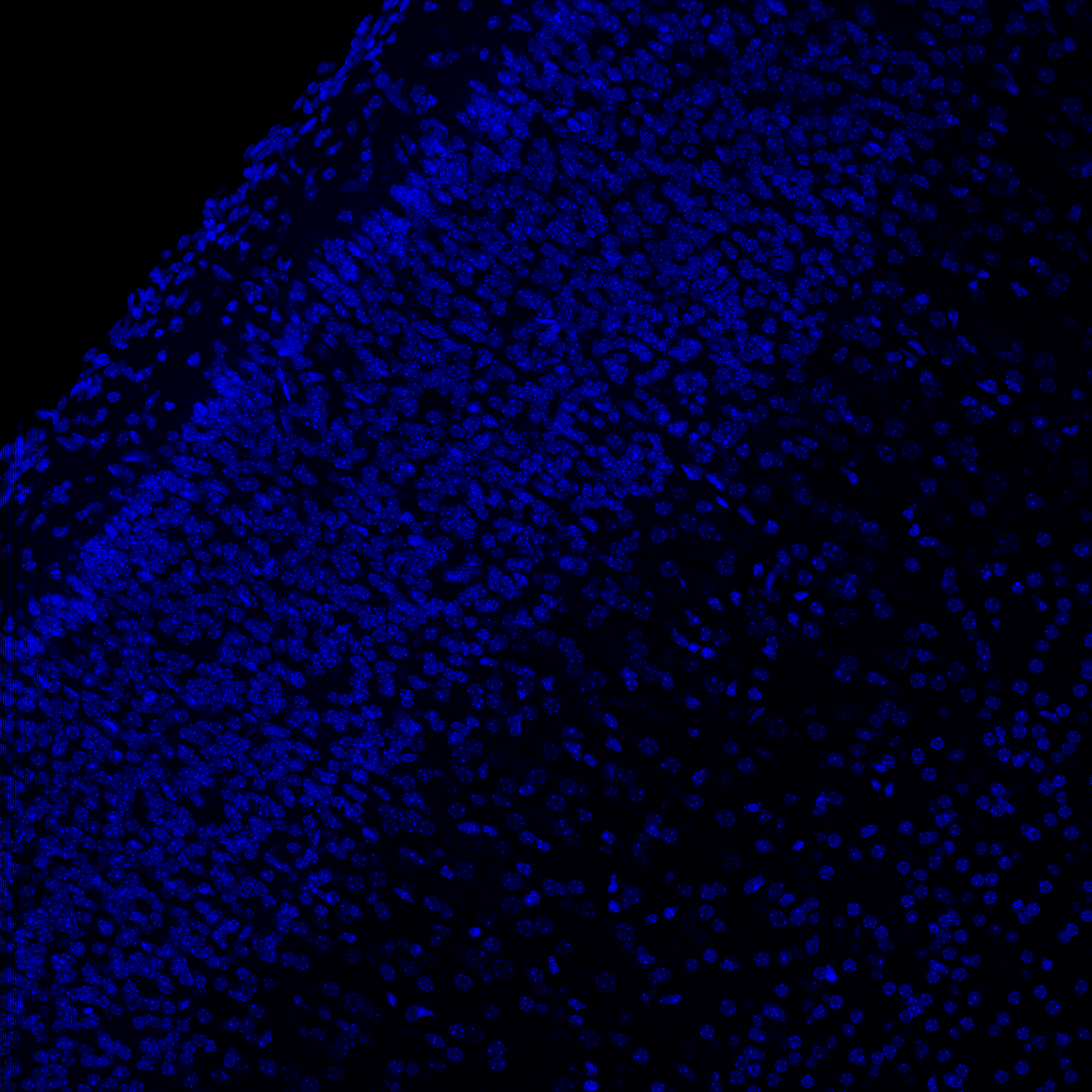

Supplement: Supplementary file 12 — Appendix Source Data [file 44318_2024_50_MOESM12_ESM.zip › Appendix-source files/Figure S6/S6A-P4-mouse2-DAPI-1.tif]

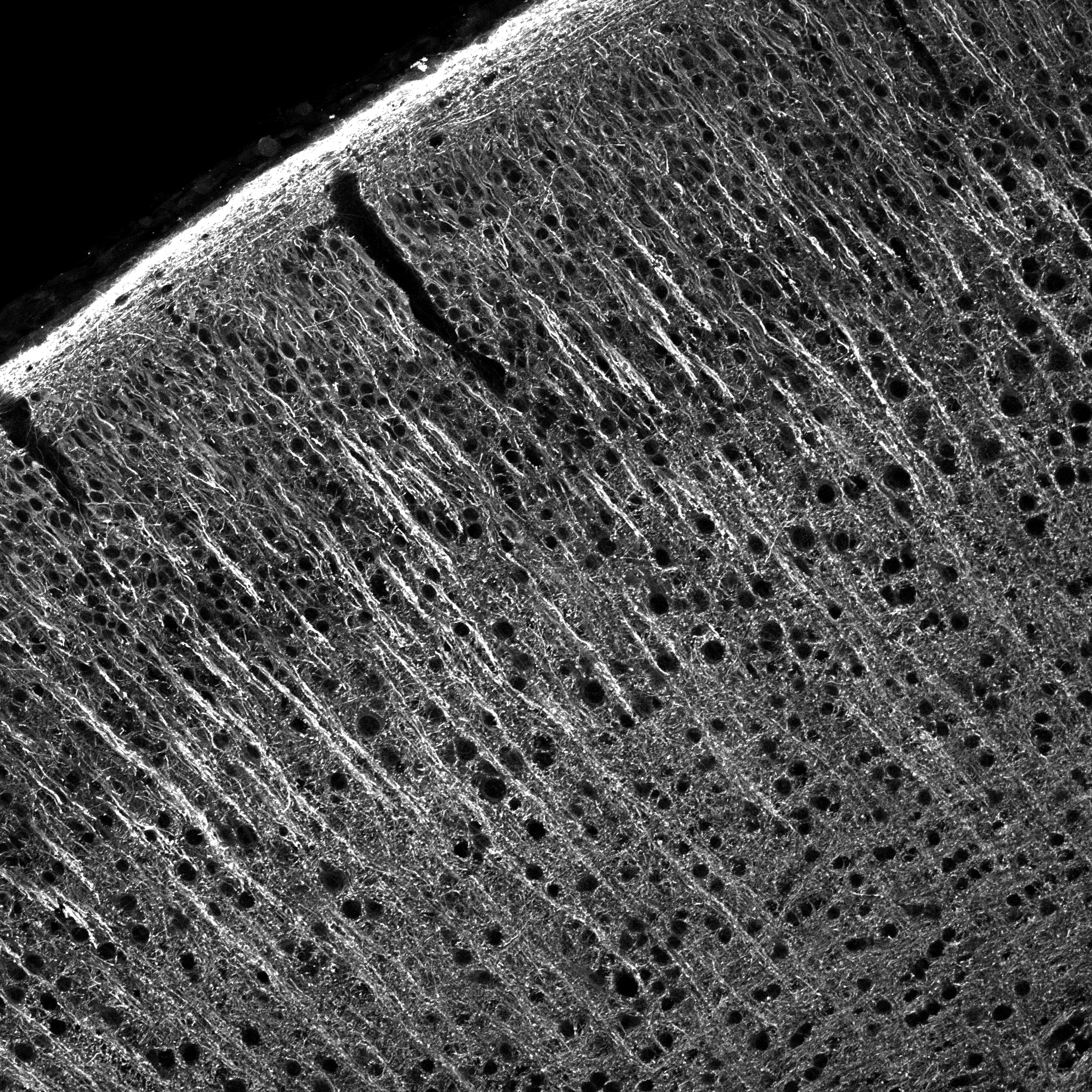

Supplement: Supplementary file 12 — Appendix Source Data [file 44318_2024_50_MOESM12_ESM.zip › Appendix-source files/Figure S6/S6B-PolyE-Tubulin-example.tif]

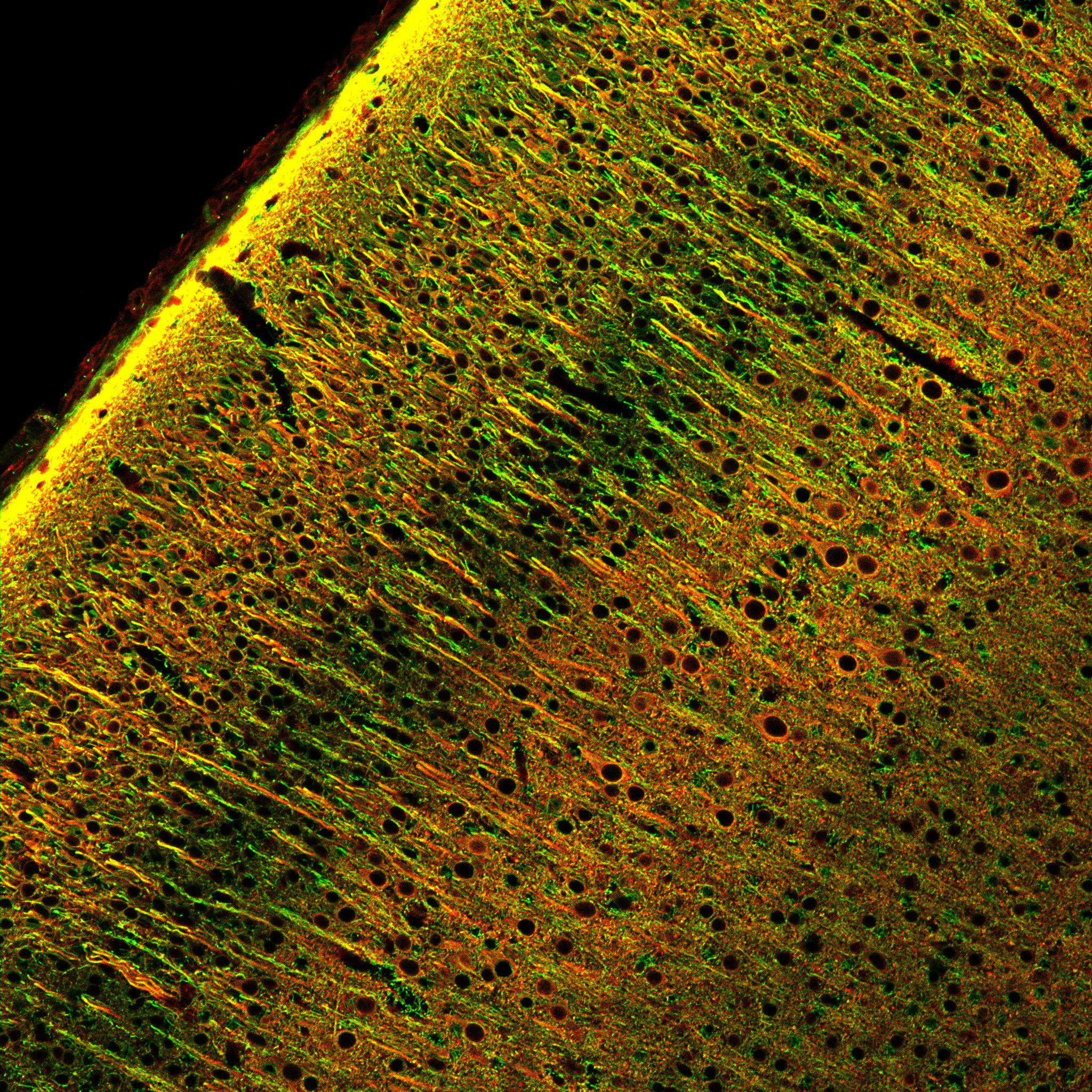

Supplement: Supplementary file 12 — Appendix Source Data [file 44318_2024_50_MOESM12_ESM.zip › Appendix-source files/Figure S6/S6A-P4-mouse2-tyr&detyr.tif]

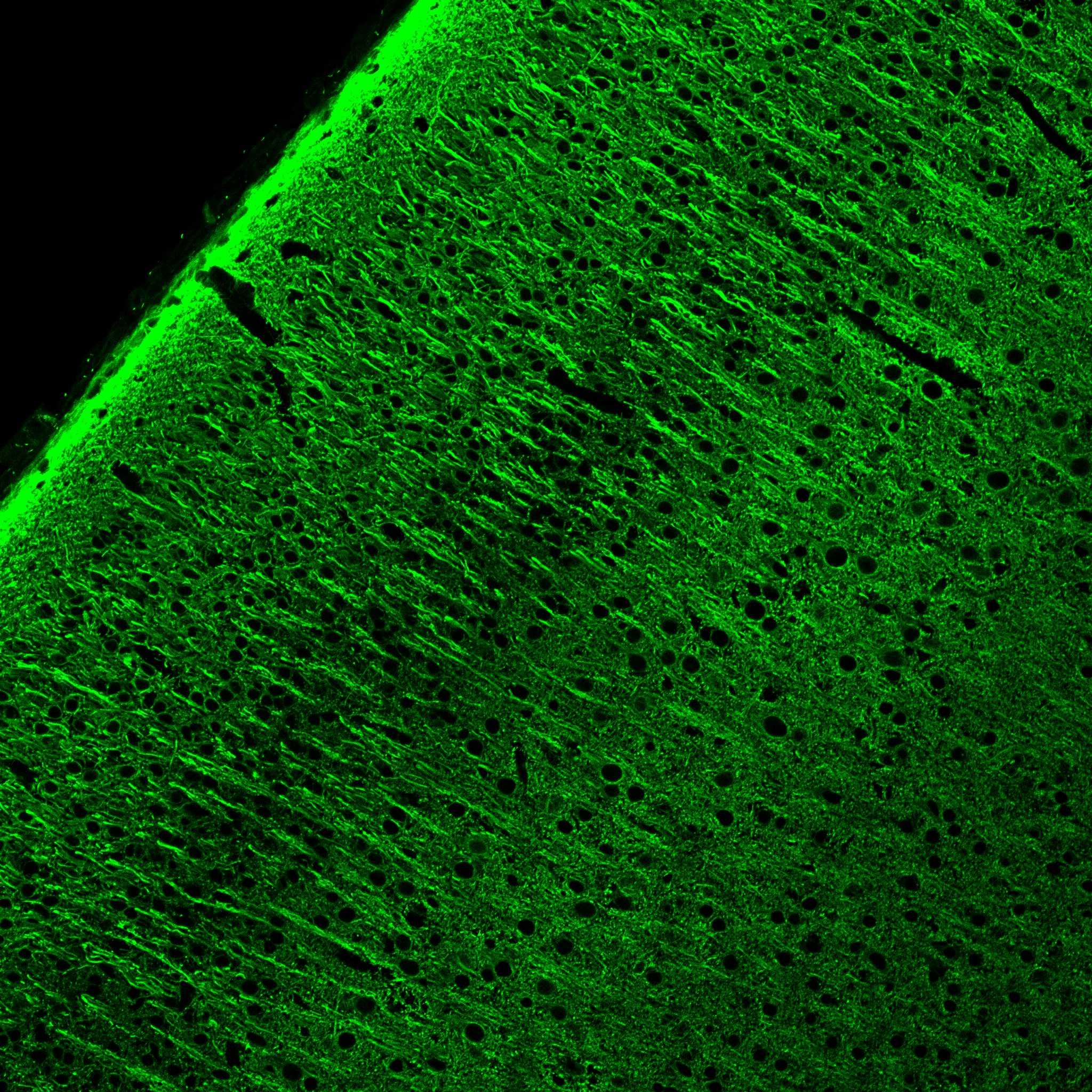

Supplement: Supplementary file 12 — Appendix Source Data [file 44318_2024_50_MOESM12_ESM.zip › Appendix-source files/Figure S6/S6A-P4-mouse2-detyr-1.tif]

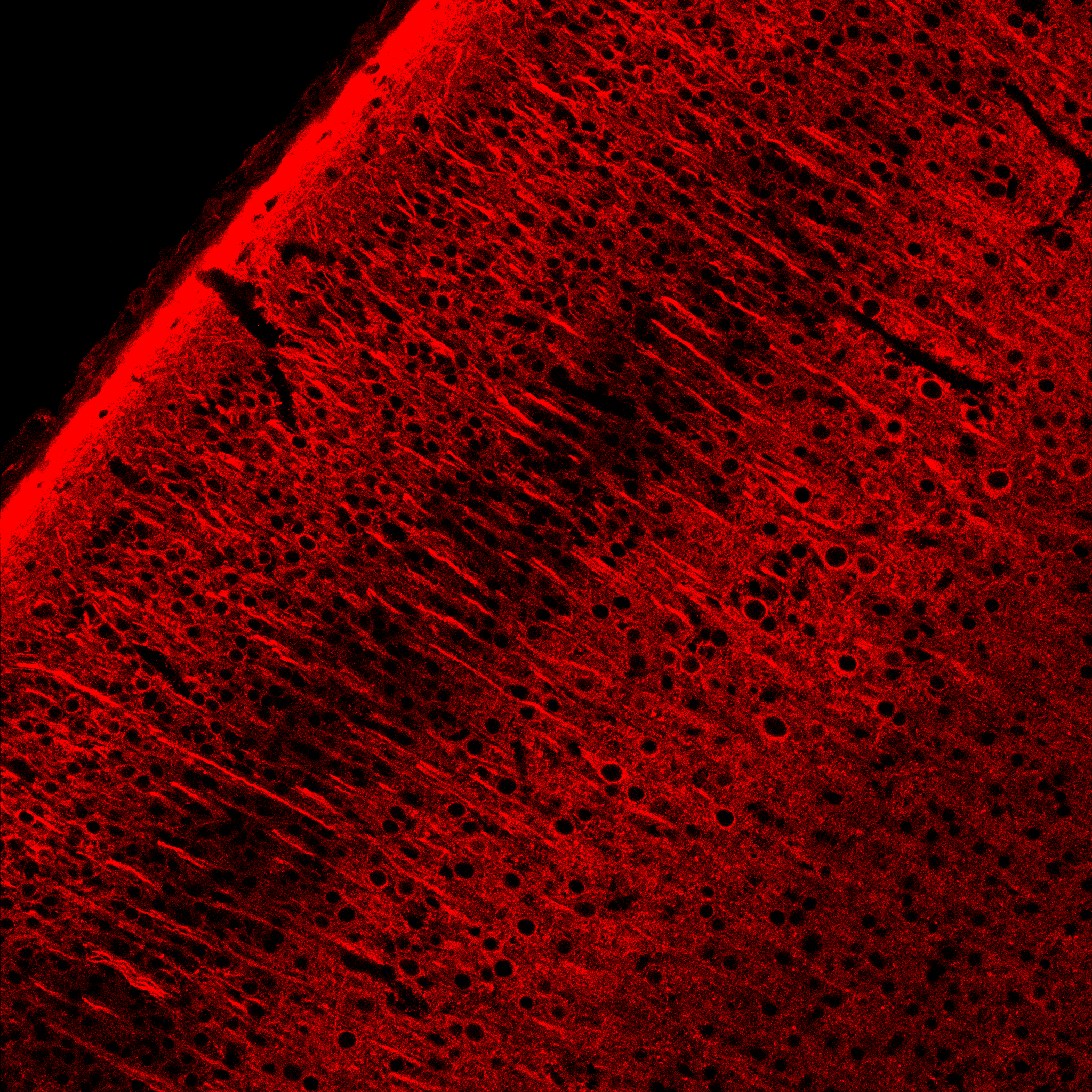

Supplement: Supplementary file 12 — Appendix Source Data [file 44318_2024_50_MOESM12_ESM.zip › Appendix-source files/Figure S6/S6A-P4-mouse2-tyr-1.tif]

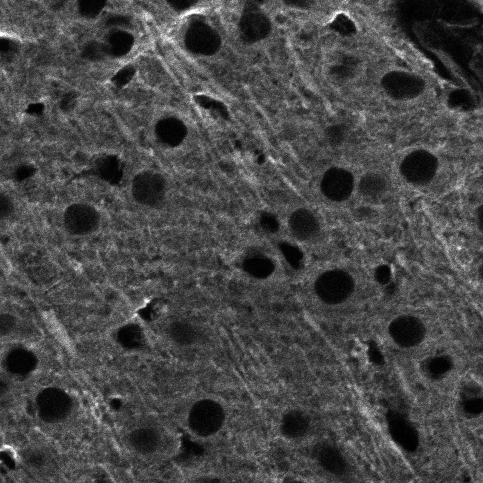

Supplement: Supplementary file 12 — Appendix Source Data [file 44318_2024_50_MOESM12_ESM.zip › Appendix-source files/Figure S7/C2-MAX_exp401-P7-L2-Tub647-PolyET555-slice4.tif]

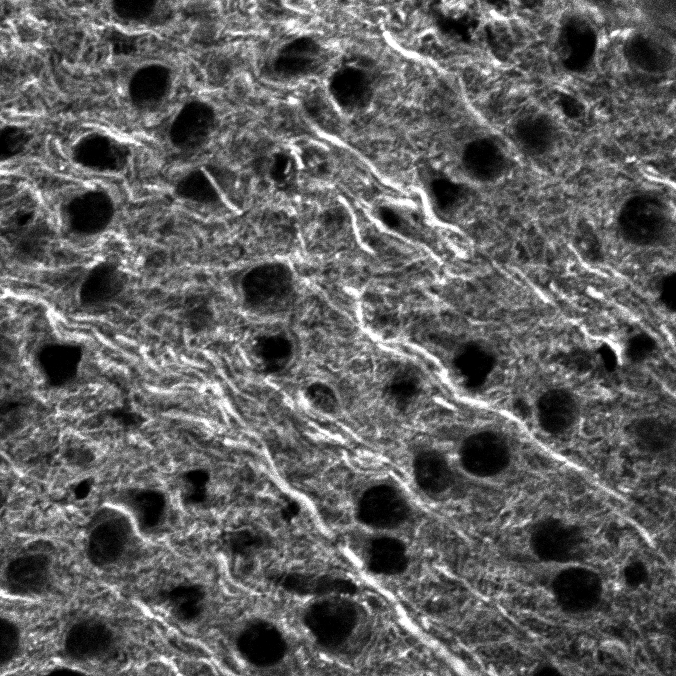

Supplement: Supplementary file 12 — Appendix Source Data [file 44318_2024_50_MOESM12_ESM.zip › Appendix-source files/Figure S7/C2-MAX_exp401-P7-L1-AceT647-PolyET555-slice4.tif]

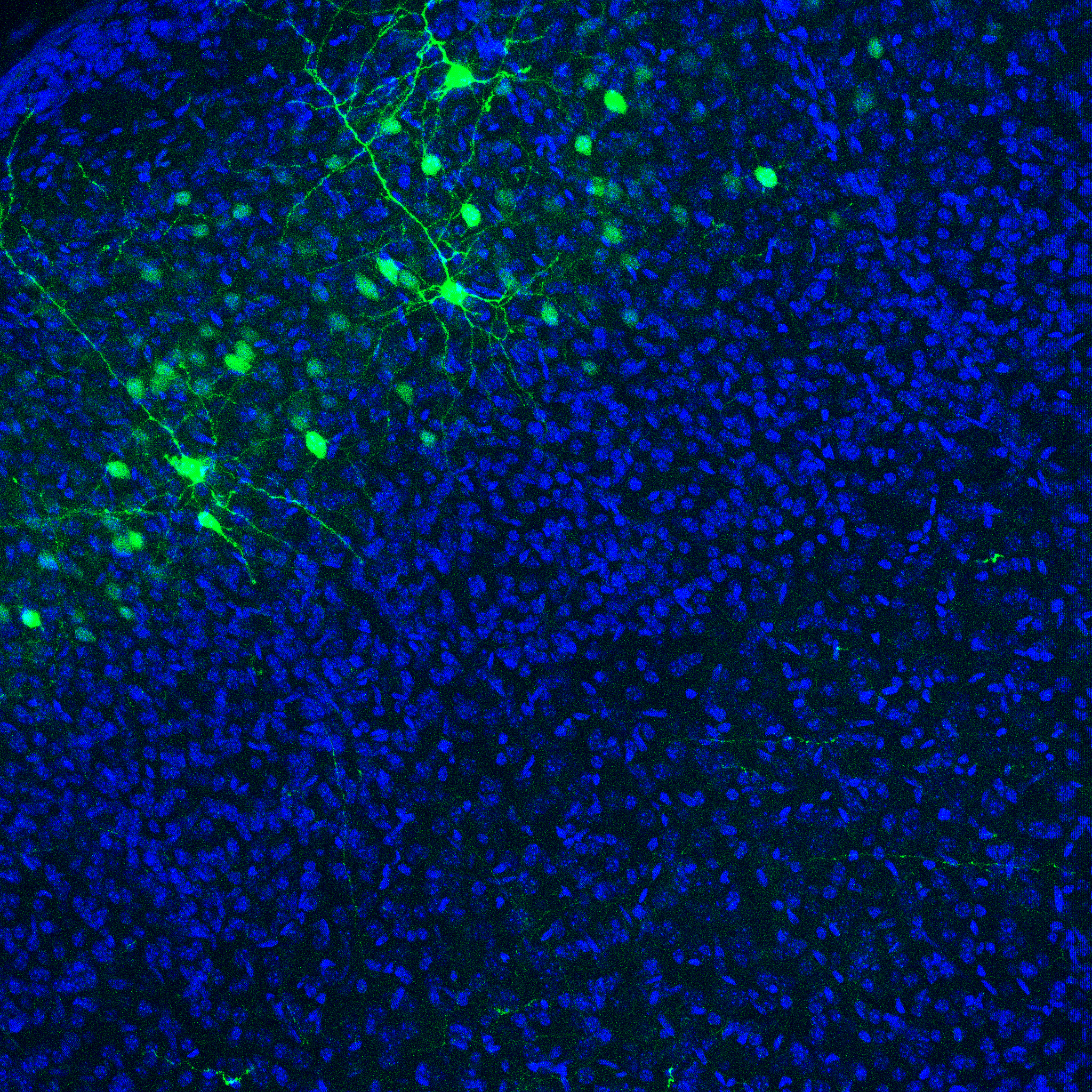

Supplement: Supplementary file 12 — Appendix Source Data [file 44318_2024_50_MOESM12_ESM.zip › Appendix-source files/Figure S7/Appendix Figure S7C-SVBP.tif]

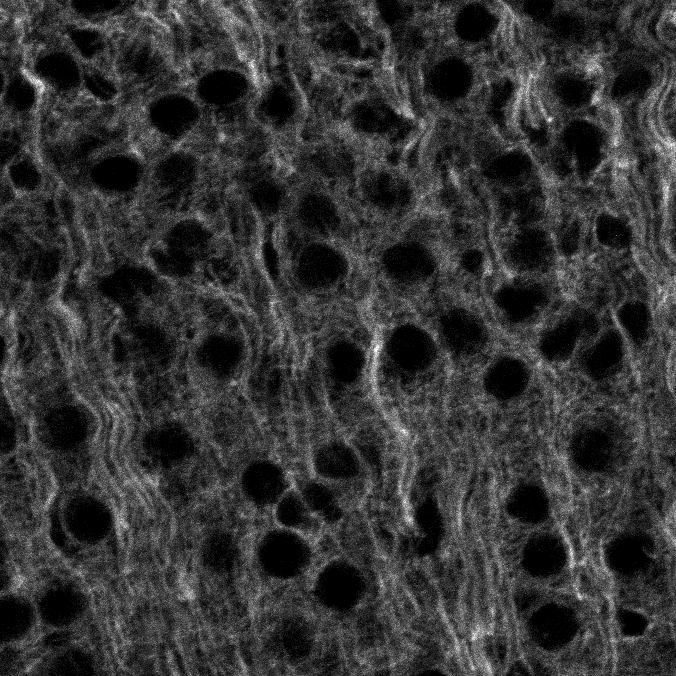

Supplement: Supplementary file 12 — Appendix Source Data [file 44318_2024_50_MOESM12_ESM.zip › Appendix-source files/Figure S7/C2-MAX_exp419-R-mouse3-Tubulin-PolyE-slice3.tif]

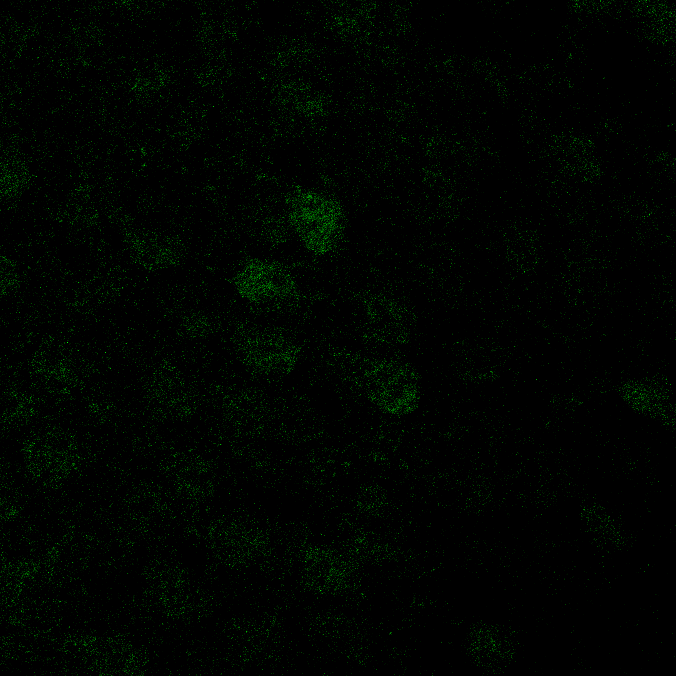

Supplement: Supplementary file 12 — Appendix Source Data [file 44318_2024_50_MOESM12_ESM.zip › Appendix-source files/Figure S7/C1-MAX_exp401-P7-L1-AceT647-PolyET555-slice4.tif]

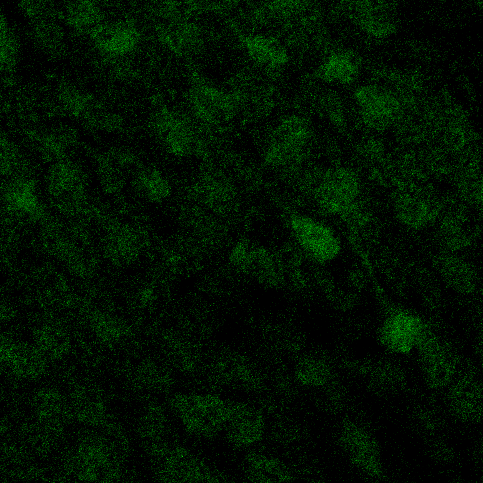

Supplement: Supplementary file 12 — Appendix Source Data [file 44318_2024_50_MOESM12_ESM.zip › Appendix-source files/Figure S7/C1-MAX_exp401-P7-L2-Tub647-PolyET555-slice4.tif]

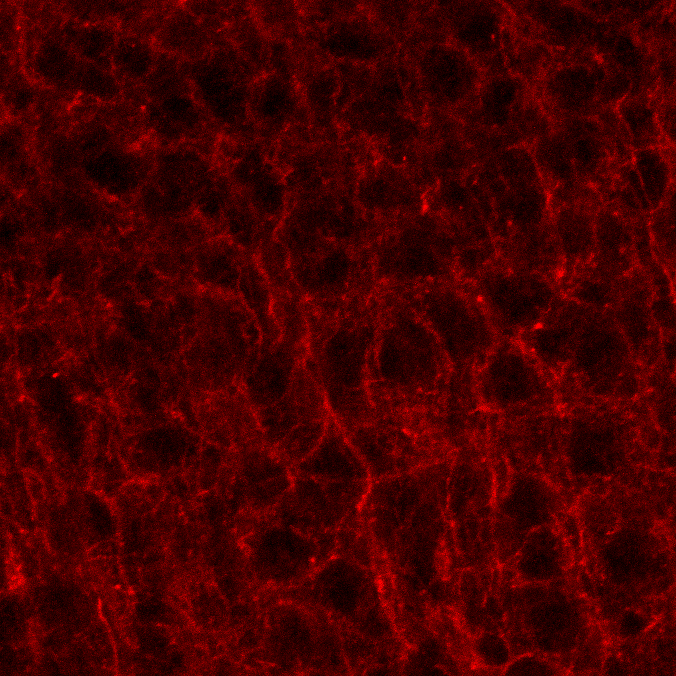

Supplement: Supplementary file 12 — Appendix Source Data [file 44318_2024_50_MOESM12_ESM.zip › Appendix-source files/Figure S7/C4-MAX_exp419-R-mouse3-Tubulin-PolyE-slice3.tif]

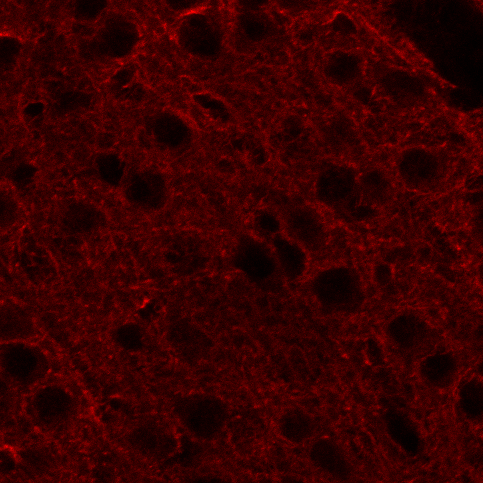

Supplement: Supplementary file 12 — Appendix Source Data [file 44318_2024_50_MOESM12_ESM.zip › Appendix-source files/Figure S7/C4-MAX_exp401-P7-L2-Tub647-PolyET555-slice4.tif]

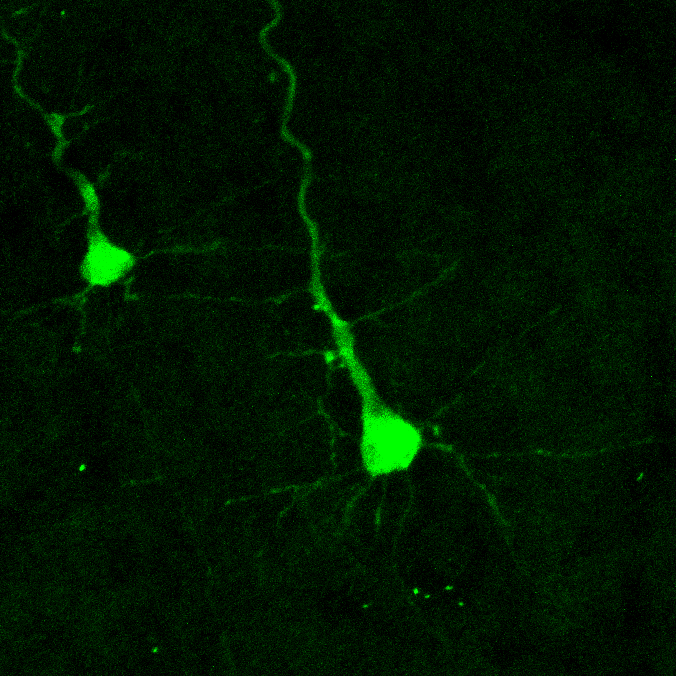

Supplement: Supplementary file 12 — Appendix Source Data [file 44318_2024_50_MOESM12_ESM.zip › Appendix-source files/Figure S7/C1-MAX_exp419-R-mouse3-Tubulin-PolyE-slice3.tif]

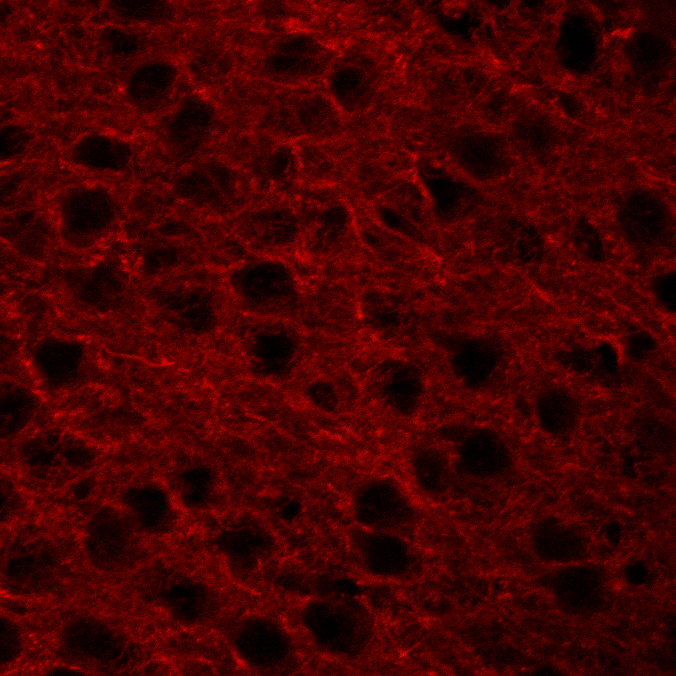

Supplement: Supplementary file 12 — Appendix Source Data [file 44318_2024_50_MOESM12_ESM.zip › Appendix-source files/Figure S7/C4-MAX_exp401-P7-L1-AceT647-PolyET555-slice4.tif]

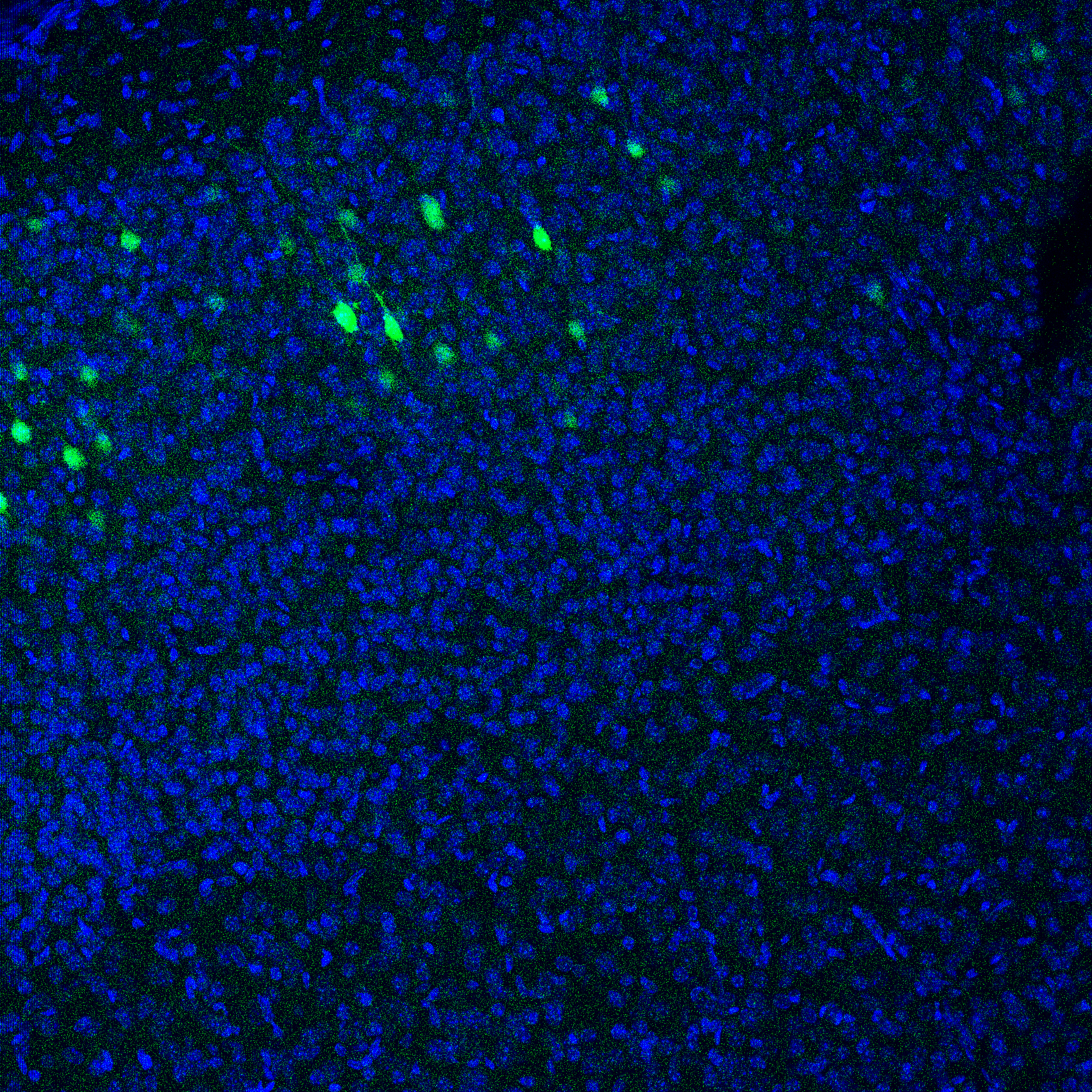

Supplement: Supplementary file 12 — Appendix Source Data [file 44318_2024_50_MOESM12_ESM.zip › Appendix-source files/Figure S7/Appendix Figure S7C-control.tif]

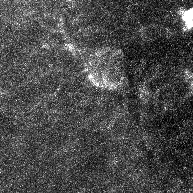

Supplement: Supplementary file 12 — Appendix Source Data [file 44318_2024_50_MOESM12_ESM.zip › Appendix-source files/Figure S9/MAP1B+TTL-DN-inset-FLAG-1.tif]

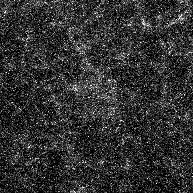

Supplement: Supplementary file 12 — Appendix Source Data [file 44318_2024_50_MOESM12_ESM.zip › Appendix-source files/Figure S9/MAP1B+TTL-inset-FLAG-1.tif]

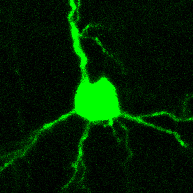

Supplement: Supplementary file 12 — Appendix Source Data [file 44318_2024_50_MOESM12_ESM.zip › Appendix-source files/Figure S9/MAP1B+TTL-inset-GFP-1.tif]

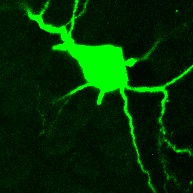

Supplement: Supplementary file 12 — Appendix Source Data [file 44318_2024_50_MOESM12_ESM.zip › Appendix-source files/Figure S9/MAP1B+TTL-DN-inset-GFP-1.tif]
