# Supplementary material for: Microtubule-binding protein MAP1B regulates interstitial axon branching of cortical neurons via the tubulin tyrosination cycle
Source: EMBO J. 2024 Feb 22;43(7):5. doi: 10.1038/s44318-024-00050-3 (PMC10987652; doi:10.1038/s44318-024-00050-3)
Supplement: Supplementary file 14 — Expanded View Figures [file 44318_2024_50_MOESM14_ESM.pdf]

# Expanded View Figures

**Figure EV1. GSK3β regulates neuronal morphology in layer 2/3 CPNs (related to Fig. 1).**

(**A**) Overview of original candidate molecule screen (Dorskind et al, 2023) for identification of CPN interstitial axon branching regulators. (**B**) Overexpression of human constitutively active GSK3β (GSK3β-CA) induces ectopic interstitial axon branching in layer 2/3 CPNs. Scale bars: 100 µm. (**C**) Additional changes in axonal morphology induced by human GSK3β-CA include axonal loops and spiny protrusions along axons (arrowheads). Scale bars: 100 µm (including callosal loop and protrusions, right), 50 µm (primary axon loop, bottom right). (**D**) GSK3β-CA-induced interstitial axon branching (arrows) in layer 2/3 CPNs persist until adulthood. Scale bars: 100 µm. (**E**) Example of a severe loss of interstitial axon branches following removal of both *Gsk3α* and *Gsk3β* isoforms in the mouse (related to Fig. 1D). Scale bars: 100 µm. (**F**) Quantification of axon interstitial branching in *GSK3*$\alpha^{-/-}$;$\beta^{+/+}$ mutant mice. Controls are the same as those shown in Fig. 1D. (**G**) Basal dendrite patterning in layer 2/3 CPNs is regulated by GSK3 isoforms. Red dotted line (placed at right angle to the apicobasal orientation of a neuron) highlights the region where dendritic intersections were calculated. Scale bars: 50 µm. (**H**) Quantification of dendritic intersections from (**G**). Each column represents a single animal; mean ± SD is plotted. *GSK3*$\alpha^{+/-}$;$\beta^{fl/fl}$ $n = 3$ mice, *GSK3*$\alpha^{-/-}$;$\beta^{fl/fl}$ $n = 4$ mice, *$p < 0.05$, *t*-test. Source data are available online for this figure.

© The Author(s)

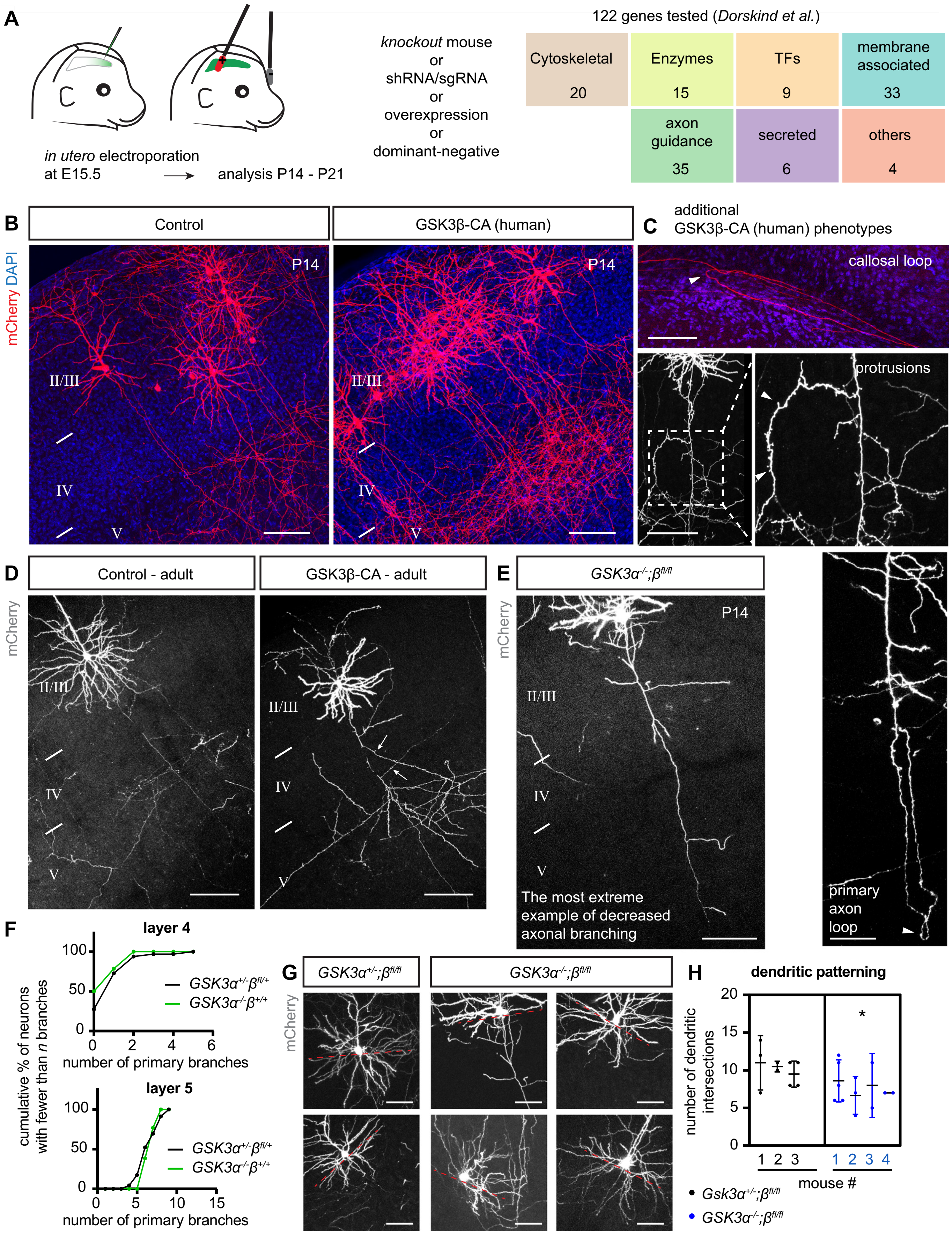
A
in utero electroporation at E15.5
analysis P14 - P21
knockout mouse or shRNA/sgRNA or overexpression or dominant-negative
122 genes tested (Dorskind et al.)
Cytoskeletal 20
Enzymes 15
TFs 9
membrane associated 33
axon guidance 35
secreted 6
others 4
B
Control
GSK3β-CA (human)
mCherry DAPI
P14
II/III
IV
V
C
additional GSK3β-CA (human) phenotypes
callosal loop
protrusions
D
Control - adult
GSK3β-CA - adult
mCherry
E
GSK3α-/-;βfl/fl
P14
The most extreme example of decreased axonal branching
primary axon loop
F
layer 4
layer 5
cumulative % of neurons with fewer than n branches
number of primary branches
GSK3α+/-β fl/+
GSK3α-/-β+/+
G
GSK3α+/-;βfl/fl
GSK3α-/-;βfl/fl
H
dendritic patterning
number of dendritic intersections
mouse #
Gsk3α+/-;βfl/fl
GSK3α-/-;βfl/fl

 © The Author(s)

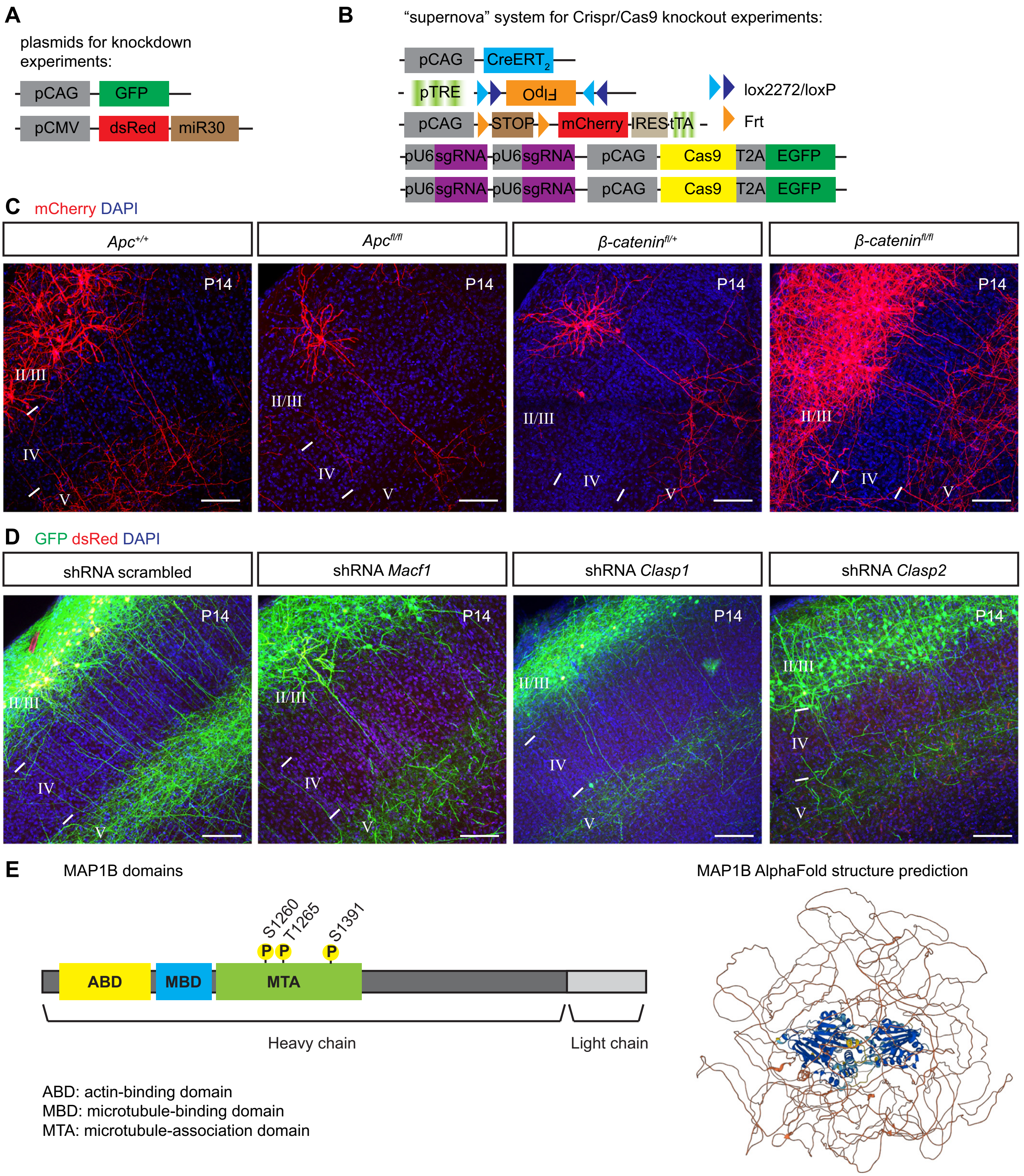
A
plasmids for knockdown experiments:
pCAG GFP
pCMV dsRed miR30
B
“supernova” system for Crispr/Cas9 knockout experiments:
pCAG CreERT2
pTRE Flpo
lox2272/loxP
pCAG STOP mCherry IRES tTA
Frt
pU6 sgRNA pU6 sgRNA pCAG Cas9 T2A EGFP
pU6 sgRNA pU6 sgRNA pCAG Cas9 T2A EGFP
C
mCherry DAPI
Apc+/+
Apcfl/fl
β-cateninfl/+
β-cateninfl/fl
P14
II/III
IV
V
D
GFP dsRed DAPI
shRNA scrambled
shRNA Macf1
shRNA Clasp1
shRNA Clasp2
P14
II/III
IV
V
E
MAP1B domains
S1260
T1265
S1391
P
ABD
MBD
MTA
Heavy chain
Light chain
ABD: actin-binding domain
MBD: microtubule-binding domain
MTA: microtubule-association domain
MAP1B AlphaFold structure prediction

**Figure EV2. Candidate survey for GSK3β targets that regulate interstitial axon branching (related to Fig. 2).**

(**A**) Experimental approach for shRNA-mediated knockdown experiments. (**B**) Experimental approach for CRISPR/Cas9-mediated knockout experiments. (**C**) Removal of *Apc* or β-*catenin* using conditional knockout mice does not influence interstitial axon branching in layer 4. Scale bars: 100 µm. (**D**) Knockdown of *Macf1*, *Clasp1* or *Clasp2* does not influence interstitial axon branching in layer 4. Scale bars: 100 µm. (**E**) Domain structure of MAP1B protein and an AlphaFold structure prediction model (AF-P14873-F1). Note that the confidence provided by the AlphaFold algorithm model is very low for MAP1B. Source data are available online for this figure.

 © The Author(s)

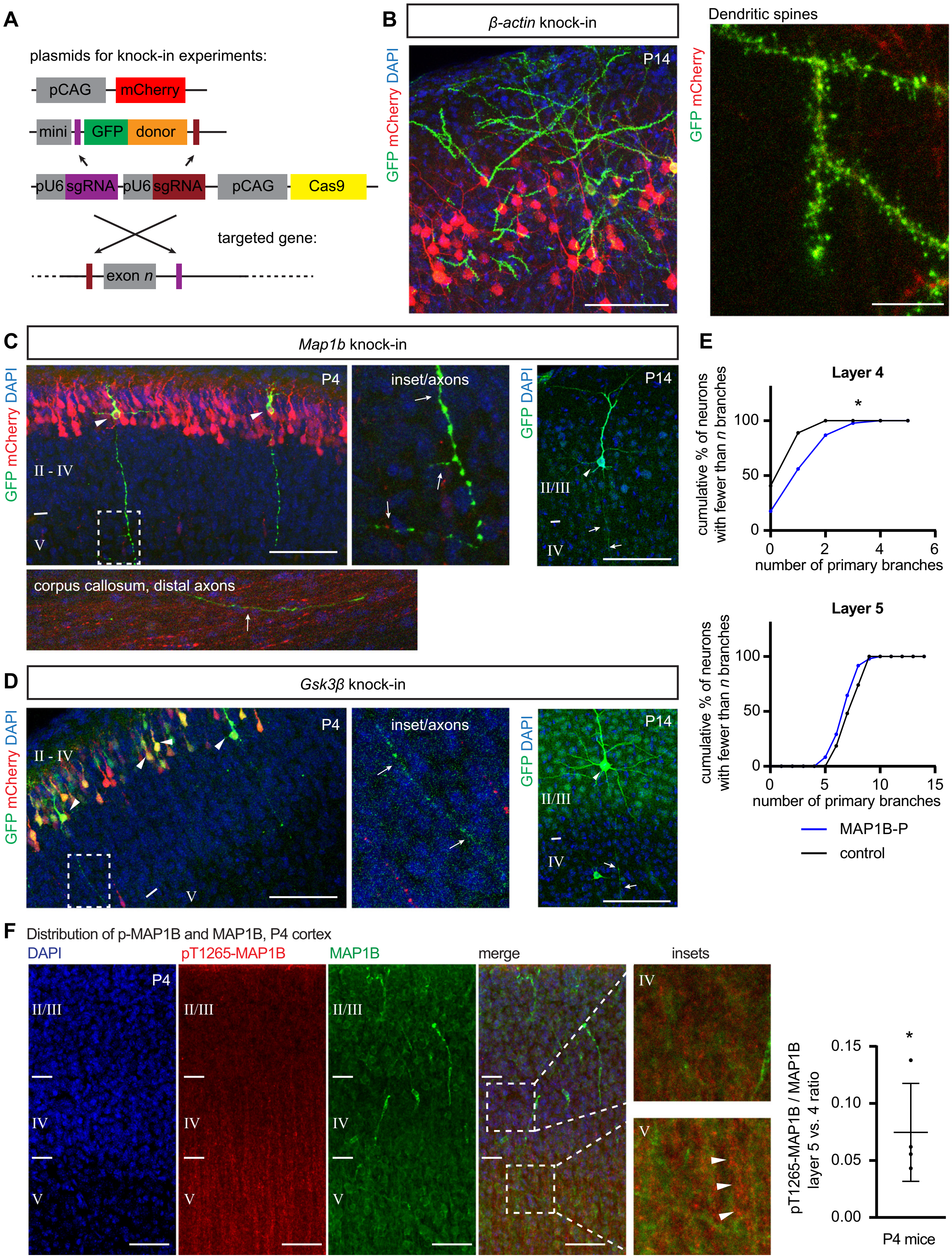
A
plasmids for knock-in experiments:
pCAG
mCherry
mini
GFP
donor
pU6
sgRNA
pU6
sgRNA
pCAG
Cas9
targeted gene:
exon n
B
β-actin knock-in
GFP mCherry DAPI
P14
Dendritic spines
GFP mCherry
C
Map1b knock-in
GFP mCherry DAPI
P4
II - IV
V
inset/axons
GFP DAPI
P14
II/III
IV
corpus callosum, distal axons
E
Layer 4
cumulative % of neurons with fewer than n branches
number of primary branches
Layer 5
MAP1B-P
control
D
Gsk3β knock-in
GFP mCherry DAPI
P4
II - IV
V
inset/axons
GFP DAPI
P14
II/III
IV
F
Distribution of p-MAP1B and MAP1B, P4 cortex
DAPI
pT1265-MAP1B
MAP1B
merge
insets
P4
II/III
IV
V
pT1265-MAP1B / MAP1B layer 5 vs. 4 ratio
P4 mice

**Figure EV3. Analysis of the GSK3β target MAP1B subcellular localization in cortical neurons using CRISPR/Cas9 endogenous tagging (related to Figs. 2 and 3).**

(**A**) Experimental design for endogenous tagging experiments using CRISPR/Cas9 knock-in approach. (**B**) Example of endogenous GFP-β-actin tagging. Note accumulation of GFP in actin-rich dendritic spines (dendritic spines, right). Scale bars: 100 µm (left), 10 µm (dendritic spines). (**C**, **D**) Representative images showing endogenous tagging of *Map1b* and *Gsk3*β at P4 or P14 cortices (arrowheads and arrows highlight expression in somatodendritic compartments and axons, respectively). Note strong expression of MAP1B, which fills entire neuron, including the most distal axonal compartments in the corpus callosum (panel **C**, bottom). Insets in C and D show axonal expression. Scale bars: 100 µm. (**E**) Overexpression of MAP1B-P leads to ectopic interstitial axon branching in layer 4. For this analysis, control neurons from Fig. 2E were used. (**F**) Phosphorylation of MAP1B in the cortex. Cortical slices from P4 mice were probed with phopsho-MAP1B (pT1265-MAP1B, red) and MAP1B (green) antibodies. Phosphorylated MAP1B is enriched in cortical layer 5. Note the punctate pattern in bundled structures in layer 5 (insets, arrowheads). Right: pT1265-MAP1B/MAP1B intensity in the cortical layer 4 and layer 5 was compared using a Paired t test and is presented as a layer 5/layer 4 ratio (individual values from $n = 4$ mice, together with mean ± S.D. are plotted). *$p < 0.05$, t-test. Scale bar: 50 µm. Source data are available online for this figure.

 © The Author(s)

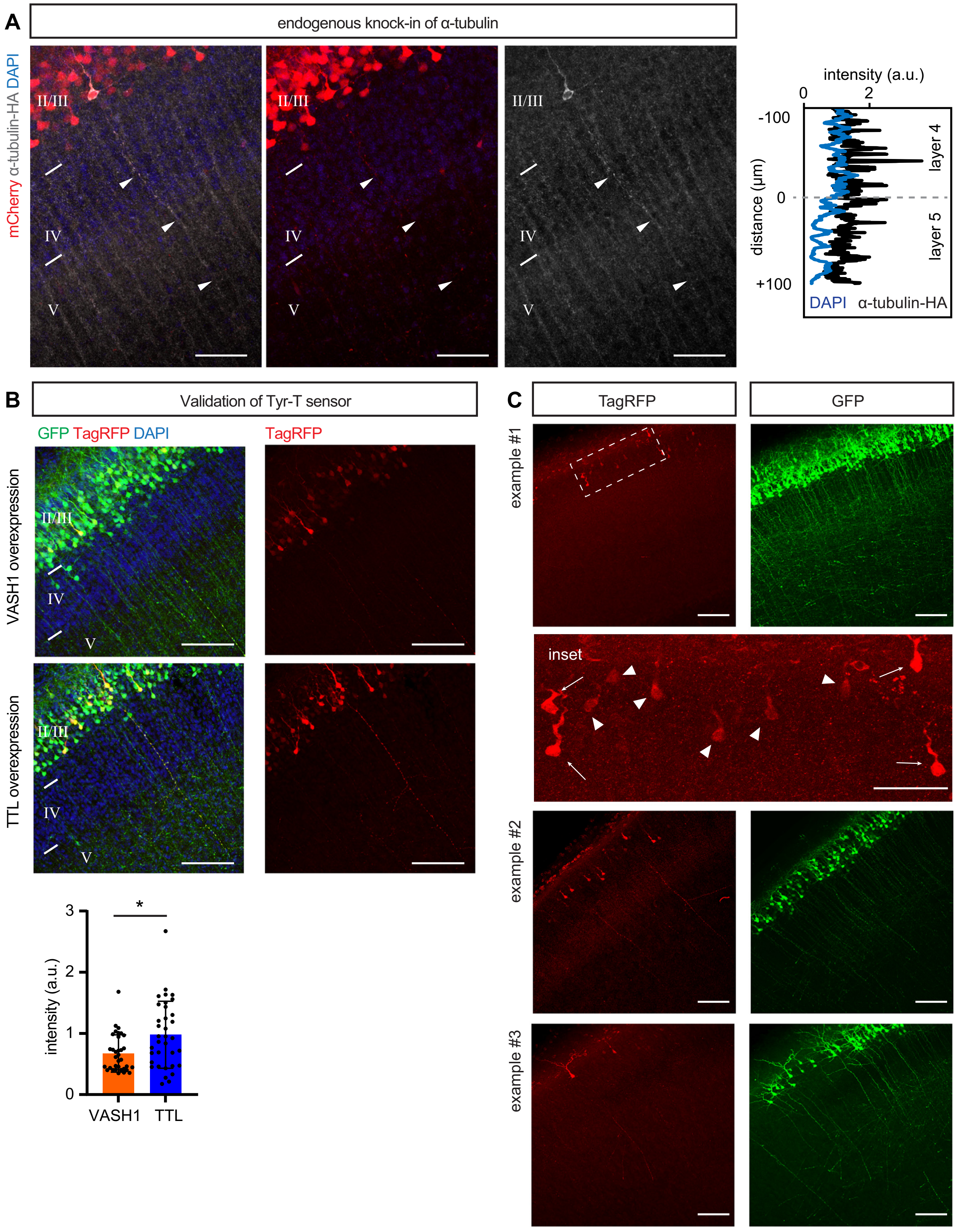
A
endogenous knock-in of α-tubulin
mCherry α-tubulin-HA DAPI
II/III
IV
V
intensity (a.u.)
0
2
-100
0
+100
distance (µm)
layer 4
layer 5
DAPI α-tubulin-HA
B
Validation of Tyr-T sensor
GFP TagRFP DAPI
TagRFP
VASH1 overexpression
TTL overexpression
intensity (a.u.)
3
2
1
0
*
VASH1 TTL
C
TagRFP
GFP
example #1
inset
example #2
example #3

**Figure EV4. Distribution of total and tyrosinated α-tubulin in S1 neocortex (related to Fig. 4).**

(**A**) Example of endogenous α-tubulin-HA tagging (exon 1). Note equal distribution of HA within the axon (arrowheads). Sample trace on the right demonstrates fluorescence intensity of DAPI (a measure of cortical layer) and HA. Scale bars: 50 μm. (**B**) Validation of the Tyr-Tubulin sensor. Neurons were electroporated with the sensor plasmids together with TTL-IRES-GFP or VASH1-IRES-GFP plasmids. Overexpression of VASH1 decreases the fluorescence of the sensor in comparison with TTL, demonstrating its' feasibility. Data shown at the bottom of this panel are presented as mean ± st.d. TTL $n = 34$ neurons, VASH1 $n = 35$ neurons $^*p < 0.05$, *t*-test. Scale bars: 100 μm. (**C**) Examples of a fluorescence intensity distributions after targeting *Rosa26* locus via the TKIT approach (see main text). In the detail for the example #1, note that a small population of neurons expresses high levels of TagRFP (arrows), while a larger population of neurons expresses lower levels of TagRFP (arrowheads). Scale bar: 100 μm, 50 μm (inset). Source data are available online for this figure.

 © The Author(s)

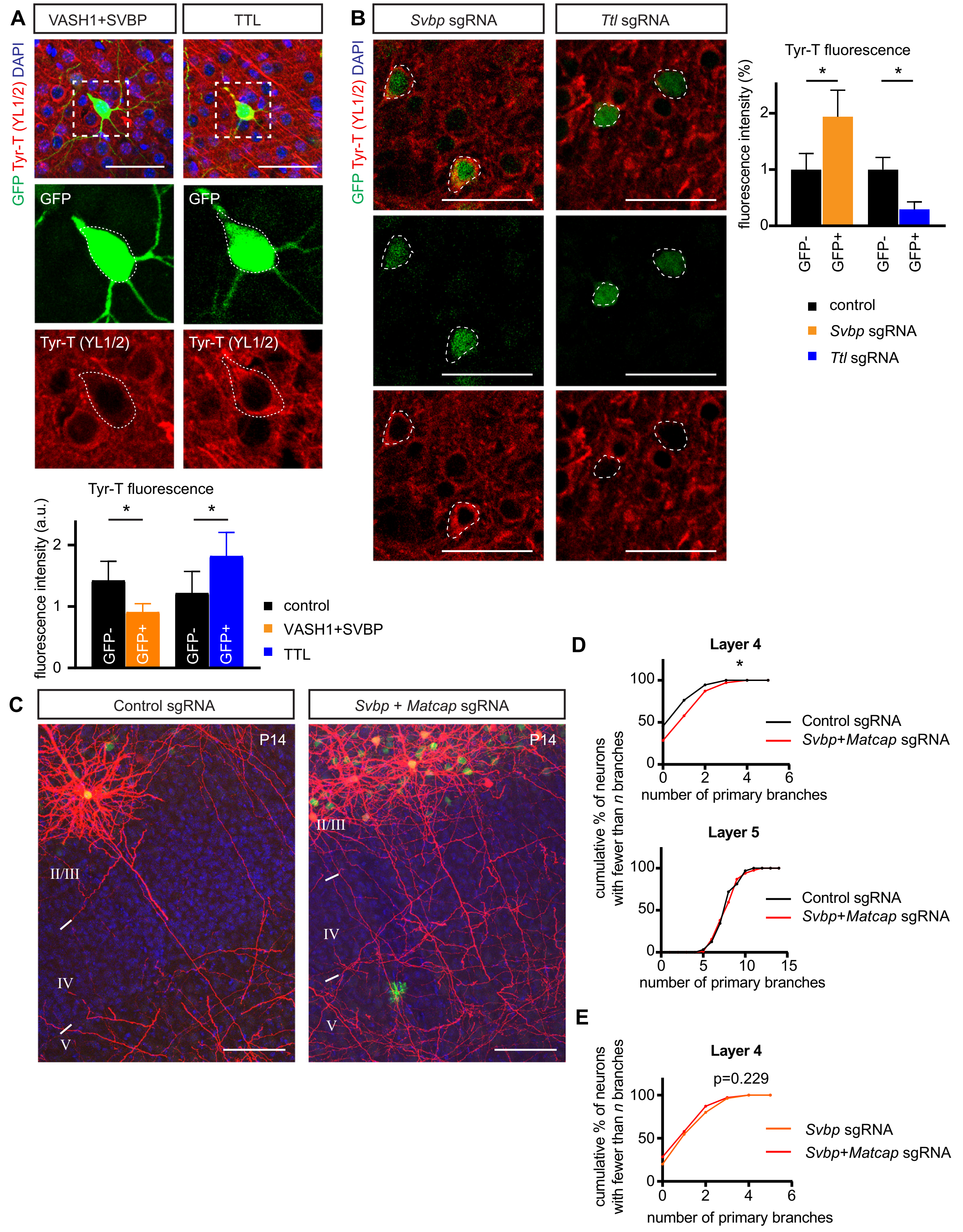
A
VASH1+SVBP
TTL
GFP Tyr-T (YL1/2) DAPI
GFP
Tyr-T (YL1/2)
Tyr-T fluorescence
fluorescence intensity (a.u.)
GFP-
GFP+
control
VASH1+SVBP
TTL
B
Svbp sgRNA
Ttl sgRNA
Tyr-T fluorescence
fluorescence intensity (%)
control
Svbp sgRNA
Ttl sgRNA
C
Control sgRNA
Svbp + Matcap sgRNA
P14
II/III
IV
V
D
Layer 4
Layer 5
cumulative % of neurons with fewer than n branches
number of primary branches
Control sgRNA
Svbp+Matcap sgRNA
E
Layer 4
p=0.229
Svbp sgRNA
Svbp+Matcap sgRNA

**Figure EV5. Tyrosination of α-tubulin in S1 neocortex under normal and experimental conditions (related to Figs. 4 and 5).**

(**A**) Immunostaining of Tyr-T after TTL and VASH1/SVBP overexpression experiments. Note lack of a tyrosination signal following VASH1/SVBP overexpression and an increase in the tyrosination signal after TTL overexpression. Data shown at the bottom are presented as mean ± st.d. TTL control $n = 10$ neurons, TTL overexpression $n = 3$ neurons, SVBP control $n = 12$ neurons, SVBP overexpression $n = 4$ neurons. *$p < 0.05$, *t*-test. Scale bar: 50 μm. (**B**) Immunostaining of Tyr-T following CRISPR knockdown. Note decrease of the tyrosination signal after TTL deletion and an increase in the signal after SVBP deletion. GFP labels electroporated cells that harbor the pX458 plasmid (cells with a dashed contour). Quantification of the tyrosination signal in targeted ($GFP^+$) cells, normalized to $GFP^-$ cells from the same slices, is shown at the bottom. Data are presented as mean ± st.d. TTL control $n = 18$ neurons, TTL deletion $n = 21$ neurons, SVBP control $n = 21$ neurons, SVBP deletion $n = 22$ neurons. *$p < 0.05$, *t*-test. Scale bar: 50 μm. (**C**, **D**) Simultaneous removal of *Svbp* and *Matcap* promotes interstitial axon branching to the same extent as *Svbp* deletion alone. Data analysis and presentation are as in Fig. 1. *$p < 0.05$, *t*-test. Scale bars: 100 μm. (**E**) Comparison of *Svbp* knockdown with combined *Svbp*+*Matcap* knockdown. *t*-test. Scale bars: 100 μm. Source data are available online for this figure.


© The Author(s)
